# Interrelationships of Changes in Outcome Domains in Patients With Schizophrenia Spectrum Disorders: A Meta‐Analysis

**DOI:** 10.1111/acps.13808

**Published:** 2025-03-30

**Authors:** Lars de Winter, Auke Jelsma, Jentien M. Vermeulen, Astrid Vellinga, Jaap van Weeghel, Ilanit Hasson‐Ohayon, Cornelis L. Mulder, Nynke Boonstra, Wim Veling, Lieuwe de Haan

**Affiliations:** ^1^ Phrenos Center of Expertise Utrecht the Netherlands; ^2^ Department of Psychiatry Amsterdam UMC Location AMC Amsterdam the Netherlands; ^3^ Mental Health Arkin Amsterdam the Netherlands; ^4^ Tranzo, Tilburg University Tilburg the Netherlands; ^5^ Department of Psychology Bar‐Ilan University Ramat‐Gan Israel; ^6^ Epidemiological and Social Psychiatric Research Institute, Erasmus MC Rotterdam the Netherlands; ^7^ Parnassia Psychiatric Institute the Netherlands; ^8^ NHL Stenden University of Applied Science Leeuwarden the Netherlands; ^9^ University Medical Center Utrecht Utrecht the Netherlands; ^10^ University of Groningen, University Medical Center Groningen Groningen the Netherlands

## Abstract

**Introduction:**

Patients with schizophrenia spectrum disorders (SSD) improve in several outcome domains over the course of illness, but to different degrees. In this meta‐analysis, we investigated whether longitudinal changes in different outcome domains are associated with each other and which factors moderate these changes over time.

**Methods:**

Our protocol was preregistered in PROSPERO (CRD42024504253). We included 109 studies, identified through searches in PsycInfo, PubMed, CINAHL, and Cochrane up until November 2023, investigating longitudinal changes in at least two outcome domains (symptoms, social functioning, cognition or personal recovery) for patients with SSD with at least 1 year follow‐up. We calculated Pearson correlation coefficients for associations of changes between outcome domains. Potential moderating effects of demographic, clinical, social, or study characteristics were explored. Quality assessment was executed using the QUIPS tool.

**Results:**

We found substantial positive associations between changes in symptoms, social functioning, and cognition. Especially, changes in negative symptoms and overall social functioning were associated with changes in several outcome domains. Changes in personal recovery were only associated with changes in symptoms. We found more substantial improvements in combinations of outcomes for patients with a shorter illness duration, females, a lower percentage of patients diagnosed with schizophrenia, and patients receiving treatment focused on targeted outcomes.

**Conclusions:**

Symptoms, social functioning, and cognition often concurrently improve and may boost each other. This suggests that an integrated approach targeting several outcome domains jointly boosts long‐term improvement. However, changes in personal recovery seem to occur separately from other outcome domains. Therefore, targeted attention for personal recovery is needed.

**Trial Registration:** PROSPERO: CRD42024504253

**Summary**

- Summations
  ○ Longitudinal changes in symptoms, social functioning, and cognition are interrelated, and therefore concurrently improve in patients with schizophrenia spectrum disorder.
  ○ Longitudinal changes in personal recovery are only to a small extent related to changes in other recovery domains, and therefore might be driven through other processes than symptoms, social functioning, and cognition.
  ○ Larger improvements in multiple recovery domains were achieved by patients with a short duration of illness, female gender, a lower percentage of patients diagnosed with schizophrenia, or patients receiving treatment that focused on targeted outcomes.
- Limitations
  ○ Due to our strict selection criteria, we may have missed important studies that might have added knowledge.
  ○ We exclusively included studies investigating diagnosed patients that are known in mental health care settings. As a consequence, we miss out on patients who might receive support outside of mental healthcare, as well as patients who have already finalized treatment and patients who are not capable to participate in research.
  ○ Included studies were executed in different cultural, clinical, and social contexts and used a wide variety of assessment instruments. Therefore, the results of our meta‐analysis are heterogeneous.

## 1 Introduction

Schizophrenia spectrum disorders (SSD) are characterized by distortions in thinking and perception, cognitive impairments, avolition, apathy, and restricted affective expression [1, 2]. In addition to these symptoms, SSD is also known to affect personal, social, and functional life domains [3, 4, 5, 6].

In the current meta‐analysis, we investigate four main outcome domains: social functioning, symptoms, cognition, and personal recovery. Improvement in these domains during the course of SSD varies substantially. In a series of previous meta‐analyses, we found indications of large improvements in positive symptoms, disorganization symptoms, and general functioning, specifically for patients with a short illness duration. In other domains of symptoms and social functioning, we found small improvements over time regardless of illness duration [7, 8]. In contrast, for cognition and personal recovery, we only found marginal improvements over time, with specifically for cognition slightly better improvement for patients with a short illness duration [9, 10]. Changes in those latter outcome domains were favorably influenced by studies investigating patients with a short duration of untreated psychosis, a young age, a high education level, provision of psychosocial rehabilitation, a low prevalence of patients with a schizophrenia diagnosis, and at baseline: low symptom severity, high levels of cognition, high levels of quality of life, and a high level of personal recovery [6, 7, 8, 9, 10, 11, 12, 13].

Improvement in different outcome domains may be related to each other. Previous studies already indicated cross‐sectional associations between social functioning, symptoms, cognition, and personal recovery [3, 14, 15, 16]. However, insights into associations between longitudinal changes in those outcome domains are currently lacking, as well as during which phases of SSD these associations predominantly take place.

## 2 Aims of the Study

The aim of the current meta‐analysis is to investigate interrelationships of changes between different recovery domains within subgroups based on the patients' duration of illness (DOI). We also investigated which personal, clinical, social and contextual characteristics of patients with SSD are associated with larger improvement in combinations of outcome domains. This adds knowledge concerning which outcome domains do concurrently improve during which phase of SSD. We aimed to answer the following questions: (1) To what extent are changes in social functioning, symptoms, cognition or personal recovery interrelated over the course of SSD? (2) Which moderators at baseline (especially DOI) are associated with favorable changes in combinations of these outcome domains over time?

## 3 Methods

We followed PRISMA guidelines [17]. The PRISMA checklist is presented in Supporting Information 1. Our protocol was preregistered in PROSPERO.

### 3.1 Data Sources

Records were identified through searches in PsycInfo, PubMed, CINAHL, and Cochrane of peer‐reviewed journals until November 2023. We used terms related to the study sample (e.g., psychosis), the study design (e.g., course, longitudinal) and outcomes (e.g., symptoms, cognition, social functioning, personal recovery); all search terms are presented in Supporting Information 2. Additional references were traced through reference tracking of included studies and systematic reviews.

### 3.2 Eligibility Criteria

Four assessors (L.d.W., K.K., R.M., and A.J.) independently selected studies. Disagreements were resolved by consensus. The included studies meet the following criteria:

1. Patient population:
  Studies including adults (mean age ≥ 18) who are all diagnosed with SSD following DSM or ICD classifications [18] were included. Studies describing patients younger than 18 years or with another main classification than SSD were excluded.
2. Study design:
  Longitudinal cohort studies or randomized controlled trials, with a follow‐up duration of at least 1 year, were included. Other study designs were excluded.
3. Outcomes:
  Studies reporting uncorrected quantitative assessments, reported at least at two time points, of a minimum of two of the four main outcome domains of symptoms, social functioning, personal recovery, and cognition were included. Qualitative studies were excluded.
4. Publication:
  Exclusively studies published in English in peer‐reviewed journals were included.

### 3.3 Outcome Domains

We focused on four main outcome domains: social functioning, symptoms, personal recovery, and cognition. Each outcome domain was divided into subdomains, following the categorization process in our previous meta‐analyses [7, 8, 9, 10], if they meet the following additional criteria: (1) being reported by at least 20 included studies; (2) the outcome domain showed at least a small significant improvement or deterioration in previous meta‐analyses to secure sufficient variability. This resulted in 14 outcome domains to be addressed in this meta‐analysis, described below and in Table 1.

- *Social functioning*: (1) overall social functioning; (2) prosocial behavior; (3) independence; (4) vocational functioning.
- *Symptoms*: (1) positive symptoms; (2) negative symptoms; (3) disorganization symptoms; (4) depressive symptoms.
- *Personal recovery*: (1) personal recovery; (2) subjective quality of life.
- *Cognition*: (1) verbal memory; (2) executive functioning; (3) processing speed; (4) overall cognition.

**TABLE 1:** Overview and definitions of outcome domains.

Social functioning		
Outcome domain	Definition	Examples of assessment instruments^a^
Overall social functioning	Outcomes measuring overall functioning in any social setting or role, based on clinician‐rated functional outcome scales and standardized composite scores clustering different domains of societal functioning.	Functional Skills Rating Form (FSRF); Global Assessment of Functioning (GAF); Personal and Social Performance scale (PSP); Social Functioning Scale (SFS); Social and Occupational Functioning Assessment Scale (SOFAS); World Health Organization's Disability Assessment Scale (WHO‐DAS)
Prosocial behavior	The level of social skills, relationships or social adaptive behavior	Assessment of Interpersonal Problem‐Solving Skills (AIPSS); Role functioning scale (RFS); Social behavior scale (SBS); Social Skills Performance Assessment (SSPA); Subscale scores of assessment instruments reported under overall social functioning.
Independence	The level of independence and independent behavior of the client including self‐care, independent living and financial management.	Alzheimer's Disease Assessment Scale—Late Version (ADAS‐L); Days independent living; Monthly income; Subscale scores of assessment instruments reported under overall social functioning.
Vocational functioning	Involvement into (competitive) employment and education, measured by percentages of participants involved into work or education and outcome scales related to vocational functioning and involvement	Employment rate; Number of hours worked; Subscale scores of assessment instruments reported under overall social functioning.

Symptoms		
Outcome domain	Definition	Examples of assessment instruments^a^
Positive symptoms	Assessments of overall levels of positive symptoms or specific aspects of positive symptoms (e.g., hallucinations or delusions).	Brief Psychiatric Rating Scale (BPRS); Positive and Negative Symptom Scale (PANSS); Scale for the Assessment of Positive symptoms (SAPS)
Negative symptoms	Assessments of overall levels of negative symptoms or specific aspects of negative symptoms (e.g., social withdrawal or apathy).	Brief Psychiatric Rating Scale (BPRS); High Ryods Evaluation of Negativity (HEN) Scale; Positive and Negative Symptom Scale (PANSS); Scale for the Assessment of Negative symptoms (SANS)
Disorganization symptoms	Assessments of symptoms of disorganization.	Brief Psychiatric Rating Scale (BPRS); Positive and Negative Symptom Scale (PANSS); Scale for the Assessment of Positive symptoms (SAPS)
Depressive symptoms	Assessments of symptoms of depression or prevalence of comorbid mood disorders.	Brief Psychiatric Rating Scale (BPRS); Calgary Depression Rating Scale (CDRS); Hamilton Rating Scale for Depression (HRSD); Montgomery–Asberg Depression Rating Scale (MADRS)

Personal recovery and quality of life		
Outcome domain	Definition	Examples of assessment instruments^a^
Personal recovery	Outcomes capturing total scores of assessment instruments focused on overall personal recovery, or at least one domain of personal recovery from the CHIME framework: Connectedness, Hope and Optimism, Identity, Meaning in Life or Empowerment	Adult Trait Hope Scale (ATHS); Client's Assessment of Strengths, Interests, and Goals (CASIG); Questionnaire About the Process of Recovery (QPR); Recovery Assessment Scale (RAS); Resilience Scale for Adults (RSA)
Subjective quality of life	Outcomes that comprise multiple domains of subjective quality of life or total scores of assessment instruments focused on overall subjective quality of life.	Heinrichs quality of life scale; Lehman's quality of life scale; Manchester Short Assessment of Quality of Life (MANSA); World Health Organization Quality of Life Scale (WHO‐QOL 26)

Cognition		
Outcome domain	Definition	Examples of assessment instruments^a^
Verbal memory	Short‐ and long‐term verbal memory tasks and its recognition.	California Verbal Learning Task (CVLT); Digit span test; Hopkins Verbal Learning Test (HVLT); Rey Auditory Verbal Learning Task (RAVLT)
Executive functioning	The set of processes that manifest control over other component cognitive abilities, such that cognitive resources can be effectively utilized to solve problems efficiently and plan for the future. Thus, tasks of problem solving, working memory tasks, planning, manipulating mazes, and other complex tasks where management of multiple cognitive abilities are required.	Category verbal fluency test; Stroop time color‐word inference test; Trail Making Test B; Wisconsin Card Sorting Test (WCST)
Processing speed	Cognitive processing assessments that require rapid performance of tasks that range from very simple to complex.	Color Trial Line 2; Digit symbol test; MATRICS Consensus Cognitive Battery (MCCB) Speed of processing; Trail making test A
Overall cognition	Overall cognition composite scores, or assessments of intelligence that is not categorized in any domains.	Brief Assessment of Cognition in Schizophrenia (BACS); Comprehensive Module Test; MATRICS Consensus Cognitive Battery (MCCB); Mini Mental State Examination (MMSE); Wechsler Adult Intelligence Scale (WAIS)

^a^ Operationalizations of all outcomes reported in our included studies are presented in Supporting Information 2.

The outcome domain of personal recovery included both personal recovery and subjective quality of life (S‐QOL), because both personal recovery and S‐QOL show considerable conceptual overlap and comparable patterns of change [10, 19].

Definitions and operationalizations of each outcome domain are presented in Table 1 and Supporting Information 3.

### 3.4 Data‐Extraction and Synthesis

Data‐extraction took place between January and May 2024. We describe data‐extraction and synthesis below.

#### 3.4.1 Duration of Illness Subgroups

We categorized study outcomes in three subgroups based on the mean baseline DOI of the study sample: (1) DOI < 5 years; (2) DOI 5–10 years; (3) DOI > 10 years. This categorization was based on previously published meta‐analyses [7, 8, 9, 10] and a landmark study that investigated clinical stages in SSD [20].

#### 3.4.2 Assessing Effect Sizes of Change

We analyzed changes in our outcome domains by calculating effect sizes of change (Cohen's *d*) between the baseline and follow‐up assessment within each study. These were used as an outcome in our meta‐analysis.

#### 3.4.3 Combinations of Outcomes and Subgroups of Change

We analyzed interrelationships of changes in two outcomes, both reported in at least 10 included studies, by calculating correlations between both outcomes. In case we found significant associations between outcomes, we investigated which moderators influenced changes in combinations of these outcomes. In order to evaluate this, we categorized these combinations into four subgroups:

1. Studies reporting at least moderate improvement (i.e., *d* ≥ 0.5) in both outcome domains;
2. Studies reporting at least moderate improvement in one outcome domain, but low to marginal improvement (i.e., *d* < 0.5) in the other outcome domain;
3. Studies reporting low to moderate improvement in both outcome domains;
4. Studies reporting no significant improvement or deterioration in at least one of both outcome domains.

#### 3.4.4 Assessing Moderators at Baseline

We extracted clinical, personal, social, contextual, and study characteristics (i.e., moderators at baseline) from our included studies through a three‐step process to investigate their influence on changes in combinations of outcomes.

1. We made a preselection of moderators that were also included in one of our four previous meta‐analyses [7, 8, 9, 10].
2. We selected moderators from this preselected long‐list that significantly influenced at least one of our outcome domains in previously published meta‐analyses [5, 7, 8, 9, 10, 13, 21, 22, 23].
3. Moderators were finally included in our analysis if they are reported in at least 10 studies, known as the optimal number to conduct subgroup analyses [24]. This means that in some combinations of outcome domains, not all moderators were analyzed.

This resulted in 31 moderators that were included in our analyses (see Supporting Information 7).

### 3.5 Quality Assessment

We conducted quality assessment using the Quality in Prognostic Studies (QUIPS) tool [25]. The first author (L.d.W.) assessed all studies, and a second assessor (A.J.) independently assessed 10% of the studies. The level of agreement was substantial (*κ* = 0.72). Disagreements were resolved by consensus.

### 3.6 Statistical Analysis

#### 3.6.1 Meta‐Analytic Procedure

Meta‐analyses were conducted using RevMan 5.3 [26]. We used random effects models, weighted by the method of inverse variance [27]. This method gives more weight to larger studies and studies with smaller standard errors. Effect sizes of categorical outcomes were converted into Cohen's *d* [28] enabling homogeneous analysis of all outcomes. For clinical trials, we analyzed both treatment and control group together. Statistical heterogeneity was assessed by calculating the *I* ^2^ statistic [27].

#### 3.6.2 Calculating Interrelationships of Outcomes

Association between changes in two outcome domains was analyzed through Pearson correlations. This is the optimal statistic to analyze linear relationships between two continuous variables. We also analyzed Pearson correlations within the baseline DOI subgroups categorized in Section 3.4.1.

#### 3.6.3 Analyses of Moderating Effects

As described in Section 3.4.3., studies were categorized in four subgroups based on combinations of interrelated outcome domains. We analyzed moderating effects by comparing differences in moderators at baseline between these four subgroups through an analysis of variance (ANOVA) for continuous and a Pearson Chi‐squared analysis for categorical moderators. When the omnibus test was significant we calculated specific subgroup differences using a Tukey's HSD test [29] or an analysis of standardized residuals. We controlled for multiple testing effects in all analyses through a Benjamini‐Hochberg correction, with the false discovery rate set on 0.3 [30].

#### 3.6.4 Handling Outliers and Publication Bias

We controlled for the influence of outliers (i.e., confidence intervals [CI] of individual study outcomes that exceeded the upper or lower bound of CI of the overall effect size) by re‐calculating the associations between effect sizes of change while omitting outliers. Potential publication bias was detected by visual inspection of funnel plots and the ‘trim and fill’ method in which we re‐evaluated effect sizes after removing studies causing funnel‐plot asymmetry and filling with potentially omitted studies through multiple imputations [31].

## 4 Results

### 4.1 Study Flow

Of the 12,304 records primarily retrieved, we excluded 11,296 records after title and abstract screening. Of the remaining 1008 records, we excluded 842 records after full‐text screening. Most of the studies were excluded because the study design was not longitudinal or because the study did not report outcomes of at least two outcome domains (see Supporting Information 4). The remaining 166 articles reported results of 109 studies.

### 4.2 Study Characteristics

Study characteristics are presented in Table 2 and Supporting Information 5. The 109 included studies investigated 19,549 patients with SSD. The mean age at baseline was 34.6 years (SD = 10.5; Range = 20.9–68.7), and 35.7% were female (see Table 2). In 42 studies (38.5%), all patients were diagnosed with schizophrenia, whereas 67 studies (61.5%) investigated patients with various SSD classifications. Forty‐three studies (39.4%) were clinical trials, and 64 studies (58.7%) were cohort studies. Sixty‐seven studies (61.5%) reported information about the provision of specific psychosocial treatments, of which in 27 treatment was specifically focused on the improvement of the investigated outcome. In 44 studies (40.4%), all participants used antipsychotics, whereas in 65 studies (59.6%) only a part did. The baseline DOI of the study sample was less than 5 years in 42 studies (38.5%), 5 to 10 years in 15 studies (13.8%), more than 10 years in 40 studies (36.7%) and unknown in 12 studies (11.0%). The mean follow‐up duration was 2.9 years (SD = 3.5). Seventy‐one studies (65.1%) had a follow‐up duration ≤ 2 years, 25 studies (22.9%) between 2 and 5 years, and 12 studies (11.0%) > 5 years. In 38 studies (34.9%) the attrition rate was low (i.e., < 20%), in 38 studies (34.9%) it was moderate (i.e., 20%–40%), and in 29 studies (26.6%), it was high (i.e., > 40%).

**TABLE 2:** Descriptive statistics of included studies.

Study name^a^	*N* (baseline‐FU)	Primary diagnosis	Baseline DOI (years)	FU duration (years)	Attrition rate	Outcome domains reported
Addington 2000^S1^	80–65	Schizophrenia (100%)	11.2	2.5	18.8%	NEG; OSF; POS; PSB; QOL
Aguilar 2018^S2^	40–40	Schizophrenia (100%)	Unclear	0.8; 1.25	28.3%	NEG; OSF; POS
Albus 2002^S3,S4^	58–58	Schizophrenia (100%)	6.2	2; 5	30.0%	DEP; EXF; NEG; OVC; POS; PRS; VEM
Alphs 2022^S5^	273–112	Schizophrenia (76.9%); Schizophreniform disorder (23.1%)	0.9	0.8; 1.5	38.1%	DEP; NEG; OSF; POS
Breier 2018^S6^	60–60	Schizophrenia (68.3%); schizophreniform disorder (13.3%); Schizoaffective disorder (8.3%); Psychotic disorder NOS (10.0%)	1.4	1	46.7%	NEG; OSF; OVC; POS
Buonocore 2018^S7^	60–60	Schizophrenia (100%)	10.8	5	6.3%	EXF; OVC; PRS; QOL; VEM
Cai 2022^S8^	277–277	Schizophrenia (100%)	17.9	0.5; 2.3	7.6%	DEP; NEG; OSF; OVC; POS
Cechnicki 2017^S9^	80–65	Schizophrenia (100%)	0.8	3; 12	16.3%	NEG; OSF; POS
Chan 2003^S10^	25–25	Schizophrenia (100%)	15.4	0.3; 0.7; 1	16.0%	OSF; PRC
Chan 2018^S11,S12^	148–107	Schizophrenia spectrum disorder (100%)	0.0	3	27.7%	NEG; POS; VOF
Chanpattana 2010^S13^	253–253	Schizophrenia (100%)	13.3	1.6	0.0%	NEG; OVC; POS
Chen 2000^S14^	50–43	Schizophrenia (100%)	23.5	3	14.0%	EXF; NEG; POS
Chen 2005^S15‐S22^	138–88	Schizophrenia (80.7%); schizophreniform disorder (14.0%); schizoaffective disorder (5.4%)	1.5	1; 2; 3	39.2%	DEP; EXF; NEG; POS; VEM; VOF
Chien 2017^S23‐S26^	333–333	Schizophrenia (52.1%); Schizophreniform disorder (12.0%); Schizoaffective disorder (22.8%); Other psychotic disorders (13.2%)	2.6	0.5; 1; 1.5; 2.5	16.4%	IND; NEG; OSF; POS; PRC; PSB
Ciudad 2009^S27‐S28^	1005–375	Schizophrenia (100%)	13.7	1	16.8%	DEP; IND; NEG; OSF; POS; PRC; PSB
Conley 2007^S29^	2228–1167	Schizophrenia (57.2%); schizoaffective disorder (33.6%); other psychotic disorder (9.3%)	21.6	3	4.3%	IND; NEG; OSF; PRC; PSB; QOL; VOF
Cullberg 2002^S30,S31^	120–115	Schizophrenia syndromes (schizophrenia, schizophreniform psychosis and schizoaffective psychosis; 40.8%); non‐schizophrenia syndromes (delusional disorder, brief psychosis and psychotic disorder not otherwise specified (NOS); 59.2%)	0.0	1; 3; 5	30.8%	DEP; NEG; OSF
Dal Santo 2020^S32^	17–17	Schizophrenia (100%)	Unclear	2.9	0.0%	OVC; POS
Dellazizzo 2023^S33^	74–30	Schizophrenia (77.0%); Schizoaffective disorder (23.0%)	16.0	0.5; 1	16.2%	DEP; DIS; NEG; POS; PRC
Dixon 2015^S34‐S36^	65–65	Schizophrenia (66.2%); schizoaffective disorder (13.9%); schizophreniform disorder (6.2%); Psychosis NOS (4.6%); Brief psychotic disorder (1.6%); no diagnosis (3.1%); unknown (4.6%)	< 2	0.5; 1; 1.5; 2	69.2%	NEG; POS; PRC; PSB; QOL; VOF
Ekerholm 2012^S37^	36–36	Schizophrenia (100%)	17.6	4.6	49.3%	EXF; OSF; PRS; VEM
Evensen 2016^S38^	148–148	Schizophrenia (88.5%); schizoaffective disorder (7.5%); Osychosis NOS (2.0%); Delusional disorder (2.1%)	7.2	2	12.2%	DEP; OSF; VOF
Fernandez‐Modamio 2021^S39^	299–188	Schizophrenia or schizoaffective disorder	22.9	0.5; 1	37.1%	IND; NEG; POS; PRC; PSB; QOL
Fond 2018^S40^	549–315	Schizophrenia (75.7%); Schizoaffective disorder (24.3%)	11.0	2	42.0%	DEP; DIS; EXF; NEG; OSF; OVC; POS; PRS; VEM
Foti 2010^S41‐S45^	248–248	Schizophrenia spectrum disorder (100%)	2.4	0.5; 2; 4; 10; 20	28.0%	DEP; DIS; EXF; NEG; OSF; POS; PRS; VEM; VOF
Fowler 2012^S46^	301–257	Schizophrenia (85.0%); Schizoaffective disorder (13.0%); Delusional disorder (2.0%)	10.7	0.25; 1	18.2%	DEP; POS; PRC
Fowler 2018^S47^	148–143	Non‐affective psychosis (100%)	2.1	0.8; 1.3	17.4%	DEP; NEG; PRC; VOF
Galderisi 2020^S48^	921–618	Schizophrenis (100%)	16.2	4	32.9%	DEP; DIS; EXF; IND; NEG; OSF; POS; PRC; PRS; PSB; VEM; VOF
Ganella 2018^S49^	29–14	First Episode Psychositic Disorder (100%)	1.4	1	51.7%	DEP; NEG; OSF; POS
Gaughran 2017^S50^	403–259	Psychotic disorder (100%)	Unclear	1; 1.3	25.9%	DEP; OSF
Godin 2019^S51^	770–325	Schizophrenia (100%)	10.7	1	61.4%	DEP; OSF; PRC
Gorna 2008^S52^	125–125	Schizophrenia (100%)	< 2	1; 5	21.3%	IND; NEG; OSF; POS; PRC; PSB; QOL; VOF
Gorwood 2019^S53^	303–228	Schizophrenia (100%)	7.6	0.5; 1	26.1%	NEG; OSF; POS
Granholm 2020^S54^	107–101	Schizophrenia (80.7%); Schizoaffective disorder (19.3%)	Unclear	0.3; 0.5; 1	40.4%	IND; NEG; OVC; POS; PSB
Grawe 2006^S55^	50–50	Schizophrenia (80.0%); Schizoaffective disorder (12.0%); Schizophreniform disorder (8.0%)	< 2	2	14.0%	NEG; OSF; POS
Gumley 2022^S56^	73–62	Schizophrenia spectrum disorder (100%)	Unclear	0.5; 1	17.8%	DEP; DIS; NEG; OSF; POS; PRC
Harrow 2005^S57‐S63^	239–239	Schizophrenia (40.8%); Schizophreniform disorder (7.6%); Other psychotic disorder (51.6%)	< 2	2; 4.5; 7.5; 10; 15	8.3%	NEG;OSF; POS; PSB; VOF
Harvey 2010^S64^	61–61	Schizophrenia (100%)	33.3	3.8	45.1%	OSF; OVC; PSB
Hayhurst 2014^S65^	363–301	Schizophrenia, schizoaffective, schizophreniform or delusional disorder	11.6	1	18.1%	DEP; NEG; POS; QOL
Heeramun‐Aubeeluck 2015^S66^	38–38	Schizophrenia (100%)	Unclear	0.5; 1	62.4%	PRS; PSB; VEM
Heering 2015^S67^	1022–602	Schizophrenia, schizofreniform disorder or schizoaffective disorder	4.4	3.3	42.1%	OSF; OVC; QOL
Hoff 2005^S68^	21–21	Schizophrenia (74.3%); Schizoaffective disorder (5.7%)	1.5	10	58.0%	EXF; NEG; OVC; POS; PRS; VEM
Horan 2012^S69^	55–55	Schizophrenia (56.8%); schizoaffective disorder (12.4%); schizophreniform disorder (30.9%)	0.7	1	32.1%	IND; NEG; POS; PSB; VOF
Hui 2023^S70^	360–360	Schizophrenia spectrum disorder (100%)	0.0	1; 2; 3	Unclear	DEP; IND; NEG; OSF; POS; PSB; VOF
Ito 2015 ^S71^	155–111	Schizophrenia spectrum disorder (100%)	2.0	0.5; 1; 1.5	53.9%	NEG; OSF; POS; QOL
Jørgensen 2015 ^S72^	101–94	Schizophrenia (92.1%); Schizoaffective disorder (7.9%)	9.8	0.3; 0.5; 1	7.9%	NEG; POS; PRC
Kane 2016 ^S73,S74^	404–404	Schizophrenia (53.0%); Schizoaffective disorder, bipolar (5.9%); Schizoaffective disorder, depressive (14.1%); schizophreniform disorder (16.6%); Brief psychotic disorder (0.5%); Psychotic disorder NOS (9.9%)	3.7	0.5; 1; 1.5; 2	43.8%	DEP; DIS; NEG; POS; PRC; QOL; VOF
Kelly 2009 ^S75^	43–43	Schizophrenia (100%)	22.1	1	23.2%	OSF; POS; QOL
Kim 2019 ^S76^	87–87	Schizophrenia (100%)	8.8	0.5; 1	29.9%	POS; PRC
Klærke 2019 ^S77^	70–70	Schizophrenia (95.7%); Schizoaffective disorder (4.3%)	2.1	9.6	51.1%	NEG; OSF; POS
Koshiyama 2017 ^S78^	14–14	First Episode Psychosis (100%)	0.7	1.9	Unclear	NEG; OSF; POS
Lasser 2005 ^S79^	582–582	Schizophrenia (83.4%); schizoaffective disorder (16.6%)	Unclear	1	20.3%	DIS; NEG; POS; PRC
Lee 2023^S80^	54–54	Schizophrenia or schizoaffective disorder	12.7	14.1	40.7%	EXF; NEG; OVC; POS; PRS; QOL; VEM
Li 2017 ^S81,S82^	63–63	Schizophrenia or schizophreniform disorder	3.7	0.5; 1; 1.5; 2	43.8%	DIS; NEG; OSF; POS
Lindgren 2020 ^S83^	52–32	Schizophrenia (50.0%); schizophreniform disorder (21.2%); psychotic disorder NOS (23.1%); Brief psychotic disorder (5.8%)	0.0	1	38.5%	DEP; EXF; OSF; POS; PRS; VEM
Litman 2023 ^S84^	215–175	Schizophrenia (100%)	10.9	0.2; 0.5; 1	10.7%	DEP; IND; OSF; PRC; PSB; QOL
Liu 2023^S85^	96–76	Schizophrenia/schizophreniform disorder (78.1%); Other schizophrenia spectrum disorder (21.9%)	11.3	2	20.8%	DEP; OSF; PRC
Lopez‐Morinigo 2023^S86^	34–28	Schizophrenia spectrum disorder (100%)	> 5	0.2; 1	62.8%	DEP; DIS; NEG; OSF; POS; QOL
McGurk 2003^S87^	30–27	Schizophrenia (53.3%); schizaffective disorder (46.7%)	15.7	2	10.0%	EXF; NEG; POS; PRS; VEM
McNeely 2023 ^S88^	51–40	Schizophrenia and related psychotic disorders (100%)	21.3	1.1	21.6%	OSF; PRC; QOL
Meade 2020 ^S89^	412–238	Schizophrenia (100%)	11.3	0.5; 1	56.8%	DIS; IND; NEG; OSF; POS; PSB
Meagher 2004 ^S90^	82–82	Schizophrenia (100%)	44.7	2.9	36.4%	EXF; NEG; OVC; POS
Melle 2008^S91,S92^	281–186	Schizophrenia spectrum disorder (72.1%)	< 2	0.3; 1; 2; 10	33.2%	IND; NEG; OSF; POS; PSB
Moncrieff 2023 ^S93^	253–175	Schizophrenia (68.8%); Other psychotic disorders (31.2%)	> 5	0.5; 1; 2	24.9%	EXF; NEG; OSF; POS; PRC; PRS; QOL; VEM; VOF
Morrison 2018 ^S94^	75–65	Schizophrenia, schizoaffective disorder, or delusional disorder	< 2	0.5; 1	20.0%	DEP; NEG; OSF; POS; PRC; QOL
Na 2016 ^S95^	25–25	Schizophrenia (60.0%); Schizoaffective disorder (12.0%); Psychotic disorder NOS (28.0%)	Unclear	0.5; 1	4.0%	NEG; OSF; POS; PRC; PSB; VOF
Najarian 2023 ^S96^	178–178	Schizophrenia (100%)	12.3	0.5; 1; 2	13.5%	DIS; NEG; OSF; POS
Nakamura 2019 ^S97^	37–37	Schizophrenia (100%)	34.7	1.03	14.0%	NEG; OSF; POS
Neill 2022 ^S98^	85–43	Schizophrenia (91.9%); Schizoaffective disorder (8.1%)	15.7	0.2; 0.5; 1	49.4%	DEP; EXF; NEG; OVC; POS; PRS; QOL; VEM
Nordentoft 2006 ^S99‐S103^	255–255	Schizophrenia spectrum disorder (100%)	9.8	1; 2; 3.5	28.9%	DIS; IND; NEG; OSF; POS; PSB; VOF
Oh 2017 ^S104^	22–22	Psychotic disorder (100%)	< 2	1	45.0%	NEG; OSF; POS
Okin 1995 ^S105^	53–53	Schizophrenia (100%)	11.5	7.5	0.0%	IND; OVC; PSB; VOF
Oribe 2015 ^S106,S107^	18–18	Schizophrenia (100%)	1.2	1	0.0%	NEG; OSF; POS
Ortega 2021 ^S108^	61–61	First episode Psychosis (100%)	< 5	1	Unclear	NEG; OSF; POS; PRC
Ozawa 2019 ^S109^	35–35	Schizophrenia (100%)	35.7	1	25.5%	DEP; NEG; OSF; POS
Prouteau 2005 ^S110^	55–55	Schizophrenia (70.9%); Schizoaffective disorder (23.6%); Schizophreniform disorder (3.6%); Unspecified psychotic disorder (1.8%)	7.4	0.5; 1; 1.3	0.0%	IND; OSF; PRC; PSB
Putnam 2000 ^S111‐S117^	317–317	Schizophrenia (100%)	41.2	1; 1.2; 2.1; 4; 6	31.2%	IND; OSF; OVC; POS; VEM
Rodríguéz‐Sánchez 2008 ^S118‐S121^	549–549	Schizophrenia (60.0%); schizophreniform disorder (46.7%); psychosis NOS (3.3%); Brief psychotic disorder (6%)	2.3	0.1; 1; 3	27.5%	DEP; DIS; EXF; NEG; OSF; OVC; POS; PRS; VEM
Rossi 2009 ^S122^	326–326	Schizophrenia (74.9%); schizoaffective disorder (25.1%)	17.3	1	30.0%	NEG; OSF; POS
Rowland 2018 ^S123^	290–257	Schizophrenia (84.5%); Delusional disorder (15.5%)	0.0	1	17.9%	DEP; NEG; OSF; POS; PRC
Rund 2007 ^S124,S125^	300–280	Schizophrenia (52.3%); schizophreniform disorder (4.5%); schizoaffective disorder (10.8%); Delusional disorder (5.4%); psychosis NOS (27.0%)	0.2	0.3; 1	38.9%	DEP; EXF; NEG; OSF; POS; PRS; VEM
Ryu 2006 ^S126‐S129^	78–78	Schizophrenia (100%)	31.5	1; 2; 3; 4; 5; 6; 12; 15	28.2%	EXF; IND; NEG; OSF; OVC; POS; PRC; PRS; PSB; QOL; VEM
Salyers 2014 ^S130,S131^	118–118	Schizophrenia (46.6%); schizoaffective disorder (55.2%)	Unclear	0.8; 1.5	40.7%	NEG; POS; PRC; QOL
Schmidt 2017 ^S132^	120–120	Schizophrenia (55.0%); Schizoaffective disorder (19.2%); Schizophreniform disorder (14.2%); Delusional disorder (5.8%); Psychotic disorder NOS (5.8%)	< 5	1	15.8%	DEP; NEG; OSF; PRC; QOL
Scottish Schizophrenia Research group 1988 ^S133‐S135^	111–111	Schizophrenia (100%)	0.2	1; 2; 5	16.3%	EXF; IND; NEG; OVC; PSB; VEM; VOF
She 2017 ^S136^	170–169	Schizophrenia (100%)	7.2	0.3; 0.5; 1	36.5%	IND; NEG; POS; PSB
Siegel 2006 ^S137^	98–92	Schizophrenia (100%)	6.1	3	52.9%	DEP; NEG; OSF; POS
Sikira 2021 ^S138^	65–50	Schizophrenia spectrum disorder (100%)	Unclear	0.5; 1	23.1%	OSF; QOL
Smith 2002 ^S139^	46–45	Schizophrenia (60.9%); schizoaffective disorder (39.1%)	19.0	0.3; 0.5; 0.8; 1	37.5%	DIS; EXF; NEG; POS; PSB; VEM
Sommer 2021 ^S140^	113–70	Schizophrenia (100%)	1.3	0.3; 0.5; 0.8; 1; 2	58.8%	DEP; EXF; NEG; OSF; OVC; POS; VEM
Stouten 2014 ^S141,S142^	162–162	Schizophrenia (52.9%); Brief psychotic disorder (5.9%); Delusional disorder (5.2%); Shared psychotic disorder (1.3%); Psychotic disorder NOS (36.6%)	< 5	1	0.0%	IND; NEG; OSF; POS; PSB; VOF
Sweeney 1991 ^S143^	39–39	Schizophrenia (74.4%); schizophreniform disorder (10.3%); schizoaffective disorder (15.4%)	6.6	0.3; 1; 1.3; 1.5	0.0%	EXF; NEG; POS; PRS; VEM
Tabáres‐Seisdesos 2005 ^S144,S145^	47–47	Schizophrenia (100%)	8.7	1; 3	9.6%	DEP; EXF; IND; NEG; OSF; OVC; POS; PRS; PSB; VEM
Tabo 2017 ^S146^	120–120	Schizophrenia (100%)	16.3	1	Unclear	NEG; POS; QOL
Torgalsbøen 2015 ^S147,S148^	25–25	Schizophrenia (75.0%); schizoaffective disorder (21.4%); psychotic disorder NOS (3.6%)	< 0.5	2	10.7%	EXF; OVC; POS; VEM
Üçok 2011 ^S149‐S151^	115–105	Schizophrenia (100%)	1.2	0.3; 1; 2; 3; 4	25.6%	NEG; OSF; POS; VOF
Usui 2022 ^S152^	59–59	Schizophrenia (74.1%); Schizophreniform disorder (11.1%); Delusional disorder (3.7%); Psychotic disorder NOS (11.1%)	< 5	2	54.2%	DIS; NEG; POS; PRC
Veerman 2016 ^S153^	25–25	Schizophrenia (100%)	19.6	1	19.4%	DEP; EXF; NEG; POS; QOL; VEM
Veijola 2014 ^S154^	33–33	Schizophrenia (100%)	11.1	9	45.9%	OSF; OVC
Whitehorn 2002 ^S155^	103–56	Schizophrenia spectrum disorder (100%)	< 2	0.5; 1	52.4%	DIS; NEG; OSF; POS
Wilson‐d'Almeida 2013 ^S156^	306–306	Schizophrenia (100%)	Unclear	0.5; 1	12.3%	NEG; POS; PRC
Wittorf 2004 ^S157^	11–11	Schizophrenia (93.3%); schizoaffective disorder (6.7%)	6.1	1.1	60.5%	DIS; EXF; NEG; POS
Wittorf 2008 ^S158^	96–96	Schizophrenia (88.5%); schizoaffective disorder (11.5%)	6.1	1	36.0%	OSF; VEM
Wojtalik 2022 ^S159^	58–44	Schizophrenia (80.4%); Other psychotic disorder (19.6%)	3.7	0.8; 1.5	52.0%	DEP; EXF; IND; NEG; OSF; OVC; POS; PRS; PSB; VEM; VOF
Wunderink 2009 ^S160^	125–125	Schizophrenia (45.6%); other nonaffective psychosis (54.4%)	0.7	0.5; 1.3; 2	14.4%	NEG; POS; PSB; QOL
Xie 2005 ^S161,S162^	152–152	Schizophrenia (70.4%); schizoaffective disorder (29.6%)	12.0	0.5; 1; 1.5; 2; 2.5; 3; 4; 5; 6; 7; 8; 9; 10	23.1%	DIS; IND; NEG; POS; PRC; PSB; QOL; VOF
Xu 2014 ^S163,S164^	60–60	Schizophrenia (51.7%); schizophreniform disorder (20.0%); psychosis NOS (21.7%); schizoaffective disorder (6.7%)	0.0	1; 3	23.1%	DIS; EXF; NEG; OVC; POS
Zäske 2018 ^S165^	48–24	Schizophrenia (100%)	< 2	1	50.0%	DEP; OSF; PRC; PSB; QOL
Zhu 2022 ^S166^	270–181	Schizophrenia (100%)	23.0	1.5	33.0%	NEG; OSF; OVC; POS

Abbreviations: DEP = depressive symptoms; DIS = disorganization; EXF = executive functioning; IND = independence; NEG = negative symptoms; OSF = overall social functioning; OVC = overall cognition; POS = positive symptoms; PRC = personal recovery; PRS = processing speed; PSB = prosocial behavior; QOL = subjective quality of life; VEM = verbal memory; VOF = vocational functioning.

^a^ References of all included studies are presented in Supporting Information 11.

Effect sizes of change for each outcome domain are presented in Supporting Information 6. We found moderate improvement over time in overall social functioning (*d* = 0.62), positive symptoms (*d* = 0.69), and disorganization symptoms (*d* = 0.63). We found small improvement in prosocial behavior (*d* = 0.32), independence (*d* = 0.27), vocational functioning (*d* = 0.29), negative symptoms (*d* = 0.29), depression (*d* = 0.38), subjective quality of life (S‐QOL) (*d* = 0.34), and processing speed (*d* = 0.22). We found marginal improvement in personal recovery (*d* = 0.20), verbal memory (*d* = 0.16), executive functioning (*d* = 0.17), and overall cognition (*d* = 0.18). In all outcomes, we found significant heterogeneity. For all outcome domains of symptoms, social functioning, and for processing speed *I* ^2^ > 80%. For overall cognition, verbal memory, and executive functioning *I* ^2^ < 80%. We found a significantly larger improvement for patients with a short DOI compared to a long DOI in overall social functioning, positive symptoms, disorganization symptoms, and executive functioning. For negative symptoms, the largest improvement was found for patients with a DOI of 5–10 years.

### 4.3 Interrelationships of Change

**FIGURE 1:**
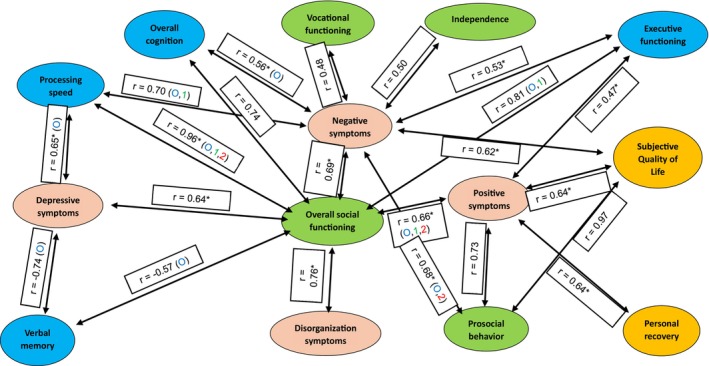
Correlations between changes in recovery domains within DOI subgroups. All correlation coefficients presented in this figure refer to the overall significant associations between recovery domains; O = overall associations; 1 = association in DOI < 5 years subgroup; 2 = association in DOI > 10 years subgroup; multiple references indicates that associations apply to multiple subgroups (e.g. O, 1 indicate both overall associations and association in DOI < 5 years subgroup between recovery domains) * = correlations remain significant after correction for outliers. Pink color = symptom subdomain; Green color = social functioning subdomain; Blue color = cognition subdomain; Orange color = personal recovery subdomain.

**FIGURE 2:**
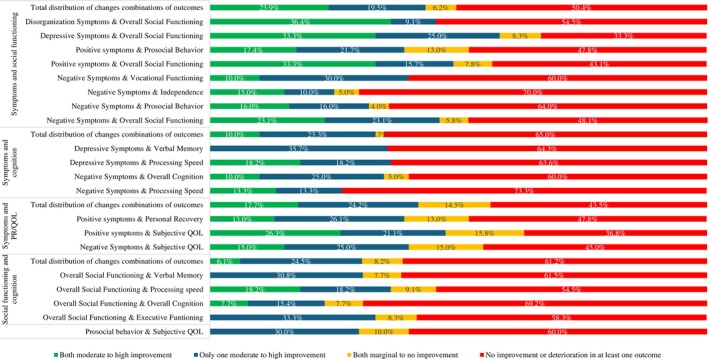
Distribution of changes in studies for combinations of outcomes or recovery domains with significant associations.

In Figure 1 and in Supporting Information 7, we presented an overview of correlations between the changes in different outcome domains. In Figure 1, we also presented whether correlations remain significant in specific DOI subgroups. We did not find any significant correlations in the DOI 5–10 years subgroup, most probably because of the limited number of studies investigating patients in this DOI subgroup. In Figure 2, we presented the distribution of combinations of outcome domains based on the four subgroups described in Section 3.4.3.

*Symptoms and social functioning*: We found strong associations between changes in positive symptoms and both prosocial behavior (*r* = 0.73, specifically for DOI > 10 years) and overall social functioning (*r* = 0.66). Most studies showed improvement in both combinations of outcome domains. Changes in negative symptoms were strongly associated with changes in prosocial behavior (*r* = 0.68, specifically for DOI > 10 years) and overall social functioning (*r* = 0.69), and moderately associated with changes in independence (*r* = 0.50) and vocational functioning (*r* = 0.48). Most studies showed improvement in the combination of negative symptoms and overall social functioning, but no improvement or deterioration in the other combinations of outcome domains. Finally, changes in both depressive symptoms (*r* = 0.64) and disorganization symptoms (*r* = 0.76) were strongly associated with changes in overall social functioning, specifically for the subgroup with a DOI > 10 years. Most studies showed improvement in combinations of depressive symptoms and overall social functioning but no improvement or deterioration in disorganization and overall social functioning.

Overall, 72 studies investigated 226 combinations of changes in symptoms and social functioning. In 49.6% of these outcome combinations, both symptoms and social functioning improved over time. In the remaining 50.4%, at least one of both outcomes showed no improvement or even deterioration over time.

*Symptoms and cognition*: Changes in negative symptoms showed strong associations with changes in executive functioning (*r* = 0.53, specifically for DOI < 5 years), overall cognition (*r* = 0.56) and processing speed (*r* = 0.70, specifically for DOI < 5 years). Changes in depressive symptoms were strongly associated with changes in verbal memory (*r* = −0.74) and processing speed (*r* = 0.65). Finally, changes in positive symptoms were moderately associated with changes in executive functioning (*r* = 0.47).

Overall, 29 studies investigated 60 combinations of changes in symptoms and cognition. In 35.0% of these outcome combinations, both symptoms and cognition improved over time.

*Symptoms and personal recovery*: Changes in negative symptoms were strongly associated with changes in S‐QOL (*r* = 0.62, specifically for DOI < 5 years). Furthermore, changes in positive symptoms were strongly associated with changes in both personal recovery (*r* = 0.64) and S‐QOL (*r* = 0.64).

Overall, 34 studies investigated 62 combinations of changes in symptoms and personal recovery. In 56.5% of these outcome combinations, both symptoms and personal recovery improved over time.

*Social functioning and cognition*: Changes in overall social functioning were strongly associated with changes in overall cognition (*r* = 0.74), processing speed (*r* = 0.96), executive functioning (*r* = 0.81, specifically for DOI < 5 years) and verbal memory (*r* = −0.57).

Overall, 19 studies investigated 49 combinations of changes in social functioning and cognition. In 38.8% of these outcome combinations, both social functioning and cognition improved over time.

*Social functioning and personal recovery*: We found strong associations between changes in prosocial behavior and S‐QOL (*r* = 0.97, specifically for DOI < 5 years). Overall, we observed no improvement or deterioration in combinations of prosocial behavior and S‐QOL in 60.0% of studies.

### 4.4 Analysis of Moderating Effects

**FIGURE 3:**
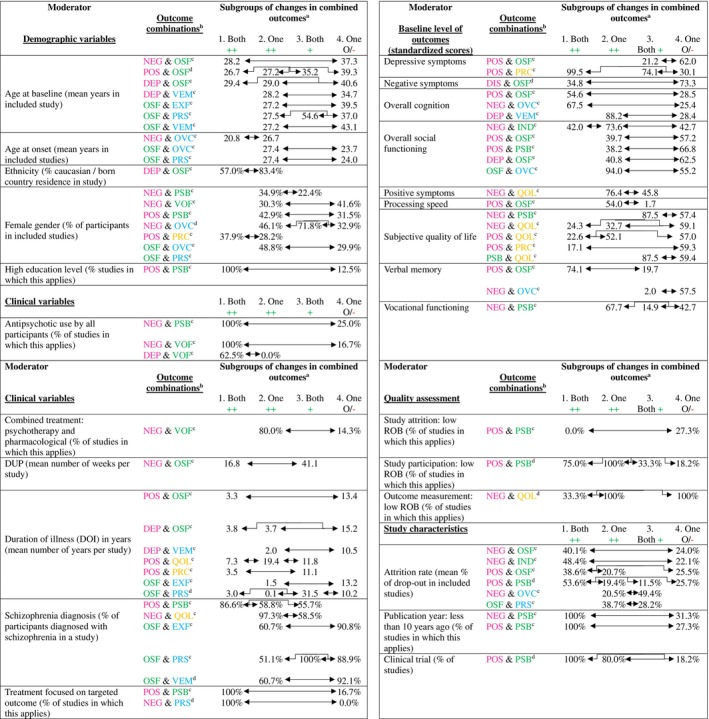
Overview of significant moderators for changes in outcome domains (mean differences or differences in prevalence). a. Arrows between subgroups indicate between which specific subgroups significant differences between moderators take place. 1. Both ++ = Studies reporting at least moderate improvement (i.e., *d* ≥ 0.5) in both outcome domains; 2. One ++ = Studies reporting at least moderate improvement (i.e., *d* ≥ 0.5) in one outcome domain, but low to marginal improvement (*d* < 0.5) in the other outcome domain; 3. Both + = Studies reporting low to moderate improvement (*d* < 0.5) in both outcome domains; 4. One O/− = Studies reporting no improvement or deterioration in at least one of the both outcome domains. b. DEP = depression; DIS = disorganization; EXF = Executive functioning; IND=Independence; NEG = Negative symptoms; OSF=Overall social functioning; OVC=Overall cognition; POS = positive symptoms; PRC=Personal recovery; PRS=Processing speed; PSB=Prosocial behavior; QOL = Subjective quality of life; VEM = Verbal memory; VOF=Vocational functioning. Pink color = symptom subdomain; Green color = social functioning subdomain; Blue color = cognition subdomain; Orange color = personal recovery subdomain. c. Significant moderator: *p* < 0.05. d. Significant moderator: *p* < 0.01.

A summary of moderating effects is presented in Figure 3. Full analysis results are presented in Supporting Information 8.

*Symptoms and social functioning*: Studies investigating patients with a younger age, a large percentage of patients using antipsychotics, a short baseline DOI, low baseline overall social functioning, more recently published studies, and a large attrition rate at follow‐up consistently showed better improvement in multiple combinations of social functioning and symptoms. We also found moderating effects of gender in multiple combinations of change in social functioning and symptoms, with better improvement in both positive and negative symptoms and prosocial behavior in studies with a larger proportion of females, but better improvement in negative symptoms and vocational functioning in studies with a lower proportion of females.

Furthermore, various moderators influenced one combination of change in symptoms and social functioning: clinical trials, studies investigating patients with a high education level, a short duration of untreated psychosis (DUP), a large percentage diagnosed with schizophrenia, a high baseline severity of negative symptoms, a high level of both overall cognition and processing speed, patients receiving combined and integrated treatment of both psychotherapy and pharmacological treatment, and a large percentage of patients receiving treatment that was focused on improving the targeted outcome domain.

*Symptoms and cognition*: Studies investigating patients with a high level of baseline overall cognition consistently showed better improvement in multiple combinations of change in symptoms and cognition. Furthermore, studies investigating patients with younger age at baseline, a younger age at onset, a shorter baseline DOI, a larger percentage of females, a larger percentage of patients receiving treatment that was focused on improving targeted outcome domains, and a large attrition rate at follow‐up are all associated with better improvement in one combination of change in symptoms and cognition.

*Symptoms and personal recovery*: Studies investigating patients with a short baseline DOI and a low level of baseline S‐QOL consistently showed better improvement in multiple combinations of change in symptoms and personal recovery. Furthermore, studies with a larger percentage of patients diagnosed with schizophrenia and a low baseline severity of depressive symptoms and positive symptoms are associated with better improvement in one combination of change in both domains.

*Social functioning and cognition*: Studies investigating patients with a younger age, an older age at onset, a larger percentage of females, and a lower percentage of patients diagnosed with schizophrenia consistently showed better improvement in multiple combinations of change in social functioning and cognition. Furthermore, studies investigating patients with a large baseline level of overall social functioning and a large attrition rate are associated with better improvement in one combination of change in both outcome domains.

*Social functioning and personal recovery*: Studies investigating patients with a high baseline level of S‐QOL are associated with more favorable changes in prosocial behavior and subjective quality of life. Of note, these results only apply to patients with a low level of improvement in both outcome domains and are based on a limited number of studies.

### 4.5 Outliers and Publication Bias

An overview of positive and negative outliers per outcome domain is presented in Supporting Information 10. Most correlations between outcome domains remain significant after outlier corrections (see Figure 1). After removal of positive outliers, we did not find any correlations between overall social functioning and verbal memory, overall cognition and executive functioning, between negative symptoms and both vocational functioning and independence, and between depressive symptoms and verbal memory. Finally, we did not find significant correlations between prosocial behavior and subjective quality of life after removal of both positive and negative outliers.

Funnel plots are presented in Supporting Information 11. We found indications of positive‐results bias for the outcome domains overall social functioning and positive symptoms. After corrections using the trim‐and‐fill method, we found a significantly smaller overall effect size (*d* = 0.29; *χ* ^2 =^ 18.6; *p* < 0.01) in the corrected outcomes of overall social functioning, but no differences in positive symptoms results. This suggests positive‐results bias of overall social functioning.

### 4.6 Quality Assessment

Quality assessment results of all included studies are presented in Supporting Information 9. Overall, most studies had a low risk of bias (ROB) in the domains of outcome assessment and statistical analysis and reporting, but a high ROB in study attrition and study confounding.

Analysis of moderating effects indicated consistent trends of associations between low ROB in study participation and more improvement in combinations of positive symptoms and prosocial behavior, and between low ROB in outcome measurement and less improvement in combinations of negative symptoms and S‐QOL (see Figure 3).

## 5 Discussion

In the current meta‐analysis, we investigated interrelationships of changes between social functioning, symptoms, cognition, and personal recovery in patients with SSD. We also investigated which moderators influenced these interrelationships. The current meta‐analysis partially builds on four previously published meta‐analyses that evaluated longitudinal changes in social functioning, symptoms, personal recovery, or quality of life and cognition separately [7, 8, 9, 10]. We found the strongest and most consistent positive associations between changes in symptoms and social functioning. Furthermore, we found consistent positive associations between changes in symptoms and cognition, between social functioning and cognition, and between symptoms and personal recovery. The strongest improvement over time was found for combinations of symptoms and social functioning, as well as for symptoms and personal recovery. In contrast, combinations of symptoms and cognition and combinations of social functioning and cognition showed relatively less improvement over time. A reflection of the current findings is discussed below.

### 5.1 Interrelationships of Change in Outcome Domains

Our findings indicated that changes in negative symptoms were associated with all domains of social functioning. This may indicate that a focus on improvement in negative symptoms might also facilitate improvement in social functioning, and vice versa. Previous research also indicated that negative symptom severity moderates improvement in social functioning [32, 33]. Of note, associations between changes in symptoms and social functioning were mostly observed in studies that investigated patients with a long illness duration (DOI). This suggests that social functioning and symptoms concurrently improve mainly later in the course of illness. This is remarkable as previous meta‐analyses [7, 8] indicated larger improvement in both symptoms and social functioning in patients with a short DOI. On the other hand, only a minority of patients with early psychosis achieved both symptomatic and functional remission [34]. Taken together, these findings might suggest that concurrent improvement of symptoms and social functioning is related to an adaptation process to the illness that takes place after attenuation of symptoms and disability earlier in the course of illness.

Changes in depressive or negative symptoms were associated with changes in multiple domains of cognition. These findings might be explained by possible shared underlying mechanisms, such as impairment in goal‐directed behavior in negative symptoms, depressive symptoms, and cognition [35].

Furthermore, changes in overall social functioning were associated with changes in all domains of cognition. This is in line with previous research [36]. However, only the minority of studies in our meta‐analysis showed improvement in both cognition and social functioning, despite favorable effects of cognitive skills training combined with psychosocial rehabilitation interventions on vocational functioning and social skills, as indicated in previous research [37, 38]. These findings suggest that improving cognition might enhance improvement in social functioning, but that concurrent improvement in both outcomes is still challenging and important to address in future research.

Finally, changes in personal recovery are consistently associated with changes in positive symptoms. Previous studies found the strongest associations between depressive symptoms and personal recovery [6, 13, 39], but weaker associations with positive symptoms. Our findings might suggest that improvement in positive symptoms enables better adaptation to illness, due to a lower level of disability. This hypothesis was supported in previous research [39]. Changes in personal recovery were not consistently associated with changes in other outcome domains. This might be partly explained by previous findings indicating that self‐reported assessments of personal recovery were not related to clinician‐rated assessments of symptoms, social functioning, and cognition [14, 40]. Furthermore, while symptoms, social functioning, and cognition are directly influenced by SSD, personal recovery is less directly related to the illness but rather expresses more general human existential experiences or capacities.

Taken together, the consistent associations between changes in symptoms, social functioning, and cognition suggest that improvement in these three outcome domains often takes place together. Especially, improvement in negative symptoms, overall social functioning, and positive symptoms concurrently improve with multiple outcome domains and might be driving forces for improvement in other subdomains. However, in the majority of studies, we found marginal or no improvement in at least one of the recovery domains in the combinations that were investigated. This indicated that associations between recovery domains do not automatically translate into collaborative improvement in both recovery domains.

### 5.2 Moderating Effects on Interrelationships of Changes in Outcome Domains

Although moderating effects differed between outcomes, we generally found that studies investigating patients with a younger age, a shorter DOI, a larger percentage of females, a lower percentage of patients diagnosed with schizophrenia, patients receiving treatment focused on improvement of targeted outcomes, and a large attrition rate positively influenced changes in multiple combinations of outcome domains.

The positive influence of studies investigating patients with a younger age and a short DOI mainly applied for combinations of symptoms, social functioning, and cognition. This is in line with previous meta‐analyses [7, 8, 10]. Although patterns of development of psychosis differ, most functional deterioration and the largest increases in severity of symptoms occur during the early phase of psychosis, and therefore patients with a short DOI have the largest potential for improvement [34, 41, 42]. In contrast, studies investigating patients with a longer DOI by definition include patients with a more chronic and less favorable prognosis of improvement. This contrast might be the main explanation for the positive influence of short DOI and young age. We also found a positive influence of a short DOI on improvement in combinations of personal recovery and positive symptoms. Previous research, however, did not find larger improvement of personal recovery in early psychosis [3, 10]. Therefore, this finding is mostly explained by larger improvement of positive symptoms earlier in the course of illness.

Furthermore, studies with a larger percentage of females showed better improvement in combinations of both social functioning and symptoms with cognition. Previous research also indicated that females showed larger improvement in cognition [9], and a better prognosis of a combination of clinical, social, and personal recovery [3]. This implies that future research should focus on exploring tailored interventions to facilitate cognition and social adjustment for males with SSD.

We also found that studies with a larger percentage of patients diagnosed with schizophrenia are associated with less favorable improvement, mainly for the combination of social functioning and cognition. This is mainly explained by the clinical characteristics of a schizophrenia classification that is given to patients with a poor prognosis of recovery in clinical and functional domains [18].

Results also indicated that studies that delivered treatment focused on the targeted outcome domain to a subsample of the study population showed larger improvement in combinations of symptoms and both social functioning and cognition. This finding is substantiated by favorable results of targeted treatment in previous research [43, 44]. We also analyzed the influence of study design (i.e., clinical trials or cohort studies) but did not find consistent moderating effects of study design on the combinations of outcomes. It is important to note that these results do not imply any treatment effects but only indicate that larger improvement in combinations of outcomes was found in studies that provided treatment that targeted outcomes that were analyzed in this meta‐analysis. Since we analyzed longitudinal changes on a study level (including both the intervention and control condition), this means that in these analyses, participating in such a study was considered a moderator, not receiving the targeted intervention. We did not find any moderating treatment effects on changes in personal recovery. This is in line with previous meta‐analysis about changes in personal recovery [10]. More focus on recovery‐oriented practices, peer support, and support by professionals with lived experiences, and research that assess its effectiveness, might be needed to facilitate personal recovery, accompanied by other outcome domains. It also indicates that a favorable clinical and social context is of influence for improvement in multiple outcome domains.

Finally, studies with a large attrition rate showed larger improvement in combinations of social functioning and symptoms. This suggests that in studies with a large attrition bias, a selective group of patients with more improvement in social functioning and symptoms remains at follow‐up. This could have substantial influences on the outcomes [45]. Therefore, attrition bias might have contributed to an overestimation of improvement in combinations of symptoms and social functioning.

### 5.3 Limitations

Several limitations should be addressed while considering our findings. A first limitation is our narrow selection of studies. We set strict selection criteria for studies based on the classification of the study sample, follow‐up length, number of studies available, and variability of effect sizes of change in previous meta‐analyses. Herewith, we may have missed important studies that might have added knowledge. We also limited ourselves to quantitative results and clinical observations of symptoms, social functioning, and cognition. This limits the interpretation of our findings, as qualitative and self‐reported assessments also may offer an important perspective on these domains.

Due to our selection of studies, we also missed out on several longitudinal studies that reported on only one outcome domain or a combination of outcomes that were not included in this meta‐analysis. This might also have influenced our results. The positive‐results bias we found for overall social functioning might be explained by this narrow selection.

Furthermore, our set of moderators is relatively elaborate, despite the fact that we used strict selection criteria for the inclusion of a priori selected moderators. This might result in a higher chance of finding false positive results [24]. We tried to limit this risk by executing Benjamini‐Hochberg corrections on our significant outcomes to control for multiple testing effects [30]. Furthermore, we tried to specifically reflect on the moderators that are influential in multiple combinations of outcome domains. These moderators were consistently found in multiple combinations of outcomes and are therefore more reliable indicators of influence for our findings.

Another limitation is that we exclusively included studies investigating diagnosed patients that are known in mental health care settings. As a consequence, we miss out on patients who might receive support outside of mental healthcare, as well as patients who already finalized treatment and patients who are not capable of participating in research. Therefore, we were not able to investigate the influence of potentially important contextual or social factors outside of mental healthcare, which were not sufficiently addressed in our included studies. We do think these factors are also of influence and an important topic for future research.

Finally, we included studies conducted in different cultural, clinical, and social contexts, and these studies used a wide variety of assessment instruments. This inevitably leads to heterogeneity [46]. We tried to explore this heterogeneity through an analysis of moderating effects and reported which moderators are consistently of influence for our outcomes. In interpreting the mostly substantial correlations between outcome domains we observed, one should realize that mutual deterioration or stability of outcome domains also contributes to observed correlations.

## 6 Conclusions

In this meta‐analysis, we found that improvement in symptoms, social functioning, and cognition are associated with each other. This suggests that improvement in these outcome domains often goes together and may even boost each other. Especially, negative symptoms, overall social functioning, and positive symptoms concurrently improve with multiple outcome domains and might be driving forces for improvement. Changes in personal recovery are less strongly related to changes in other outcome domains than changes in other outcome domains. More substantial improvements in combinations of outcome domains were observed in patients with a young age, short DOI, females, a lower percentage of patients diagnosed with schizophrenia, and in specialized treatment. This suggests that focusing on early psychosis might amplify concurrent improvement in multiple outcome domains. It also suggests that more effort is needed to facilitate and sustain improvement for patients in later phases of SSD. Special attention in research and clinical care is needed for male patients diagnosed with schizophrenia who showed the least improvement in combined outcome domains. Overall, this meta‐analysis suggests that an integrated approach targeting multiple outcome domains jointly boosts long‐term improvement, but special attention for personal recovery is needed.

## Disclosure

The authors have nothing to report.

## Conflicts of Interest

The authors declare no conflicts of interest.

### Peer Review

The peer review history for this article is available at https://www.webofscience.com/api/gateway/wos/peer‐review/10.1111/acps.13808.

## Supporting information


Data S1.

## Data Availability

The data that supports the findings of this study are available in the Supporting Information of this article. For any additional questions about data availability we suggest to contact the corresponding author.
