# Supplementary material for: Interrelationships of Changes in Outcome Domains in Patients With Schizophrenia Spectrum Disorders: A Meta‐Analysis
Source: Acta Psychiatr Scand. 2025 Mar 30;152(1):6–26. doi: 10.1111/acps.13808 (PMC12127061; doi:10.1111/acps.13808)
Supplement: Supplementary file 1 — Data S1. [file ACPS-152-6-s001.docx]

# **Supplementary Materials 1.** PRISMA Checklist

| **Section and Topic** | **Item #** | **Checklist item** | **Location where item is reported** |
| --- | --- | --- | --- |
| **TITLE** | | |  |
| Title | 1 | Identify the report as a systematic review. | Title page (p1) |
| **ABSTRACT** | | |  |
| Abstract | 2 | See the PRISMA 2020 for Abstracts checklist. | Abstract (p.3) |
| **INTRODUCTION** | | |  |
| Rationale | 3 | Describe the rationale for the review in the context of existing knowledge. | Introduction (p.4) |
| Objectives | 4 | Provide an explicit statement of the objective(s) or question(s) the review addresses. | Introduction (p.4) |
| **METHODS** | | |  |
| Eligibility criteria | 5 | Specify the inclusion and exclusion criteria for the review and how studies were grouped for the syntheses. | 2.2 Eligibility criteria (p.5) |
| Information sources | 6 | Specify all databases, registers, websites, organisations, reference lists and other sources searched or consulted to identify studies. Specify the date when each source was last searched or consulted. | 2.1 Data sources (p.5) |
| Search strategy | 7 | Present the full search strategies for all databases, registers and websites, including any filters and limits used. | Supplementary Materials 2 |
| Selection process | 8 | Specify the methods used to decide whether a study met the inclusion criteria of the review, including how many reviewers screened each record and each report retrieved, whether they worked independently, and if applicable, details of automation tools used in the process. | 2.2 Eligibility criteria (p.5) |
| Data collection process | 9 | Specify the methods used to collect data from reports, including how many reviewers collected data from each report, whether they worked independently, any processes for obtaining or confirming data from study investigators, and if applicable, details of automation tools used in the process. | 2.4 Data-extraction and synthesis (p. 6-7) |
| Data items | 10a | List and define all outcomes for which data were sought. Specify whether all results that were compatible with each outcome domain in each study were sought (e.g. for all measures, time points, analyses), and if not, the methods used to decide which results to collect. | 2.3 Outcome domains (p.5-6) and Supplementary Materials 3 |
|  | 10b | List and define all other variables for which data were sought (e.g. participant and intervention characteristics, funding sources). Describe any assumptions made about any missing or unclear information. | 2.4 Data-extraction and synthesis (p. 6-7) |
| Study risk of bias assessment | 11 | Specify the methods used to assess risk of bias in the included studies, including details of the tool(s) used, how many reviewers assessed each study and whether they worked independently, and if applicable, details of automation tools used in the process. | 2.5 Quality assessment (p.7) |
| Effect measures | 12 | Specify for each outcome the effect measure(s) (e.g. risk ratio, mean difference) used in the synthesis or presentation of results. | 2.6.1 Meta-analytic procedure (p.7-8) |
| Synthesis methods | 13a | Describe the processes used to decide which studies were eligible for each synthesis (e.g. tabulating the study intervention characteristics and comparing against the planned groups for each synthesis (item #5)). | 2.4 Data-extraction and synthesis (p. 6-7) |
|  | 13b | Describe any methods required to prepare the data for presentation or synthesis, such as handling of missing summary statistics, or data conversions. | 2.4 Data-extraction and synthesis (p. 6-7) and 2.6 Statistical analysis (p.7-8) |
|  | 13c | Describe any methods used to tabulate or visually display results of individual studies and syntheses. | 2.6 Statistical analysis (p.7-8) |
|  | 13d | Describe any methods used to synthesize results and provide a rationale for the choice(s). If meta-analysis was performed, describe the model(s), method(s) to identify the presence and extent of statistical heterogeneity, and software package(s) used. | 2.6 Statistical analysis (p.7-8) |
|  | 13e | Describe any methods used to explore possible causes of heterogeneity among study results (e.g. subgroup analysis, meta-regression). | 2.6 Statistical analysis (p.7-8) |
|  | 13f | Describe any sensitivity analyses conducted to assess robustness of the synthesized results. | 2.6 Statistical analysis (p.7-8) |
| Reporting bias assessment | 14 | Describe any methods used to assess risk of bias due to missing results in a synthesis (arising from reporting biases). | 2.5 Quality assessment (p.7) and 2.6.4. Handling outliers and publication bias |
| Certainty assessment | 15 | Describe any methods used to assess certainty (or confidence) in the body of evidence for an outcome. | 2.6 Statistical analysis (p.7-8) |
| **RESULTS** | | |  |
| Study selection | 16a | Describe the results of the search and selection process, from the number of records identified in the search to the number of studies included in the review, ideally using a flow diagram. | 3.1. Study Flow (p.8) and Supplementary Materials 4 |
|  | 16b | Cite studies that might appear to meet the inclusion criteria, but which were excluded, and explain why they were excluded. | 4.3 Limitations (p. 17-18) |
| Study characteristics | 17 | Cite each included study and present its characteristics. | 3.2. Study Characteristics (p.9), Table 2 and Supplementary Materials 11 |
| Risk of bias in studies | 18 | Present assessments of risk of bias for each included study. | 3.6. Quality assessment (p.13) and Supplementary Materials 8 |
| Results of individual studies | 19 | For all outcomes, present, for each study: (a) summary statistics for each group (where appropriate) and (b) an effect estimate and its precision (e.g. confidence/credible interval), ideally using structured tables or plots. | 3.2. Study Characteristics (p.9), Figure 1 and Supplementary Materials 5 |
| Results of syntheses | 20a | For each synthesis, briefly summarise the characteristics and risk of bias among contributing studies. | 3.3 Interrelationships of change (p.9-11), 3.6 Quality assessment (p.13) and Supplementary Materials 8 |
|  | 20b | Present results of all statistical syntheses conducted. If meta-analysis was done, present for each the summary estimate and its precision (e.g. confidence/credible interval) and measures of statistical heterogeneity. If comparing groups, describe the direction of the effect. | 3.3 Interrelationships of change (p.9-11), 3.4 analysis of moderating effects (p.11-13) and Figure 1-3 |
|  | 20c | Present results of all investigations of possible causes of heterogeneity among study results. | 3.4 analysis of moderating effects (p.11-13) |
|  | 20d | Present results of all sensitivity analyses conducted to assess the robustness of the synthesized results. | 3.4 analysis of moderating effects (p.11-13) |
| Reporting biases | 21 | Present assessments of risk of bias due to missing results (arising from reporting biases) for each synthesis assessed. | 3.5 Outliers and publication bias and Supplementary Material 10 |
| Certainty of evidence | 22 | Present assessments of certainty (or confidence) in the body of evidence for each outcome assessed. | 3.3 Interrelationships of change (p.9-11), 3.4 analysis of moderating effects (p.11-13) |
| **DISCUSSION** | | |  |
| Discussion | 23a | Provide a general interpretation of the results in the context of other evidence. | 4.1 Interrelationships of change in outcome domains (p.14-15 and 4.2. Moderating effects on interrelationships of changes in outcome domains (p.15-17) |
|  | 23b | Discuss any limitations of the evidence included in the review. | 4.3 Limitations (p.17-18) |
|  | 23c | Discuss any limitations of the review processes used. | 4.3 Limitations (p.17-18) |
|  | 23d | Discuss implications of the results for practice, policy, and future research. | 4.1 Interrelationships of change in outcome domains (p.14-15, 4.2. Moderating effects on interrelationships of changes in outcome domains (p.15-17) and 4.4 Conclusion (p.18) |
| **OTHER INFORMATION** | | |  |
| Registration and protocol | 24a | Provide registration information for the review, including register name and registration number, or state that the review was not registered. | Methods (p.5) |
|  | 24b | Indicate where the review protocol can be accessed, or state that a protocol was not prepared. | Methods (p.5) |
|  | 24c | Describe and explain any amendments to information provided at registration or in the protocol. | Methods (p.5) |
| Support | 25 | Describe sources of financial or non-financial support for the review, and the role of the funders or sponsors in the review. | Title page (p.1) |
| Competing interests | 26 | Declare any competing interests of review authors. | Title page (p.1) |
| Availability of data, code and other materials | 27 | Report which of the following are publicly available and where they can be found: template data collection forms; data extracted from included studies; data used for all analyses; analytic code; any other materials used in the review. | Supplementary Materials |

**Supplementary material 2.** Search history

**PsycInfo**

| **#** | **Query / limiters** |
| --- | --- |
| 1 | (Schizophrenia or Disorganized or Paranoid or Acute Schizophreniform disorder or Psychosis Schizoaffective disorder or Schizophrenia spectrum disorder Psychotic disorder).af. |
| 2 | (Delusion or Thought disturbances or Paranoia or Hallucinations or Visual or Auditory).af. |
| 3 | (Course or Prognosis or Disease or Evaluation or Rehabilitation or Remission or Recovery or Changes or Improvement or Deterioration or Development or Enhancement or Decrease or Decay or Depravation).af. |
| 4 | (Functioning or Social or Vocational or Work or Education or Relationships or Functional or Society or Symptom or Symptoms or Positive or Negative or Disorganized or Disorganization or Depression or Mood or Psychotic or Quality of life or QOL or Subjective or Well-being or Self-esteeem or Stigma or Personal or Recovery or Personal recovery or Cognition or Intelligence or IQ or Memory or Working or Long-term or Executive or Language or Motor or Perception or Processing speed or Recognition or Visuospatial).af. |
| 5 | 1 and 2 |
| 6 | 3 and 4 and 5 |
| 7 | limit 6 to (english language and abstracts and (2100 general psychology or 2224 clinical psychological testing or 2225 neuropsychological assessment or 2820 cognitive & perceptual development or 2840 psychosocial & personality development or 3000 social psychology or 3040 social perception & cognition or 3210 psychological disorders or 3213 schizophrenia & psychotic states or 3300 health & mental health treatment & prevention or 3310 psychotherapy & psychotherapeutic counseling or 3380 rehabilitation or 3384 occupational & vocational rehabilitation) and adulthood <18+ years> and ("300 adulthood <age 18 yrs and older>" or 320 young adulthood <age 18 to 29 yrs> or 340 thirties <age 30 to 39 yrs> or 360 middle age <age 40 to 64 yrs> or "380 aged <age 65 yrs and older>") and ("0100 journal" or "0110 peer-reviewed journal") and journal article and human") |
| **Results** | **5267** |

**Pubmed**

| Search number | Query | Results |
| --- | --- | --- |
| 1 | (((((((schizophrenia[MeSH Terms]) OR (disorganized schizophrenia[MeSH Terms])) OR (catatonic schizophrenia[MeSH Terms])) OR (disorders, schizophreniform[MeSH Terms])) OR (disorders, schizophrenic[MeSH Terms])) OR (disorders, schizoaffective[MeSH Terms])) OR (psychosis[MeSH Terms])) OR (disorder, psychotic[MeSH Terms]) | 152,257 |
| 2 | ((((delusion[MeSH Terms]) OR (thought disturbance[MeSH Terms])) OR (behavior, paranoid[MeSH Terms])) OR (auditory hallucination[MeSH Terms])) OR (visual hallucinations[MeSH Terms]) | 12,750 |
| 3 | (((((((((((((((course, short term[MeSH Terms]) OR (course[MeSH Terms])) OR (prognosis[MeSH Terms])) OR (evaluation[MeSH Terms])) OR (care, self rehabilitation[MeSH Terms])) OR (rehabilitation[MeSH Terms])) OR (remission[MeSH Terms])) OR (recovery[MeSH Terms])) OR (changes[MeSH Terms])) OR (improvement[MeSH Terms])) OR (deterioration[MeSH Terms])) OR (development[MeSH Terms])) OR (enhancement[MeSH Terms])) OR (decrease[MeSH Terms])) OR (decay[MeSH Terms])) OR (depravation[MeSH Terms]) | 550,904 |
| 4 | ((((((((((((((((((((((((((((((((((((((functioning[MeSH Terms]) OR (social[MeSH Terms])) OR (vocational[MeSH Terms])) OR (work[MeSH Terms])) OR (education[MeSH Terms])) OR (relationship[MeSH Terms])) OR (functional[MeSH Terms])) OR (society[MeSH Terms])) OR (friends society[MeSH Terms])) OR (symptoms[MeSH Terms])) OR (affective symptoms[MeSH Terms])) OR (positive symptoms[MeSH Terms])) OR (negative symptoms[MeSH Terms])) OR (disorganization[MeSH Terms])) OR (depression[MeSH Terms])) OR (disorder, mood[MeSH Terms])) OR (psychotic[MeSH Terms])) OR (quality of life[MeSH Terms])) OR (qol[MeSH Terms])) OR (subjective[MeSH Terms])) OR (wellbeing[MeSH Terms])) OR (self-esteem[MeSH Terms])) OR (social stigma[MeSH Terms])) OR (internalized stigma[MeSH Terms])) OR (self-stigma[MeSH Terms])) OR (personal recovery[MeSH Terms])) OR (cognition[MeSH Terms])) OR (intelligence[MeSH Terms])) OR (IQ[MeSH Terms])) OR (memory[MeSH Terms])) OR (working memory[MeSH Terms])) OR (long-term memory[MeSH Terms])) OR (executive functions[MeSH Terms])) OR (language[MeSH Terms])) OR (activity, motor[MeSH Terms])) OR (perception[MeSH Terms])) OR (processing speed[MeSH Terms])) OR (recognition[MeSH Terms])) OR (visuospatial[MeSH Terms]) | 1,655,594 |
| 5 | #1 AND #2 AND #3 AND #4 | 2862 |

**Cochrane**

| **ID** | **Search** | **Hits** |
| --- | --- | --- |
| #1 | MeSH descriptor: [Schizophrenia] explode all trees | 7876 |
| #2 | MeSH descriptor: [Schizophrenia Spectrum and Other Psychotic Disorders] explode all trees | 9619 |
| #3 | MeSH descriptor: [Psychotic Disorders] explode all trees | 3174 |
| #4 | MeSH descriptor: [Delusions] explode all trees | 175 |
| #5 | MeSH descriptor: [Hallucinations] explode all trees | 365 |
| #6 | #1 OR #2 OR #3 OR #4 OR #5 | 9795 |
| #7 | MeSH descriptor: [Disease Progression] explode all trees | 7781 |
| #8 | MeSH descriptor: [Mental Health Recovery] explode all trees | 8 |
| #9 | (course of illness):ti,ab,kw | 3268 |
| #10 | (prognosis of illness):ti,ab,kw | 1772 |
| #11 | (changes in illness):ti,ab,kw | 6552 |
| #12 | #7 OR #8 OR #9 OR #10 OR #11 | 18104 |
| #13 | (psychotic symptoms):ti,ab,kw | 3700 |
| #14 | (negative symptoms):ti,ab,kw | 13981 |
| #15 | (functioning):ti,ab,kw | 27292 |
| #16 | (social adjustment):ti,ab,kw | 2751 |
| #17 | (vocational functioning):ti,ab,kw | 293 |
| #18 | (personal recovery):ti,ab,kw | 895 |
| #19 | MeSH descriptor: [Quality of Life] this term only | 27155 |
| #20 | (self-stigma):ti,ab,kw | 167 |
| #21 | (empowerment):ti,ab,kw | 2464 |
| #22 | (self-esteem):ti,ab,kw | 3690 |
| #23 | (well-being):ti,ab,kw | 15275 |
| #24 | MeSH descriptor: [Mental Processes] explode all trees | 46642 |
| #25 | #13 OR #14 OR #15 OR #16 OR #17 OR #18 OR #19 OR #20 OR #21 OR #22 OR #23 OR #24 | 124937 |
| #26 | #6 AND #12 AND #25 | 1357 |

**CINAHL**

| **#** | **Query** | **Limiters/Expanders** | **Results** |
| --- | --- | --- | --- |
| S1 | TI schizophrenia OR TI disorganized OR TI paranoid OR TI acute OR TI schizophreniform disorder OR TI schizoaffective disorder OR TI psychosis OR TI psychotic disorder OR TI schizophrenia spectrum OR TI delusion OR TI hallucination OR TI thought disturbance | Limiters - Abstract Available; English Language; Peer Reviewed; Research Article; Human; Journal Subset: Peer Reviewed; Publication Type: Journal Article; Age Groups: Adult: 19-44 years, Middle Aged: 45-64 years | 49,264 |
| S2 | TI course OR TI prognosis OR TI evaluation OR TI rehabilitation OR TI remission OR TI recovery OR TI changes OR TI improvement OR TI enhancement OR TI development OR TI decrease OR TI deterioration | Limiters - Abstract Available; English Language; Peer Reviewed; Research Article; Journal Subset: Peer Reviewed; Publication Type: Journal Article; Age Groups: Adult: 19-44 years, Middle Aged: 45-64 years | 13,042 |
| S3 | TI quality of life OR TI qol OR TI subjective OR TI well-being OR TI self-esteem OR TI self-efficacy OR TI empowerment OR TI stigma OR TI self-stigma OR TI personal recovery OR TI recovery | Limiters - Abstract Available; English Language; Peer Reviewed; Research Article; Journal Subset: Peer Reviewed; Publication Type: Journal Article; Age Groups: Adult: 19-44 years, Middle Aged: 45-64 years | 2,236 |
| S4 | S1 OR S2 OR S3 |  | 11,490 |
| S5 | S1 AND S2 AND S4 |  | 1568 |

# **Supplementary material 3.** Overview definitions and assessment instruments of outcome domains

| **1. Overall social functioning** | | |
| --- | --- | --- |
| *Definition:* Outcomes measuring overall functioning in any social setting or role, based on clinician-rated functional outcome scales and standardized composite scores clustering different domains of societal functioning. | | |
| **Outcome name** | **Measurement** | **Explanation of outcome** |
| Autonomous daily living | Global Assessment of Functioning (GAF) | Number (%) of people who have a GAF score > 50 |
| FSRF | Functional Skills Rating Form (FSRF) | FSRF total score |
| Functional remission | 1. The Strauss Carpenter Level of Functioning Scale (SCS)  2. Functional remission composite rate | 1. Functional remission rate operationalized as a score > 3 on the SCS  2. Functional remission composite rate defined as: 1. occupational ⁄ vocational status: i.e. paid or unpaid full- or part-time employment,  being an active student in a university or head of household with employed partner; 2. independent living: i.e. living alone, with partner or with peers, and 3. social relationships: i.e. having ≥ 2 social contacts during the last 4 weeks or spouse |
| GAF | Global Assessment of Functioning (GAF) | GAF total score or number of participants with a GAF score ≥ 61 |
| GAF functioning | Global Assessment of Functioning (GAF) | GAF function score |
| General psychosocial functioning | Personal and Social Performance scale (PSP) | PSP general functioning scale score |
| Grip on life | Grip on life questionnaire | Grip on life assessment outcome |
| LFS overall level of functioning | Strauss-Carpenter Level of Function scale (LFS) | LFS overall level of functioning score |
| LKP score | Levenstein-Klein-Pollack (LKP) scale | LKP scale total score |
| LOF | Level of Functioning (LOF) scale | LOF scale total score |
| SAFE (total score) | Social and Adaptive Functioning Evaluation (SAFE) | SAFE Total Score |
| SCOS | Strauss-Carpenter Outcome Scale (SCOS) | SCOS total score |
| Social Functioning Scale (SFS) | Social Functioning Scale (SFS) | SFS total score |
| SOFAS | Social and Occupational Functioning Assessment Scale (SOFAS) | SOFAS total score |
| UPSA | University of California, San Diego Performance-Based Skills Assessment (UPSA) | UPSA total score |
| WHO-DAS (total score) | World Health Organization's Disability Assessment Scale (WHO-DAS) | WHO-DAS total score |
| **2. Prosocial behavior** | | |
| *Definition:* the level of social skills, relationships and social adaptive behavior | | |
| **Outcome name** | **Measurement** | **Explanation of outcome** |
| AIPSS processing | Assessment of Interpersonal Problem-Solving Skills (AIPSS) | AIPSS processing subscale |
| AIPSS receiving | Assessment of Interpersonal Problem-Solving Skills (AIPSS) | AIPSS receiving subscale |
| AIPSS sending | Assessment of Interpersonal Problem-Solving Skills (AIPSS) | AIPSS sending subscale |
| Emergency or crisis behaviour | Disability Assessment Schedule – II (DAS-II) Spanish version | Proportion of participants with an improvement in the DAS-II emergency or crisis behaviour score |
| Family relationships | Schizophrenia Care and Assessment Program Health Questionnaire (SCAP-HQ) | Family relationships subscale of the SCAP-HQ |
| FSRF communication | Functional Skills Rating Form (FSRF) | FSRF communication item score |
| FSRF social skills | Functional Skills Rating Form (FSRF) | FSRF social skills item score |
| GSDS | Groningen Social Disability Schedule (GSDS) | GSDS total score |
| Interpersonal functioning | Quality of Life Scale (QLS) Interpersonal functioning | QLS score on the interpersonal domain; clinician-rated |
| Personal and Social Performance Score (PSP) | Personal and Social Performance scale (PSPS) | PSPS total score |
| Personal and social relationships | Personal and Social Performance Scale (PSPS) | PSPS personal and social relationships |
| QOLI family contact | Quality of Life interview (QOLI) | QOLI family contact subscale |
| QOLI social contact | Quality of Life interview (QOLI) | QOLI social contact subscale |
| Percentage (%) arrested / jailed | Percentage arrested / jailed | Percentage of participants arrested / jailed |
| Prison stay past year | Jail/prison stay past year | Proportion of participants that are in jail or prison the past year |
| PSP DAB | Personal and Social Performance Scale (PSPS) | PSPS disturbing and aggressive behaviors score |
| REHAB general behaviour | Rehabilitation Evaluation Hall and Baker Scale (REHAB) | REHAB general behaviour subscale |
| REHAB social activity | Rehabilitation Evaluation Hall and Baker Scale (REHAB) | REHAB social activity subscale |
| Relationship impairment | 1. Psychosocial impairment score  2. Personal and Social Performance Scale (PSPS) | 1. Score of relationship impairment  2. PSPS problems in relationships subscale score |
| RFS social | Role functioning scale | RFS social subscale score |
| Sexual intercourse past month | Sexual intercourse past month | Number of participants with sexual intercourse the past month |
| SFQ frequency index | Social Functioning Questionnaire (SFQ) | SFQ frequency index score |
| SFQ global index | Social Functioning Questionnaire (SFQ) | SFQ global index score |
| SFQ satisfaction index | Social Functioning Questionnaire (SFQ) | SFQ satisfaction index score |
| SFS interpersonal | Social Functioning Scale (SFS) | SFS interpersonal subscale score |
| SFS withdrawal | Social Functioning Scale (SFS) | SFS withdrawal subscale score |
| Social behavior scale | Social behavior scale (SBS) | SBS total score |
| Social contact with non-abuser | Social contact with a non-abuser | Proportion of participants that had social contact with a non-abuser |
| Social functioning | 1. Mental Illness Research, Education, and Clinical Center version of the Global Assessment of Functioning scale (MIRECC GAF)  2. Multi-Function Needs Assessment (MFNA)  3. Strauss-Carpenter Scale | 1. MIRECC GAF social functioning score  2. MFNA social function subscale score  3. Strauss Carpenter Scale social functioning subscale score |
| Social relationships | Schizophrenia Care and Assessment Program Health Questionnaire (SCAP-HQ) | Mean item score on 3 SCAP-HQ items measuring frequency of social activities in the past 4 weeks |
| SSPA | Social Skills Performance Assessment (SSPA) | SSPA total score |
| WHO-DAS family functioning | WHO-DAS | WHO-DAS family functioning score |
| WHO-DAS social functioning | WHO's Disability Assessment Scale (WHO-DAS) | WHO-DAS social functioning score |
| **3. Independence** | | |
| *Definition:* The level of independence and independent behavior of the client including self-care, independent living and financial management. | | |
| **Outcome name** | **Measurement** | **Explanation of outcome** |
| ADAS-L ambulation | Alzheimer’s Disease Assessment Scale—Late Version (ADAS-L) | ADAS-L ambulation subscale score |
| ADAS-L dressing | Alzheimer’s Disease Assessment Scale—Late Version (ADAS-L) | ADAS-L dressing subscale score |
| ADAS-L feeding | Alzheimer’s Disease Assessment Scale—Late Version (ADAS-L) | ADAS-L feeding subscale score |
| ADAS-L toileting | Alzheimer’s Disease Assessment Scale—Late Version (ADAS-L) | ADAS-L toileting subscale score |
| ADAS-L total score | Alzheimer’s Disease Assessment Scale—Late Version (ADAS-L) | ADAS-L total score |
| Days in appartments / houses | Days spent into appartments/houses | Number of days spent into appartments or houses |
| Days of independent living | 1. Proportion of days of independent living  2. Number of days of independent living | 1. proportion of days of independent living in the past year  2. Number of days of independent living in the past year |
| FSRF meal preparation | Functional Skills Rating Form (FSRF) | FSRF meal preparation item score |
| FSRF personal hygiene | Functional Skills Rating Form (FSRF) | FSRF personal hygiene item score |
| FSRF punctuality | Functional Skills Rating Form (FSRF) | FSRF punctuality item score |
| Monthly income | Monthly income | Monthly income in HK dollar |
| Percentage (%) independent living | Schizophrenia Care and Assessment Program Health Questionnaire (SCAP-HQ) | Percentage of participants with housing arrangement not supervised by mental health professionals on the day of assessment (e.g., boarding homes, halfway houses, yes/no) per SCAP-HQ |
| Proportion of independent living | Days independent living | Proportion of days living independently the past year |
| PSP self-care | Personal and Social Performance Scale (PSPS) | PSPS self-care score |
| RFS independent living | Role Functioning Scale (RFS) | RFS independent living subscale score |
| Self-care | 1. Disability Assessment Schedule – II (DAS-II) Spanish version  2. Multi-Function Needs Assessment (MFNA) | 1. proportion of participants with an improvement in the DAS-II self-care score  2. MFNA self-care subscale score |
| Self-care problems | Personal and Social Performance scale (PSPS) | PSPS self-care problems subscale score |
| SFS independence-competence | Social Functioning Scale (SFS) | SFS independence-competence subscale score |
| SFS independence-performance | Social Functioning Scale (SFS) | SFS independence-performancel subscale score |
| WHO-DAS personal care | WHO's Disability Assessment Scale (WHO-DAS) | WHO-DAS personal care score |
| **4. Vocational functioning** | | |
| *Definition:* Involvement into (competitive) employment and education, measured by percentages of participants involved into work or education and outcome scales related to vocational functioning and involvement | | |
| **Outcome name** | **Measurement** | **Explanation of outcome** |
| Competitive job past year | Competitive employment rate | Number of subjects involved in competitive employment |
| Employment rate | Employment rate | Number of subjects who are employed |
| Hours worked | 1. Hours worked per week  2. Hours worked competitive | 1. Number of hours worked per week  2. Hours worked per week in competitive employment |
| MIRECC GAF occupational functioning | Mental Illness Research, Education, and Clinical Center version of the Global Assessment of Functioning scale (MIRECC GAF) | MIRECC GAF occupational functioning score |
| Percentage (%) at work | 1. Percentage (%) working full-time  2. Percentage (%) working effectively | 1. The percentage of participants that are working full time  2. The percentage of participants that are working effectively |
| Percentage (%) involved in work or education | Percentage in work or education | Percentage of participants involved in work or education |
| Percentage (%) paid work | 1. Percentage (%) with competitive wage earning employment  2. Schizophrenia Care and Assessment Program Health Questionnaire (SCAP-HQ) | 1. Number of participants with competitive wage earning employment  2. Paid employment in the past 4 weeks per SCAP-HQ |
| Percentage (%) working more than part-time | Percentage (%) working more than part-time | The number of patients that worked more than part-time |
| RFS work | Role functioning scale (RFS) | RFS work subscale score |
| SFS employment | Social Functioning Scale (SFS) | SFS employment subscale score |
| vocational and educational functioning | Multi-Function Needs Assessment (MFNA) | MFNA vocational and educational functioning subscale score |
| Work and study problems | Personal and Social Performance scale (PSPS) | PSPS work and study problems subscale score |
| Work days | Schizophrenia Care and Assessment Program Health Questionnaire (SCAP-HQ) | Number of days worked for pay in the past 4 weeks per SCAP-HQ |
| Work hours | Schizophrenia Care and Assessment Program Health Questionnaire (SCAP-HQ) | Number of hours worked for pay per day in the past 4 weeks per SCAP-HQ |
| Work functioning | 1. Strauss-Carpenter Scale  2. Index of ability to work | 1. Strauss-Carpenter scale work functioning subscale score  2. Total index of ability to work |
| **5. Positive symptoms** | | |
| *Definition:* Assessments of overall levels of positive symptoms or specific aspects of positive symptoms (e.g. hallucinations or delusions). | | |
| **Outcome name** | **Measurement** | **Explanation of outcome** |
| BPRS positive symptoms subscale | Brief Psychiatric Rating Scale (BPRS) | BPRS positive symptoms subscale score |
| BPRS thought disorder subscale | Brief Psychiatric Rating Scale (BPRS) | BPRS thought disorder subscale score |
| CDRPSS | CDRPSS positive symptoms | CDRPSS positive symptoms subscale score |
| CGI-S positive symptoms | Clinical Global Impression – Severity (CGI-S) | CGI-S positive symptoms subscale score |
| EPP | Ego psychopathology interview | Ego psychopathology interview score |
| PANSS Conceptual disorganization | Positive and Negative Symptom Scale (PANSS) | PANSS item P2 score: Conceptual disorganization |
| PANSS Delusions | Positive and Negative Symptom Scale (PANSS) | PANSS item P1 score: Delusions |
| PANSS Hallucinations | Positive and Negative Symptom Scale (PANSS) | PANSS item P3 score: Hallucinations |
| PANSS positive symptoms | Positive and Negative Symptom Scale (PANSS) | PANSS positive symptoms subscale score |
| Percentage psychotic symptoms | Krawiecka scale | Percentage of participants with A score of at least moderate on the positive symptom score items of the Krawiecka scale |
| Presence of auditory hallucinations | Diagnostic Intake Schedule | Presence of auditory hallucinations according to clinician ratings and the diagnostic intake schedule |
| PSYRATS-AH | Psychotic Symptom Rating Scale-Auditory Hallucinations (PSYRATS-AH) | PSYRATS-AH total score and subscale scores |
| PSYRATS-D | Psychotic Symptom Rating Scale-Delusions (PSYRATS-D) | PSYRATS-D total score and subscale scores |
| SAPS positive dimension | Scale for the Assessment of Positive symptoms (SAPS) | SAPS positive dimension score |
| SAPS positive symptoms | Scale for the Assessment of Positive symptoms (SAPS) | SAPS positive symptoms item score |
| SAPS total score | Scale for the Assessment of Positive symptoms (SAPS) | SAPS total score |
| **6. Negative symptoms** | | |
| *Definition:* Assessments of overall levels of negative symptoms or specific aspects of negative symptoms (e.g. social withdrawal or apathy). | | |
| **Outcome name** | **Measurement** | **Explanation of outcome** |
| BNSS avolition | Brief Negative symptom Scale (BNSS) | BNSS avolition subscale score |
| BNSS expressive deficits | Brief Negative symptom Scale (BNSS) | BNSS expressive deficits subscale score |
| BPRS activation | Brief Psychiatric Rating Scale (BPRS) | BPRS activation subscale score |
| BPRS anergia | Brief Psychiatric Rating Scale (BPRS) | BPRS anergia subscale score |
| BPRS negative symptoms | Brief Psychiatric Rating Scale (BPRS) | BPRS negative symptoms subscale score |
| CGI-S negative symptoms | Clinical Global Impression – Severity (CGI-S) | CGI-S negative symptoms subscale score |
| Diminished expression | High Ryods Evaluation of Negativity (HEN) Scale | Sum of individual item scores in Affect, Behavior and Speech subscales in High Ryods Evaluation of Negativity (HEN) Scale |
| HEN total score | High Ryods Evaluation of Negativity (HEN) Scale | HEN total score |
| MPRC TD total score | Marryland Psychiatric Rehabilitation Center Tardive Dyskinesia (MPRC TD) scale | MPRC TD total score |
| PANSS activation | Positive and Negative Symptom Scale (PANSS) | PANSS activation subscale score |
| PANSS amotivation | Positive and Negative Symptom Scale (PANSS) | PANSS amotivation subscale score |
| PANSS blunted affect | Positive and Negative Symptom Scale (PANSS) | PANSS item N1 score: blunted affect |
| PANSS diminished expression | Positive and Negative Symptom Scale (PANSS) | PANSS diminished expression score: sum of PANSS items blunted affect, poor rapport, lack of spontaneity and flow of conversation, and motor retardation |
| PANSS lack of spontaneity | Positive and Negative Symptom Scale (PANSS) | PANSS item N6 score: lack of spontaneity |
| PANSS negative symptoms | Positive and Negative Symptom Scale (PANSS) | PANSS negative symptoms subscale score |
| PANSS social withdrawal | Positive and Negative Symptom Scale (PANSS) | PANSS item N4 score: social withdrawal |
| Percentage negative symptoms | Krawiecka scale | Percentage with score of at least moderate on the negative symptom score items of the Krawiecka scale |
| QLS motivation level | Quality of Life Scale (QLS) | QLS score on the intrapsychic foundations domain; clinician-rated |
| SANS Anhedonia / Avolition | Scale for the Assessment of Negative symptoms (SANS) | SANS Anhedonia / Avolition subscale score |
| SANS Attention | Scale for the Assessment of Negative symptoms (SANS) | SANS Attention subscale score |
| SANS Flat affect / Alogia | Scale for the Assessment of Negative symptoms (SANS) | SANS Flat affect / Alogia subscale score |
| SANS Negative Dimension | Scale for the Assessment of Negative symptoms (SANS) | SANS Negative Dimension score |
| SANS total score | Scale for the Assessment of Negative symptoms (SANS) | SANS total score |
| **7. Disorganization symptoms** | | |
| *Definition:* Assessments of symptoms of disorganization. | | |
| **Outcome name** | **Measurement** | **Explanation of outcome** |
| BPRS disorganization | Brief Psychiatric Rating Scale (BPRS) | BPRS disorganization subscale score |
| PANSS disorganized / cognitive subscore | Positive and Negative Symptom Scale (PANSS) | PANSS disorganized / cognitive subscore |
| PANSS disorganized thoughts | Positive and Negative Symptom Scale (PANSS) | PANSS disorganized thoughts subscale score |
| SAPS disorganized subscale | Scale for the Assessment of Positive symptoms (SAPS) | SAPS disorganized subscale score |
| **8. Depressive symptoms** | | |
| *Definition:* Assessments of symptoms of depression or prevalence of comorbid mood disorders. | | |
| **Outcome name** | **Measurement** | **Explanation of outcome** |
| BPRS depression | Brief Psychiatric Rating Scale (BPRS) | BPRS depression factor score |
| CDRPSS | CDRPSS depressive symptoms | CDRPSS depressive symptoms subscale score |
| CDRS total score | Calgary Depression Rating Scale (CDRS) | CDRS total score |
| CDSS total score | Calgary Depression Scale for Schizophrenia (CDSS) | CDSS total score |
| CGI-S depression | Clinical Global Impression – Severity (CGI-S) | CGI-S depressive symptom score |
| HRSD total score | Hamilton Rating Scale for Depression (HRSD) | HRSD total score |
| MADRS total score | Montgomery–Asberg Depression Rating Scale (MADRS) | MADRS total score |
| PANSS depression | Positive and Negative Symptom Scale (PANSS) | PANSS depression subscale score |
| PANSS dysphoria | Positive and Negative Symptom Scale (PANSS) | PANSS dysphoria subscale score |
| Percentage depressive symptoms | Brief Psychiatric Rating Scale (BPRS) 24-item version | Percentage of patients displaying symptoms (Brief Psychiatric Rating Scale, BPRS) defined as one or more of the three items in each subscale receiving 3–7 points |
| **9. Personal recovery** | | |
| *Definition:* Outcomes capturing total scores of assessment instruments focused on overall personal recovery, or at least one domain of personal recovery from the CHIME framework: Connectedness, Hope and Optimism, Identity, Meaning in Life or Empowerment | | |
| **Outcome name** | **Measurement** | **Explanation of outcome** |
| Assessment of recovery | self-report assessment of recovery | Combination of MARS and MHSIP assessment of recovery scores |
| Control over life | Recovery Assessment Scale (RAS) | RAS No domination by symptoms subscale |
| Expectations | OPS quality of life score | OPS level of acceptability of the patients’ position in life relative to their expectations |
| Focus on strength and goal orientation | the Client's Assessment of Strengths, Interests, and Goals (CASIG) | CASIG total score |
|  | Recovery Assessment Scale (RAS) | RAS Goal and success orientation |
| Meaning in life | Meaning in Life Questionnaire | MLQ total score |
|  | OPS | OPS position in life subscale score |
| Optimism | Adult Trait Hope Scale | ATHS optimism subscale score |
|  | OPS quality of life score | OPS expectation subscale |
|  | Resilience Scale for Adults | Resilience Scale for Adults (RSA) planned future subscale score |
| Perceived discrimination | Perceived Devaluation and Discrimination Questionnaire | PDDQ total score |
| QPR | Questionnaire About the Process of Recovery (QPR) | QPR Total score |
| RAS | Recovery Assessment Scale (RAS) | RAS total score |
| Resilience | Connor-Davidson resilience scale (CDRISC) | CDRISC score |
|  | Resilience Scale for Adults (RSA) | RSA perception of self score |
| Satisfaction with family | Modified Lehman Quality of Life Inventory (QOLI-M) | QOLI-M Subjective Family Quality of Life Scale |
|  | SCAP-HQ | SCAP-HQ feelings about relationships with family members score |
| Satisfaction with life domains | Lehman's quality of life scale | Lehman's QOLI satisfaction with leisure subscale |
|  | Q-LES-Q-18 | Q-LES-Q-18 total score |
|  | Sense of Coherence (SOC) scale | SOC total |
|  |  | SOC meaningfulness |
|  | WHO-QOL 26/BREF | WHO-QOL BREF global life satisfaction score |
|  |  | WHO-QOL 26/BREF psychological domain score |
| Satisfaction with relationships | Lehman's quality of life scale | Lehman's QOL satisfaction with family relations subscale |
|  |  | Lehman's QOL satisfaction with social relations subscale |
|  |  | Lehman's S-QOL family contacts subscale score |
|  |  | Lehman's S-QOL activities and social contacts subscale score |
|  | Q-LES-Q | Q-LES-Q social relationships scale |
|  | SF-36 | SCAP-HQ social relationships |
|  |  | SF-36 Social Functioning item score |
|  | WHO-QOL BREF | WHO-QOL BREF satisfaction with social relationships domain score |
|  |  | WHO-QOL BREF social relationships subscale score |
| Self-concept | Brief Core Schema Scale (BCSS) | BCSS negative self subscale score |
|  |  | BCSS negative others subscale score |
|  |  | BCSS positive self subscale score |
|  |  | BCSS positive others subscale score |
| Self-control | SWN-20 | SWN-20 self-control subscale score |
| Sense of agency | Adult Trait Hope Scale | ATHS sense of agency subscale score |
| Sense of purpose | Heinrichs Quality of Life Scale | Heinrich's QLS Intrapsychic Foundation Subscale degree of motivation, sense of purpose, and curiosity item scores |
| Social cohesion | Recovery Assessment Scale (RAS) | RAS Willingness to ask for help item score |
|  |  | RAS reliance on others subscale score |
|  | Resilience Scale for Adults | RSA social competence score |
|  |  | RSA family cohesion score |
| Social integration | SWN-20 | SWN-20 social integration score |
| Social support | Social Support Questionnaire-6 | SSQ-6 total score |
| Stigma | Burden due to Stigma Experiences | B-STE total score |
|  | ISMI | ISMI mean item score excluding stigma resistence |
| Subjective rating of mental health | EQ-5D | EQ-5D total score |
|  |  | EQ-5D Health Thermometer |
|  |  | EQ-5D VAS score |
|  | Hamilton Program for Schizophrenia Voices Questionnaire (HPSVQ) | HPSVQ item 7 score: how bad do they make you feel? |
| Subjective value of interpersonal relations | Heinrichs Quality of Life Scale | Heinrich's QOL interpersonal relations score |
| **10. Subjective quality of life** | | |
| *Definition:* Outcomes that comprise multiple domains of subjective quality of life or total scores of assessment instruments focused on overall subjective quality of life. | | |
| **Outcome name** | **Measurement** | **Explanation of outcome** |
| Heinrichs QLS | Heinrichs quality of life scale | Heinrichs QLS Intrapsychic Foundations |
|  |  | Heinrichs QLS total score |
| Lehman’s QLS | Lehman's quality of life scale | Lehman's quality of life scale general life satisfaction subscale |
|  |  | Lehmans QLS total score |
| LQLP | Lancashire Quality of Life profile | LQLP total score |
| MANSA | MANSA | MANSA total score |
| QOLI-M | Modified Lehman Quality of Life Inventory (QOLI-M) | QOLI-M Overall Subjective Quality of Life Scale |
|  |  | QOLI-M total score |
| SLDS | SLDS | SLDS total score |
| SWN-20 | SWN-20 | SWN-20 total score |
| WHO-QOL 26 | WHO-QOL 26 | WHO-QOL 26 total score |
| **11. Verbal memory** | | |
| *Definition:* Short- and long-term verbal memory tasks and its recognition. | | |
| **Outcome name** | **Measurement** | **Explanation of outcome** |
| Auditory Verbal Learning | Rey AVLT score | delayed recall score of the Rey AVLT |
|  |  | total score of the Rey AVLT |
|  |  | RAVLT-B score within the CPI |
|  |  | RAVLT-B total score |
|  |  | RAVLT total score within the CPI |
|  |  | RAVLT total score |
|  |  | RAVLT total score z-score compared with baseline healthy control group score |
|  |  | RAVLT T-score after trials 1-5 |
|  |  | Rey auditory verbal learning test total words recalled |
|  |  | Rey auditory verbal learning test recognition memory score |
| Babcock story retell test | Babcock story retell test score | Babcock story retell delayed recall score |
|  |  | Babcock story retell delayed recall score |
|  |  | Babcock story retell test score |
|  |  | Babcock story retell test score |
| California Verbal Learning | CVLT scores | CVLT sumscore of trial 1-5 |
|  |  | California Verbal Learning Test total words recalled |
|  |  | CVLT mean number of hits (correct reproductions) during the first 5 trials |
|  |  | CVLT mean number of errors (incorrect reproductions) during the first 5 trials |
|  |  | CVLT total number of words recalled in the most productive of five learning trials minus the total number of words recalled in the first trial |
|  |  | California Verbal Learning Test learning score |
|  |  | CVLT delayed free recall score |
|  |  | CVLT recognition score |
|  |  | CVLT total number of words recalled in the most productive of five learning trials minus the total number of words recalled in a free-recall condition after a delay of 20 minutes |
|  |  | CVLT sum of trials 1-5 |
|  |  | CVLT z-score compared with healthy controls |
| Digit span | Digit span test score | the proportion of words recalled on learning trial 3 of the word list that were reproduced at the delayed recall. |
|  |  | Total score on the digit span test |
|  |  | Digit span forward score |
|  |  | Digit span forward z-score compared with healthy controls |
|  |  | Wechsler Adult Intelligence Scale (WAIS)-R digit span test forward score (average number of reproduced digits) |
|  |  | scores for forward digit span (average number of reproduced digits) |
|  |  | Digit span score of the Wechsler Adult Intelligence Scale (WAIS)-R-HK |
|  |  | z-scores for digit span forward on the basis of performance of healthy controls |
|  |  | scores for forward digit span (average number of reproduced digits) |
|  |  | scores for forward digit span (average number of reproduced digits) |
|  |  | Number of correct reproductions of the digit span forward test |
|  |  | proportion of correct number of digits reproduced for the neutral strings |
|  |  | proportion of correct number of digits reproduced for the neutral strings |
|  |  | Digit copying test outcome score |
| Hopkins Verbal learning | Hopkins Verbal Learning Test score | HVLT-R delayed recall score |
|  |  | HVLT-R immediate recall total learning score |
|  |  | HVLT-R standardized score |
| Logical memory | Logical memory score | Logical memory passages of the WMS-R delayed recall |
|  |  | logical memory delayed recall score of the WMS-R-HK |
|  |  | Logical memory test of the WMS-R delayed recall |
|  |  | Wechsler Memory Scale Logical Memory and Visual Reproduction delayed condition (% correct) |
|  |  | Logical memory passages of the WMS-R immediate recall |
|  |  | logical memory score of the WMS-R-HK |
|  |  | z-scores for logical memory test on the basis of performance of healthy controls |
|  |  | WMS-R logical memory z-score compared with healthy controls |
|  |  | logical memory score of the WMS-R-HK |
|  |  | Logical memory test of the WMS-R immediate recall |
|  |  | Wechsler Memory Scale Logical Memory immediate condition (% correct) |
|  |  | WMS-R logical memory z-score compared with healthy controls |
| Paired associates | Paired associates test score | Wechsler Paired Associates delayed recall score |
|  |  | Paired Association Learning test delayed recall |
| Verbal memory | Verbal memory composite scores | CANTAB verbal memory free recall score |
|  |  | The sum of the correct reproductions in the five immediate recall trials |
|  |  | Verbal learning subscale of the MCCB |
|  |  | Verbal learning subscale of the MCCB |
|  |  | Secondary verbal working memory factor z-score based on baseline level of cognition of the healthy control group |
|  |  | verbal learning z-score based on the total patient group |
|  |  | verbal memory z-scores with baseline healthy controls as reference group |
|  |  | verbal memory z-scores with baseline healthy controls as reference group |
|  |  | verbal memory z-scores with baseline healthy controls as reference group |
|  |  | Brief Assessment of Cognition in Schizophrenia (BACS) verbal memory score |
|  |  | WMS-III recognition score |
|  |  | Warrington recognition memory tests for words |
| Word list learning | Word list learning test score | 10 item word list immediate recall score |
|  |  | Performance on the first learning trial of the word list learning test |
|  |  | proportion of information learned by trial three of the word list learning test that was present at delayed recall |
|  |  | Word list learning delayed recall score, percentage of savings, as part of the CERAD cognitive battery |
|  |  | proportion of information learned by trial three of the word list learning test that was present at delayed recall |
| **12. Executive Functioning** | | |
| *Definition:* The set of processes that manifest control over other component cognitive abilities, such that cognitive resources can be effectively utilized to solve problems efficiently and plan for the future. Thus, tasks of problem solving, working memory tasks, planning, manipulating mazes, and other complex tasks where management of multiple cognitive abilities are required. | | |
| **Outcome name** | **Measurement** | **Explanation of outcome** |
| Executive functioning | Brief Assessment of Cognition in Schizophrenia (BACS) | BACS executive functions score |
|  | Cambridge Neuropsychological Test Automated Battery (CANTAB) executive functioning composite | CANTAB executive functioning composite score |
|  | Cambridge Neuropsychological Test Automated Battery (CANTAB) Visual planning, reasoning and impulsivity | CANTAB Visual planning, reasoning and impulsivity score |
|  | Executive functioning z-score | Combination of z-scores compared with healthy controls from the digit symbol test, TMT-B and Color-Word Interference Test |
|  | Executive interview | Executive interview score |
| Fluency | Category verbal fluency | z-scores for the category verbal fluency test on the basis of performance of healthy controls |
|  |  | Supermarket test of the Dementia Rating scale z-score compared with healthy controls |
|  |  | Category verbal fluency test score |
| Inhibition | Disinhibition | CNI disinhibition score |
| Picture arrangement | Picture arrangement | Wechsler Adult Intelligence Scale (WAIS) picture arrangement test score |
| Reasoning and problem solving | Problem solving z-score | Problem solving factor z-score based on baseline level of cognition of the healthy control group |
|  | Reasoning / problem solving | The reasoning / problem solving subscale of the MATRICS Consensus Cognitive Battery (MCCB) |
|  |  | reasoning and problem solving score according to the NAB |
| Similarities | Similarities | Similarities standard score of the Wechsler Adult Intelligence Scale (WAIS) |
| Stockings of Cambridge | Stockings of Cambridge (SoC) initial thinking time | Stockings of Cambridge (SoC) task initial thinking time |
|  | Stockings of Cambridge planning | Stockings of Cambridge planning score |
|  | Stockings of Cambridge (SoC) subsequent thinking time | Stockings of Cambridge (SoC) task subsequent thinking time |
|  | Stockings of Cambridge (SoC) optimal solutions | Stockings of Cambridge (SoC) task optimal solutions score |
|  |  | Stockings of Cambridge task number of sequences correctly identified |
|  | Stockings of Cambridge thinking time | Stockings of Cambridge thinking time score |
| Stroop test | Stroop test color-word naming | Stroop test color-word naming task |
|  |  | Stroop test color-word naming task |
|  |  | Stroop test color-word naming task |
|  | Stroop time color-word inference test | Stroop Color-Word-Interference test score |
|  |  | Stroop test color-word inference task |
|  |  | Stroop color-Word-Interference test number of correct responses |
|  |  | Stroop test color-word interference task |
|  |  | Stroop 3 color and word interference test |
| Trail Making Test | Trail Making Test B | TMT part B time spent on completion |
|  |  | TMT part B time spent on completion |
|  |  | Trail Making Test B time in seconds to completion |
|  |  | Trail making test B time in seconds to completion |
|  |  | Trail making test B score within the CPI |
|  |  | TMT part B number of seconds spent on completion |
|  |  | TMT part B number of seconds spent on completion |
|  |  | Trail making test part B number of seconds spent on completion |
|  |  | TMT part B number of seconds spent on completion |
|  |  | TMT part B standardized test score |
|  |  | TMT part B number of seconds spent on completion |
|  |  | TMT-B number of seconds until completion |
|  |  | TMT part B number of seconds spent on completion |
|  |  | TMT part B number of seconds spent on completion (negative) |
|  |  | TMT part B z-score compared with healthy controls |
|  |  | Trail Making part B number of seconds spent on completion |
|  |  | Trail making test part B number of seconds spent on completion |
| Word association | Controlled Oral Word Association Test (COWAT) | Total number of generated words generated in one minute for the letters F, A and S |
|  | Controlled oral word association test (FAS) | Controlled oral word association test (FAS) T-score |
| Wisconsin Card Sorting test | Wisconsin Card Sorting Test (WCST) categories | WCST number of categories |
|  |  | MWCST number of categories completed |
|  |  | WCST categories completed z-score compared to healthy controls |
|  |  | Modified (M)WCST number of categories completed |
|  |  | WCST categories completed |
|  |  | WCST number of categories completed |
|  |  | Wisconsin Card Sorting test categories completed |
|  |  | WCST categories completed |
|  |  | WCST categories completed |
|  |  | MWCST categories completed |
|  |  | WCST number of categories completed |
|  |  | Wisconsin Card Sorting test number of categories completed |
|  |  | WCST categories completed |
|  |  | WCST categories score |
|  |  | WCST number of categories completed |
|  |  | WCST categories score |
|  | Wisconsin Card Sorting Test (WCST) set maintenance | WCST failure to maintain sets |
|  |  | WCST failure to maintain sets |
|  | Wisconsin Card Sorting Test (WCST) perseverations | MWCST number of perseverations |
|  |  | WCST perseverative errors z-score compared to healthy controls |
|  |  | MWCST number of categories completed |
|  |  | WCST number of perservative errors |
|  |  | WCST number of perseverative errors |
|  |  | Wisconsin Card Sorting test number of perseverative errors |
|  |  | WCST % perseverative errors |
|  |  | MWCST perseverative errors |
|  |  | WCST number of perseverative errors |
|  |  | Wisconsin Card Sorting test percentage of perseverations |
|  |  | WCST perseverative errors |
|  |  | WCST perseverations score |
|  |  | WCST perseverative errors |
|  |  | WCST number of perseverative responses |
|  |  | WCST number of perservations |
|  |  | WCST number of perservations |
|  |  | WCST perseverative responses |
|  | Wisconsin Card Sorting Test (WCST) total errors | WCST number of total errors |
|  |  | WCST number of total errors |
|  |  | WCST number of total errors |
|  |  | WCST total number of errors |
|  | Wisconsin Card Sorting Test (WCST) trials to first category | WCST trials to first category |
|  |  | WCST trials to first category |
|  | Wisconsin Card Sorting Test z-score | z-scores for the Modified Wisconsin Card Sorting test on the basis of performance of healthy controls |
|  |  | Mean WCST categories and perseverative errors z-score compared with healthy controls |
| Working memory | Digit span backward | Digit span backward score |
|  |  | WAIS-R digit span test backward score (average number of reproduced digits) |
|  |  | WAIS-R digit span test backward score (average number of reproduced digits) |
|  |  | WAIS-R digit span test backward z-score compared with healthy controls |
|  |  | scores for backward digit span (average number of reproduced digits) |
|  |  | scores for backward digit span (average number of reproduced digits) |
|  | Digit span with distractor | proportion of correct number of digits reproduced for the distractor strings |
|  |  | proportion of correct number of digits reproduced for the distractor strings |
|  | Working memory | BACS working memory score |
|  |  | Reading span and digit span forward (WAIS) z-score compared with healthy controls |
|  |  | Working memory subscale of the MCCB |
|  |  | DSDT percent correct |
|  |  | Working memory z-score with baseline normal controls score as reference |
|  |  | Working memory task within the MCCB |
|  |  | Working memory factor z-score based on baseline level of cognition of the healthy control group |
|  |  | Working memory / fluency z-score compared with the whole patient group |
| **13. Processing speed** | | |
| *Definition:* Cognitive processing assessments that require rapid performance of tasks that range from very simple to complex. | | |
| **Outcome name** | **Measurement** | **Explanation of outcome** |
| Color trial line | Color Trial Line 2 | Color Trial Line 2 total time taken to complete lines |
| Digit symbol test | Digit symbol | number of correctly assigned symbols |
|  |  | Wechsler Adult Intelligence Scale (WAIS) digit symbol score |
|  |  | Digit symbol test score |
|  |  | Digit symbol test written score |
|  |  | Digit symbol test oral score |
|  |  | Wechsler Adult Intelligence Scale (WAIS)-III digit symbol total count |
|  |  | Wechsler Adult Intelligence Scale (WAIS)-R digit symbol test number of symbols reproduced in 90 seconds |
|  |  | Wechsler Adult Intelligence Scale (WAIS)-III digit symbol score |
|  |  | Wechsler Adult Intelligence Scale (WAIS)-R digit symbol test number of symbols reproduced in 90 seconds |
|  |  | Wechsler Adult Intelligence Scale (WAIS)-R digit symbol test z-score compared with healthy controls |
|  |  | The number of symbols correctly completed in 90 seconds |
|  |  | digit symbol test score |
| Processing speed | MATRICS Consensus Cognitive Battery (MCCB) Speed of processing | MCCB processing speed |
|  |  | MCCB processing speed |
|  | Brief Assessment of Cognition in Schizophrenia (BACS) processing speed | BACS processing speed score |
|  | visual motor / processing speed | Visual motor / processing speed z-score with baseline healthy controls as reference |
| Stroop test | Stroop test color naming | Stroop test color naming task |
|  |  | Stroop test color naming task |
|  |  | Stroop test color naming task |
|  |  | Stroop 2 color naming test |
| Symbol search | Wechsler Adult Intelligence Scale (WAIS)-III symbol search | Wechsler Adult Intelligence Scale (WAIS)-III symbol search total score |
| Trail making test | Trail making test A | TMT part A time spent on completion |
|  |  | TMT-A score |
|  |  | Trail making test A score within the CPI |
|  |  | TMT-A score |
|  |  | TMT part A number of seconds spent on completion |
|  |  | TMT part A number of seconds spent on completion |
|  |  | Trail making test part A number of seconds spent on completion |
|  |  | TMT part A number of seconds spent on completion |
|  |  | TMT part A standardized test score |
|  |  | TMT part A number of seconds spent on completion |
|  |  | TMT-A number of seconds until completion |
|  |  | Trail making test part A number of seconds spent on completion |
|  |  | TMT part A number of seconds spent on completion |
|  |  | Trail Making part A number of seconds spent on completion |
|  |  | Trail making test part A number of seconds spent on completion |
| **14. Overall cognition** | | |
| *Definition:* Overall cognition composite scores, or assessments of intelligence that is not categorized in any domains. | | |
| **Outcome name** | **Measurement** | **Explanation of outcome** |
| Cognitive composite score | Average impairment rating T-score | neuropsychological T-scores |
|  | Cognitive composite score | The composite score of all separate cognitive test and standardized by comparing it with the baseline cognitive composite score of healthy controls (z-score) |
|  |  | Composite z-score of variety of cognitive tests compared with healthy controls |
|  |  | Brief Assessment of Cognition in Schizophrenia (BACS) cognitive index score |
|  |  | Brief Assessment of Cognition in Schizophrenia (BACS) cognitive z-score compared with healthy controls |
|  |  | The composite score of all separate cognitive test |
|  |  | Global cognitive z-score compared with healthy controls |
|  |  | MATRICS Consensus Cognitive Battery (MCCB) cognitive composite score calculated by means of norm data published in Nuechterlein & Green, 2006 |
|  |  | averaged standardized scores of cognitive functioning on several domains at baseline and follow-up |
|  |  | cognitive function score based on 10 questions |
|  |  | z-score of all neurocognitive measures compared with the normal control group |
|  | Comprehensive Module Test | CMT score |
|  | Executive functioning / reasoning | z-score of the executive functioning / reasoning subsection compared with a normal control group |
|  | Executive functioning - processing speed | z-score of all executive functioning and speed of processing compared with a healthy control group at baseline |
|  |  | z-score of all executive functioning and speed of processing compared with a healthy control group at baseline |
|  | Global neurocognitive T-score | Global neuropsychological test T-score for the comprehensive battery of neurocognitive tests |
|  |  | Global neuropsychological T-score from the Halstead Reitan battery |
|  | Neurocognitive composite score | Neurocognitive composite z-score from the CERAD with healthy controls as reference group |
|  |  | composite score for cognitive functioning based on results of tests of word list learning, praxic drawings, and the Modified Boston Naming Test |
|  |  | MATRICS Consensus Cognitive Battery (MCCB) composite neurocognitive score z-score with healthy controls as comparison |
|  | Neuropsychological performance | Composition of different neuropsychological test performancesconverted to Z-scores from published norms |
| Intelligence | IQ (total) | Wechsler Adult Intelligence Scale (WAIS)-R total IQ score |
|  |  | Wechsler Adult Intelligence Scale (WAIS)-R total IQ score |
|  |  | Wechsler Adult Intelligence Scale (WAIS)-R total IQ score |
|  |  | Wechsler Adult Intelligence Scale (WAIS) total IQ score |
|  |  | Wechsler Adult Intelligence Scale (WAIS) total IQ score |
|  | Matrices | IQ score of the progressive matrices test |
|  | Wechsler Adult Intelligence Scale (WAIS)-R | Wechsler Adult Intelligence Scale (WAIS)-R total IQ difference score between baseline and follow-up |
|  | Wechsler Adult Intelligence Scale (WAIS)-R IQ | Wechsler Adult Intelligence Scale (WAIS)-R IQ score |
|  | Wide Range Achievement Test – Revised (WRAT-R) reading | WRAT-R reading score |
|  |  | WRAT-R IQ score |
| Overall cognition | MATRICS Consensus Cognitive Battery (MCCB) total cognition | MCCB cognitive composite score |
|  | Mini Mental State Examination (MMSE) | MMSE total score |
| Performance IQ | Performance IQ | Wechsler Adult Intelligence Scale (WAIS)-R performance IQ difference score between baseline and follow-up |
|  |  | Wechsler Adult Intelligence Scale (WAIS)-R performance IQ score |
|  |  | Wechsler Adult Intelligence Scale (WAIS) performal IQ score |
| Verbal IQ | Verbal IQ | Wechsler Adult Intelligence Scale (WAIS) verbal IQ z-score compared with healthy controls |
|  |  | Wechsler Adult Intelligence Scale (WAIS)-R verbal IQ difference score between baseline and follow-up |
|  |  | Pro-Rated Verbal IQ score |
|  |  | Wechsler Adult Intelligence Scale (WAIS)-R verbal IQ score |
|  |  | Wechsler Adult Intelligence Scale (WAIS) verbal IQ score |

# **Supplementary material 4.** Flow chart of included studies

Additional records identified through other sources
(k = 133)

Records identified through database searching
(k = 12,457)

## Identification

Records after duplicates removed
(k = 12,304)

## Screening

Records screened
(k = 12,304)

Records excluded
(k = 11,296)

**Full-text articles excluded**

**(total: k = 842):**

**No longitudinal study design: k=360**

**Wrong patient population: k=140**

**No combination of at least two outcome domains reported: k=257**

**Data is not extractable: k=81**

**Not published in English: k=4**

Full-text articles assessed for eligibility
(k = 1,008)

## Eligibility

Articles included in quantitative synthesis (meta-analysis)
(k = 166)

## Included

Studies included in quantitative synthesis (meta-analysis)
(k = 109)

**Supplementary material 5**. Descriptive statistics of included studies

| **Study name*** | **N**  **(baseline-FU)** | **Age (SD)** | **% female** | **Primary diagnosis** | **Comorbidity** | **Treatment** | **Baseline DOI (y)** | **FU duration (y)** | **Attrition rate** | **Outcome domains reported**** |
| --- | --- | --- | --- | --- | --- | --- | --- | --- | --- | --- |
| Addington 2000^S1^ | 80-65 | 33.2 (8.9) | 21.1% | Schizophrenia (100%) | unclear | Antipsychotics (100%); routine care (100%) | 11.2y | 2.5 | 18.8% | NEG; OSF; POS; PSB; QOL |
| Aguilar 2018^S2^ | 40-40 | 36.6 (6.8) | 37.5% | Schizophrenia (100%) | unclear | Antipsychotics (75.0%); CBT (42.5%) | Unclear | 0.8; 1.25 | 28.3% | NEG; OSF; POS |
| Albus 2002^S3,S4^ | 58-58 | 29.7 (9.1) | 49.3% | Schizophrenia (100%) | unclear | butyrophenones (100%) | 6.2y | 2; 5 | 30.0% | DEP; EXF; NEG; OVC; POS; PRS; VEM |
| Alphs 2022^S5^ | 273-112 | 23.2 (4.4) | 22.0% | Schizophrenia (76.9%); Schizophreniform disorder (23.1%) | unclear | Antipsychotics (100%) | 0.9y | 0.8; 1.5 | 38.1% | DEP; NEG; OSF; POS |
| Breier 2018^S6^ | 60-60 | 23.6 (4.9) | 21.7% | Schizophrenia (68.3%); schizophreniform disorder (13.3%); Schizoaffective disorder (8.3%); Psychotic disorder NOS (10.0%) | unclear | Antipsychotics (100%) | 1.4y | 1 | 46.7% | NEG; OSF; OVC; POS |
| Buonocore 2018^S7^ | 60-60 | 34.9 (9.7) | 45.3% | Schizophrenia (100%) | unclear | Computer-assisted CRT (100%); standard rehabilitation therapy (SRT; 100%); Risperidone (23.0%); Haloperidol (15.0%); Clozapine (39.0%); Olanzapine (7.0%); Aripiprazole (8.0%); Paliperidone (2.0%); Fluphenazine (3.0%); Chlorpromazine (3.0%) | 10.8y | 5 | 6.3% | EXF; OVC; PRS; QOL; VEM |
| Cai 2022^S8^ | 277-277 | 46.0 (12.7) | 55.6% | Schizophrenia (100%) | unclear | Antipsychotics (100%); Medication monitoring intervention (50.0%); Community mental health program (100%) | 17.9y | 0.5; 2.3 | 7.6% | DEP; NEG; OSF; OVC; POS |
| Cechnicki 2017^S9^ | 80-65 | 26.6 (5.8) | 56.7% | Schizophrenia (100%) | unclear | Community treatment program (50.0%); Individual treatment program (50.0%) | 0.8y | 3; 12 | 16.3% | NEG; OSF; POS |
| Chan 2003^S10^ | 25-25 | 40.4 (7.8) | 44.0% | Schizophrenia (100%) | unclear | Not reported | 15.4y | 0.3; 0.7; 1 | 16.0% | OSF; PRC |
| Chan 2018^S11,S12^ | 148-107 | 20.9 (3.1) | 49.3% | Schizophrenia spectrum disorder (100%) | unclear | Early intervention service (100%) | 0.0y | 3 | 27.7% | NEG; POS; VOF |
| Chanpattana 2010^S13^ | 253-253 | 34.1 (8.0) | 53.8% | Schizophrenia (100%) | unclear | Electroconvulsive therapy; flupenthixol | 13.3y | 1.6 | 0.0% | NEG; OVC; POS |
| Chen 2000^S14^ | 50-43 | 48.9 (8.9) | 30.2% | Schizophrenia (100%) | unclear | Not reported | 23.5y | 3 | 14.0% | EXF; NEG; POS |
| Chen 2005^S15-S22^ | 138-88 | 31.7 (9.2) | 54.8% | Schizophrenia (80.7%); schizophreniform disorder (14.0%); schizoaffective disorder (5.4%) | unclear | antipsychotics (48.4%); antidepressants (12.9%); benzodiazepines (12.9%) | 1.5y | 1; 2; 3 | 39.2% | DEP; EXF; NEG; POS; VEM; VOF |
| Chien 2017^S23-S26^ | 333-333 | 25.6 (7.7) | 36.8% | Schizophrenia (52.1%); Schizophreniform disorder (12.0%); Schizoaffective disorder (22.8%); Other psychotic disorders (13.2%) | unclear | Medical consultation (99.7%); Psychoeducation (58.5%); Social welfare interventions (59.9%); Individual counseling (26.0%) | 2.6y | 0.5; 1; 1.5; 2.5 | 16.4% | IND; NEG; OSF; POS; PRC; PSB |
| Ciudad 2009^S27-S28^ | 1005-375 | 37.7 (10.5) | 35.6% | Schizophrenia (100%) | substance / alcohol abuse (34.3%) | Not reported | 13.7y | 1 | 16.8% | DEP; IND; NEG; OSF; POS; PRC; PSB |
| Conley 2007^S29^ | 2228-1167 | 41.8 (11.2) | 38.5% | Schizophrenia (57.2%); schizoaffective disorder (33.6%); other psychotic disorder (9.3%) | substance use disorder (28.0%); personality disorder (14.5%); depressive disorder (39.4%) | Antidepressants (38.8%); Anti-anxiety agents (11.3%); Mood stabilizers (31.2%); Hypnotics (1.7%); Antiparkinsonian agents (44.8%); atypical antipsychotics (59.8%); Typical antipsychotics (58.2%) | 21.6y | 3 | 4.3% | IND; NEG; OSF; PRC; PSB; QOL; VOF |
| Cullberg 2002^S30,S31^ | 120-115 | 28.2 (7.1) | 45.0% | schizophrenia syndromes (schizophrenia, schizophreniform psychosis and schizoaffective psychosis; 40.8%); non-schizophrenia syndromes (delusional disorder, brief psychosis and psychotic disorder not otherwise specified (NOS); 59.2%) | unclear | Need adapted treatment (100%); antipsychotics (41.8%); benzodiazepines (70.6%); antidepressants or lithium (44.7%) | 0.0y | 1; 3; 5 | 30.8% | DEP; NEG; OSF |
| Dal Santo 2020^S32^ | 17-17 | 45.4 (8.1) | 0.0% | Schizophrenia (100%) | unclear | Clozapine (100%) | Unclear | 2.9 | 0.0% | OVC; POS |
| Dellazizzo 2023^S33^ | 74-30 | 42.5 (12.7) | 24.3% | Schizophrenia (77.0%); Schizoaffective disorder (23.0%) | unclear | Atypical antipsychotics (96.0%); Virtual reality (VR)-assisted therapy (50.0%); Cognitive behavioral therapy (50.0%) | 16.0y | 0.5; 1 | 16.2% | DEP; DIS; NEG; POS; PRC |
| Dixon 2015^S34-S36^ | 65-65 | 22.2 (4.2) | 36.9% | Schizophrenia (66.2%); schizoaffective disorder (13.9%); schizophreniform disorder (6.2%); Psychosis NOS (4.6%); Brief psychotic disorder (1.6%); no diagnosis (3.1%); unknown (4.6%) | Bipolar disorder NOS (3.1%); Depressive disorder NOS (23.1%); Panic disorder (4.6%); Social phobia (3.1%); obsessive compulsive disorder (1.5%); PTSD (7.7%); anxiety disorder NOS (4.6%); alcohol use disorder (18.5%); sedative-hypnotic-anxiolytic use disorder (1.5%); Cannabis use disorder (33.9%); Stimulant use disorder (1.5%); Opioid use disorder (3.1%); Cocaine use disorder (4.6%); Hallucinogen use disorder (4.6%) | Treatment connection program (100%) | <2y | 0.5; 1; 1.5; 2 | 69.2% | NEG; POS; PRC; PSB; QOL; VOF |
| Ekerholm 2012^S37^ | 36-36 | 41.1 (7.9) | 13.9% | Schizophrenia (100%) | unclear | Antipsychotics (95.8%) | 17.6y | 4.6 | 49.3% | EXF; OSF; PRS; VEM |
| Evensen 2016^S38^ | 148-148 | 32.9 (7.9) | 30.6% | Schizophrenia (88.5%); schizoaffective disorder (7.5%); Osychosis NOS (2.0%); Delusional disorder (2.1%) | unclear | CBT (56.8%); Cognitive remediation (43.2%) | 7.2y | 2 | 12.2% | DEP; OSF; VOF |
| Fernandez-Modamio 2021^S39^ | 299-188 | 44.3 (13.4) | 39.5% | Schizophrenia or schizoaffective disorder | unclear | Social Cognition Training Program (SCTP; 50.0%); Neurocognitive training (100%); Antipsychotics (100%) | 22.9y | 0.5; 1 | 37.1% | IND; NEG; POS; PRC; PSB; QOL |
| Fond 2018^S40^ | 549-315 | 32.6 (9.7) | 22.0% | Schizophrenia (75.7%); Schizoaffective disorder (24.3%) | Alcohol use disorder (6.2%); Cannabis use disorder (7.9%); Anxiety disorder (36.7%); Eating disorder (2.6%) | Antipsychotics (84.6%) | 11.0y | 2 | 42.0% | DEP; DIS; EXF; NEG; OSF; OVC; POS; PRS; VEM |
| Foti 2010^S41-S45^ | 248-248 | 28.5 (8.5) | 34.6% | Schizophrenia spectrum disorder (100%) | unclear | Not reported | 2.4y | 0.5; 2; 4; 10; 20 | 28.0% | DEP; DIS; EXF; NEG; OSF; POS; PRS; VEM; VOF |
| Fowler 2012^S46^ | 301-257 | 37.6 (11.0) | 30.0% | Schizophrenia (85.0%); Schizoaffective disorder (13.0%); Delusional disorder (2.0%) | unclear | Not reported | 10.7y | 0.25; 1 | 18.2% | DEP; POS; PRC |
| Fowler 2018^S47^ | 148-143 | 24.5 (7.9) | 21.8% | Non-affective psychosis (100%) | unclear | Early Intervention Services (100%); Social recovery therapy (49.0%) | 2.1y | 0.8; 1.3 | 17.4% | DEP; NEG; PRC; VOF |
| Galderisi 2020^S48^ | 921-618 | 40.2 (10.7) | 30.4% | Schizophrenis (100%) | Substance abuse (5.0%); Alcohol abuse (4.9%) | Antipsychotics (76.8%); Integrated treatment (26.8%) | 16.2y | 4 | 32.9% | DEP; DIS; EXF; IND; NEG; OSF; POS; PRC; PRS; PSB; VEM; VOF |
| Ganella 2018^S49^ | 29-14 | 21.3 (2.0) | 24.1% | First Episode Psychositic Disorder (100%) | unclear | Not reported | 1.4y | 1 | 51.7% | DEP; NEG; OSF; POS |
| Gaughran 2017^S50^ | 403-259 | 44.2 (10.1) | 42.4% | Psychotic disorder (100%) | unclear | Health promotion intervention (52.5%) | Unclear | 1; 1.3 | 25.9% | DEP; OSF |
| Godin 2019^S51^ | 770-325 | 32.7 (9.9) | 26.0% | Schizophrenia (100%) | Anxiety disorder (37.4%); Tobacco smoking (51.6%); Cannabis use disorder (28.4%); Alcohol use disorder (20.1%) | Antipsychotics (21.2%); antidepressants (25.7%) | 10.7y | 1 | 61.4% | DEP; OSF; PRC |
| Gorna 2008^S52^ | 125-125 | 24.7 (6.7) | 37.8% | Schizophrenia (100%) | unclear | Not reported | <2y | 1; 5 | 21.3% | IND; NEG; OSF; POS; PRC; PSB; QOL; VOF |
| Gorwood 2019^S53^ | 303-228 | 29.3 (4.9) | 26.4% | Schizophrenia (100%) | unclear | Antipsychotics (100%); Benzodiazepines (38.3%); Other psychotropic treatment (59.4%); Psychotherapy (57.3%); Psychosocial care (37.8%) | 7.6y | 0.5; 1 | 26.1% | NEG; OSF; POS |
| Granholm 2020^S54^ | 107-101 | 56.0 (7.5) | 17.5% | Schizophrenia (80.7%); Schizoaffective disorder (19.3%) | unclear | Cognitive-behavioral social skills training (CBSST; 45.6%); Mobile assisted CBSST (MA-CBSST; 29.8%) | Unclear | 0.3; 0.5; 1 | 40.4% | IND; NEG; OVC; POS; PSB |
| Grawe 2006^S55^ | 50-50 | 25.4 (4.6) | 38.0% | Schizophrenia (80.0%); Schizoaffective disorder (12.0%); Schizophreniform disorder (8.0%) | unclear | Antipsychotics (100%); Integrated treatment (60.0%); Standard treatment (40.0%) | <2y | 2 | 14.0% | NEG; OSF; POS |
| Gumley 2022^S56^ | 73-62 | 43.0 (12.0) | 49.3% | Schizophrenia spectrum disorder (100%) | unclear | Empwer intervention (57.5%); TAU (42.5%) | Unclear | 0.5; 1 | 17.8% | DEP; DIS; NEG; OSF; POS; PRC |
| Harrow 2005^S57-S63^ | 239-239 | 22.9 (2.3) | 44.0% | Schizophrenia (40.8%); Schizophreniform disorder (7.6%); Other psychotic disorder (51.6%) | unclear | Not reported | <2y | 2; 4.5; 7.5; 10; 15 | 8.3% | NEG;OSF; POS; PSB; VOF |
| Harvey 2010^S64^ | 61-61 | 57.0 (9.0) | 27.0% | Schizophrenia (100%) | unclear | Second generation antipsychotics (100%) | 33.3y | 3.8 | 45.1% | OSF; OVC; PSB |
| Hayhurst 2014^S65^ | 363-301 | 39.5 (11.4) | 32.0% | Schizophrenia, schizoaffective, schizophreniform or delusional disorder, | unclear | Antipsychotics (100%) | 11.6y | 1 | 18.1% | DEP; NEG; POS; QOL |
| Heeramun-Aubeeluck 2015^S66^ | 38-38 | 25.9 (7.3) | 51.5% | Schizophrenia (100%) | unclear | Aripiprazole (33.7%); Olanzapine (32.7%); Risperidone (32.7%) | Unclear | 0.5; 1 | 62.4% | PRS; PSB; VEM |
| Heering 2015^S67^ | 1022-602 | 27.7 (8.0) | 23.8% | Schizophrenia, schizofreniform disorder or schizoaffective disorder | unclear | Not reported | 4.4y | 3.3 | 42.1% | OSF; OVC; QOL |
| Hoff 2005^S68^ | 21-21 | 37.9 (5.7) | 28.6% | Schizophrenia (74.3%); Schizoaffective disorder (5.7%) | unclear | Antipsychotics (92.9%) | 1.5y | 10 | 58.0% | EXF; NEG; OVC; POS; PRS; VEM |
| Horan 2012^S69^ | 55-55 | 22.3 (4.3) | 23.6% | Schizophrenia (56.8%); schizoaffective disorder (12.4%); schizophreniform disorder (30.9%) | unclear | Risperidone (100%) | 0.7y | 1 | 32.1% | IND; NEG; POS; PSB; VOF |
| Hui 2023^S70^ | 360-360 | 32.1 (10.4) | 48.7% | Schizophrenia spectrum disorder (100%) | unclear | Antipsychotics (48.7%) | 0.0y | 1; 2; 3 | unclear | DEP; IND; NEG; OSF; POS; PSB; VOF |
| Ito 2015 ^S71^ | 155-111 | 30.6 (10.1) | 53.2% | Schizophrenia spectrum disorder (100%) | unclear | Antipsychotics (100%) | 2.0y | 0.5; 1; 1.5 | 53.9% | NEG; OSF; POS; QOL |
| Jørgensen 2015 ^S72^ | 101-94 | 37.5 (12.6) | 53.5% | Schizophrenia (92.1%); Schizoaffective disorder (7.9%) | unclear | Antipsychotics (100%); Guided self-determination intervention (100%) | 9.8y | 0.3; 0.5; 1 | 7.9% | NEG; POS; PRC |
| Kane 2016 ^S73,S74^ | 404-404 | 23.1 (5.1) | 27.5% | Schizophrenia (53.0%); Schizoaffective disorder, bipolar (5.9%); Schizoaffective disorder, depressive (14.1%); schizophreniform disorder (16.6%); Brief psychotic disorder (0.5%); Psychotic disorder NOS (9.9%) | alcohol abuse / dependence (36.4%); Cannabis abuse / dependence (35.6%) | Antipsychotics (83.4%); personalized medication management, family psychoeducation, resilience-focused individual therapy and supported employment and education (55.2%); Community care (44.8%) | 3.7y | 0.5; 1; 1.5; 2 | 43.8% | DEP; DIS; NEG; POS; PRC; QOL; VOF |
| Kelly 2009 ^S75^ | 43-43 | 44.1 (8.3) | 27.9% | Schizophrenia (100%) | unclear | Haloperidol (58.1%); olanzapine (41.9%) | 22.1y | 1 | 23.2% | OSF; POS; QOL |
| Kim 2019 ^S76^ | 87-87 | 33.6 (9.8) | 49.4% | Schizophrenia (100%) | unclear | Antipsychotics (100%) | 8.8y | 0.5; 1 | 29.9% | POS; PRC |
| Klærke 2019 ^S77^ | 70-70 | 26.5 (6.2) | 28.6% | Schizophrenia (95.7%); Schizoaffective disorder (4.3%) | unclear | Substance abuse (11.0%) | 2.1y | 9.6 | 51.1% | NEG; OSF; POS |
| Koshiyama 2017 ^S78^ | 14-14 | 22.6 (5.4) | 21.4% | First Episode Psychosis (100%) | unclear | antipsychotics (100%) | 0.7y | 1.9 | unclear | NEG; OSF; POS |
| Lasser 2005 ^S79^ | 582-582 | 40.9 (13.0) | 33.9% | Schizophrenia (83.4%); schizoaffective disorder (16.6%) | unclear | Antipsychotics (98.8%) | Unclear | 1 | 20.3% | DIS; NEG; POS; PRC |
| Lee 2023^S80^ | 54-54 | 32.3 (9.3) | 38.9% | Schizophrenia or schizoaffective disorder | unclear | Clozapine (100%) | 12.7y | 14.1 | 40.7% | EXF; NEG; OVC; POS; PRS; QOL; VEM |
| Li 2017 ^S81,S82^ | 63-63 | 23.1 (5.1) | 27.5% | Schizophrenia or schizophreniform disorder | alcohol abuse / dependence (36.4%); Cannabis abuse / dependence (35.6%) | Antipsychotics (83.4%); personalized medication management, family psychoeducation, resilience-focused individual therapy and supported employment and education (55.2%); Community care (44.8%) | 3.7y | 0.5; 1; 1.5; 2 | 43.8% | DIS; NEG; OSF; POS |
| Lindgren 2020 ^S83^ | 52-32 | 26.7 (5.7) | 40.4% | Schizophrenia (50.0%); schizophreniform disorder (21.2%); psychotic disorder NOS (23.1%); Brief psychotic disorder (5.8%) | unclear | Antipsychotics (94.2%); antidepressants (26.9%) | 0.0y | 1 | 38.5% | DEP; EXF; OSF; POS; PRS; VEM |
| Litman 2023 ^S84^ | 215-175 | 39.3 (10.8) | 39.1% | Schizophrenia (100%) | unclear | Risperidone (100%) | 10.9y | 0.2; 0.5; 1 | 10.7% | DEP; IND; OSF; PRC; PSB; QOL |
| Liu 2023^S85^ | 96-76 | 36.3 (10.2) | 55.2% | Schizophrenia / schizophreniform disorder (78.1%); Other schizophrenia spectrum disorder (21.9%) | unclear | Antipsychotics (100%) | 11.3y | 2 | 20.8% | DEP; OSF; PRC |
| Lopez-Morinigo 2023^S86^ | 34-28 | 47.7 (9.6) | 46.8% | Schizophrenia spectrum disorder (100%) | unclear | Metacognitive training (50.6%); Psychoeducation (49.4%); Clozapine (15.6%); other antipsychotics (58.4%) | >5y | 0.2; 1 | 62.8% | DEP; DIS; NEG; OSF; POS; QOL |
| McGurk 2003^S87^ | 30-27 | 39.7 (6.9) | 23.3% | Schizophrenia (53.3%); schizaffective disorder (46.7%) | unclear | Supported employment (100%); antipsychotics (100%); clozapine (23,33%); risperidone (26,67%); olanzapine (13,33%) | 15.7y | 2 | 10.0% | EXF; NEG; POS; PRS; VEM |
| McNeely 2023 ^S88^ | 51-40 | 45.5 (13.7) | 41.2% | Schizophrenia and related psychotic disorders (100%) | unclear | Self-management Engaging Together (SET) for Health (100%) | 21.3y | 1.1 | 21.6% | OSF; PRC; QOL |
| Meade 2020 ^S89^ | 412-238 | 38.7 (10.6) | 42.2% | Schizophrenia (100%) | unclear | Antipsychotics (100%) | 11.3y | 0.5; 1 | 56.8% | DIS; IND; NEG; OSF; POS; PSB |
| Meagher 2004 ^S90^ | 82-82 | 68.7 (10.1) | 41.9% | Schizophrenia (100%) | unclear | Antipsychotics (100%) | 44.7y | 2.9 | 36.4% | EXF; NEG; OVC; POS |
| Melle 2008^S91,S92^ | 281-186 | 30.0 (10.0) | 44.3% | schizophrenia spectrum disorder (72.1%) | Alcohol use problems (7.5%); Drug use problems (10.0%) | First-episode treatment programs consisting of antipsychotic psychopharmacology, assertively oriented individual outpatient treatment, and psychoeducational family work. | <2y | 0.3; 1; 2; 10 | 33.2% | IND; NEG; OSF; POS; PSB |
| Moncrieff 2023 ^S93^ | 253-175 | 46.3 (11.8) | 32.4% | Schizophrenia (68.8%); Other psychotic disorders (31.2%) | unclear | Antipsychotics (100%) | >5y | 0.5; 1; 2 | 24.9% | EXF; NEG; OSF; POS; PRC; PRS; QOL; VEM; VOF |
| Morrison 2018 ^S94^ | 75-65 | 23.6 (6.1) | 42.7% | Schizophrenia, schizoaffective disorder, or delusional disorder | unclear | CBT (68.0%); antipsychotics (65.3%) | <2y | 0.5; 1 | 20.0% | DEP; NEG; OSF; POS; PRC; QOL |
| Na 2016 ^S95^ | 25-25 | 28.2 (6.4) | 48.0% | Schizophrenia (60.0%); Schizoaffective disorder (12.0%); Psychotic disorder NOS (28.0%) | unclear | Antipsychotics (100%); Mind flower programs (100%) | Unclear | 0.5; 1 | 4.0% | NEG; OSF; POS; PRC; PSB; VOF |
| Najarian 2023 ^S96^ | 178-178 | 40.4 (10.8) | 29.2% | Schizophrenia (100%) | unclear | Antipsychotics (100%) | 12.3y | 0.5; 1; 2 | 13.5% | DIS; NEG; OSF; POS |
| Nakamura 2019 ^S97^ | 37-37 | 61.0 (7.8) | 43.2% | Schizophrenia (100%) | unclear | IMR (100%) | 34.7y | 1.03 | 14.0% | NEG; OSF; POS |
| Neill 2022 ^S98^ | 85-43 | 39.7 (9.3) | 71.8% | Schizophrenia (91.9%); Schizoaffective disorder (8.1%) | unclear | Clozapine (100%) | 15.7y | 0.2; 0.5; 1 | 49.4% | DEP; EXF; NEG; OVC; POS; PRS; QOL; VEM |
| Nordentoft 2006 ^S99-S103^ | 255-255 | 26.6 (24.4) | 54.2% | Schizophrenia spectrum disorder (100%) | Alcohol or substance abuse (20.5%) | Antipsychotics (67.5%) | 9.8y | 1; 2; 3.5 | 28.9% | DIS; IND; NEG; OSF; POS; PSB; VOF |
| Oh 2017 ^S104^ | 22-22 | 22.9 (5.6) | 52.5% | Psychotic disorder (100%) | unclear | Not reported | <2y | 1 | 45.0% | NEG; OSF; POS |
| Okin 1995 ^S105^ | 53-53 | 37.6 (14.2) | 41.5% | Schizophrenia (100%) | unclear | Community residential treatment (100%) | 11.5y | 7.5 | 0.0% | IND; OVC; PSB; VOF |
| Oribe 2015 ^S106,S107^ | 18-18 | 21.7 (4.6) | 27.8% | Schizophrenia (100%) | unclear | Atypical antipsychotics (72.2%); mood stabilizers (5.6%); antidepressants (33.3%); anxiolytics (16.7%) | 1.2y | 1 | 0.0% | NEG; OSF; POS |
| Ortega 2021 ^S108^ | 61-61 | 24.1 (4.3) | 24.6% | First episode Psychosis (100%) | unclear | Antipsychotics (100%) | <5y | 1 | unclear | NEG; OSF; POS; PRC |
| Ozawa 2019 ^S109^ | 35-35 | 63.9 (7.9) | 34.3% | Schizophrenia (100%) | unclear | Antipsychotics (100%) | 35.7y | 1 | 25.5% | DEP; NEG; OSF; POS |
| Prouteau 2005 ^S110^ | 55-55 | 34.3 (12.0) | 34.6% | Schizophrenia (70.9%); Schizoaffective disorder (23.6%); Schizophreniform disorder (3.6%); Unspecified psychotic disorder (1.8%) | unclear | Antipsychotics (100%); Integrated Psychological Treatment (100%); antidepressants (23.6%); Mood stabilizers (20.0%); Antiparkinsonians (40.0%); Anxiolytics (3.6%) | 7.4y | 0.5; 1; 1.3 | 0.0% | IND; OSF; PRC; PSB |
| Putnam 2000 ^S111-S117^ | 317-317 | 67.7 (11.5) | 37.5% | Schizophrenia (100%) | unclear | Antipsychotics (99.7%) | 41.2y | 1; 1.2; 2.1; 4; 6 | 31.2% | IND; OSF; OVC; POS; VEM |
| Rodríguéz-Sánchez 2008 ^S118-S121^ | 549-549 | 29.1 (8.9) | 42.0% | Schizophrenia (60.0%); schizophreniform disorder (46.7%); psychosis NOS (3.3%); Brief psychotic disorder (6%) | unclear | Antipsychotics (100%); anticholinergics (6.5%); hypnotics (15.5%); benzodiazepines (52.9%); PAFIP integrated treatment (100%) | 2.3y | 0.1; 1; 3 | 27.5% | DEP; DIS; EXF; NEG; OSF; OVC; POS; PRS; VEM |
| Rossi 2009 ^S122^ | 326-326 | 44.2 (11.4) | 38.0% | schizophrenia (74.9%); schizoaffective disorder (25.1%) | unclear | Risperidone long-acting injectable (100%); benzodiazepines (45.56%) | 17.3y | 1 | 30.0% | NEG; OSF; POS |
| Rowland 2018 ^S123^ | 290-257 | 21.3 (4.9) | 25.8% | Schizophrenia (84.5%); Delusional disorder (15.5%) | Substance use (70.0%) | Early Intervention Services (100%) | 0.0y | 1 | 17.9% | DEP; NEG; OSF; POS; PRC |
| Rund 2007 ^S124,S125^ | 300-280 | 28.2 (9.0) | 42.3% | Schizophrenia (52.3%); schizophreniform disorder (4.5%); schizoaffective disorder (10.8%); Delusional disorder (5.4%); psychosis NOS (27.0%) | affective disorder (18.9%) | TIPS treatment program: antipsychotic medication, individual psychosocial treatment and psychoeducational family work; psychotherapy (100%) | 0.2y | 0.3; 1 | 38.9% | DEP; EXF; NEG; OSF; POS; PRS; VEM |
| Ryu 2006 ^S126-S129^ | 78-78 | 54.6 (7.2) | 34.6% | Schizophrenia (100%) | unclear | Optimal Treatment Project (OTP); Integrated community treatment; Antipsychotics | 31.5y | 1; 2; 3; 4; 5; 6; 12; 15 | 28.2% | EXF; IND; NEG; OSF; OVC; POS; PRC; PRS; PSB; QOL; VEM |
| Salyers 2014 ^S130,S131^ | 118-118 | 47.7 (8.9) | 20.7% | Schizophrenia (46.6%); schizoaffective disorder (55.2%) | unclear | Psychosocial treatment | Unclear | 0.8; 1.5 | 40.7% | NEG; POS; PRC; QOL |
| Schmidt 2017 ^S132^ | 120-120 | 34.3 (11.2) | 43.3% | Schizophrenia (55.0%); Schizoaffective disorder (19.2%); Schizophreniform disorder (14.2%); Delusional disorder (5.8%); Psychotic disorder NOS (5.8%) | Substance use disorder (41.7%) | Quetiapine (100%); ACT integrated care treatment (100%) | <5y | 1 | 15.8% | DEP; NEG; OSF; PRC; QOL |
| Scottish Schizophrenia Research group 1988 ^S133-S135^ | 111-111 | 30.6 (5.8) | 53.1% | Schizophrenia (100%) | unclear | Antipsychotics (100%) | 0.2y | 1; 2; 5 | 16.3% | EXF; IND; NEG; OVC; PSB; VEM; VOF |
| She 2017 ^S136^ | 170-169 | 32.4 (8.3) | 37.1% | Schizophrenia (100%) | unclear | Integrated group treatment (50.6%); antipsychotics (100%) | 7.2y | 0.3; 0.5; 1 | 36.5% | IND; NEG; POS; PSB |
| Siegel 2006 ^S137^ | 98-92 | 28.6 (7.4) | 40.8% | Schizophrenia (100%) | unclear | Antipsychotics (85.9%) | 6.1y | 3 | 52.9% | DEP; NEG; OSF; POS |
| Sikira 2021 ^S138^ | 65-50 | 43.0 (11.1) | 53.9% | Schizophrenia spectrum disorder (100%) | unclear | Volunteer befriending intervention (50.8%); Antipsychotics (95.4%) | Unclear | 0.5; 1 | 23.1% | OSF; QOL |
| Smith 2002 ^S139^ | 46-45 | 37.0 (9.0) | 41.3% | Schizophrenia (60.9%); schizoaffective disorder (39.1%) | unclear | Outpatient treatment program; antipsychotics | 19.0y | 0.3; 0.5; 0.8; 1 | 37.5% | DIS; EXF; NEG; POS; PSB; VEM |
| Sommer 2021 ^S140^ | 113-70 | 26.3 (6.4) | 26.2% | Schizophrenia (100%) | unclear | Simvastatin (51.3%) | 1.3y | 0.3; 0.5; 0.8; 1; 2 | 58.8% | DEP; EXF; NEG; OSF; OVC; POS; VEM |
| Stouten 2014 ^S141,S142^ | 162-162 | 27.8 (8.3) | 27.5% | Schizophrenia (52.9%); Brief psychotic disorder (5.9%); Delusional disorder (5.2%); Shared psychotic disorder (1.3%); Psychotic disorder NOS (36.6%) | unclear | Not reported | <5y | 1 | 0.0% | IND; NEG; OSF; POS; PSB; VOF |
| Sweeney 1991 ^S143^ | 39-39 | 28.6 (8.6) | 38.5% | Schizophrenia (74.4%); schizophreniform disorder (10.3%); schizoaffective disorder (15.4%) | unclear | Antipsychotics (100%) | 6.6y | 0.3; 1; 1.3; 1.5 | 0.0% | EXF; NEG; POS; PRS; VEM |
| Tabáres-Seisdesos 2005 ^S144,S145^ | 47-47 | 33.4 (8.2) | 21.3% | Schizophrenia (100%) | unclear | Antipsychotics (100%); antidepressants (12.8%); benzodiazepines (31.9%); psychosocial rehabilitation (19.2%) | 8.7y | 1; 3 | 9.6% | DEP; EXF; IND; NEG; OSF; OVC; POS; PRS; PSB; VEM |
| Tabo 2017 ^S146^ | 120-120 | 40.9 (10.9) | 28.3% | Schizophrenia (100%) | unclear | Not reported | 16.3y | 1 | unclear | NEG; POS; QOL |
| Torgalsbøen 2015 ^S147,S148^ | 25-25 | 21.0 (2.6) | 39.3% | Schizophrenia (75.0%); schizoaffective disorder (21.4%); psychotic disorder NOS (3.6%) | Substance abuse (3.6%) | Psychotherapy (71.4%); group therapy (7.1%); psychoeducation (64.3%) | <0.5y | 2 | 10.7% | EXF; OVC; POS; VEM |
| Üçok 2011 ^S149-S151^ | 115-105 | 21.1 (4.8) | 43.3% | Schizophrenia (100%) | Alcohol and/or substance use (16.5%) | antipsychotics (63.0%) | 1.2y | 0.3; 1; 2; 3; 4 | 25.6% | NEG; OSF; POS; VOF |
| Usui 2022 ^S152^ | 59-59 | 22.6 (5.2) | 37.0% | Schizophrenia (74.1%); Schizophreniform disorder (11.1%); Delusional disorder (3.7%); Psychotic disorder NOS (11.1%) | unclear | Antipsychotics (100%) | <5y | 2 | 54.2% | DIS; NEG; POS; PRC |
| Veerman 2016 ^S153^ | 25-25 | 42.0 (10.4) | 24.0% | Schizophrenia (100%) | Alcohol use (20.0%); nicotine use (56.0%); cocaine use (12.0%) | Clozapine (100%); Psychotherapy (8%) | 19.6y | 1 | 19.4% | DEP; EXF; NEG; POS; QOL; VEM |
| Veijola 2014 ^S154^ | 33-33 | 34.0 (0.6) | 42.4% | Schizophrenia (100%) | unclear | Antipsychotics (100%) | 11.1y | 9 | 45.9% | OSF; OVC |
| Whitehorn 2002 ^S155^ | 103-56 | 21.9 (5.7) | 33.1% | Schizophrenia spectrum disorder (100%) | unclear | Second generation antipsychotic use; multidisciplinary treatment; psychoeducation | <2y | 0.5; 1 | 52.4% | DIS; NEG; OSF; POS |
| Wilson-d'Almeida 2013 ^S156^ | 306-306 | 41.1 (10.1) | 30.1% | Schizophrenia (100%) | unclear | Antipsychotics (100%) | Unclear | 0.5; 1 | 12.3% | NEG; POS; PRC |
| Wittorf 2004 ^S157^ | 11-11 | 31.9 (10.9) | 66.7% | schizophrenia (93.3%); schizoaffective disorder (6.7%) | unclear | Antipsychotics (100%) | 6.1y | 1.1 | 60.5% | DIS; EXF; NEG; POS |
| Wittorf 2008 ^S158^ | 96-96 | 33.9 (9.7) | 49.0% | schizophrenia (88.5%); schizoaffective disorder (11.5%) | Personality disorder (33.3%) | Cognitive-behaviorally oriented group treatment (51.0%); Antipsychotics (100%) | 6.1y | 1 | 36.0% | OSF; VEM |
| Wojtalik 2022 ^S159^ | 58-44 | 24.8 (5.2) | 25.5% | Schizophrenia (80.4%); Other psychotic disorder (19.6%) | Substance use disorder (49.0%) | Antipsychotics (100%) | 3.7y | 0.8; 1.5 | 52.0% | DEP; EXF; IND; NEG; OSF; OVC; POS; PRS; PSB; VEM; VOF |
| Wunderink 2009 ^S160^ | 125-125 | 26.4 (6.4) | 31.2% | schizophrenia (45.6%); other nonaffective psychosis (54.4%) | canabis dependence (24.0%) | Antipsychotics (100%) | 0.7y | 0.5; 1.3; 2 | 14.4% | NEG; POS; PSB; QOL |
| Xie 2005 ^S161,S162^ | 152-152 | 32.4 (7.2) | 22.4% | Schizophrenia (70.4%); schizoaffective disorder (29.6%) | substance use disorder (100%); alcohol use disorder (81.6%); cannabis use disorder (44.7%); cocaine use disorder (15.1%); bipolar disorder (100%) | Dual disorder treatment (100.0%) | 12.0y | 0.5; 1; 1.5; 2; 2.5; 3; 4; 5; 6; 7; 8; 9; 10 | 23.1% | DIS; IND; NEG; POS; PRC; PSB; QOL; VOF |
| Xu 2014 ^S163,S164^ | 60-60 | 25.3 (10.4) | 45.0% | Schizophrenia (51.7%); schizophreniform disorder (20.0%); psychosis NOS (21.7%); schizoaffective disorder (6.7%) | unclear | Antipsychotics (95.0%); Anticholinergics (18.3%) | 0.0y | 1; 3 | 23.1% | DIS; EXF; NEG; OVC; POS |
| Zäske 2018 ^S165^ | 48-24 | 32.0 (10.1) | 45.8% | Schizophrenia (100%) | unclear | Antipsychotics (100%) | <2y | 1 | 50.0% | DEP; OSF; PRC; PSB; QOL |
| Zhu 2022 ^S166^ | 270-181 | 46.8 (8.6) | 36.3% | Schizophrenia (100%) | unclear | CRT (80.0%); antipsychotics (100%) | 23.0y | 1.5 | 33.0% | NEG; OSF; OVC; POS |

* References of all included studies are presented in Supplementary Materials 12

** DEP = Depressive symptoms; DIS=disorganization; EXF=Executive functioning; IND=Independence; NEG=Negative symptoms; OSF=Overall social functioning; OVC=Overall cognition; POS=positive symptoms; PRC=Personal recovery; PRS=Processing speed; PSB=Prosocial behavior; QOL=Subjective quality of life; VEM=Verbal memory; VOF=Vocational functioning

# **Supplementary material 6**. Overall meta-analysis of outcome domains per DOI subgroup*

* DEP = Depressive symptoms; DIS=disorganization; EXF=Executive functioning; IND=Independence; NEG=Negative symptoms; OSF=Overall social functioning; OVC=Overall cognition; POS=positive symptoms; PRC=Personal recovery; PRS=Processing speed; PSB=Prosocial behavior; QOL=Subjective quality of life; VEM=Verbal memory; VOF=Vocational functioning

# **Supplementary materials 7.** Correlation matrix effect sizes of change combined outcome domains.

| **Overall outcomes** | | | | | | | | | | | |
| --- | --- | --- | --- | --- | --- | --- | --- | --- | --- | --- | --- |
|  |  | POS | NEG | DIS | DEP | PRC | QOL | VEM | EXF | PRS | OVC |
| OSF | r | **0.66** | **0.69** | **0.76** | **0.64** | 0.36 | 0.55 | **-0.57** | **0.81** | **0.96** | **0.74** |
|  | p | 0.00 | 0.00 | 0.01 | 0.00 | 0.13 | 0.05 | 0.04 | 0.00 | 0.00 | 0.00 |
|  | k | 51 | 53 | 12 | 27 | 20 | 13 | 13 | 12 | 11 | 13 |
| PSB | r | **0.73** | **0.68** | NA | NA | 0.40 | 0.63 | NA | NA | NA | NA |
|  | p | 0.00 | 0.00 | NA | NA | 0.18 | 0.05 | NA | NA | NA | NA |
|  | k | 23 | 25 | NA | NA | 13 | 10 | NA | NA | NA | NA |
| IND | r | 0.46 | **0.50** | NA | NA | -0.26 | NA | NA | NA | NA | NA |
|  | p | 0.06 | 0.03 | NA | NA | 0.44 | NA | NA | NA | NA | NA |
|  | k | 18 | 20 | NA | NA | 11 | NA | NA | NA | NA | NA |
| VOF | r | 0.01 | **0.48** | NA | NA | 0.56 | NA | NA | NA | NA | NA |
|  | p | 0.98 | 0.04 | NA | NA | 0.12 | NA | NA | NA | NA | NA |
|  | k | 17 | 19 | NA | NA | 9 | NA | NA | NA | NA | NA |
| POS | r | X | X | X | X | **0.64** | **0.64** | -0.02 | **0.47** | 0.22 | 0.01 |
|  | p | X | X | X | X | 0.00 | 0.00 | 0.92 | 0.02 | 0.41 | 0.97 |
|  | k | X | X | X | X | 23 | 19 | 23 | 25 | 16 | 20 |
| NEG | r | X | X | X | X | 0.40 | **0.62** | -0.19 | **0.53** | **0.70** | **0.56** |
|  | p | X | X | X | X | 0.06 | 0.00 | 0.40 | 0.01 | 0.00 | 0.01 |
|  | k | X | X | X | X | 23 | 20 | 23 | 25 | 15 | 20 |
| DIS | r | X | X | X | X | NA | NA | NA | NA | NA | NA |
|  | p | X | X | X | X | NA | NA | NA | NA | NA | NA |
|  | k | X | X | X | X | NA | NA | NA | NA | NA | NA |
| DEP | r | X | X | X | X | 0.50 | NA | **-0.74** | 0.48 | **0.65** | NA |
|  | p | X | X | X | X | 0.10 | NA | 0.00 | 0.08 | 0.03 | NA |
|  | k | X | X | X | X | 12 | NA | 14 | 14 | 11 | NA |
| PRC | r | X | X | X | X | X | X | NA | NA | NA | NA |
|  | p | X | X | X | X | X | X | NA | NA | NA | NA |
|  | k | X | X | X | X | X | X | NA | NA | NA | NA |
| QOL | r | X | X | X | X | X | X | NA | NA | NA | NA |
|  | p | X | X | X | X | X | X | NA | NA | NA | NA |
|  | k | X | X | X | X | X | X | NA | NA | NA | NA |
| **DOI <5 years subgroup** | | | | | | | | | | | |
|  |  | POS | NEG | DIS | DEP | PRC | QOL | VEM | EXF | PRS | OVC |
| OSF | r | **0.50** | **0.64** | 0.56 | 0.42 | -0.02 | 0.48 | -0.59 | **0.98** | **0.98** | 0.89 |
|  | p | 0.01 | 0.00 | 0.45 | 0.16 | 0.97 | 0.41 | 0.29 | 0.00 | 0.01 | 0.11 |
|  | k | 26 | 27 | 4 | 13 | 7 | 5 | 5 | 5 | 5 | 4 |
| PSB | r | 0.14 | 0.48 | NA | NA | 0.48 | **0.97** | NA | NA | NA | NA |
|  | p | 0.70 | 0.14 | NA | NA | 0.52 | 0.03 | NA | NA | NA | NA |
|  | k | 10 | 11 | NA | NA | 4 | 4 | NA | NA | NA | NA |
| IND | r | 0.19 | **0.78** | NA | NA | NA | NA | NA | NA | NA | NA |
|  | p | 0.69 | 0.02 | NA | NA | NA | NA | NA | NA | NA | NA |
|  | k | 7 | 8 | NA | NA | NA | NA | NA | NA | NA | NA |
| VOF | r | -0.01 | 0.45 | NA | NA | 0.41 | NA | NA | NA | NA | NA |
|  | p | 0.98 | 0.13 | NA | NA | 0.59 | NA | NA | NA | NA | NA |
|  | k | 12 | 13 | NA | NA | 4 | NA | NA | NA | NA | NA |
| POS | r | X | X | X | X | **0.90** | **0.83** | 0.38 | 0.34 | 0.02 | -0.42 |
|  | p | X | X | X | X | 0.00 | 0.04 | 0.36 | 0.37 | 0.97 | 0.40 |
|  | k | X | X | X | X | 8 | 6 | 8 | 9 | 6 | 6 |
| NEG | r | X | X | X | X | 0.60 | **0.87** | -0.60 | **0.73** | **0.93** | 0.63 |
|  | p | X | X | X | X | 0.09 | 0.03 | 0.12 | 0.03 | 0.02 | 0.13 |
|  | k | X | X | X | X | 9 | 6 | 8 | 9 | 5 | 7 |
| DIS | r | X | X | X | X | NA | NA | NA | NA | NA | NA |
|  | p | X | X | X | X | NA | NA | NA | NA | NA | NA |
|  | k | X | X | X | X | NA | NA | NA | NA | NA | NA |
| DEP | r | X | X | X | X | 0.42 | NA | -0.78 | 0.60 | 0.58 | NA |
|  | p | X | X | X | X | 0.41 | NA | 0.07 | 0.21 | 0.30 | NA |
|  | k | X | X | X | X | 6 | NA | 6 | 6 | 5 | NA |
| PRC | r | X | X | X | X | X | X | NA | NA | NA | NA |
|  | p | X | X | X | X | X | X | NA | NA | NA | NA |
|  | k | X | X | X | X | X | X | NA | NA | NA | NA |
| QOL | r | X | X | X | X | X | X | NA | NA | NA | NA |
|  | p | X | X | X | X | X | X | NA | NA | NA | NA |
|  | k | X | X | X | X | X | X | NA | NA | NA | NA |
| **DOI 5-10 years subgroup** | | | | | | | | | | | |
|  |  | POS | NEG | DIS | DEP | PRC | QOL | VEM | EXF | PRS | OVC |
| OSF | r | 0.73 | 0.70 | NA | 0.71 | NA | NA | NA | NA | NA | NA |
|  | p | 0.10 | 0.13 | NA | 0.29 | NA | NA | NA | NA | NA | NA |
|  | k | 6 | 6 | NA | 4 | NA | NA | NA | NA | NA | NA |
| PSB | r | 0.92 | 0.95 | NA | NA | NA | NA | NA | NA | NA | NA |
|  | p | 0.26 | 0.20 | NA | NA | NA | NA | NA | NA | NA | NA |
|  | k | 3 | 3 | NA | NA | NA | NA | NA | NA | NA | NA |
| IND | r | 0.56 | 0.64 | NA | NA | NA | NA | NA | NA | NA | NA |
|  | p | 0.62 | 0.56 | NA | NA | NA | NA | NA | NA | NA | NA |
|  | k | 3 | 3 | NA | NA | NA | NA | NA | NA | NA | NA |
| VOF | r | NA | NA | NA | NA | NA | NA | NA | NA | NA | NA |
|  | p | NA | NA | NA | NA | NA | NA | NA | NA | NA | NA |
|  | k | NA | NA | NA | NA | NA | NA | NA | NA | NA | NA |
| POS | r | X | X | X | X | 0.94 | NA | 0.10 | 0.53 | 0.44 | NA |
|  | p | X | X | X | X | 0.22 | NA | 0.87 | 0.36 | 0.56 | NA |
|  | k | X | X | X | X | 3 | NA | 5 | 5 | 4 | NA |
| NEG | r | X | X | X | X | NA | NA | 0.66 | 0.78 | 0.43 | NA |
|  | p | X | X | X | X | NA | NA | 0.22 | 0.12 | 0.57 | NA |
|  | k | X | X | X | X | NA | NA | 5 | 5 | 4 | NA |
| DIS | r | X | X | X | X | NA | NA | NA | NA | NA | NA |
|  | p | X | X | X | X | NA | NA | NA | NA | NA | NA |
|  | k | X | X | X | X | NA | NA | NA | NA | NA | NA |
| DEP | r | X | X | X | X | NA | NA | NA | NA | NA | NA |
|  | p | X | X | X | X | NA | NA | NA | NA | NA | NA |
|  | k | X | X | X | X | NA | NA | NA | NA | NA | NA |
| PRC | r | X | X | X | X | X | X | NA | NA | NA | NA |
|  | p | X | X | X | X | X | X | NA | NA | NA | NA |
|  | k | X | X | X | X | X | X | NA | NA | NA | NA |
| QOL | r | X | X | X | X | X | X | NA | NA | NA | NA |
|  | p | X | X | X | X | X | X | NA | NA | NA | NA |
|  | k | X | X | X | X | X | X | NA | NA | NA | NA |
| **DOI >10 years subgroup** | | | | | | | | | | | |
|  |  | POS | NEG | DIS | DEP | PRC | QOL | VEM | EXF | PRS | OVC |
| OSF | r | **0.91** | **0.88** | **0.99** | **0.96** | 0.11 | 0.59 | 0.43 | 0.80 | **0.99** | -0.46 |
|  | p | 0.00 | 0.00 | 0.01 | 0.00 | 0.78 | 0.29 | 0.40 | 0.11 | 0.01 | 0.25 |
|  | k | 15 | 16 | 4 | 8 | 9 | 5 | 6 | 5 | 4 | 8 |
| PSB | r | **0.91** | **0.80** | NA | NA | 0.07 | 0.48 | NA | NA | NA | NA |
|  | p | 0.00 | 0.01 | NA | NA | 0.88 | 0.34 | NA | NA | NA | NA |
|  | k | 8 | 9 | NA | NA | 7 | 6 | NA | NA | NA | NA |
| IND | r | 0.51 | 0.43 | NA | NA | -0.33 | NA | NA | NA | NA | NA |
|  | p | 0.24 | 0.29 | NA | NA | 0.43 | NA | NA | NA | NA | NA |
|  | k | 7 | 8 | NA | NA | 8 | NA | NA | NA | NA | NA |
| VOF | r | NA | 0.77 | NA | NA | 0.96 | NA | NA | NA | NA | NA |
|  | p | NA | 0.44 | NA | NA | 0.18 | NA | NA | NA | NA | NA |
|  | k | NA | 3 | NA | NA | 3 | NA | NA | NA | NA | NA |
| POS | r | X | X | X | X | **0.87** | **0.64** | -0.37 | 0.52 | -0.04 | 0.02 |
|  | p | X | X | X | X | 0.01 | 0.05 | 0.30 | 0.10 | 0.94 | 0.97 |
|  | k | X | X | X | X | 7 | 10 | 10 | 11 | 6 | 10 |
| NEG | r | X | X | X | X | -0.30 | 0.58 | -0.14 | -0.13 | -0.16 | -0.05 |
|  | p | X | X | X | X | 0.51 | 0.06 | 0.69 | 0.70 | 0.76 | 0.90 |
|  | k | X | X | X | X | 7 | 11 | 10 | 11 | 6 | 10 |
| DIS | r | X | X | X | X | NA | NA | NA | NA | NA | NA |
|  | p | X | X | X | X | NA | NA | NA | NA | NA | NA |
|  | k | X | X | X | X | NA | NA | NA | NA | NA | NA |
| DEP | r | X | X | X | X | 0.74 | NA | -0.77 | -0.09 | 0.52 | NA |
|  | p | X | X | X | X | 0.15 | NA | 0.08 | 0.86 | 0.48 | NA |
|  | k | X | X | X | X | 5 | NA | 6 | 6 | 4 | NA |
| PRC | r | X | X | X | X | X | X | NA | NA | NA | NA |
|  | p | X | X | X | X | X | X | NA | NA | NA | NA |
|  | k | X | X | X | X | X | X | NA | NA | NA | NA |
| QOL | r | X | X | X | X | X | X | NA | NA | NA | NA |
|  | p | X | X | X | X | X | X | NA | NA | NA | NA |
|  | k | X | X | X | X | X | X | NA | NA | NA | NA |

* NA = not applicable because less than 10 studies reported on this combination of outcomes. X = Correlations are from the same outcome domain, or already presented elsewhere in the table. DEP=depression; DIS=disorganization; EXF=Executive functioning; IND=Independence; NEG=Negative symptoms; OSF=Overall social functioning; OVC=Overall cognition; POS=positive symptoms; PRC=Personal recovery; PRS=Processing speed; PSB=Prosocial behavior; QOL=Subjective quality of life; VEM=Verbal memory; VOF=Vocational functioning

# **Supplementary material 8.** Analysis of moderating effects subgroups of changes in combination of outcome domains.

| **Negative symptoms and overall social functioning** | | | | | | | | | | | | | | | | |
| --- | --- | --- | --- | --- | --- | --- | --- | --- | --- | --- | --- | --- | --- | --- | --- | --- |
| **Subgroup:** | 1. both moderate-high improvement | | | 2. Only one moderate- high improvement | | | 3. Both marginal-no improvement | | | 4. No improvement or deterioration | | | **ANOVA** | | | |
| **Demographic variables** | M | SD | N | M | SD | N | M | SD | N | M | SD | N | F | Df | p | Subgroup differences |
| Age* | 28.2 | 4.9 | 12 | 29.3 | 10.0 | 12 | 29.2 | 7.5 | 3 | 37.3 | 13.1 | 25 | 2.7 | 3 | 0.06 | 1<4 |
| Age at onset | 23.3 | 3.3 | 10 | 23.4 | 3.6 | 10 | 23.5 | 1.7 | 3 | 24.4 | 3.3 | 19 | 0.3 | 3 | 0.79 |  |
| Ethnicity: % Caucasian or born in country of residence | 53.7 | 15.0 | 3 | 76.7 | 20.7 | 5 |  |  |  | 64.0 | 16.0 | 9 | 1.8 | 2 | 0.21 |  |
| Female gender (%) | 38.6 | 10.1 | 12 | 37.2 | 9.0 | 12 | 34.8 | 8.2 | 3 | 35.3 | 10.9 | 24 | 0.3 | 3 | 0.80 |  |
| Marital status: % not married/in relationship | 84.1 | 10.4 | 3 | 78.7 | 1.1 | 2 | 41.1 |  | 1 | 18.9 | 13.8 | 5 | 0.20 | 3 | 0.90 |  |
|  | n | % | N | n | % | N | n | % | N | n | % | N | χ^2^ | df | p | Subgroup differences |
| Education level (high level) | 6 | 66.7% | 9 | 5 | 62.5% | 8 |  |  |  | 6 | 43.8% | 16 | 1.5 | 2 | 0.48 |  |
| **Clinical variables** | M | SD | N | M | SD | N | M | SD | N | M | SD | N | F | Df | p | Subgroup differences |
| Duration of untreated psychosis in weeks* | 16.8 | 6.3 | 5 | 22.1 | 13.6 | 9 | 41.1 |  | 1 | 18.9 | 13.8 | 5 | 1.2 | 3 | 0.35 | 1<3 |
| Duration of illness (DOI) in years | 4.3 | 3.0 | 11 | 6.0 | 9.5 | 12 | 5.7 | 7.0 | 3 | 11.4 | 12.2 | 23 | 1.6 | 3 | 0.20 |  |
| Schizophrenia diagnosis (%) | 78.2 | 21.1 | 9 | 77.2 | 16.2 | 10 | 100.0 | 0.0 | 2 | 84.6 | 22.1 | 19 | 0.92 | 3 | 0.44 |  |
|  | n | % | N | n | % | N | n | % | N | n | % | N | χ^2^ | df | p | Subgroup differences |
| Antipsychotic use by all participants | 5 | 41.7% | 12 | 9 | 75.0% | 12 | 3 | 100.0% | 3 | 16 | 64.0% | 25 | 4.9 | 3 | 0.18 |  |
| Combined treatment of psychosocial and pharmacological therapy provided | 6 | 60.0% | 10 | 3 | 42.9% | 7 | 1 | 100.0% | 1 | 10 | 76.9% | 13 | 2.9 | 3 | 0.40 |  |
| Treatment provided focused on targeted outcomes. | 3 | 42.9% | 7 | 3 | 42.9% | 7 | 1 | 100.0% | 1 | 13 | 72.2% | 18 | 3.5 | 3 | 0.32 |  |
| **Symptoms** | M | SD | N | M | SD | N | M | SD | N | M | SD | N | F | Df | p | Subgroup differences |
| Depressive symptoms | 41.0 | 23.3 | 3 | 54.2 | 22.8 | 5 | 80.7 | 26.7 | 2 | 56.9 | 19.1 | 14 | 1.5 | 3 | 0.25 |  |
| Disorganization symptoms | 37.7 | 31.8 | 6 | 63.7 |  | 1 | 10.6 |  | 1 | 60.5 | 38.0 | 6 | 0.9 | 3 | 0.49 |  |
| Negative symptoms | 54.1 | 26.9 | 12 | 51.5 | 29.1 | 11 | 56.5 | 53.8 | 2 | 57.9 | 25.0 | 24 | 0.1 | 3 | 0.93 |  |
| Overall symptoms | 55.5 | 27.9 | 8 | 52.9 | 21.1 | 8 | 61.8 |  | 1 | 56.8 | 21.7 | 16 | 0.1 | 3 | 0.97 |  |
| Positive symptoms | 47.2 | 29.2 | 11 | 64.0 | 24.2 | 11 | 82.6 | 9.6 | 2 | 66.1 | 25.4 | 24 | 1.9 | 3 | 0.15 |  |
| **Functioning** | M | SD | N | M | SD | N | M | SD | N | M | SD | N | F | Df | p | Subgroup differences |
| Independence | 65.4 | 21.6 | 2 | 41.9 | 43.0 | 2 |  |  |  | 67.3 | 27.2 | 6 | 0.6 | 2 | 0.58 |  |
| Overall social functioning | 48.5 | 20.0 | 11 | 43.7 | 16.0 | 12 | 50.4 | 8.1 | 2 | 54.8 | 18.3 | 24 | 1.1 | 3 | 0.37 |  |
| Prosocial behavior | 45.5 | 14.0 | 4 | 48.9 | 2.9 | 2 |  |  |  | 58.3 | 16.8 | 7 | 1.0 | 2 | 0.40 |  |
| Vocational functioning | 40.1 | 18.9 | 4 | 52.8 | 35.4 | 4 |  |  |  | 39.5 | 16.8 | 11 | 0.6 | 2 | 0.58 |  |
| **Personal recovery and QOL** | M | SD | N | M | SD | N | M | SD | N | M | SD | N | F | Df | p | Subgroup differences |
| Personal recovery | 30.8 |  | 1 | 40.3 | 8.0 | 4 | 18.4 |  | 1 | 55.6 | 22.9 | 6 | 1.6 | 3 | 0.27 |  |
| Subjective quality of life | 46.4 | 24.4 | 2 | 57.7 | 5.7 | 2 | 30.5 | 18.9 | 2 | 48.3 | 15.9 | 7 | 0.9 | 3 | 0.46 |  |
| **Cognition** | M | SD | N | M | SD | N | M | SD | N | M | SD | N | F | Df | p | Subgroup differences |
| Executive functioning | 78.8 |  | 1 | 59.9 | 50.4 | 2 |  |  |  | 37.7 | 44.7 | 7 | 0.5 | 2 | 0.64 |  |
| Overall cognition | 54.8 | 31.7 | 6 | 48.6 | 19.5 | 5 |  |  |  | 32.8 | 24.9 | 9 | 1.4 | 2 | 0.27 |  |
| Processing speed |  |  |  |  |  |  |  |  |  |  |  |  |  |  |  |  |
| Verbal memory | 65.5 |  | 1 | 57.6 | 45.0 | 2 |  |  |  | 53.4 | 28.6 | 7 | 0.1 | 2 | 0.93 |  |
| **Study characteristics** | M | SD | N | M | SD | N | M | SD | N | M | SD | N | F | Df | p | Subgroup differences |
| Attrition rate* | 40.1 | 17.4 | 12 | 27.5 | 14.0 | 12 | 31.9 | 23.4 | 3 | 24.0 | 15.9 | 22 | 2.6 | 3 | 0.06 | 1>4 |
|  | n | % | N | n | % | N | n | % | N | n | % | N | χ^2^ | df | p | Subgroup differences |
| DOI subgroup overlap (yes vs no) | 3 | 50.0% | 6 | 3 | 37.5% | 8 | 1 | 100.0% | 1 | 10 | 58.8% | 17 | 1.9 | 3 | 0.59 |  |
| Publication year <10 years | 9 | 75.0% | 12 | 5 | 41.7% | 12 | 2 | 66.7% | 3 | 17 | 68.0% | 25 | 3.4 | 3 | 0.34 |  |
| Study design: clinical trial | 3 | 25.0% | 12 | 5 | 45.5% | 11 | 1 | 50.0% | 2 | 11 | 44.0% | 25 | 1.5 | 3 | 0.68 |  |
| **Quality assessment** | n | % | N | n | % | N | n | % | N | n | % | N | χ^2^ | df | p | Subgroup differences |
| Study participation: Low ROB | 9 | 75.0% | 12 | 10 | 83.3% | 12 | 1 | 33.3% | 3 | 10 | 40.0% | 25 | 9.1 | 3 | 0.17 |  |
| Study attrition: Low ROB | 4 | 33.3% | 12 | 4 | 33.3% | 12 | 2 | 66.7% | 3 | 9 | 36.0% | 25 | 4.4 | 3 | 0.63 |  |
| Prognostic Factor Measurement: Low ROB | 3 | 25.0% | 12 | 6 | 50.0% | 12 | 0 | 0.0% | 3 | 7 | 28.0% | 25 | 5.4 | 3 | 0.80 |  |
| Outcome measurement: Low ROB | 10 | 83.3% | 12 | 10 | 83.3% | 12 | 3 | 100.0% | 3 | 20 | 80.0% | 25 | 1.7 | 3 | 0.95 |  |
| Study confounding: Low ROB | 5 | 41.7% | 12 | 8 | 66.7% | 12 | 3 | 100.0% | 3 | 9 | 36.0% | 25 | 8.4 | 3 | 0.21 |  |
| Statistical analysis and reporting: Low ROB | 8 | 66.7% | 12 | 12 | 100.0% | 12 | 3 | 100.0% | 3 | 20 | 80.0% | 25 | 6.8 | 3 | 0.34 |  |
| **Negative symptoms and Prosocial behavior** | | | | | | | | | | | | | | | | |
| **Subgroup:** | 1. both moderate-high improvement | | | 2. Only one moderate- high improvement | | | 3. Both marginal-no improvement | | | 4. No improvement or deterioration | | | **ANOVA** | | | |
| **Demographic variables** | M | SD | N | M | SD | N | M | SD | N | M | SD | N | F | Df | p | Subgroup differences |
| Age | 31.0 | 6.0 | 4 | 32.2 | 15.0 | 4 | 32.4 |  | 1 | 33.8 | 8.9 | 16 | 0.1 | 3 | 0.96 |  |
| Age at onset | 24.5 | 3.2 | 3 | 23.0 | 2.2 | 4 | 20.4 |  | 1 | 23.0 | 4.3 | 13 | 0.3 | 3 | 0.83 |  |
| Ethnicity: % Caucasian or born in country of residence | 57.0 | 19.6 | 2 | 37.9 |  | 1 | 96.7 |  | 1 | 59.9 | 27.4 | 6 | 0.9 | 3 | 0.50 |  |
| Female gender (%)* | 38.2 | 9.6 | 4 | 34.9 | 2.7 | 4 | 22.4 |  | 1 | 36.1 | 11.6 | 16 | 0.6 | 3 | 0.60 | 2>3 |
| Marital status: % not married/in relationship | 86.8 | 13.0 | 2 | 82.1 | 3.8 | 2 | 68.4 |  | 1 | 76.9 | 12.0 | 6 | 0.7 | 3 | 0.57 |  |
|  | n | % | N | n | % | N | n | % | N | n | % | N | χ^2^ | df | p | Subgroup differences |
| Education level (high level) | 3 | 100.0% | 3 | 1 | 50.0% | 2 | 1 | 100.0% | 1 | 4 | 33.3% | 12 | 5.3 | 3 | 0.15 |  |
| **Clinical variables** | M | SD | N | M | SD | N | M | SD | N | M | SD | N | F | Df | p | Subgroup differences |
| Duration of untreated psychosis in weeks | 8.7 |  | 1 | 18.6 | 12.9 | 4 |  |  |  | 20.7 | 13.4 | 5 | 0.3 | 2 | 0.72 |  |
| Duration of illness (DOI) in years | 7.4 | 3.8 | 3 | 13.7 | 14.7 | 4 | 12.0 |  | 1 | 9.0 | 8.1 | 15 | 0.4 | 3 | 0.78 |  |
| Schizophrenia diagnosis (%) | 85.1 | 19.1 | 4 | 66.0 | 24.3 | 4 | 70.4 |  | 1 | 76.4 | 23.6 | 14 | 0.5 | 3 | 0.70 |  |
|  | n | % | N | n | % | N | n | % | N | n | % | N | χ^2^ | df | p | Subgroup differences |
| Antipsychotic use by all participants* | 4 | 100.0% | 4 | 1 | 25.0% | 4 | 0 | 0.0% | 4 | 4 | 25.0% | 16 | 8.7 | 3 | 0.03 | 1>4 |
| Combined treatment of psychosocial and pharmacological therapy provided | 2 | 66.7% | 3 | 2 | 66.7% | 3 | 1 | 100.0% | 1 | 3 | 27.3% | 11 | 3.8 | 3 | 0.29 |  |
| Treatment provided focused on targeted outcomes. | 3 | 100.0% | 3 | 2 | 50.0% | 4 | 0 | 0.0% | 1 | 2 | 25.0% | 8 | 5.8 | 3 | 0.12 |  |
| **Symptoms** | M | SD | N | M | SD | N | M | SD | N | M | SD | N | F | Df | p | Subgroup differences |
| Depressive symptoms |  |  |  |  |  |  |  |  |  |  |  |  |  |  |  |  |
| Disorganization symptoms |  |  |  |  |  |  |  |  |  |  |  |  |  |  |  |  |
| Negative symptoms | 68.3 | 27.8 | 4 | 55.5 | 39.0 | 3 | 85.4 |  | 1 | 66.1 | 25.0 | 15 | 0.3 | 3 | 0.81 |  |
| Overall symptoms | 57.9 | 30.1 | 3 | 57.2 | 41.2 | 3 | 56.0 |  | 1 | 49.6 | 27.2 | 7 | 0.1 | 3 | 0.97 |  |
| Positive symptoms | 50.5 | 43.2 | 4 | 56.3 | 43.4 | 3 | 62.8 |  | 1 | 63.9 | 32.1 | 15 | 0.2 | 3 | 0.92 |  |
| **Functioning** | M | SD | N | M | SD | N | M | SD | N | M | SD | N | F | Df | p | Subgroup differences |
| Independence | 61.1 | 27.6 | 2 | 72.3 |  | 1 | 49.2 |  | 1 | 45.1 | 31.7 | 9 | 0.3 | 3 | 0.81 |  |
| Overall social functioning | 48.0 | 10.6 | 3 | 31.8 | 32.6 | 3 |  |  |  | 57.7 | 18.6 | 12 | 2.0 | 2 | 0.17 |  |
| Prosocial behavior | 41.7 | 19.1 | 4 | 55.3 | 11.9 | 2 | 42.7 |  | 1 | 54.0 | 15.9 | 11 | 0.7 | 3 | 0.58 |  |
| Vocational functioning* | 35.0 |  | 1 | 67.7 | 26.8 | 3 | 14.9 |  | 1 | 42.7 | 11.1 | 9 | 3.6 | 3 | 0.05 | 2>4 / 3<4 |
| **Personal recovery and QOL** | M | SD | N | M | SD | N | M | SD | N | M | SD | N | F | Df | p | Subgroup differences |
| Personal recovery |  |  |  |  |  |  |  |  |  |  |  |  |  |  |  |  |
| Subjective quality of life* | 63.7 |  | 1 | 63.1 | 11.4 | 3 | 87.5 |  | 1 | 57.4 | 10.3 | 6 | 2.3 | 3 | 0.16 | 3>4 |
| **Cognition** | M | SD | N | M | SD | N | M | SD | N | M | SD | N | F | Df | p | Subgroup differences |
| Executive functioning |  |  |  |  |  |  |  |  |  |  |  |  |  |  |  |  |
| Overall cognition |  |  |  |  |  |  |  |  |  |  |  |  |  |  |  |  |
| Processing speed |  |  |  |  |  |  |  |  |  |  |  |  |  |  |  |  |
| Verbal memory |  |  |  |  |  |  |  |  |  |  |  |  |  |  |  |  |
| **Study characteristics** | M | SD | N | M | SD | N | M | SD | N | M | SD | N | F | Df | p | Subgroup differences |
| Attrition rate | 37.3 | 23.8 | 4 | 32.1 | 25.5 | 4 | 23.1 |  | 1 | 22.5 | 13.1 | 15 | 0.9 | 3 | 0.45 |  |
|  | n | % | N | n | % | N | n | % | N | n | % | N | χ^2^ | df | p | Subgroup differences |
| DOI subgroup overlap | 1 | 100.0% | 1 | 1 | 25.0% | 4 | 1 | 100.0% | 1 | 4 | 50.0% | 8 | 3.0 | 3 | 0.39 |  |
| Publication year <10 years* | 4 | 100.0% | 4 | 2 | 50.0% | 4 | 0 | 0.0% | 1 | 5 | 31.3% | 16 | 7.0 | 3 | 0.07 | 1>4 |
| Study design: clinical trial | 3 | 75.0% | 4 | 3 | 75.0% | 4 | 1 | 100.0% | 1 | 4 | 25.0% | 16 | 6.7 | 3 | 0.08 |  |
| **Quality assessment** | n | % | N | n | % | N | n | % | N | n | % | N | χ^2^ | df | p | Subgroup differences |
| Study participation: Low ROB | 3 | 75.0% | 4 | 3 | 75.0% | 4 | 1 | 100.0% | 1 | 5 | 31.3% | 16 | 5.2 | 3 | 0.16 |  |
| Study attrition: Low ROB | 1 | 25.0% | 4 | 1 | 25.0% | 4 | 1 | 100.0% | 1 | 6 | 37.5% | 16 | 6.5 | 3 | 0.37 |  |
| Prognostic Factor Measurement: Low ROB | 1 | 25.0% | 4 | 2 | 50.0% | 4 | 0 | 0.0% | 1 | 7 | 43.8% | 16 | 8.3 | 3 | 0.50 |  |
| Outcome measurement: Low ROB | 3 | 75.0% | 4 | 4 | 100.0% | 4 | 1 | 100.0% | 1 | 13 | 81.3% | 16 | 3.2 | 3 | 0.78 |  |
| Study confounding: Low ROB | 1 | 25.0% | 4 | 2 | 50.0% | 4 | 0 | 0.0% | 1 | 9 | 56.3% | 16 | 3.6 | 3 | 0.73 |  |
| Statistical analysis and reporting: Low ROB | 4 | 100.0% | 4 | 4 | 100.0% | 4 | 1 | 100.0% | 1 | 13 | 81.3% | 16 | 1.9 | 3 | 0.93 |  |
| **Negative symptoms and Independence** | | | | | | | | | | | | | | | | |
| **Subgroup:** | 1. both moderate-high improvement | | | 2. Only one moderate- high improvement | | | 3. Both marginal-no improvement | | | 4. No improvement or deterioration | | | **ANOVA** | | | |
| **Demographic variables** | M | SD | N | M | SD | N | M | SD | N | M | SD | N | F | Df | p | Subgroup differences |
| Age | 31.9 | 7.0 | 3 | 47.4 | 10.2 | 2 | 32.4 |  | 1 | 35.8 | 12.9 | 14 | 0.7 | 3 | 0.55 |  |
| Age at onset | 24.5 | 3.2 | 3 | 23.6 | 0.6 | 2 | 20.4 |  | 1 | 23.9 | 4.4 | 11 | 0.3 | 3 | 0.85 |  |
| Ethnicity: % Caucasian or born in country of residence |  |  |  |  |  |  |  |  |  |  |  |  |  |  |  |  |
| Female gender (%) | 34.9 | 8.6 | 3 | 32.4 | 3.1 | 2 | 22.4 |  | 1 | 36.8 | 11.3 | 14 | 0.6 | 3 | 0.60 |  |
| Marital status: % not married/in relationship |  |  |  |  |  |  |  |  |  |  |  |  |  |  |  |  |
|  | n | % | N | n | % | N | n | % | N | n | % | N | χ^2^ | df | p | Subgroup differences |
| Education level (high level) | 2 | 100.0% | 2 | 0 | 0.0% | 2 | 1 | 100.0% | 1 | 5 | 41.7% | 12 | 5.3 | 3 | 0.15 |  |
| **Clinical variables** | M | SD | N | M | SD | N | M | SD | N | M | SD | N | F | Df | p | Subgroup differences |
| Duration of untreated psychosis in weeks |  |  |  |  |  |  |  |  |  |  |  |  |  |  |  |  |
| Duration of illness (DOI) in years | 7.4 | 3.8 | 3 | 23.9 | 10.8 | 2 | 12.0 |  | 1 | 10.0 | 12.2 | 13 | 1.0 | 3 | 0.42 |  |
| Schizophrenia diagnosis (%) | 93.5 | 11.3 | 3 | 100.0 | 0.0 | 2 | 70.4 |  | 1 | 76.7 | 22.9 | 12 | 1.2 | 3 | 0.36 |  |
|  | n | % | N | n | % | N | n | % | N | n | % | N | χ^2^ | df | p | Subgroup differences |
| Antipsychotic use by all participants | 3 | 100.0% | 3 | 0 | 0.0% | 2 | 0 | 0.0% | 1 | 4 | 28.6% | 14 | 7.4 | 3 | 0.06 |  |
| Combined treatment of psychosocial and pharmacological therapy provided | 1 | 50.0% | 2 | 1 | 50.0% | 2 | 1 | 100.0% | 1 | 2 | 22.2% | 9 | 2.9 | 3 | 0.41 |  |
| Treatment provided focused on targeted outcomes. | 2 | 100.0% | 2 | 0 | 0.0% | 1 | 0 | 0.0% | 1 | 3 | 37.5% | 8 | 4.3 | 3 | 0.23 |  |
| **Symptoms** | M | SD | N | M | SD | N | M | SD | N | M | SD | N | F | Df | p | Subgroup differences |
| Depressive symptoms |  |  |  |  |  |  |  |  |  |  |  |  |  |  |  |  |
| Disorganization symptoms |  |  |  |  |  |  |  |  |  |  |  |  |  |  |  |  |
| Negative symptoms | 60.9 | 28.9 | 3 | 62.1 | 17.1 | 2 | 85.4 |  | 1 | 60.5 | 28.1 | 13 | 0.3 | 3 | 0.86 |  |
| Overall symptoms | 41.3 | 12.3 | 2 | 77.9 | 15.0 | 2 | 56.0 |  | 1 | 50.1 | 27.6 | 7 | 0.8 | 3 | 0.51 |  |
| Positive symptoms | 38.5 | 44.1 | 3 | 51.8 | 61.8 | 2 | 62.8 |  | 1 | 64.0 | 31.4 | 12 | 0.4 | 3 | 0.74 |  |
| **Functioning** | M | SD | N | M | SD | N | M | SD | N | M | SD | N | F | Df | p | Subgroup differences |
| Independence | 61.1 | 27.6 | 2 | 72.3 |  | 1 | 49.2 |  | 1 | 49.7 | 33.2 | 10 | 0.2 | 3 | 0.90 |  |
| Overall social functioning* | 42.0 | 3.0 | 2 | 73.6 | 9.1 | 2 |  |  |  | 42.7 | 18.5 | 10 | 2.9 | 2 | 0.10 | 1<2 / 2>4 |
| Prosocial behavior | 44.0 | 22.6 | 3 | 46.9 |  | 1 | 42.7 |  | 1 | 56.6 | 12.8 | 8 | 0.7 | 3 | 0.60 |  |
| Vocational functioning |  |  |  | 62.2 | 39.3 | 2 | 14.9 |  | 1 | 48.0 | 15.7 | 8 | 1.8 | 2 | 0.22 |  |
| **Personal recovery and QOL** | M | SD | N | M | SD | N | M | SD | N | M | SD | N | F | Df | p | Subgroup differences |
| Personal recovery |  |  |  |  |  |  |  |  |  |  |  |  |  |  |  |  |
| Subjective quality of life |  |  |  |  |  |  |  |  |  |  |  |  |  |  |  |  |
| **Cognition** | M | SD | N | M | SD | N | M | SD | N | M | SD | N | F | Df | p | Subgroup differences |
| Executive functioning |  |  |  |  |  |  |  |  |  |  |  |  |  |  |  |  |
| Overall cognition |  |  |  |  |  |  |  |  |  |  |  |  |  |  |  |  |
| Processing speed |  |  |  |  |  |  |  |  |  |  |  |  |  |  |  |  |
| Verbal memory |  |  |  |  |  |  |  |  |  |  |  |  |  |  |  |  |
| **Study characteristics** | M | SD | N | M | SD | N | M | SD | N | M | SD | N | F | Df | p | Subgroup differences |
| Attrition rate* | 48.4 | 10.6 | 3 | 30.6 | 3.3 | 2 | 23.1 |  | 1 | 22.1 | 12.8 | 13 | 3.9 | 3 | 0.03 | 1>4 |
|  | n | % | N | n | % | N | n | % | N | n | % | N | χ^2^ | df | p | Subgroup differences |
| DOI subgroup overlap (yes vs no) | 1 | 100.0% | 1 | 0 | 0.0% | 1 | 1 | 100.0% | 1 | 5 | 55.6% | 9 | 2.9 | 3 | 0.41 |  |
| Publication year <10 years | 3 | 100.0% | 3 | 1 | 50.0% | 2 | 0 | 0.0% | 1 | 5 | 35.7% | 14 | 5.0 | 3 | 0.17 |  |
| Study design: clinical trial | 3 | 100.0% | 3 | 0 | 0.0% | 2 | 1 | 100.0% | 1 | 5 | 35.7% | 14 | 7.0 | 3 | 0.07 |  |
| **Quality assessment** | n | % | N | n | % | N | n | % | N | n | % | N | χ^2^ | df | p | Subgroup differences |
| Study participation: Low ROB | 2 | 66.7% | 3 | 1 | 50.0% | 2 | 1 | 100.0% | 1 | 5 | 35.7% | 14 | 2.3 | 3 | 0.51 |  |
| Study attrition: Low ROB | 0 | 0.0% | 3 | 1 | 50.0% | 2 | 1 | 100.0% | 1 | 5 | 37.5% | 14 | 12.5 | 3 | 0.06 |  |
| Prognostic Factor Measurement: Low ROB | 1 | 33.3% | 3 | 0 | 0.0% | 2 | 0 | 0.0% | 1 | 6 | 42.9% | 14 | 8.1 | 3 | 0.52 |  |
| Outcome measurement: Low ROB | 2 | 66.7% | 3 | 2 | 100.0% | 2 | 1 | 100.0% | 1 | 12 | 85.7% | 14 | 2.7 | 3 | 0.85 |  |
| Study confounding: Low ROB | 1 | 33.3% | 3 | 1 | 50.0% | 2 | 0 | 0.0% | 1 | 9 | 64.3% | 14 | 7.2 | 3 | 0.30 |  |
| Statistical analysis and reporting: Low ROB | 3 | 100.0% | 3 | 2 | 100.0% | 2 | 1 | 100.0% | 1 | 12 | 85.7% | 14 | 0.95 | 3 | 0.82 |  |
| **Negative symptoms and Vocational functioning** | | | | | | | | | | | | | | | | |
| **Subgroup:** | 1. both moderate-high improvement | | | 2. Only one moderate- high improvement | | | 3. Both marginal-no improvement | | | 4. No improvement or deterioration | | | **ANOVA** | | | |
| **Demographic variables** | M | SD | N | M | SD | N | M | SD | N | M | SD | N | F | Df | p | Subgroup differences |
| Age | 26.5 | 2.4 | 2 | 24.2 | 4.2 | 6 |  |  |  | 31.2 | 7.8 | 12 | 2.2 | 2 | 0.14 |  |
| Age at onset | 21.1 |  | 1 | 20.5 | 0.8 | 5 |  |  |  | 24.3 | 5.2 | 10 | 1.4 | 2 | 0.29 |  |
| Ethnicity: % Caucasian or born in country of residence | 43.1 |  | 1 | 62.8 | 31.9 | 6 |  |  |  | 61.2 | 13.5 | 4 | 0.2 | 2 | 0.79 |  |
| Female gender (%)* | 36.7 | 15.9 | 2 | 30.3 | 10.9 | 6 |  |  |  | 41.6 | 9.6 | 12 | 2.4 | 2 | 0.12 | 2<4 |
| Marital status: % not married/in relationship | 96.0 |  | 1 | 83.6 | 11.2 | 4 |  |  |  | 78.7 | 12.4 | 6 | 1.0 | 2 | 0.43 |  |
|  | n | % | N | n | % | N | n | % | N | n | % | N | χ^2^ | df | p | Subgroup differences |
| Education level (high level) | 2 | 100.0% | 2 | 3 | 60.0% | 5 |  |  |  | 3 | 30.0% | 10 | 3.8 | 2 | 0.15 |  |
| **Clinical variables** | M | SD | N | M | SD | N | M | SD | N | M | SD | N | F | Df | p | Subgroup differences |
| Duration of untreated psychosis in weeks | 8.7 |  | 1 | 20.6 | 16.0 | 5 |  |  |  | 22.3 | 13.0 | 5 | 0.4 | 2 | 0.71 |  |
| Duration of illness (DOI) in years | 3.7 |  | 1 | 9.5 | 9.4 | 6 |  |  |  | 5.6 | 6.9 | 12 | 0.6 | 2 | 0.56 |  |
| Schizophrenia diagnosis (%) | 70.2 | 14.4 | 2 | 61.6 | 8.1 | 4 |  |  |  | 74.7 | 23.5 | 11 | 0.6 | 2 | 0.57 |  |
|  | n | % | N | n | % | N | n | % | N | n | % | N | χ^2^ | df | p | Subgroup differences |
| Antipsychotic use by all participants* | 2 | 100.0% | 2 | 1 | 16.7% | 6 |  |  |  | 2 | 16.7% | 12 | 6.7 | 2 | 0.04 |  |
| Combined treatment of psychosocial and pharmacological therapy provided* | 1 | 50.0% | 2 | 4 | 80.0% | 5 |  |  |  | 1 | 14.3% | 7 | 5.2 | 2 | 0.08 | 2>4 |
| Treatment provided focused on targeted outcomes. | 1 | 100.0% | 1 | 3 | 75.0% | 4 |  |  |  | 1 | 14.3% | 7 | 5.4 | 2 | 0.07 |  |
| **Symptoms** | M | SD | N | M | SD | N | M | SD | N | M | SD | N | F | Df | p | Subgroup differences |
| Depressive symptoms |  |  |  |  |  |  |  |  |  |  |  |  |  |  |  |  |
| Disorganization symptoms |  |  |  |  |  |  |  |  |  |  |  |  |  |  |  |  |
| Negative symptoms | 90.3 | 0.0 | 2 | 68.6 | 21.3 | 6 |  |  |  | 64.1 | 28.1 | 11 | 0.9 | 2 | 0.42 |  |
| Overall symptoms | 70.6 | 29.1 | 2 | 62.5 | 9.0 | 4 |  |  |  | 51.4 | 29.6 | 6 | 0.5 | 2 | 0.60 |  |
| Positive symptoms | 87.9 | 2.1 | 2 | 67.4 | 21.2 | 5 |  |  |  | 48.5 | 33.0 | 11 | 1.9 | 2 | 0.18 |  |
| **Functioning** | M | SD | N | M | SD | N | M | SD | N | M | SD | N | F | Df | p | Subgroup differences |
| Independence |  |  |  |  |  |  |  |  |  |  |  |  |  |  |  |  |
| Overall social functioning | 60.0 |  | 1 |  |  |  |  |  |  | 51.3 | 17.5 | 9 | 0.2 | 1 | 0.65 |  |
| Prosocial behavior | 49.6 | 20.9 | 2 | 56.1 | 11.7 | 3 |  |  |  | 57.1 | 15.3 | 6 | 0.2 | 2 | 0.83 |  |
| Vocational functioning | 35.0 |  | 1 | 32.0 | 12.5 | 4 |  |  |  | 48.2 | 19.8 | 11 | 1.3 | 2 | 0.32 |  |
| **Personal recovery and QOL** | M | SD | N | M | SD | N | M | SD | N | M | SD | N | F | Df | p | Subgroup differences |
| Personal recovery |  |  |  |  |  |  |  |  |  |  |  |  |  |  |  |  |
| Subjective quality of life |  |  |  |  |  |  |  |  |  |  |  |  |  |  |  |  |
| **Cognition** | M | SD | N | M | SD | N | M | SD | N | M | SD | N | F | Df | p | Subgroup differences |
| Executive functioning |  |  |  |  |  |  |  |  |  |  |  |  |  |  |  |  |
| Overall cognition |  |  |  |  |  |  |  |  |  |  |  |  |  |  |  |  |
| Processing speed |  |  |  |  |  |  |  |  |  |  |  |  |  |  |  |  |
| Verbal memory |  |  |  |  |  |  |  |  |  |  |  |  |  |  |  |  |
| **Study characteristics** | M | SD | N | M | SD | N | M | SD | N | M | SD | N | F | Df | p | Subgroup differences |
| Attrition rate | 28.0 | 33.9 | 2 | 35.6 | 18.8 | 6 |  |  |  | 20.9 | 12.4 | 11 | 1.5 | 2 | 0.25 |  |
|  | n | % | N | n | % | N | n | % | N | n | % | N | χ^2^ | df | p | Subgroup differences |
| DOI subgroup overlap (yes vs no) |  |  |  | 2 | 50.0% | 4 |  |  |  | 5 | 71.4% | 7 | 0.5 | 1 | 0.58 |  |
| Publication year <10 years | 2 | 100.0% | 2 | 4 | 66.7% | 6 |  |  |  | 4 | 33.3% | 12 | 4.0 | 2 | 0.14 |  |
| Study design: clinical trial | 1 | 50.0% | 2 | 4 | 66.7% | 6 |  |  |  | 2 | 16.7% | 12 | 4.6 | 2 | 0.10 |  |
| **Quality assessment** | n | % | N | n | % | N | n | % | N | n | % | N | χ^2^ | df | p | Subgroup differences |
| Study participation: Low ROB | 2 | 100.0% | 2 | 4 | 66.7% | 6 |  |  |  | 5 | 41.7% | 12 | 2.8 | 2 | 0.24 |  |
| Study attrition: Low ROB | 1 | 50.0% | 2 | 2 | 33.3% | 6 |  |  |  | 4 | 33.3% | 12 | 2.3 | 2 | 0.68 |  |
| Prognostic Factor Measurement: Low ROB | 1 | 50.0% | 2 | 1 | 16.7% | 6 |  |  |  | 5 | 41.7% | 12 | 4.7 | 3 | 0.58 |  |
| Outcome measurement: Low ROB | 1 | 50.0% | 2 | 5 | 83.3% | 6 |  |  |  | 8 | 66.7% | 12 | 2.6 | 2 | 0.62 |  |
| Study confounding: Low ROB | 0 | 0.0% | 2 | 1 | 16.7% | 6 |  |  |  | 8 | 66.7% | 12 | 7.2 | 2 | 0.13 |  |
| Statistical analysis and reporting: Low ROB | 2 | 100.0% | 2 | 5 | 83.3% | 6 |  |  |  | 10 | 83.3% | 12 | 0.4 | 2 | 0.82 |  |
| **Positive symptoms and overall social functioning** | | | | | | | | | | | | | | | | |
| **Subgroup:** | 1. both moderate-high improvement | | | 2. Only one moderate- high improvement | | | 3. Both marginal-no improvement | | | 4. No improvement or deterioration | | | **ANOVA** | | | |
| **Demographic variables** | M | SD | N | M | SD | N | M | SD | N | M | SD | N | F | Df | p | Subgroup differences |
| Age* | 26.7 | 4.4 | 17 | 27.2 | 7.3 | 8 | 35.2 | 13.2 | 4 | 39.3 | 13.2 | 22 | 5.9 | 3 | 0.00 | 1<3;4 / 2>4 |
| Age at onset | 23.0 | 3.5 | 16 | 23.2 | 2.7 | 6 | 26.7 | 4.7 | 3 | 24.1 | 2.6 | 18 | 1.3 | 3 | 0.27 |  |
| Ethnicity: % Caucasian or born in country of residence | 65.9 | 22.0 | 7 | 68.3 |  | 1 | 67.5 |  | 1 | 66.6 | 17.0 | 6 | 0.0 | 3 | 1.00 |  |
| Female gender (%) | 37.0 | 10.7 | 17 | 38.3 | 7.5 | 7 | 34.2 | 10.3 | 4 | 35.6 | 10.6 | 22 | 0.2 | 3 | 0.89 |  |
| Marital status: % not married/in relationship | 79.5 |  | 1 | 96.0 |  | 1 | 73.0 | 9.1 | 2 | 83.2 | 10.0 | 7 | 1.3 | 3 | 0.35 |  |
|  | n | % | N | n | % | N | n | % | N | n | % | N | χ^2^ | df | p | Subgroup differences |
| Education level (high level) | 9 | 69.2% | 13 | 3 | 60.0% | 5 | 1 | 33.3% | 3 | 4 | 33.3% | 12 | 3.8 | 3 | 0.29 |  |
| **Clinical variables** | M | SD | N | M | SD | N | M | SD | N | M | SD | N | F | Df | p | Subgroup differences |
| Duration of untreated psychosis in weeks | 20.6 | 15.0 | 8 | 25.8 | 11.0 | 5 | 13.7 | 0.6 | 2 | 19.3 | 18.9 | 3 | 0.4 | 3 | 0.76 |  |
| Duration of illness (DOI) in years* | 3.3 | 3.4 | 17 | 4.5 | 5.8 | 7 | 9.5 | 14.9 | 4 | 13.4 | 12.4 | 20 | 3.8 | 3 | 0.02 | 1<4 |
| Schizophrenia diagnosis (%) | 79.2 | 19.7 | 13 | 71.7 | 21.1 | 4 | 74.1 | 30.1 | 4 | 91.2 | 16.7 | 19 | 2.0 | 3 | 0.13 |  |
|  | n | % | N | n | % | N | n | % | N | n | % | N | χ^2^ | df | p | Subgroup differences |
| Antipsychotic use by all participants | 6 | 35.3% | 17 | 3 | 37.5% | 8 | 0 | 0.0% | 4 | 9 | 40.9% | 22 | 2.5 | 3 | 0.48 |  |
| Combined treatment of psychosocial and pharmacological therapy provided | 5 | 38.5% | 13 | 1 | 25.0% | 4 | 2 | 100.0% | 2 | 2 | 18.2% | 11 | 5.4 | 3 | 0.14 |  |
| Treatment provided focused on targeted outcomes. | 5 | 62.5% | 8 | 2 | 40.0% | 5 | 0 | 0.0% | 2 | 6 | 35.3% | 17 | 3.2 | 3 | 0.37 |  |
| **Symptoms** | M | SD | N | M | SD | N | M | SD | N | M | SD | N | F | Df | p | Subgroup differences |
| Depressive symptoms* | 53.0 | 18.0 | 8 | 80.3 | 27.2 | 2 | 21.2 |  | 1 | 62.0 | 15.3 | 12 | 3.1 | 3 | 0.05 | 3<4 |
| Disorganization symptoms | 41.4 | 30.6 | 7 | 15.4 | 6.8 | 2 |  |  |  | 68.5 | 36.3 | 5 | 2.3 | 2 | 0.15 |  |
| Negative symptoms | 45.1 | 26.6 | 16 | 58.6 | 26.0 | 7 | 63.4 | 33.6 | 3 | 60.7 | 26.0 | 21 | 1.2 | 3 | 0.32 |  |
| Overall symptoms | 51.0 | 19.4 | 10 | 61.8 | 19.1 | 6 | 65.3 | 32.8 | 2 | 59.0 | 22.7 | 15 | 0.5 | 3 | 0.69 |  |
| Positive symptoms | 55.3 | 26.7 | 16 | 64.6 | 21.4 | 7 | 76.9 | 18.8 | 3 | 68.9 | 25.1 | 22 | 1.2 | 3 | 0.32 |  |
| **Functioning** | M | SD | N | M | SD | N | M | SD | N | M | SD | N | F | Df | p | Subgroup differences |
| Independence |  |  |  |  |  |  |  |  |  |  |  |  |  |  |  |  |
| Overall social functioning* | 47.2 | 16.7 | 16 | 39.7 | 21.3 | 6 | 49.1 | 21.2 | 3 | 57.2 | 16.9 | 22 | 2.0 | 3 | 0.13 | 2>4 |
| Prosocial behavior | 49.6 | 12.1 | 4 | 34.8 |  | 1 | 58.3 | 14.3 | 3 | 57.5 | 21.2 | 4 | 0.7 | 3 | 0.60 |  |
| Vocational functioning | 36.6 | 22.1 | 4 | 58.3 | 29.9 | 4 | 63.8 | 23.0 | 3 | 35.3 | 18.7 | 7 | 1.7 | 3 | 0.21 |  |
| **Personal recovery and QOL** | M | SD | N | M | SD | N | M | SD | N | M | SD | N | F | Df | p | Subgroup differences |
| Personal recovery | 36.3 |  | 1 | 32.9 | 12.5 | 3 | 51.6 |  | 1 | 62.9 | 27.3 | 5 | 1.2 | 3 | 0.40 |  |
| Subjective quality of life | 51.5 | 19.4 | 3 | 17.1 |  | 1 | 53.7 |  | 1 | 47.7 | 15.9 | 7 | 1.2 | 3 | 0.38 |  |
| **Cognition** | M | SD | N | M | SD | N | M | SD | N | M | SD | N | F | Df | p | Subgroup differences |
| Executive functioning | 58.0 | 29.5 | 3 | 46.0 |  | 1 | 55.9 | 56.1 | 2 | 39.1 | 47.8 | 6 | 0.1 | 3 | 0.93 |  |
| Overall cognition* | 54.6 | 25.5 | 9 | 69.2 |  | 1 | 44.0 | 27.9 | 2 | 28.5 | 22.3 | 8 | 2.0 | 3 | 0.15 | 1>4 |
| Processing speed* | 49.4 | 23.3 | 3 | 54.0 |  | 1 | 1.7 | 2.2 | 2 | 36.8 | 39.8 | 4 | 1.1 | 3 | 0.42 | 2>3 |
| Verbal memory* | 74.1 | 13.3 | 3 | 72.6 |  | 1 | 19.7 | 8.6 | 2 | 53.0 | 26.3 | 6 | 2.7 | 3 | 0.12 | 1>3 |
| **Study characteristics** | M | SD | N | M | SD | N | M | SD | N | M | SD | N | F | Df | p | Subgroup differences |
| Attrition rate* | 38.6 | 15.2 | 17 | 20.7 | 9.6 | 6 | 29.0 | 29.4 | 3 | 25.5 | 14.7 | 22 | 3.2 | 3 | 0.03 | 1>2;4 |
|  | n | % | N | n | % | N | n | % | N | n | % | N | χ^2^ | df | p | Subgroup differences |
| DOI subgroup overlap (yes vs no) | 5 | 45.5% | 11 | 2 | 40.0% | 5 | 0 | 0.0% | 2 | 9 | 64.3% | 14 | 3.4 | 3 | 0.33 |  |
| Publication year <10 years | 11 | 64.7% | 17 | 5 | 62.5% | 8 | 3 | 75.0% | 4 | 14 | 63.6% | 22 | 0.2 | 3 | 0.98 |  |
| Study design: clinical trial | 5 | 29.4% | 17 | 2 | 25.0% | 8 | 0 | 0.0% | 3 | 12 | 57.1% | 21 | 6.2 | 3 | 0.10 |  |
| **Quality assessment** | n | % | N | n | % | N | n | % | N | n | % | N | χ^2^ | df | p | Subgroup differences |
| Study participation: Low ROB | 13 | 76.5% | 17 | 5 | 62.5% | 8 | 0 | 0.0% | 4 | 9 | 40.9% | 22 | 10.9 | 3 | 0.09 |  |
| Study attrition: Low ROB | 3 | 17.6% | 17 | 3 | 37.5% | 8 | 1 | 25.0% | 4 | 10 | 45.5% | 22 | 5.6 | 3 | 0.47 |  |
| Prognostic Factor Measurement: Low ROB | 7 | 41.2% | 17 | 3 | 37.5% | 8 | 1 | 25.0% | 4 | 4 | 18.2% | 22 | 10.8 | 3 | 0.29 |  |
| Outcome measurement: Low ROB | 14 | 82.4% | 17 | 7 | 87.5% | 8 | 4 | 100.0% | 4 | 16 | 72.7% | 22 | 2.9 | 3 | 0.82 |  |
| Study confounding: Low ROB | 10 | 58.8% | 17 | 5 | 62.5% | 8 | 3 | 75.0% | 4 | 7 | 31.8% | 22 | 7.0 | 3 | 0.32 |  |
| Statistical analysis and reporting: Low ROB | 13 | 76.5% | 17 | 7 | 87.5% | 8 | 3 | 75.0% | 4 | 19 | 86.4% | 22 | 3.0 | 3 | 0.81 |  |
| **Positive symptoms and Prosocial behavior** | | | | | | | | | | | | | | | | |
| **Subgroup:** | 1. both moderate-high improvement | | | 2. Only one moderate- high improvement | | | 3. Both marginal-no improvement | | | 4. No improvement or deterioration | | | **ANOVA** | | | |
| **Demographic variables** | M | SD | N | M | SD | N | M | SD | N | M | SD | N | F | Df | p | Subgroup differences |
| Age | 29.5 | 7.5 | 4 | 27.4 | 1.7 | 5 | 30.8 | 2.6 | 3 | 36.9 | 11.5 | 11 | 1.6 | 3 | 0.22 |  |
| Age at onset | 23.4 | 3.4 | 4 | 21.8 | 4.6 | 3 | 26.3 | 8.3 | 2 | 22.2 | 1.9 | 10 | 0.9 | 3 | 0.48 |  |
| Ethnicity: % Caucasian or born in country of residence |  |  |  |  |  |  |  |  |  |  |  |  |  |  |  |  |
| Female gender (%)* | 35.4 | 7.1 | 4 | 42.9 | 9.1 | 5 | 32.8 | 13.9 | 3 | 31.5 | 9.2 | 11 | 1.7 | 3 | 0.20 | 2>4 |
| Marital status: % not married/in relationship |  |  |  |  |  |  |  |  |  |  |  |  |  |  |  |  |
|  | n | % | N | n | % | N | n | % | N | n | % | N | χ^2^ | df | p | Subgroup differences |
| Education level (high level)* | 2 | 100.0% | 2 | 3 | 75.0% | 4 | 2 | 66.7% | 3 | 1 | 12.5% | 8 | 7.8 | 3 | 0.05 |  |
| **Clinical variables** | M | SD | N | M | SD | N | M | SD | N | M | SD | N | F | Df | p | Subgroup differences |
| Duration of untreated psychosis in weeks | 9.5 | 1.1 | 2 | 25.1 | 16.0 | 4 | 13.3 |  | 1 | 18.0 | 10.2 | 3 | 0.8 | 3 | 0.56 |  |
| Duration of illness (DOI) in years | 10.6 | 7.0 | 4 | 3.8 | 4.1 | 4 | 5.7 | 6.0 | 3 | 12.8 | 10.0 | 10 | 1.4 | 3 | 0.28 |  |
| Schizophrenia diagnosis (%)* | 86.6 | 16.5 | 4 | 58.8 | 13.8 | 4 | 55.7 | 13.6 | 3 | 83.9 | 22.8 | 10 | 3.1 | 3 | 0.06 | 1>2;3 |
|  | n | % | N | n | % | N | n | % | N | n | % | N | χ^2^ | df | p | Subgroup differences |
| Antipsychotic use by all participants | 3 | 75.0% | 4 | 2 | 40.0% | 5 | 0 | 0.0% | 3 | 3 | 27.3% | 11 | 4.8 | 3 | 0.19 |  |
| Combined treatment of psychosocial and pharmacological therapy provided | 2 | 66.7% | 3 | 2 | 50.0% | 4 | 2 | 100.0% | 2 | 2 | 28.6% | 7 | 3.6 | 3 | 0.31 |  |
| Treatment provided focused on targeted outcomes* | 3 | 100.0% | 3 | 3 | 75.0% | 4 | 0 | 0.0% | 2 | 1 | 16.7% | 6 | 8.6 | 3 | 0.04 | 1>4 |
| **Symptoms** | M | SD | N | M | SD | N | M | SD | N | M | SD | N | F | Df | p | Subgroup differences |
| Depressive symptoms |  |  |  |  |  |  |  |  |  |  |  |  |  |  |  |  |
| Disorganization symptoms |  |  |  |  |  |  |  |  |  |  |  |  |  |  |  |  |
| Negative symptoms | 66.1 | 25.7 | 4 | 63.1 | 40.5 | 4 | 67.2 | 35.9 | 3 | 69.2 | 23.0 | 10 | 0.0 | 3 | 0.99 |  |
| Overall symptoms | 51.7 | 20.1 | 3 | 51.1 | 40.3 | 3 | 49.1 | 9.8 | 2 | 68.0 | 22.0 | 5 | 0.5 | 3 | 0.72 |  |
| Positive symptoms | 44.8 | 38.1 | 4 | 65.7 | 39.1 | 4 | 66.0 | 10.1 | 3 | 67.6 | 32.9 | 11 | 0.5 | 3 | 0.70 |  |
| **Functioning** | M | SD | N | M | SD | N | M | SD | N | M | SD | N | F | Df | p | Subgroup differences |
| Independence | 61.1 | 27.6 | 2 | 11.4 |  | 1 | 74.3 | 21.8 | 3 | 42.8 | 25.4 | 5 | 2.0 | 3 | 0.20 |  |
| Overall social functioning* | 42.0 | 3.0 | 2 | 38.2 | 23.7 | 5 | 40.1 | 20.3 | 2 | 66.8 | 16.5 | 8 | 3.0 | 3 | 0.07 | 2<4 |
| Prosocial behavior | 48.9 | 20.9 | 4 | 42.9 | 11.4 | 2 | 56.9 | 16.0 | 3 | 53.8 | 17.2 | 7 | 0.3 | 3 | 0.81 |  |
| Vocational functioning | 38.0 |  | 1 | 46.9 | 24.5 | 3 | 38.8 | 21.0 | 3 | 46.7 | 25.3 | 5 | 0.1 | 3 | 0.96 |  |
| **Personal recovery and QOL** | M | SD | N | M | SD | N | M | SD | N | M | SD | N | F | Df | p | Subgroup differences |
| Personal recovery |  |  |  |  |  |  |  |  |  |  |  |  |  |  |  |  |
| Subjective quality of life | 61.8 | 2.7 | 2 | 68.7 | 10.0 | 2 | 87.5 |  | 1 | 57.7 | 10.5 | 5 | 3.0 | 3 | 0.12 |  |
| **Cognition** | M | SD | N | M | SD | N | M | SD | N | M | SD | N | F | Df | p | Subgroup differences |
| Executive functioning |  |  |  |  |  |  |  |  |  |  |  |  |  |  |  |  |
| Overall cognition |  |  |  |  |  |  |  |  |  |  |  |  |  |  |  |  |
| Processing speed |  |  |  |  |  |  |  |  |  |  |  |  |  |  |  |  |
| Verbal memory |  |  |  |  |  |  |  |  |  |  |  |  |  |  |  |  |
| **Study characteristics** | M | SD | N | M | SD | N | M | SD | N | M | SD | N | F | Df | p | Subgroup differences |
| Attrition rate* | 53.6 | 13.5 | 4 | 19.4 | 11.8 | 5 | 11.5 | 16.3 | 2 | 25.7 | 11.4 | 11 | 8.1 | 3 | 0.00 | 1>2;3;4 |
|  | n | % | N | n | % | N | n | % | N | n | % | N | χ^2^ | df | p | Subgroup differences |
| DOI subgroup overlap (yes vs no) | 1 | 50.0% | 2 | 2 | 66.7% | 3 | 1 | 50.0% | 2 | 2 | 33.3% | 6 | 0.9 | 3 | 0.82 |  |
| Publication year <10 years* | 4 | 100.0% | 4 | 2 | 40.0% | 5 | 2 | 66.7% | 3 | 3 | 27.3% | 11 | 6.8 | 3 | 0.08 | 1>4 |
| Study design: clinical trial* | 4 | 100.0% | 4 | 4 | 80.0% | 5 | 1 | 33.3% | 3 | 2 | 18.2% | 11 | 10.6 | 3 | 0.01 | 1>4 / 2>4 |
| **Quality assessment** | n | % | N | n | % | N | n | % | N | n | % | N | χ^2^ | df | p | Subgroup differences |
| Study participation: Low ROB* | 3 | 75.0% | 4 | 5 | 100.0% | 5 | 1 | 33.3% | 3 | 2 | 18.2% | 11 | 10.8 | 3 | 0.01 | 1>4 /2>3 / 2>4 |
| Study attrition: Low ROB* | 0 | 0.0% | 4 | 2 | 40.0% | 5 | 2 | 66.7% | 3 | 3 | 27.3% | 11 | 13.2 | 3 | 0.04 | 1<4 |
| Prognostic Factor Measurement: Low ROB | 1 | 25.0% | 4 | 4 | 80.0% | 5 | 1 | 33.3% | 3 | 3 | 27.3% | 11 | 15.4 | 3 | 0.08 |  |
| Outcome measurement: Low ROB | 3 | 75.0% | 4 | 4 | 80.0% | 5 | 3 | 100.0% | 3 | 10 | 90.9% | 11 | 4.4 | 3 | 0.62 |  |
| Study confounding: Low ROB | 1 | 25.0% | 4 | 3 | 60.0% | 5 | 1 | 33.3% | 3 | 7 | 63.6% | 11 | 8.3 | 3 | 0.22 |  |
| Statistical analysis and reporting: Low ROB | 4 | 100.0% | 4 | 5 | 100.0% | 5 | 2 | 66.7% | 3 | 9 | 81.8% | 11 | 4.3 | 3 | 0.63 |  |
| **Depression and overall social functioning** | | | | | | | | | | | | | | | | |
| **Subgroup:** | 1. both moderate-high improvement | | | 2. Only one moderate- high improvement | | | 3. Both marginal-no improvement | | | 4. No improvement or deterioration | | | **ANOVA** | | | |
| **Demographic variables** | M | SD | N | M | SD | N | M | SD | N | M | SD | N | F | Df | p | Subgroup differences |
| Age* | 29.4 | 5.5 | 8 | 29.0 | 3.4 | 6 | 32.0 | 8.1 | 2 | 40.6 | 12.4 | 8 | 3.1 | 3 | 0.05 | 1<4 / 2<4 |
| Age at onset | 25.1 | 3.5 | 7 | 25.3 | 2.9 | 6 | 24.5 | 0.7 | 2 | 24.4 | 3.4 | 6 | 0.1 | 3 | 0.96 |  |
| Ethnicity: % Caucasian or born in country of residence* | 57.0 | 14.0 | 4 | 83.4 | 11.0 | 4 |  |  |  | 68.0 | 18.2 | 2 | 3.8 | 2 | 0.08 | 1<2 |
| Female gender (%) | 38.2 | 7.9 | 8 | 36.9 | 7.7 | 6 | 30.9 | 6.6 | 2 | 34.9 | 13.0 | 8 | 0.4 | 3 | 0.78 |  |
| Marital status: % not married/in relationship |  |  |  |  |  |  |  |  |  |  |  |  |  |  |  |  |
|  | n | % | N | n | % | N | n | % | N | n | % | N | χ^2^ | df | p | Subgroup differences |
| Education level (high level) | 3 | 50.0% | 6 | 2 | 66.7% | 3 |  |  |  | 3 | 50.0% | 6 | 0.3 | 2 | 0.88 |  |
| **Clinical variables** | M | SD | N | M | SD | N | M | SD | N | M | SD | N | F | Df | p | Subgroup differences |
| Duration of untreated psychosis in weeks |  |  |  |  |  |  |  |  |  |  |  |  |  |  |  |  |
| Duration of illness (DOI) in years* | 3.8 | 3.7 | 8 | 3.7 | 4.3 | 6 | 7.5 | 8.8 | 2 | 15.2 | 11.7 | 6 | 3.5 | 3 | 0.04 | 1<4 / 2<4 |
| Schizophrenia diagnosis (%) | 78.7 | 21.2 | 8 | 80.3 | 24.9 | 3 | 100.0 | 0.0 | 2 | 95.1 | 10.8 | 5 | 1.3 | 3 | 0.32 |  |
|  | n | % | N | n | % | N | n | % | N | n | % | N | χ^2^ | df | p | Subgroup differences |
| Antipsychotic use by all participants* | 5 | 62.5% | 8 | 0 | 0.0% | 6 | 0 | 0.0% | 2 | 3 | 37.5% | 8 | 7.1 | 3 | 0.07 | 1>2 |
| Combined treatment of psychosocial and pharmacological therapy provided | 1 | 33.3% | 3 | 2 | 50.0% | 4 | 0 | 0.0% | 1 | 1 | 20.0% | 5 | 1.4 | 3 | 0.70 |  |
| Treatment provided focused on targeted outcomes. | 2 | 66.7% | 3 | 2 | 50.0% | 4 |  |  |  | 3 | 42.9% | 7 | 0.5 | 2 | 0.78 |  |
| **Symptoms** | M | SD | N | M | SD | N | M | SD | N | M | SD | N | F | Df | p | Subgroup differences |
| Depressive symptoms | 51.1 | 12.6 | 5 | 59.4 | 27.9 | 6 | 61.8 |  | 1 | 64.6 | 18.9 | 8 | 0.4 | 3 | 0.73 |  |
| Disorganization symptoms |  |  |  |  |  |  |  |  |  |  |  |  |  |  |  |  |
| Negative symptoms | 44.4 | 35.7 | 6 | 46.7 | 29.9 | 6 | 94.5 |  | 1 | 63.7 | 19.2 | 7 | 1.3 | 3 | 0.31 |  |
| Overall symptoms | 59.9 | 36.4 | 4 | 45.4 | 14.3 | 4 |  |  |  | 62.5 | 20.0 | 6 | 0.6 | 2 | 0.55 |  |
| Positive symptoms | 37.8 | 29.0 | 7 | 37.3 | 30.6 | 5 | 89.4 |  | 1 | 66.3 | 27.1 | 7 | 2.1 | 3 | 0.14 |  |
| **Functioning** | M | SD | N | M | SD | N | M | SD | N | M | SD | N | F | Df | p | Subgroup differences |
| Independence |  |  |  |  |  |  |  |  |  |  |  |  |  |  |  |  |
| Overall social functioning* | 55.6 | 20.7 | 7 | 40.8 | 11.7 | 6 | 44.7 |  | 1 | 62.5 | 15.7 | 8 | 2.1 | 3 | 0.14 | 2<4 |
| Prosocial behavior |  |  |  |  |  |  |  |  |  |  |  |  |  |  |  |  |
| Vocational functioning |  |  |  |  |  |  |  |  |  |  |  |  |  |  |  |  |
| **Personal recovery and QOL** | M | SD | N | M | SD | N | M | SD | N | M | SD | N | F | Df | p | Subgroup differences |
| Personal recovery |  |  |  |  |  |  |  |  |  |  |  |  |  |  |  |  |
| Subjective quality of life |  |  |  |  |  |  |  |  |  |  |  |  |  |  |  |  |
| **Cognition** | M | SD | N | M | SD | N | M | SD | N | M | SD | N | F | Df | p | Subgroup differences |
| Executive functioning |  |  |  |  |  |  |  |  |  |  |  |  |  |  |  |  |
| Overall cognition |  |  |  |  |  |  |  |  |  |  |  |  |  |  |  |  |
| Processing speed |  |  |  |  |  |  |  |  |  |  |  |  |  |  |  |  |
| Verbal memory |  |  |  |  |  |  |  |  |  |  |  |  |  |  |  |  |
| **Study characteristics** | M | SD | N | M | SD | N | M | SD | N | M | SD | N | F | Df | p | Subgroup differences |
| Attrition rate | 38.9 | 16.5 | 8 | 31.9 | 17.1 | 6 | 37.8 | 29.7 | 2 | 26.6 | 15.3 | 8 | 0.7 | 3 | 0.54 |  |
|  | n | % | N | n | % | N | n | % | N | n | % | N | χ^2^ | df | p | Subgroup differences |
| DOI subgroup overlap (yes vs no) | 1 | 33.3% | 3 | 3 | 75.0% | 4 | 1 | 100.0% | 1 | 3 | 60.0% | 5 | 1.9 | 3 | 0.58 |  |
| Publication year <10 years | 6 | 75.0% | 8 | 3 | 50.0% | 6 | 1 | 50.0% | 2 | 7 | 87.5% | 8 | 2.8 | 3 | 0.42 |  |
| Study design: clinical trial | 4 | 50.0% | 8 | 3 | 50.0% | 6 | 0 | 0.0% | 1 | 4 | 50.0% | 8 | 1.0 | 3 | 0.81 |  |
| **Quality assessment** | n | % | N | n | % | N | n | % | N | n | % | N | χ^2^ | df | p | Subgroup differences |
| Study participation: Low ROB | 5 | 62.5% | 8 | 6 | 100.0% | 6 | 0 | 0.0% | 2 | 5 | 62.5% | 8 | 7.1 | 3 | 0.07 |  |
| Study attrition: Low ROB | 2 | 25.0% | 8 | 2 | 33.3% | 3 | 1 | 50.0% | 2 | 3 | 37.5% | 8 | 3.3 | 3 | 0.78 |  |
| Prognostic Factor Measurement: Low ROB | 5 | 62.5% | 8 | 3 | 50.0% | 6 | 0 | 0.0% | 2 | 1 | 12.5% | 8 | 9.3 | 3 | 0.16 |  |
| Outcome measurement: Low ROB | 6 | 75.0% | 8 | 5 | 83.3% | 6 | 2 | 100.0% | 2 | 5 | 62.5% | 8 | 1.6 | 3 | 0.67 |  |
| Study confounding: Low ROB* | 5 | 62.5% | 8 | 4 | 66.7% | 6 | 2 | 100.0% | 2 | 1 | 12.6% | 8 | 14.7 | 3 | 0.02 |  |
| Statistical analysis and reporting: Low ROB | 7 | 87.5% | 8 | 6 | 100.0% | 6 | 2 | 100.0% | 2 | 8 | 100.0% | 8 | 2.1 | 3 | 0.56 |  |
| **Disorganization and overall social functioning** | | | | | | | | | | | | | | | | |
| **Subgroup:** | 1. both moderate-high improvement | | | 2. Only one moderate- high improvement | | | 3. Both marginal-no improvement | | | 4. No improvement or deterioration | | | **ANOVA** | | | |
| **Demographic variables** | M | SD | N | M | SD | N | M | SD | N | M | SD | N | F | Df | p | Subgroup differences |
| Age | 28.2 | 7.7 | 4 | 26.6 |  | 1 |  |  |  | 38.7 | 7.0 | 6 | 3.1 | 2 | 0.10 |  |
| Age at onset |  |  |  |  |  |  |  |  |  |  |  |  |  |  |  |  |
| Ethnicity: % Caucasian or born in country of residence |  |  |  |  |  |  |  |  |  |  |  |  |  |  |  |  |
| Female gender (%) | 36.2 | 7.2 | 4 | 54.2 |  | 1 |  |  |  | 35.3 | 10.7 | 6 | 1.7 | 2 | 0.24 |  |
| Marital status: % not married/in relationship |  |  |  |  |  |  |  |  |  |  |  |  |  |  |  |  |
|  | n | % | N | n | % | N | n | % | N | n | % | N | χ^2^ | df | p | Subgroup differences |
| Education level (high level) |  |  |  |  |  |  |  |  |  |  |  |  |  |  |  |  |
| **Clinical variables** | M | SD | N | M | SD | N | M | SD | N | M | SD | N | F | Df | p | Subgroup differences |
| Duration of untreated psychosis in weeks |  |  |  |  |  |  |  |  |  |  |  |  |  |  |  |  |
| Duration of illness (DOI) in years | 4.8 | 4.4 | 4 | 9.8 |  | 1 |  |  |  | 9.4 | 5.6 | 5 | 1.0 | 2 | 0.42 |  |
| Schizophrenia diagnosis (%) |  |  |  |  |  |  |  |  |  |  |  |  |  |  |  |  |
|  | n | % | N | n | % | N | n | % | N | n | % | N | χ^2^ | df | p | Subgroup differences |
| Antipsychotic use by all participants | 2 | 50.0% | 4 | 0 | 0.0% | 1 |  |  |  | 1 | 16.7% | 6 | 1.8 | 2 | 0.42 |  |
| Combined treatment of psychosocial and pharmacological therapy provided |  |  |  |  |  |  |  |  |  |  |  |  |  |  |  |  |
| Treatment provided focused on targeted outcomes. |  |  |  |  |  |  |  |  |  |  |  |  |  |  |  |  |
| **Symptoms** | M | SD | N | M | SD | N | M | SD | N | M | SD | N | F | Df | p | Subgroup differences |
| Depressive symptoms |  |  |  |  |  |  |  |  |  |  |  |  |  |  |  |  |
| Disorganization symptoms | 32.1 | 39.2 | 4 | 63.7 |  | 1 |  |  |  | 58.4 | 42.1 | 5 | 0.5 | 2 | 0.61 |  |
| Negative symptoms* | 34.8 | 20.5 | 4 | 99.3 |  | 1 |  |  |  | 73.3 | 14.7 | 6 | 8.7 | 2 | 0.01 | 1<4 |
| Overall symptoms |  |  |  |  |  |  |  |  |  |  |  |  |  |  |  |  |
| Positive symptoms | 31.7 | 17.9 | 3 | 97.4 |  | 1 |  |  |  | 63.7 | 38.2 | 6 | 1.7 | 2 | 0.25 |  |
| **Functioning** | M | SD | N | M | SD | N | M | SD | N | M | SD | N | F | Df | p | Subgroup differences |
| Independence |  |  |  |  |  |  |  |  |  |  |  |  |  |  |  |  |
| Overall social functioning | 48.6 | 35.4 | 4 | 51.5 |  | 1 |  |  |  | 58.0 | 18.3 | 6 | 0.2 | 2 | 0.85 |  |
| Prosocial behavior |  |  |  |  |  |  |  |  |  |  |  |  |  |  |  |  |
| Vocational functioning |  |  |  |  |  |  |  |  |  |  |  |  |  |  |  |  |
| **Personal recovery and QOL** | M | SD | N | M | SD | N | M | SD | N | M | SD | N | F | Df | p | Subgroup differences |
| Personal recovery |  |  |  |  |  |  |  |  |  |  |  |  |  |  |  |  |
| Subjective quality of life |  |  |  |  |  |  |  |  |  |  |  |  |  |  |  |  |
| **Cognition** | M | SD | N | M | SD | N | M | SD | N | M | SD | N | F | Df | p | Subgroup differences |
| Executive functioning |  |  |  |  |  |  |  |  |  |  |  |  |  |  |  |  |
| Overall cognition |  |  |  |  |  |  |  |  |  |  |  |  |  |  |  |  |
| Processing speed |  |  |  |  |  |  |  |  |  |  |  |  |  |  |  |  |
| Verbal memory |  |  |  |  |  |  |  |  |  |  |  |  |  |  |  |  |
| **Study characteristics** | M | SD | N | M | SD | N | M | SD | N | M | SD | N | F | Df | p | Subgroup differences |
| Attrition rate | 45.1 | 12.9 | 4 | 28.9 |  | 1 |  |  |  | 32.8 | 17.9 | 6 | 0.8 | 2 | 0.47 |  |
|  | n | % | N | n | % | N | n | % | N | n | % | N | χ^2^ | df | p | Subgroup differences |
| DOI subgroup overlap (yes vs no) |  |  |  |  |  |  |  |  |  |  |  |  |  |  |  |  |
| Publication year <10 years | 2 | 50.0% | 4 | 0 | 0.0% | 1 |  |  |  | 5 | 83.3% | 6 | 3.1 | 2 | 0.22 |  |
| Study design: clinical trial | 1 | 25.0% | 4 | 1 | 100.0% | 1 |  |  |  | 3 | 50.0% | 6 | 1.9 | 2 | 0.38 |  |
| **Quality assessment** | n | % | N | n | % | N | n | % | N | n | % | N | χ^2^ | df | p | Subgroup differences |
| Study participation: Low ROB | 2 | 50.0% | 4 | 1 | 100.0% | 1 |  |  |  | 4 | 66.7% | 6 | 0.9 | 2 | 0.63 |  |
| Study attrition: Low ROB | 1 | 25.0% | 4 | 0 | 0.0% | 1 |  |  |  | 3 | 50.0% | 6 | 6.6 | 2 | 0.16 |  |
| Prognostic Factor Measurement: Low ROB | 0 | 0.0% | 4 | 1 | 100.0% | 1 |  |  |  | 0 | 100.0% | 6 | 13.7 | 2 | 0.06 |  |
| Outcome measurement: Low ROB | 4 | 100.0% | 4 | 0 | 0.0% | 1 |  |  |  | 4 | 66.7% | 6 | 4.3 | 2 | 0.12 |  |
| Study confounding: Low ROB | 3 | 75.0% | 4 | 1 | 100.0% | 1 |  |  |  | 1 | 16.7% | 6 | 4.6 | 2 | 0.10 |  |
| Statistical analysis and reporting: Low ROB | 3 | 75.0% | 4 | 1 | 100.0% | 1 |  |  |  | 4 | 66.7% | 6 | 0.5 | 2 | 0.78 |  |

**Symptoms and cognition associations**

| **Negative symptoms and Processing speed** | | | | | | | | | | | | | | | | | | |
| --- | --- | --- | --- | --- | --- | --- | --- | --- | --- | --- | --- | --- | --- | --- | --- | --- | --- | --- |
| **Subgroup:** | 1. both moderate-high improvement | | | 2. Only one moderate- high improvement | | | | | 3. Both marginal-no improvement | | | 4. No improvement or deterioration | | | **ANOVA** | | | |
| **Demographic variables** | M | SD | N | M | SD | | N | | M | SD | N | M | SD | N | F | Df | p | Subgroup differences |
| Age | 26.9 | 3.1 | 2 | 41.6 | 18.4 | | 2 | |  |  |  | 35.3 | 5.8 | 11 | 1.9 | 2 | 0.19 |  |
| Age at onset | 23.9 | 4.1 | 2 | 22.6 | 0.8 | | 2 | |  |  |  | 25.2 | 4.5 | 10 | 0.4 | 2 | 0.71 |  |
| Ethnicity: % Caucasian or born in country of residence |  |  |  |  |  | |  | |  |  |  |  |  |  |  |  |  |  |
| Female gender (%) | 33.7 | 11.7 | 2 | 36.5 | 2.7 | | 2 | |  |  |  | 35.9 | 14.8 | 11 | 0.0 | 2 | 0.98 |  |
| Marital status: % not married/in relationship |  |  |  |  |  | |  | |  |  |  |  |  |  |  |  |  |  |
|  | n | % | N | n | % | | N | | n | % | N | n | % | N | χ^2^ | df | p | Subgroup differences |
| Education level (high level) | 1 | 50.0% | 2 | 1 | 100.0% | | 1 | |  |  |  | 5 | 45.5% | 11 | 1.1 | 2 | 0.58 |  |
| **Clinical variables** | M | SD | N | M | SD | | N | | M | SD | N | M | SD | N | F | Df | p | Subgroup differences |
| Duration of untreated psychosis in weeks |  |  |  |  |  | |  | |  |  |  |  |  |  |  |  |  |  |
| Duration of illness (DOI) in years | 3.0 | 1.0 | 2 | 19.1 | 17.6 | | 2 | |  |  |  | 8.7 | 6.0 | 11 | 2.4 | 2 | 0.13 |  |
| Schizophrenia diagnosis (%) | 70.2 | 14.4 | 2 | 87.2 | 18.1 | | 2 | |  |  |  | 79.6 | 19.4 | 9 | 0.4 | 2 | 0.67 |  |
|  | n | % | N | n | % | | N | | n | % | N | n | % | N | χ^2^ | df | p | Subgroup differences |
| Antipsychotic use by all participants | 2 | 100.0% | 2 | 1 | 50.0% | | 2 | |  |  |  | 6 | 54.5% | 11 | 1.5 | 2 | 0.46 |  |
| Combined treatment of psychosocial and pharmacological therapy provided |  |  |  |  |  | |  | |  |  |  |  |  |  |  |  |  |  |
| Treatment provided focused on targeted outcomes* | 1 | 100.0% | 1 | 0 | 0.0% | | 2 | |  |  |  | 0 | 0.0% | 7 | 10.0 | 2 | 0.01 |  |
| **Symptoms** | M | SD | N | M | SD | | N | | M | SD | N | M | SD | N | F | Df | p | Subgroup differences |
| Depressive symptoms |  |  |  |  |  | |  | |  |  |  |  |  |  |  |  |  |  |
| Disorganization symptoms |  |  |  |  |  | |  | |  |  |  |  |  |  |  |  |  |  |
| Negative symptoms | 48.5 | 59.1 | 2 | 43.9 | 42.9 | | 2 | |  |  |  | 48.0 | 32.3 | 11 | 0.0 | 2 | 0.99 |  |
| Overall symptoms | 68.8 | 26.5 | 2 | 79.7 | 12.4 | | 2 | |  |  |  | 57.4 | 30.8 | 7 | 0.5 | 2 | 0.62 |  |
| Positive symptoms | 62.0 | 38.8 | 2 | 62.3 | 47.0 | | 2 | |  |  |  | 47.3 | 34.3 | 11 | 0.3 | 2 | 0.78 |  |
| **Functioning** | M | SD | N | M | SD | | N | | M | SD | N | M | SD | N | F | Df | p | Subgroup differences |
| Independence |  |  |  |  |  | |  | |  |  |  |  |  |  |  |  |  |  |
| Overall social functioning |  |  |  |  |  | |  | |  |  |  |  |  |  |  |  |  |  |
| Prosocial behavior |  |  |  |  |  | |  | |  |  |  |  |  |  |  |  |  |  |
| Vocational functioning |  |  |  |  |  | |  | |  |  |  |  |  |  |  |  |  |  |
| **Personal recovery and QOL** | M | SD | N | M | SD | | N | | M | SD | N | M | SD | N | F | Df | p | Subgroup differences |
| Personal recovery |  |  |  |  |  | |  | |  |  |  |  |  |  |  |  |  |  |
| Subjective quality of life |  |  |  |  |  | |  | |  |  |  |  |  |  |  |  |  |  |
| **Cognition** | M | SD | N | M | SD | | N | | M | SD | N | M | SD | N | F | Df | p | Subgroup differences |
| Executive functioning | 78.8 |  | 1 | 74.8 | 29.3 | | 2 | |  |  |  | 29.0 | 34.0 | 9 | 2.2 | 2 | 0.16 |  |
| Overall cognition | 85.9 | 19.8 | 2 | 61.8 | 2.7 | | 2 | |  |  |  | 34.7 | 29.0 | 7 | 3.3 | 2 | 0.09 |  |
| Processing speed | 22.7 |  | 1 | 28.1 | 39.5 | | 2 | |  |  |  | 44.2 | 31.7 | 9 | 0.4 | 2 | 0.71 |  |
| Verbal memory | 65.5 |  | 1 | 60.7 | 49.3 | | 2 | |  |  |  | 58.7 | 28.7 | 9 | 0.0 | 2 | 0.98 |  |
| **Study characteristics** | M | SD | N | M | SD | | N | | M | SD | N | M | SD | N | F | Df | p | Subgroup differences |
| Attrition rate | 39.8 | 17.3 | 2 | 14.1 | 19.9 | | 2 | |  |  |  | 33.1 | 15.0 | 11 | 1.6 | 2 | 0.25 |  |
|  | n | % | N | n | % | | N | | n | % | N | n | % | N | χ^2^ | df | p | Subgroup differences |
| DOI subgroup overlap (yes vs no) | 0 | 0.0% | 1 | 1 | 50.0% | | 2 | |  |  |  | 8 | 80.0% | 10 | 3.1 | 2 | 0.21 |  |
| Publication year <10 years | 1 | 50.0% | 2 | 0 | 0.0% | | 2 | |  |  |  | 5 | 45.5% | 11 | 1.6 | 2 | 0.46 |  |
| Study design: clinical trial | 1 | 50.0% | 2 | 0 | 0.0% | | 2 | |  |  |  | 3 | 27.3% | 11 | 1.3 | 2 | 0.53 |  |
| **Quality assessment** | n | % | N | n | | % | | N | n | % | N | n | % | N | χ^2^ | df | p | Subgroup differences |
| Study participation: Low ROB | 2 | 100.0% | 2 | 0 | | 0.0% | | 2 |  |  |  | 5 | 45.5% | 11 | 4.7 | 2 | 0.32 |  |
| Study attrition: Low ROB | 1 | 50.0% | 2 | 1 | | 50.0% | | 2 |  |  |  | 2 | 18.2% | 11 | 2.9 | 2 | 0.57 |  |
| Prognostic Factor Measurement: Low ROB | 1 | 50.0% | 2 | 0 | | 0.0% | | 2 |  |  |  | 2 | 18.2% | 11 | 4.2 | 2 | 0.39 |  |
| Outcome measurement: Low ROB | 1 | 50.0% | 2 | 2 | | 100.0% | | 2 |  |  |  | 10 | 90.9% | 11 | 2.8 | 2 | 0.25 |  |
| Study confounding: Low ROB | 1 | 50.0% | 2 | 1 | | 50.0% | | 2 |  |  |  | 5 | 45.5% | 11 | 2.2 | 2 | 0.70 |  |
| Statistical analysis and reporting: Low ROB |  |  |  |  | |  | |  |  |  |  |  |  |  |  |  |  |  |
| **Negative symptoms and Overall cognition** | | | | | | | | | | | | | | | | | | |
| **Subgroup:** | 1. both moderate-high improvement | | | 2. Only one moderate- high improvement | | | | | 3. Both marginal-no improvement | | | 4. No improvement or deterioration | | | **ANOVA** | | | |
| **Demographic variables** | M | SD | N | M | SD | | N | | M | SD | N | M | SD | N | F | Df | p | Subgroup differences |
| Age | 22.9 | 2.7 | 2 | 37.1 | 12.6 | | 5 | | 39.7 |  | 1 | 40.8 | 15.5 | 12 | 0.9 | 3 | 0.46 |  |
| Age at onset* | 20.8 | 0.4 | 2 | 26.7 | 2.8 | | 5 | | 24.0 |  | 1 | 24.4 | 4.5 | 11 | 1.1 | 3 | 0.36 | 1<2 |
| Ethnicity: % Caucasian or born in country of residence |  |  |  |  |  | |  | |  |  |  |  |  |  |  |  |  |  |
| Female gender (%)* | 32.4 | 9.8 | 2 | 46.1 | 8.5 | | 5 | | 71.8 |  | 1 | 32.9 | 11.8 | 12 | 5.1 | 3 | 0.01 | 2>4 / 3>4 |
| Marital status: % not married/in relationship |  |  |  |  |  | |  | |  |  |  |  |  |  |  |  |  |  |
|  | n | % | N | n | % | | N | | n | % | N | n | % | N | χ^2^ | df | p | Subgroup differences |
| Education level (high level) | 1 | 50.0% | 2 | 4 | 100.0% | | 4 | | 0 | 0.0% | 1 | 7 | 77.8% | 9 | 5.0 | 3 | 0.17 |  |
| **Clinical variables** | M | SD | N | M | SD | | N | | M | SD | N | M | SD | N | F | Df | p | Subgroup differences |
| Duration of untreated psychosis in weeks |  |  |  |  |  | |  | |  |  |  |  |  |  |  |  |  |  |
| Duration of illness (DOI) in years | 2.1 | 2.3 | 2 | 10.4 | 14.0 | | 5 | | 15.7 |  | 1 | 15.0 | 15.3 | 11 | 0.5 | 3 | 0.68 |  |
| Schizophrenia diagnosis (%) | 77.7 | 3.8 | 2 | 82.3 | 24.4 | | 5 | | 91.9 |  | 1 | 90.8 | 13.0 | 11 | 0.6 | 3 | 0.64 |  |
|  | n | % | N | n | % | | N | | n | % | N | n | % | N | χ^2^ | df | p | Subgroup differences |
| Antipsychotic use by all participants | 1 | 50.0% | 2 | 3 | 60.0% | | 5 | | 1 | 100.0% | 1 | 5 | 41.7% | 12 | 1.5 | 3 | 0.68 |  |
| Combined treatment of psychosocial and pharmacological therapy provided | 0 | 0.0% | 2 | 2 | 50.0% | | 4 | |  |  |  | 0 | 0.0% | 6 | 4.8 | 2 | 0.09 |  |
| Treatment provided focused on targeted outcomes. | 0 | 0.0% | 1 | 2 | 66.7% | | 3 | | 0 | 0.0% | 1 | 2 | 25.0% | 8 | 2.8 | 3 | 0.42 |  |
| **Symptoms** | M | SD | N | M | SD | | N | | M | SD | N | M | SD | N | F | Df | p | Subgroup differences |
| Depressive symptoms |  |  |  |  |  | |  | |  |  |  |  |  |  |  |  |  |  |
| Disorganization symptoms |  |  |  |  |  | |  | |  |  |  |  |  |  |  |  |  |  |
| Negative symptoms | 71.2 | 27.1 | 2 | 44.8 | 32.6 | | 5 | | 52.0 |  | 1 | 52.5 | 33.0 | 11 | 0.3 | 3 | 0.82 |  |
| Overall symptoms | 53.0 | 4.2 | 2 | 56.5 | 40.9 | | 4 | | 59.9 |  | 1 | 50.3 | 32.1 | 7 | 0.0 | 3 | 0.99 |  |
| Positive symptoms | 71.7 | 25.0 | 2 | 54.7 | 33.6 | | 5 | | 54.0 |  | 1 | 54.5 | 25.9 | 11 | 0.2 | 3 | 0.88 |  |
| **Functioning** | M | SD | N | M | SD | | N | | M | SD | N | M | SD | N | F | Df | p | Subgroup differences |
| Independence |  |  |  |  |  | |  | |  |  |  |  |  |  |  |  |  |  |
| Overall social functioning |  |  |  |  |  | |  | |  |  |  |  |  |  |  |  |  |  |
| Prosocial behavior |  |  |  |  |  | |  | |  |  |  |  |  |  |  |  |  |  |
| Vocational functioning |  |  |  |  |  | |  | |  |  |  |  |  |  |  |  |  |  |
| **Personal recovery and QOL** | M | SD | N | M | SD | | N | | M | SD | N | M | SD | N | F | Df | p | Subgroup differences |
| Personal recovery |  |  |  |  |  | |  | |  |  |  |  |  |  |  |  |  |  |
| Subjective quality of life |  |  |  |  |  | |  | |  |  |  |  |  |  |  |  |  |  |
| **Cognition** | M | SD | N | M | SD | | N | | M | SD | N | M | SD | N | F | Df | p | Subgroup differences |
| Executive functioning | 51.0 |  | 1 | 71.6 | 19.2 | | 4 | | 14.0 |  | 1 | 47.1 | 41.2 | 6 | 0.9 | 3 | 0.50 |  |
| Overall cognition* | 67.5 | 6.3 | 2 | 53.4 | 37.2 | | 4 | | 1.0 |  | 1 | 25.4 | 25.4 | 10 | 2.4 | 3 | 0.11 | 1>4 |
| Processing speed |  |  |  |  |  | |  | |  |  |  |  |  |  |  |  |  |  |
| Verbal memory* | 41.9 |  | 1 | 63.0 | 36.0 | | 3 | | 2.0 |  | 1 | 57.5 | 18.2 | 6 | 1.7 | 3 | 0.25 | 3<4 |
| **Study characteristics** | M | SD | N | M | SD | | N | | M | SD | N | M | SD | N | F | Df | p | Subgroup differences |
| Attrition rate* | 31.3 | 29.2 | 2 | 20.5 | 8.7 | | 5 | | 49.4 |  | 1 | 35.6 | 17.2 | 12 | 1.3 | 3 | 0.29 | 2<3 |
|  | n | % | N | n | % | | N | | n | % | N | n | % | N | χ^2^ | df | p | Subgroup differences |
| DOI subgroup overlap (yes vs no) | 0 | 0.0% | 1 | 1 | 25.0% | | 4 | | 1 | 100.0% | 1 | 6 | 66.7% | 9 | 4.0 | 4 | 0.27 |  |
| Publication year <10 years | 2 | 100.0% | 2 | 2 | 40.0% | | 5 | | 1 | 100.0% | 1 | 6 | 50.0% | 12 | 3.0 | 3 | 0.39 |  |
| Study design: clinical trial | 1 | 50.0% | 2 | 1 | 20.0% | | 5 | | 1 | 100.0% | 1 | 3 | 27.3% | 11 | 2.9 | 3 | 0.41 |  |
| **Quality assessment** | n | % | N | n | | % | | N | n | % | N | n | % | N | χ^2^ | df | p | Subgroup differences |
| Study participation: Low ROB | 2 | 100.0% | 2 | 2 | | 40.0% | | 5 | 0 | 0.0% | 1 | 3 | 25.0% | 12 | 4.8 | 3 | 0.18 |  |
| Study attrition: Low ROB | 1 | 50.0% | 2 | 3 | | 60.0% | | 5 | 0 | 0.0% | 1 | 2 | 16.7% | 12 | 5.4 | 3 | 0.50 |  |
| Prognostic Factor Measurement: Low ROB | 2 | 100.0% | 2 | 2 | | 40.0% | | 5 | 0 | 0.0% | 1 | 1 | 8.3% | 12 | 10.8 | 3 | 0.10 |  |
| Outcome measurement: Low ROB | 1 | 50.0% | 2 | 4 | | 80.0% | | 5 | 1 | 100.0% | 1 | 10 | 83.3% | 12 | 5.8 | 3 | 0.44 |  |
| Study confounding: Low ROB | 1 | 50.0% | 2 | 3 | | 60.0% | | 5 | 0 | 0.0% | 1 | 7 | 58.3% | 12 | 2.4 | 3 | 0.88 |  |
| Statistical analysis and reporting: Low ROB |  |  |  |  | |  | |  |  |  |  |  |  |  |  |  |  |  |
| **Depression and Processing speed** | | | | | | | | | | | | | | | | | | |
| **Subgroup:** | 1. both moderate-high improvement | | | 2. Only one moderate- high improvement | | | | | 3. Both marginal-no improvement | | | 4. No improvement or deterioration | | | **ANOVA** | | | |
| **Demographic variables** | M | SD | N | M | SD | | N | | M | SD | N | M | SD | N | F | Df | p | Subgroup differences |
| Age | 26.9 | 3.1 | 2 | 27.6 | 1.3 | | 2 | |  |  |  | 34.8 | 5.1 | 7 | 3.5 | 2 | 0.08 |  |
| Age at onset | 23.9 | 4.1 | 2 | 26.4 | 0.4 | | 2 | |  |  |  | 24.3 | 1.9 | 7 | 0.8 | 2 | 0.47 |  |
| Ethnicity: % Caucasian or born in country of residence |  |  |  |  |  | |  | |  |  |  |  |  |  |  |  |  |  |
| Female gender (%) | 33.7 | 11.7 | 2 | 37.5 | 4.1 | | 2 | |  |  |  | 37.2 | 18.7 | 7 | 0.0 | 2 | 0.96 |  |
| Marital status: % not married/in relationship |  |  |  |  |  | |  | |  |  |  |  |  |  |  |  |  |  |
|  | n | % | N | n | % | | N | | n | % | N | n | % | N | χ^2^ | df | p | Subgroup differences |
| Education level (high level) | 1 | 50.0% | 2 | 1 | 50.0% | | 2 | |  |  |  | 3 | 42.9% | 7 | 0.1 | 2 | 0.97 |  |
| **Clinical variables** | M | SD | N | M | SD | | N | | M | SD | N | M | SD | N | F | Df | p | Subgroup differences |
| Duration of untreated psychosis in weeks |  |  |  |  |  | |  | |  |  |  |  |  |  |  |  |  |  |
| Duration of illness (DOI) in years | 3.0 | 1.0 | 2 | 1.2 | 1.7 | | 2 | |  |  |  | 10.5 | 6.0 | 7 | 3.3 | 2 | 0.09 |  |
| Schizophrenia diagnosis (%) | 70.2 | 14.4 | 2 | 50.0 |  | | 1 | |  |  |  | 81.9 | 21.7 | 7 | 1.1 | 2 | 0.37 |  |
|  | n | % | N | n | % | | N | | n | % | N | n | % | N | χ^2^ | df | p | Subgroup differences |
| Antipsychotic use by all participants | 2 | 100.0% | 2 | 0 | 0.0% | | 2 | |  |  |  | 4 | 57.1% | 7 | 4.1 | 2 | 0.13 |  |
| Combined treatment of psychosocial and pharmacological therapy provided |  |  |  |  |  | |  | |  |  |  |  |  |  |  |  |  |  |
| Treatment provided focused on targeted outcomes. |  |  |  |  |  | |  | |  |  |  |  |  |  |  |  |  |  |
| **Symptoms** | M | SD | N | M | SD | | N | | M | SD | N | M | SD | N | F | Df | p | Subgroup differences |
| Depressive symptoms |  |  |  |  |  | |  | |  |  |  |  |  |  |  |  |  |  |
| Disorganization symptoms |  |  |  |  |  | |  | |  |  |  |  |  |  |  |  |  |  |
| Negative symptoms | 48.5 | 59.1 | 2 | 63.7 |  | | 1 | |  |  |  | 38.8 | 26.4 | 7 | 0.3 | 2 | 0.76 |  |
| Overall symptoms |  |  |  |  |  | |  | |  |  |  |  |  |  |  |  |  |  |
| Positive symptoms | 62.0 | 38.8 | 2 | 42.1 | 25.2 | | 2 | |  |  |  | 45.4 | 36.6 | 7 | 0.2 | 2 | 0.82 |  |
| **Functioning** | M | SD | N | M | SD | | N | | M | SD | N | M | SD | N | F | Df | p | Subgroup differences |
| Independence |  |  |  |  |  | |  | |  |  |  |  |  |  |  |  |  |  |
| Overall social functioning |  |  |  |  |  | |  | |  |  |  |  |  |  |  |  |  |  |
| Prosocial behavior |  |  |  |  |  | |  | |  |  |  |  |  |  |  |  |  |  |
| Vocational functioning |  |  |  |  |  | |  | |  |  |  |  |  |  |  |  |  |  |
| **Personal recovery and QOL** | M | SD | N | M | SD | | N | | M | SD | N | M | SD | N | F | Df | p | Subgroup differences |
| Personal recovery |  |  |  |  |  | |  | |  |  |  |  |  |  |  |  |  |  |
| Subjective quality of life |  |  |  |  |  | |  | |  |  |  |  |  |  |  |  |  |  |
| **Cognition** | M | SD | N | M | SD | | N | | M | SD | N | M | SD | N | F | Df | p | Subgroup differences |
| Executive functioning | 78.8 |  | 1 | 58.5 | 17.6 | | 2 | |  |  |  | 30.5 | 37.0 | 7 | 1.2 | 2 | 0.37 |  |
| Overall cognition |  |  |  |  |  | |  | |  |  |  |  |  |  |  |  |  |  |
| Processing speed | 22.7 |  | 1 | 59.8 | 8.1 | | 2 | |  |  |  | 42.0 | 36.2 | 7 | 0.4 | 2 | 0.67 |  |
| Verbal memory | 65.5 |  | 1 | 70.0 | 3.7 | | 2 | |  |  |  | 56.8 | 32.6 | 7 | 0.2 | 2 | 0.85 |  |
| **Study characteristics** | M | SD | N | M | SD | | N | | M | SD | N | M | SD | N | F | Df | p | Subgroup differences |
| Attrition rate | 39.8 | 17.3 | 2 | 33.2 | 7.4 | | 2 | |  |  |  | 30.4 | 15.4 | 7 | 0.3 | 2 | 0.74 |  |
|  | n | % | N | n | % | | N | | n | % | N | n | % | N | χ^2^ | df | p | Subgroup differences |
| DOI subgroup overlap (yes vs no) |  |  |  |  |  | |  | |  |  |  |  |  |  |  |  |  |  |
| Publication year <10 years | 1 | 50.0% | 2 | 1 | 50.0% | | 2 | |  |  |  | 3 | 42.9% | 7 | 0.1 | 2 | 0.97 |  |
| Study design: clinical trial | 1 | 50.0% | 2 | 0 | 0.0% | | 2 | |  |  |  | 2 | 28.6% | 7 | 1.3 | 2 | 0.53 |  |
| **Quality assessment** | n | % | N | n | | % | | N | n | % | N | n | % | N | χ^2^ | df | p | Subgroup differences |
| Study participation: Low ROB | 2 | 100.0% | 2 | 2 | | 100.0% | | 2 |  |  |  | 3 | 42.9% | 7 | 3.6 | 2 | 0.46 |  |
| Study attrition: Low ROB | 1 | 50.0% | 2 | 0 | | 0.0% | | 2 |  |  |  | 1 | 14.3% | 7 | 5.7 | 2 | 0.22 |  |
| Prognostic Factor Measurement: Low ROB | 1 | 50.0% | 2 | 1 | | 50.0% | | 2 |  |  |  | 1 | 14.3% | 7 | 4.2 | 2 | 0.38 |  |
| Outcome measurement: Low ROB | 1 | 50.0% | 2 | 2 | | 100.0% | | 2 |  |  |  | 6 | 85.7% | 7 | 1.9 | 2 | 0.39 |  |
| Study confounding: Low ROB | 1 | 50.0% | 2 | 2 | | 100.0% | | 2 |  |  |  | 3 | 42.9% | 7 | 3.0 | 2 | 0.56 |  |
| Statistical analysis and reporting: Low ROB |  |  |  |  | |  | |  |  |  |  |  |  |  |  |  |  |  |
| **Depression and Verbal memory** | | | | | | | | | | | | | | | | | | |
| **Subgroup:** | 1. both moderate-high improvement | | | 2. Only one moderate- high improvement | | | | | 3. Both marginal-no improvement | | | 4. No improvement or deterioration | | | **ANOVA** | | | |
| **Demographic variables** | M | SD | N | M | SD | | N | | M | SD | N | M | SD | N | F | Df | p | Subgroup differences |
| Age* |  |  |  | 28.2 | 2.6 | | 5 | |  |  |  | 34.7 | 5.9 | 9 | 5.4 | 1 | 0.04 | 2<4 |
| Age at onset |  |  |  | 26.2 | 3.3 | | 5 | |  |  |  | 24.1 | 1.8 | 9 | 2.3 | 1 | 0.16 |  |
| Ethnicity: % Caucasian or born in country of residence |  |  |  |  |  | |  | |  |  |  |  |  |  |  |  |  |  |
| Female gender (%) |  |  |  | 39.5 | 10.8 | | 5 | |  |  |  | 34.5 | 17.0 | 9 | 0.3 | 1 | 0.57 |  |
| Marital status: % not married/in relationship |  |  |  |  |  | |  | |  |  |  |  |  |  |  |  |  |  |
|  | n | % | N | n | % | | N | | n | % | N | n | % | N | χ^2^ | df | p | Subgroup differences |
| Education level (high level) |  |  |  | 2 | 40.0% | | 5 | |  |  |  | 5 | 62.5% | 8 | 0.6 | 1 | 0.59 |  |
| **Clinical variables** | M | SD | N | M | SD | | N | | M | SD | N | M | SD | N | F | Df | p | Subgroup differences |
| Duration of untreated psychosis in weeks |  |  |  |  |  | |  | |  |  |  |  |  |  |  |  |  |  |
| Duration of illness (DOI) in years* |  |  |  | 2.0 | 1.4 | | 5 | |  |  |  | 10.5 | 6.9 | 9 | 7.2 | 1 | 0.02 | 2<4 |
| Schizophrenia diagnosis (%) |  |  |  | 67.8 | 15.3 | | 4 | |  |  |  | 85.9 | 20.4 | 9 | 2.5 | 1 | 0.14 |  |
|  | n | % | N | n | % | | N | | n | % | N | n | % | N | χ^2^ | df | p | Subgroup differences |
| Antipsychotic use by all participants |  |  |  | 2 | 40.0% | | 5 | |  |  |  | 5 | 55.6% | 9 | 0.3 | 1 | 0.50 |  |
| Combined treatment of psychosocial and pharmacological therapy provided |  |  |  |  |  | |  | |  |  |  |  |  |  |  |  |  |  |
| Treatment provided focused on targeted outcomes. |  |  |  |  |  | |  | |  |  |  |  |  |  |  |  |  |  |
| **Symptoms** | M | SD | N | M | SD | | N | | M | SD | N | M | SD | N | F | Df | p | Subgroup differences |
| Depressive symptoms |  |  |  | 62.4 | 13.4 | | 4 | |  |  |  | 60.3 | 15.2 | 7 | 0.0 | 1 | 0.83 |  |
| Disorganization symptoms |  |  |  |  |  | |  | |  |  |  |  |  |  |  |  |  |  |
| Negative symptoms |  |  |  | 62.5 | 39.2 | | 4 | |  |  |  | 37.4 | 24.8 | 8 | 1.9 | 1 | 0.20 |  |
| Overall symptoms |  |  |  |  |  | |  | |  |  |  |  |  |  |  |  |  |  |
| Positive symptoms |  |  |  | 49.6 | 25.8 | | 5 | |  |  |  | 43.4 | 34.3 | 8 | 0.1 | 1 | 0.73 |  |
| **Functioning** | M | SD | N | M | SD | | N | | M | SD | N | M | SD | N | F | Df | p | Subgroup differences |
| Independence |  |  |  |  |  | |  | |  |  |  |  |  |  |  |  |  |  |
| Overall social functioning |  |  |  |  |  | |  | |  |  |  |  |  |  |  |  |  |  |
| Prosocial behavior |  |  |  |  |  | |  | |  |  |  |  |  |  |  |  |  |  |
| Vocational functioning |  |  |  |  |  | |  | |  |  |  |  |  |  |  |  |  |  |
| **Personal recovery and QOL** | M | SD | N | M | SD | | N | | M | SD | N | M | SD | N | F | Df | p | Subgroup differences |
| Personal recovery |  |  |  |  |  | |  | |  |  |  |  |  |  |  |  |  |  |
| Subjective quality of life |  |  |  |  |  | |  | |  |  |  |  |  |  |  |  |  |  |
| **Cognition** | M | SD | N | M | SD | | N | | M | SD | N | M | SD | N | F | Df | p | Subgroup differences |
| Executive functioning |  |  |  | 64.9 | 14.0 | | 4 | |  |  |  | 36.9 | 38.7 | 8 | 1.9 | 1 | 0.20 |  |
| Overall cognition* |  |  |  | 88.2 | 14.6 | | 3 | |  |  |  | 28.4 | 31.3 | 7 | 9.5 | 1 | 0.02 | 2>4 |
| Processing speed |  |  |  | 47.4 | 22.2 | | 3 | |  |  |  | 42.0 | 36.2 | 7 | 0.1 | 1 | 0.82 |  |
| Verbal memory |  |  |  | 72.1 | 7.8 | | 4 | |  |  |  | 58.1 | 30.4 | 8 | 0.8 | 1 | 0.40 |  |
| **Study characteristics** | M | SD | N | M | SD | | N | | M | SD | N | M | SD | N | F | Df | p | Subgroup differences |
| Attrition rate |  |  |  | 37.0 | 10.0 | | 5 | |  |  |  | 32.3 | 17.0 | 9 | 0.3 | 1 | 0.59 |  |
|  | n | % | N | n | % | | N | | n | % | N | n | % | N | χ^2^ | df | p | Subgroup differences |
| DOI subgroup overlap (yes vs no) |  |  |  | 2 | 50.0% | | 4 | |  |  |  | 4 | 57.1% | 7 | 0.1 | 1 | 0.65 |  |
| Publication year <10 years |  |  |  | 2 | 40.0% | | 5 | |  |  |  | 5 | 55.6% | 9 | 0.3 | 1 | 0.50 |  |
| Study design: clinical trial |  |  |  | 1 | 20.0% | | 5 | |  |  |  | 2 | 25.0% | 8 | 0.0 | 1 | 0.69 |  |
| **Quality assessment** | n | % | N | n | | % | | N | n | % | N | n | % | N | χ^2^ | df | p | Subgroup differences |
| Study participation: Low ROB |  |  |  | 4 | | 80.0% | | 5 |  |  |  | 4 | 44.4% | 9 | 1.8 | 1 | 0.41 |  |
| Study attrition: Low ROB |  |  |  | 1 | | 20.0% | | 5 |  |  |  | 2 | 22.2% | 9 | 2.2 | 1 | 0.33 |  |
| Prognostic Factor Measurement: Low ROB |  |  |  | 2 | | 40.0% | | 5 |  |  |  | 2 | 22.2% | 9 | 0.9 | 1 | 0.63 |  |
| Outcome measurement: Low ROB |  |  |  | 4 | | 80.0% | | 5 |  |  |  | 8 | 88.9% | 9 | 0.2 | 1 | 0.65 |  |
| Study confounding: Low ROB |  |  |  | 4 | | 80.0% | | 5 |  |  |  | 4 | 44.4% | 9 | 2.0 | 1 | 0.36 |  |
| Statistical analysis and reporting: Low ROB |  |  |  | 4 | | 80.0% | | 5 |  |  |  | 8 | 88.9% | 9 | 0.2 | 1 | 0.65 |  |

**Symptoms and PR/QOL associations**

| **Negative symptoms and Subjective quality of life** | | | | | | | | | | | | | | | | | | |
| --- | --- | --- | --- | --- | --- | --- | --- | --- | --- | --- | --- | --- | --- | --- | --- | --- | --- | --- |
| **Subgroup**:** | 1. both moderate-high improvement | | | 2. Only one moderate- high improvement | | | | | 3. Both marginal-no improvement | | | 4. No improvement or deterioration | | | **ANOVA** | | | |
| **Demographic variables** | M | SD | N | M | SD | | N | | M | SD | N | M | SD | N | F | Df | p | Subgroup differences |
| Age | 31.5 | 8.9 | 3 | 38.9 | 11.7 | | 5 | | 39.9 | 7.7 | 3 | 36.5 | 10.0 | 9 | 0.4 | 3 | 0.73 |  |
| Age at onset | 24.2 | 4.6 | 3 | 22.1 | 1.7 | | 5 | | 24.2 | 5.3 | 2 | 22.1 | 1.9 | 7 | 0.7 | 3 | 0.59 |  |
| Ethnicity: % Caucasian or born in country of residence |  |  |  |  |  | |  | |  |  |  |  |  |  |  |  |  |  |
| Female gender (%) | 36.3 | 14.6 | 3 | 43.2 | 16.9 | | 5 | | 25.0 | 6.1 | 3 | 34.2 | 8.0 | 9 | 1.6 | 3 | 0.23 |  |
| Marital status: % not married/in relationship | 79.8 | 6.6 | 3 | 79.5 |  | | 1 | | 77.3 | 12.5 | 2 | 79.8 | 10.7 | 5 | 0.0 | 3 | 0.99 |  |
|  | n | % | N | n | % | | N | | n | % | N | n | % | N | χ^2^ | df | p | Subgroup differences |
| Education level (high level) | 1 | 33.3% | 3 | 1 | 33.3% | | 3 | | 2 | 100.0% | 2 | 3 | 50.0% | 6 | 2.7 | 3 | 0.45 |  |
| **Clinical variables** | M | SD | N | M | SD | | N | | M | SD | N | M | SD | N | F | Df | p | Subgroup differences |
| Duration of untreated psychosis in weeks |  |  |  |  |  | |  | |  |  |  |  |  |  |  |  |  |  |
| Duration of illness (DOI) in years | 7.3 | 7.8 | 3 | 16.8 | 11.0 | | 5 | | 11.8 | 0.3 | 2 | 12.0 | 9.1 | 9 | 0.7 | 3 | 0.57 |  |
| Schizophrenia diagnosis (%)* | 76.5 | 33.3 | 2 | 97.3 | 4.7 | | 3 | | 58.5 | 16.9 | 2 | 76.8 | 22.9 | 7 | 1.4 | 3 | 0.31 | 2>3 |
|  | n | % | N | n | % | | N | | n | % | N | n | % | N | χ^2^ | df | p | Subgroup differences |
| Antipsychotic use by all participants | 1 | 33.3% | 3 | 3 | 60.0% | | 5 | | 1 | 33.3% | 3 | 4 | 44.4% | 9 | 0.8 | 3 | 0.85 |  |
| Combined treatment of psychosocial and pharmacological therapy provided | 1 | 33.3% | 3 | 1 | 50.0% | | 2 | | 2 | 100.0% | 2 | 1 | 20.0% | 5 | 3.9 | 3 | 0.27 |  |
| Treatment provided focused on targeted outcomes. | 1 | 33.3% | 3 | 0 | 0.0% | | 3 | | 1 | 33.3% | 3 | 2 | 25.0% | 8 | 1.3 | 3 | 0.74 |  |
| **Symptoms** | M | SD | N | M | SD | | N | | M | SD | N | M | SD | N | F | Df | p | Subgroup differences |
| Depressive symptoms | 44.0 |  | 1 | 74.2 | 23.2 | | 3 | | 46.5 | 41.5 | 2 | 48.1 | 14.6 | 4 | 0.9 | 3 | 0.49 |  |
| Disorganization symptoms |  |  |  |  |  | |  | |  |  |  |  |  |  |  |  |  |  |
| Negative symptoms | 77.0 | 15.1 | 3 | 58.2 | 26.4 | | 5 | | 57.2 | 25.3 | 3 | 56.2 | 31.9 | 9 | 0.4 | 3 | 0.73 |  |
| Overall symptoms | 68.6 | 16.4 | 3 | 66.6 | 29.0 | | 5 | | 51.0 | 7.1 | 2 | 55.9 | 37.3 | 5 | 0.3 | 3 | 0.86 |  |
| Positive symptoms* | 60.8 | 24.9 | 3 | 76.4 | 16.1 | | 5 | | 45.8 | 15.4 | 3 | 58.6 | 37.8 | 8 | 0.8 | 3 | 0.54 | 2>3 |
| **Functioning** | M | SD | N | M | SD | | N | | M | SD | N | M | SD | N | F | Df | p | Subgroup differences |
| Independence |  |  |  |  |  | |  | |  |  |  |  |  |  |  |  |  |  |
| Overall social functioning | 49.8 |  | 1 | 56.9 | 9.9 | | 3 | |  |  |  | 57.7 | 24.7 | 6 | 0.1 | 2 | 0.94 |  |
| Prosocial behavior |  |  |  |  |  | |  | |  |  |  |  |  |  |  |  |  |  |
| Vocational functioning |  |  |  |  |  | |  | |  |  |  |  |  |  |  |  |  |  |
| **Personal recovery and QOL** | M | SD | N | M | SD | | N | | M | SD | N | M | SD | N | F | Df | p | Subgroup differences |
| Personal recovery | 46.0 |  | 1 | 35.0 | 23.4 | | 2 | | 46.6 | 14.5 | 2 | 62.5 | 23.6 | 5 | 0.8 | 3 | 0.53 |  |
| Subjective quality of life* | 24.3 | 14.5 | 3 | 32.7 | 15.5 | | 5 | | 51.5 | 35.2 | 3 | 59.1 | 15.2 | 9 | 3.7 | 3 | 0.03 | 1<4 / 2<4 |
| **Cognition** | M | SD | N | M | SD | | N | | M | SD | N | M | SD | N | F | Df | p | Subgroup differences |
| Executive functioning |  |  |  |  |  | |  | |  |  |  |  |  |  |  |  |  |  |
| Overall cognition |  |  |  |  |  | |  | |  |  |  |  |  |  |  |  |  |  |
| Processing speed |  |  |  |  |  | |  | |  |  |  |  |  |  |  |  |  |  |
| Verbal memory |  |  |  |  |  | |  | |  |  |  |  |  |  |  |  |  |  |
| **Study characteristics** | M | SD | N | M | SD | | N | | M | SD | N | M | SD | N | F | Df | p | Subgroup differences |
| Attrition rate | 48.8 | 7.1 | 2 | 32.3 | 12.4 | | 5 | | 27.3 | 11.9 | 3 | 30.2 | 22.1 | 9 | 0.7 | 3 | 0.58 |  |
|  | n | % | N | n | % | | N | | n | % | N | n | % | N | χ^2^ | df | p | Subgroup differences |
| DOI subgroup overlap (yes vs no) | 2 | 100.0% | 2 | 2 | 50.0% | | 4 | | 2 | 100.0% | 2 | 3 | 42.9% | 7 | 3.7 | 3 | 0.30 |  |
| Publication year <10 years | 3 | 100.0% | 3 | 3 | 60.0% | | 5 | | 2 | 66.7% | 3 | 5 | 55.6% | 9 | 2.0 | 3 | 0.57 |  |
| Study design: clinical trial | 1 | 33.3% | 3 | 3 | 60.0% | | 5 | | 3 | 100.0% | 3 | 5 | 55.6% | 9 | 2.9 | 3 | 0.40 |  |
| **Quality assessment** | n | % | N | n | | % | | N | n | % | N | n | % | N | χ^2^ | df | p | Subgroup differences |
| Study participation: Low ROB | 2 | 66.7% | 3 | 1 | | 20.0% | | 5 | 2 | 66.7% | 3 | 6 | 66.7% | 9 | 3.3 | 3 | 0.35 |  |
| Study attrition: Low ROB | 1 | 33.3% | 3 | 1 | | 20.0% | | 5 | 1 | 33.3% | 3 | 4 | 44.4% | 9 | 3.0 | 3 | 0.81 |  |
| Prognostic Factor Measurement: Low ROB | 1 | 33.3% | 3 | 0 | | 0.0% | | 5 | 2 | 66.7% | 3 | 6 | 66.7% | 9 | 10.2 | 3 | 0.12 |  |
| Outcome measurement: Low ROB* | 1 | 33.3% | 3 | 5 | | 100.0% | | 5 | 3 | 100.0% | 3 | 9 | 100.0% | 9 | 12.6 | 3 | 0.01 | 1<2;4 |
| Study confounding: Low ROB | 1 | 33.3% | 3 | 3 | | 60.0% | | 5 | 2 | 66.7% | 3 | 3 | 33.3% | 9 | 3.2 | 3 | 0.78 |  |
| Statistical analysis and reporting: Low ROB | 3 | 100.0% | 3 | 5 | | 100.0% | | 5 | 3 | 100.0% | 3 | 5 | 55.6% | 9 | 6.1 | 3 | 0.41 |  |
| **Positive symptoms and Subjective quality of life** | | | | | | | | | | | | | | | | | | |
| **Subgroup**:** | 1. both moderate-high improvement | | | 2. Only one moderate- high improvement | | | | | 3. Both marginal-no improvement | | | 4. No improvement or deterioration | | | **ANOVA** | | | |
| **Demographic variables** | M | SD | N | M | SD | | N | | M | SD | N | M | SD | N | F | Df | p | Subgroup differences |
| Age | 30.1 | 7.3 | 5 | 37.0 | 10.0 | | 4 | | 39.9 | 7.7 | 3 | 39.6 | 11.5 | 7 | 1.1 | 3 | 0.39 |  |
| Age at onset | 22.8 | 3.9 | 5 | 22.2 | 1.6 | | 4 | | 24.2 | 5.3 | 2 | 23.0 | 1.7 | 5 | 0.2 | 3 | 0.89 |  |
| Ethnicity: % Caucasian or born in country of residence |  |  |  |  |  | |  | |  |  |  |  |  |  |  |  |  |  |
| Female gender (%) | 38.1 | 10.7 | 5 | 40.1 | 21.8 | | 4 | | 25.0 | 6.1 | 3 | 34.8 | 8.0 | 7 | 1.0 | 3 | 0.43 |  |
| Marital status: % not married/in relationship | 79.8 | 6.6 | 3 |  |  | |  | | 77.3 | 12.5 | 2 | 83.4 | 3.9 | 5 | 0.7 | 2 | 0.53 |  |
|  | n | % | N | n | % | | N | | n | % | N | n | % | N | χ^2^ | df | p | Subgroup differences |
| Education level (high level) | 1 | 25.0% | 4 | 2 | 100.0% | | 2 | | 2 | 100.0% | 2 | 1 | 20.0% | 5 | 6.8 | 3 | 0.08 |  |
| **Clinical variables** | M | SD | N | M | SD | | N | | M | SD | N | M | SD | N | F | Df | p | Subgroup differences |
| Duration of untreated psychosis in weeks |  |  |  |  |  | |  | |  |  |  |  |  |  |  |  |  |  |
| Duration of illness (DOI) in years* | 7.3 | 6.7 | 5 | 19.4 | 2.7 | | 4 | | 11.8 | 0.3 | 2 | 11.2 | 11.7 | 7 | 1.5 | 3 | 0.26 | 1<2 / 2>3 |
| Schizophrenia diagnosis (%) | 76.5 | 33.3 | 2 | 89.5 | 16.0 | | 4 | | 58.5 | 16.9 | 2 | 82.9 | 24.8 | 5 | 0.9 | 3 | 0.49 |  |
|  | n | % | N | n | % | | N | | n | % | N | n | % | N | χ^2^ | df | p | Subgroup differences |
| Antipsychotic use by all participants | 2 | 40.0% | 5 | 3 | 75.0% | | 4 | | 1 | 33.3% | 3 | 3 | 42.9% | 7 | 1.6 | 3 | 0.65 |  |
| Combined treatment of psychosocial and pharmacological therapy provided | 1 | 33.3% | 3 | 1 | 33.3% | | 3 | | 2 | 100.0% | 2 | 1 | 33.3% | 3 | 2.9 | 3 | 0.40 |  |
| Treatment provided focused on targeted outcomes. | 1 | 25.0% | 4 | 1 | 33.3% | | 3 | | 1 | 33.3% | 3 | 1 | 16.% | 6 | 0.44 | 3 | 0.93 |  |
| **Symptoms** | M | SD | N | M | SD | | N | | M | SD | N | M | SD | N | F | Df | p | Subgroup differences |
| Depressive symptoms |  |  |  |  |  | |  | |  |  |  |  |  |  |  |  |  |  |
| Disorganization symptoms |  |  |  |  |  | |  | |  |  |  |  |  |  |  |  |  |  |
| Negative symptoms | 67.6 | 29.9 | 5 | 54.7 | 22.3 | | 4 | | 57.2 | 25.3 | 3 | 60.7 | 33.5 | 7 | 0.2 | 3 | 0.92 |  |
| Overall symptoms | 73.2 | 18.5 | 5 | 44.5 | 25.7 | | 4 | | 51.0 | 7.1 | 2 | 68.6 | 38.8 | 4 | 1.1 | 3 | 0.41 |  |
| Positive symptoms | 65.5 | 18.9 | 5 | 58.6 | 24.2 | | 4 | | 45.8 | 15.4 | 3 | 67.3 | 40.7 | 7 | 0.4 | 3 | 0.76 |  |
| **Functioning** | M | SD | N | M | SD | | N | | M | SD | N | M | SD | N | F | Df | p | Subgroup differences |
| Independence |  |  |  |  |  | |  | |  |  |  |  |  |  |  |  |  |  |
| Overall social functioning |  |  |  |  |  | |  | |  |  |  |  |  |  |  |  |  |  |
| Prosocial behavior |  |  |  |  |  | |  | |  |  |  |  |  |  |  |  |  |  |
| Vocational functioning |  |  |  |  |  | |  | |  |  |  |  |  |  |  |  |  |  |
| **Personal recovery and QOL** | M | SD | N | M | SD | | N | | M | SD | N | M | SD | N | F | Df | p | Subgroup differences |
| Personal recovery |  |  |  |  |  | |  | |  |  |  |  |  |  |  |  |  |  |
| Subjective quality of life* | 22.6 | 10.7 | 5 | 52.1 | 22.6 | | 4 | | 51.5 | 35.2 | 3 | 57.0 | 14.9 | 7 | 3.3 | 3 | 0.05 | 1<2;4 |
| **Cognition** | M | SD | N | M | SD | | N | | M | SD | N | M | SD | N | F | Df | p | Subgroup differences |
| Executive functioning |  |  |  |  |  | |  | |  |  |  |  |  |  |  |  |  |  |
| Overall cognition |  |  |  |  |  | |  | |  |  |  |  |  |  |  |  |  |  |
| Processing speed |  |  |  |  |  | |  | |  |  |  |  |  |  |  |  |  |  |
| Verbal memory |  |  |  |  |  | |  | |  |  |  |  |  |  |  |  |  |  |
| **Study characteristics** | M | SD | N | M | SD | | N | | M | SD | N | M | SD | N | F | Df | p | Subgroup differences |
| Attrition rate | 39.6 | 14.2 | 4 | 40.3 | 23.5 | | 4 | | 27.3 | 11.9 | 3 | 29.6 | 16.3 | 7 | 0.6 | 3 | 0.61 |  |
|  | n | % | N | n | % | | N | | n | % | N | n | % | N | χ^2^ | df | p | Subgroup differences |
| DOI subgroup overlap (yes vs no) | 3 | 100.0% | 3 | 1 | 25.0% | | 4 | | 2 | 100.0% | 2 | 2 | 40.0% | 5 | 6.0 | 3 | 0.11 |  |
| Publication year <10 years | 5 | 100.0% | 5 | 3 | 75.0% | | 4 | | 2 | 66.7% | 3 | 3 | 42.9% | 7 | 4.5 | 3 | 0.21 |  |
| Study design: clinical trial | 2 | 40.0% | 5 | 3 | 75.0% | | 4 | | 3 | 100.0% | 3 | 4 | 57.1% | 7 | 3.3 | 3 | 0.35 |  |
| **Quality assessment** | n | % | N | n | | % | | N | n | % | N | n | % | N | χ^2^ | df | p | Subgroup differences |
| Study participation: Low ROB | 3 | 60.0% | 5 | 2 | | 50.0% | | 4 | 2 | 66.7% | 3 | 3 | 42.9% | 7 | 0.6 | 3 | 0.89 |  |
| Study attrition: Low ROB | 2 | 40.0% | 5 | 1 | | 25.0% | | 4 | 1 | 33.3% | 3 | 2 | 28.6% | 7 | 5.7 | 3 | 0.46 |  |
| Prognostic Factor Measurement: Low ROB | 1 | 20.0% | 5 | 1 | | 25.0% | | 4 | 2 | 66.7% | 3 | 4 | 57.1% | 7 | 4.0 | 3 | 0.68 |  |
| Outcome measurement: Low ROB | 3 | 60.0% | 5 | 4 | | 100.0% | | 4 | 3 | 100.0% | 3 | 7 | 100.0% | 7 | 6.3 | 3 | 0.10 |  |
| Study confounding: Low ROB | 2 | 40.0% | 5 | 1 | | 25.0% | | 4 | 2 | 66.7% | 3 | 4 | 57.1% | 7 | 5.3 | 3 | 0.50 |  |
| Statistical analysis and reporting: Low ROB | 5 | 100.0% | 5 | 3 | | 75.0% | | 4 | 3 | 100.0% | 3 | 4 | 57.1% | 7 | 4.8 | 3 | 0.57 |  |
| **Positive symptoms and Personal recovery** | | | | | | | | | | | | | | | | | | |
| **Subgroup**:** | 1. both moderate-high improvement | | | 2. Only one moderate- high improvement | | | | | 3. Both marginal-no improvement | | | 4. No improvement or deterioration | | | **ANOVA** | | | |
| **Demographic variables** | M | SD | N | M | SD | | N | | M | SD | N | M | SD | N | F | Df | p | Subgroup differences |
| Age | 29.0 | 10.3 | 3 | 29.1 | 9.9 | | 6 | | 36.3 | 2.3 | 3 | 38.6 | 9.8 | 11 | 1.7 | 3 | 0.19 |  |
| Age at onset | 21.6 |  | 1 | 21.9 | 3.2 | | 4 | | 25.2 | 1.5 | 3 | 23.2 | 2.3 | 7 | 1.2 | 3 | 0.36 |  |
| Ethnicity: % Caucasian or born in country of residence | 91.9 |  | 1 | 60.3 | 18.9 | | 4 | | 72.0 |  | 1 | 69.9 | 26.6 | 4 | 0.5 | 3 | 0.68 |  |
| Female gender (%)* | 37.9 | 4.4 | 3 | 28.2 | 4.8 | | 6 | | 38.4 | 10.0 | 3 | 36.8 | 10.5 | 11 | 1.6 | 3 | 0.22 | 1>2 |
| Marital status: % not married/in relationship |  |  |  |  |  | |  | |  |  |  |  |  |  |  |  |  |  |
|  | n | % | N | n | % | | N | | n | % | N | n | % | N | χ^2^ | df | p | Subgroup differences |
| Education level (high level) | 1 | 100.0% | 1 | 1 | 33.3% | | 3 | |  |  |  | 6 | 60.0% | 10 | 1.5 | 2 | 0.48 |  |
| **Clinical variables** | M | SD | N | M | SD | | N | | M | SD | N | M | SD | N | F | Df | p | Subgroup differences |
| Duration of untreated psychosis in weeks |  |  |  |  |  | |  | |  |  |  |  |  |  |  |  |  |  |
| Duration of illness (DOI) in years* | 3.5 | 2.1 | 2 | 37.7 | 68.8 | | 5 | | 11.1 | 2.5 | 3 | 12.7 | 10.4 | 8 | 0.6 | 3 | 0.60 | 1<3 |
| Schizophrenia diagnosis (%) | 78.7 | 6.6 | 2 | 76.1 | 17.9 | | 5 | | 95.0 | 8.7 | 3 | 76.7 | 21.7 | 9 | 0.8 | 3 | 0.51 |  |
|  | n | % | N | n | % | | N | | n | % | N | n | % | N | χ^2^ | df | p | Subgroup differences |
| Antipsychotic use by all participants | 1 | 33.3% | 3 | 2 | 33.3% | | 6 | | 1 | 33.3% | 3 | 3 | 27.3% | 11 | 0.1 | 3 | 0.99 |  |
| Combined treatment of psychosocial and pharmacological therapy provided | 2 | 66.7% | 3 |  |  | |  | | 0 | 0.0% | 1 | 5 | 83.3% | 6 | 2.9 | 2 | 0.24 |  |
| Treatment provided focused on targeted outcomes. | 1 | 50.0% | 2 | 3 | 75.0% | | 4 | | 0 | 0.0% | 1 | 5 | 55.6% | 9 | 1.9 | 3 | 0.60 |  |
| **Symptoms** | M | SD | N | M | SD | | N | | M | SD | N | M | SD | N | F | Df | p | Subgroup differences |
| Depressive symptoms* | 99.5 |  | 1 | 39.0 | 28.8 | | 5 | | 74.1 | 17.4 | 2 | 30.1 | 14.2 | 4 | 3.6 | 3 | 0.07 | 1>4 / 3>4 |
| Disorganization symptoms |  |  |  |  |  | |  | |  |  |  |  |  |  |  |  |  |  |
| Negative symptoms | 36.9 | 17.8 | 3 | 67.5 | 25.3 | | 6 | | 94.5 |  | 1 | 64.4 | 27.5 | 10 | 1.6 | 3 | 0.23 |  |
| Overall symptoms | 61.8 | 0.0 | 2 | 60.0 | 10.2 | | 5 | |  |  |  | 70.4 | 21.9 | 9 | 0.6 | 2 | 0.56 |  |
| Positive symptoms | 56.6 | 17.3 | 3 | 64.1 | 21.3 | | 6 | | 64.9 | 29.2 | 3 | 57.3 | 36.2 | 10 | 0.1 | 3 | 0.96 |  |
| **Functioning** | M | SD | N | M | SD | | N | | M | SD | N | M | SD | N | F | Df | p | Subgroup differences |
| Independence |  |  |  |  |  | |  | |  |  |  |  |  |  |  |  |  |  |
| Overall social functioning | 47.6 | 12.1 | 2 | 47.8 |  | | 1 | | 44.7 |  | 1 | 55.3 | 24.8 | 7 | 0.1 | 3 | 0.95 |  |
| Prosocial behavior |  |  |  |  |  | |  | |  |  |  |  |  |  |  |  |  |  |
| Vocational functioning |  |  |  | 28.9 | 9.5 | | 4 | |  |  |  | 39.5 | 28.0 | 8 | 0.5 | 1 | 0.49 |  |
| **Personal recovery and QOL** | M | SD | N | M | SD | | N | | M | SD | N | M | SD | N | F | Df | p | Subgroup differences |
| Personal recovery | 25.3 | 7.5 | 3 | 45.8 | 13.5 | | 5 | | 38.2 |  | 1 | 55.5 | 23.4 | 10 | 1.9 | 3 | 0.17 |  |
| Subjective quality of life* | 17.1 |  | 1 | 45.3 | 12.9 | | 3 | | 43.8 |  | 1 | 59.3 | 14.8 | 6 | 2.8 | 3 | 0.12 | 1<4 |
| **Cognition** | M | SD | N | M | SD | | N | | M | SD | N | M | SD | N | F | Df | p | Subgroup differences |
| Executive functioning |  |  |  |  |  | |  | |  |  |  |  |  |  |  |  |  |  |
| Overall cognition |  |  |  |  |  | |  | |  |  |  |  |  |  |  |  |  |  |
| Processing speed |  |  |  |  |  | |  | |  |  |  |  |  |  |  |  |  |  |
| Verbal memory |  |  |  |  |  | |  | |  |  |  |  |  |  |  |  |  |  |
| **Study characteristics** | M | SD | N | M | SD | | N | | M | SD | N | M | SD | N | F | Df | p | Subgroup differences |
| Attrition rate | 31.5 | 19.7 | 3 | 31.9 | 24.3 | | 5 | | 21.6 | 7.2 | 3 | 23.1 | 11.4 | 11 | 0.5 | 3 | 0.66 |  |
|  | n | % | N | n | % | | N | | n | % | N | n | % | N | χ^2^ | df | p | Subgroup differences |
| DOI subgroup overlap (yes vs no) | 0 | 0.0% | 1 | 1 | 50.0% | | 2 | | 3 | 100.0% | 3 | 4 | 66.7% | 6 | 3.8 | 3 | 0.29 |  |
| Publication year <10 years | 2 | 66.7% | 3 | 5 | 83.3% | | 6 | | 1 | 33.3% | 3 | 8 | 72.7% | 11 | 2.5 | 3 | 0.48 |  |
| Study design: clinical trial | 2 | 66.7% | 3 | 4 | 66.7% | | 6 | | 0 | 0.0% | 3 | 7 | 63.6% | 11 | 4.5 | 3 | 0.21 |  |
| **Quality assessment** | n | % | N | n | | % | | N | n | % | N | n | % | N | χ^2^ | df | p | Subgroup differences |
| Study participation: Low ROB | 2 | 66.7% | 3 | 4 | | 66.7% | | 6 | 0 | 0.0% | 3 | 8 | 72.7% | 11 | 5.4 | 3 | 0.14 |  |
| Study attrition: Low ROB | 2 | 66.7% | 3 | 2 | | 33.3% | | 6 | 2 | 66.7% | 3 | 5 | 45.5% | 11 | 2.7 | 3 | 0.85 |  |
| Prognostic Factor Measurement: Low ROB | 0 | 0.0% | 3 | 4 | | 66.7% | | 6 | 1 | 33.3% | 3 | 4 | 36.4% | 11 | 5.7 | 3 | 0.45 |  |
| Outcome measurement: Low ROB | 3 | 100.0% | 3 | 5 | | 83.3% | | 6 | 2 | 66.7% | 3 | 10 | 90.9% | 11 | 1.8 | 3 | 0.62 |  |
| Study confounding: Low ROB | 3 | 100.0% | 3 | 2 | | 33.3% | | 6 | 2 | 66.7% | 3 | 5 | 45.5% | 11 | 7.0 | 3 | 0.32 |  |
| Statistical analysis and reporting: Low ROB | 3 | 100.0% | 3 | 5 | | 83.3% | | 6 | 2 | 66.7% | 3 | 10 | 90.9% | 11 | 1.8 | 3 | 0.62 |  |

**Social functioning and cognition associations**

| **Overall social functioning and Executive functioning** | | | | | | | | | | | | | | | | |
| --- | --- | --- | --- | --- | --- | --- | --- | --- | --- | --- | --- | --- | --- | --- | --- | --- |
| **Subgroup**:** | 1. both moderate-high improvement | | | 2. Only one moderate- high improvement | | | 3. Both marginal-no improvement | | | 4. No improvement or deterioration | | | **ANOVA** | | | |
| **Demographic variables** | M | SD | N | M | SD | N | M | SD | N | M | SD | N | F | Df | p | Subgroup differences |
| Age* |  |  |  | 27.2 | 1.9 | 4 | 26.3 |  | 1 | 39.5 | 9.0 | 7 | 4.2 | 2 | 0.05 | 2<4 |
| Age at onset |  |  |  | 25.6 | 3.1 | 4 | 25.0 |  | 1 | 23.8 | 1.5 | 6 | 0.8 | 2 | 0.49 |  |
| Ethnicity: % Caucasian or born in country of residence |  |  |  |  |  |  |  |  |  |  |  |  |  |  |  |  |
| Female gender (%) |  |  |  | 37.6 | 8.1 | 4 | 26.2 |  | 1 | 27.0 | 8.0 | 7 | 2.3 | 2 | 0.15 |  |
| Marital status: % not married/in relationship |  |  |  |  |  |  |  |  |  |  |  |  |  |  |  |  |
|  | n | % | N | n | % | N | n | % | N | n | % | N | χ^2^ | df | p | Subgroup differences |
| Education level (high level) |  |  |  | 3 | 75.0% | 4 |  |  |  | 3 | 42.7% | 7 | 1.1 | 1 | 0.55 |  |
| **Clinical variables** | M | SD | N | M | SD | N | M | SD | N | M | SD | N | F | Df | p | Subgroup differences |
| Duration of untreated psychosis in weeks |  |  |  |  |  |  |  |  |  |  |  |  |  |  |  |  |
| Duration of illness (DOI) in years* |  |  |  | 1.5 | 1.8 | 4 | 1.3 |  | 1 | 13.2 | 9.8 | 7 | 3.1 | 2 | 0.10 | 2<4 |
| Schizophrenia diagnosis (%)* |  |  |  | 60.7 | 13.8 | 4 | 100.0 |  | 1 | 90.8 | 14.5 | 6 | 6.4 | 2 | 0.02 | 2<4 |
|  | n | % | N | n | % | N | n | % | N | n | % | N | χ^2^ | df | p | Subgroup differences |
| Antipsychotic use by all participants |  |  |  | 2 | 50.0% | 4 | 0 | 0.0% | 1 | 2 | 28.6% | 7 | 1.1 | 1 | 0.59 |  |
| Combined treatment of psychosocial and pharmacological therapy provided |  |  |  |  |  |  |  |  |  |  |  |  |  |  |  |  |
| Treatment provided focused on targeted outcomes. |  |  |  |  |  |  |  |  |  |  |  |  |  |  |  |  |
| **Symptoms** | M | SD | N | M | SD | N | M | SD | N | M | SD | N | F | Df | p | Subgroup differences |
| Depressive symptoms |  |  |  |  |  |  |  |  |  |  |  |  |  |  |  |  |
| Disorganization symptoms |  |  |  |  |  |  |  |  |  |  |  |  |  |  |  |  |
| Negative symptoms |  |  |  |  |  |  |  |  |  |  |  |  |  |  |  |  |
| Overall symptoms |  |  |  |  |  |  |  |  |  |  |  |  |  |  |  |  |
| Positive symptoms |  |  |  | 61.9 | 22.5 | 4 |  |  |  | 65.4 | 38.8 | 6 | 0.0 | 1 | 0.88 |  |
| **Functioning** | M | SD | N | M | SD | N | M | SD | N | M | SD | N | F | Df | p | Subgroup differences |
| Independence |  |  |  |  |  |  |  |  |  |  |  |  |  |  |  |  |
| Overall social functioning |  |  |  | 58.0 | 36.7 | 3 |  |  |  | 58.5 | 17.6 | 7 | 0.0 | 1 | 0.98 |  |
| Prosocial behavior |  |  |  |  |  |  |  |  |  |  |  |  |  |  |  |  |
| Vocational functioning |  |  |  |  |  |  |  |  |  |  |  |  |  |  |  |  |
| **Personal recovery and QOL** | M | SD | N | M | SD | N | M | SD | N | M | SD | N | F | Df | p | Subgroup differences |
| Personal recovery |  |  |  |  |  |  |  |  |  |  |  |  |  |  |  |  |
| Subjective quality of life |  |  |  |  |  |  |  |  |  |  |  |  |  |  |  |  |
| **Cognition** | M | SD | N | M | SD | N | M | SD | N | M | SD | N | F | Df | p | Subgroup differences |
| Executive functioning |  |  |  |  |  |  |  |  |  |  |  |  |  |  |  |  |
| Overall cognition |  |  |  |  |  |  |  |  |  |  |  |  |  |  |  |  |
| Processing speed |  |  |  |  |  |  |  |  |  |  |  |  |  |  |  |  |
| Verbal memory |  |  |  |  |  |  |  |  |  |  |  |  |  |  |  |  |
| **Study characteristics** | M | SD | N | M | SD | N | M | SD | N | M | SD | N | F | Df | p | Subgroup differences |
| Attrition rate |  |  |  | 39.2 | 10.0 | 4 | 58.8 |  | 1 | 30.7 | 12.7 | 7 | 2.7 | 2 | 0.12 |  |
|  | n | % | N | n | % | N | n | % | N | n | % | N | χ^2^ | df | p | Subgroup differences |
| DOI subgroup overlap (yes vs no) |  |  |  |  |  |  |  |  |  |  |  |  |  |  |  |  |
| Publication year <10 years |  |  |  | 2 | 50.0% | 4 | 1 | 100.0% | 1 | 3 | 42.9% | 7 | 1.1 | 2 | 0.57 |  |
| Study design: clinical trial |  |  |  | 1 | 25.0% | 4 |  |  |  | 1 | 14.3% | 7 | 0.2 | 1 | 1.00 |  |
| **Quality assessment** | n | % | N | n | % | N | n | % | N | n | % | N | χ^2^ | df | p | Subgroup differences |
| Study participation: Low ROB |  |  |  | 4 | 100.0% | 4 | 0 | 0.0% | 1 | 3 | 42.9% | 7 | 4.9 | 2 | 0.08 |  |
| Study attrition: Low ROB |  |  |  | 1 | 25.0% | 4 | 0 | 0.0% | 1 | 1 | 14.3% | 7 | 2.6 | 2 | 0.62 |  |
| Prognostic Factor Measurement: Low ROB |  |  |  | 3 | 75.0% | 4 | 0 | 0.0% | 1 | 1 | 14.3% | 7 | 6.7 | 2 | 0.15 |  |
| Outcome measurement: Low ROB |  |  |  | 3 | 75.0% | 4 | 1 | 100.0% | 1 | 5 | 71.4% | 7 | 0.4 | 2 | 0.83 |  |
| Study confounding: Low ROB |  |  |  | 3 | 75.0% | 4 | 1 | 100.0% | 1 | 4 | 57.1% | 7 | 2.1 | 2 | 0.72 |  |
| Statistical analysis and reporting: Low ROB |  |  |  |  |  |  |  |  |  |  |  |  |  |  |  |  |
| **Overall social functioning and Overall cognition** | | | | | | | | | | | | | | | | |
| **Subgroup**:** | 1. both moderate-high improvement | | | 2. Only one moderate- high improvement | | | 3. Both marginal-no improvement | | | 4. No improvement or deterioration | | | **ANOVA** | | | |
| **Demographic variables** | M | SD | N | M | SD | N | M | SD | N | M | SD | N | F | Df | p | Subgroup differences |
| Age | 24.8 |  | 1 | 37.6 | 12.0 | 2 | 27.7 |  | 1 | 41.8 | 15.3 | 9 | 0.6 | 3 | 0.63 |  |
| Age at onset* | 21.1 |  | 1 | 27.4 | 0.9 | 2 | 23.3 |  | 1 | 23.7 | 1.5 | 9 | 5.3 | 3 | 0.02 | 2>4 |
| Ethnicity: % Caucasian or born in country of residence |  |  |  |  |  |  |  |  |  |  |  |  |  |  |  |  |
| Female gender (%)* | 25.5 |  | 1 | 48.8 | 9.6 | 2 | 23.8 |  | 1 | 29.9 | 7.9 | 9 | 3.6 | 3 | 0.06 | 2>4 |
| Marital status: % not married/in relationship |  |  |  |  |  |  |  |  |  |  |  |  |  |  |  |  |
|  | n | % | N | n | % | N | n | % | N | n | % | N | χ^2^ | df | p | Subgroup differences |
| Education level (high level) |  |  |  |  |  |  |  |  |  |  |  |  |  |  |  |  |
| **Clinical variables** | M | SD | N | M | SD | N | M | SD | N | M | SD | N | F | Df | p | Subgroup differences |
| Duration of untreated psychosis in weeks |  |  |  |  |  |  |  |  |  |  |  |  |  |  |  |  |
| Duration of illness (DOI) in years | 3.7 |  | 1 | 10.1 | 11.0 | 2 | 4.4 |  | 1 | 18.1 | 14.7 | 9 | 0.6 | 3 | 0.63 |  |
| Schizophrenia diagnosis (%) | 80.4 |  | 1 | 80.0 | 28.3 | 2 |  |  |  | 93.8 | 12.5 | 9 | 0.9 | 2 | 0.43 |  |
|  | n | % | N | n | % | N | n | % | N | n | % | N | χ^2^ | df | p | Subgroup differences |
| Antipsychotic use by all participants | 1 | 100.0% | 1 | 2 | 100.0% | 2 | 0 | 0.0% | 1 | 4 | 44.4% | 9 | 4.1 | 3 | 0.26 |  |
| Combined treatment of psychosocial and pharmacological therapy provided |  |  |  |  |  |  |  |  |  |  |  |  |  |  |  |  |
| Treatment provided focused on targeted outcomes. |  |  |  |  |  |  |  |  |  |  |  |  |  |  |  |  |
| **Symptoms** | M | SD | N | M | SD | N | M | SD | N | M | SD | N | F | Df | p | Subgroup differences |
| Depressive symptoms |  |  |  |  |  |  |  |  |  |  |  |  |  |  |  |  |
| Disorganization symptoms |  |  |  |  |  |  |  |  |  |  |  |  |  |  |  |  |
| Negative symptoms | 90.3 |  | 1 | 38.4 | 44.8 | 2 | 96.4 |  | 1 | 53.5 | 27.4 | 7 | 1.2 | 3 | 0.37 |  |
| Overall symptoms |  |  |  |  |  |  |  |  |  |  |  |  |  |  |  |  |
| Positive symptoms | 89.4 |  | 1 | 52.8 | 25.9 | 2 | 93.3 |  | 1 | 73.5 | 23.0 | 6 | 0.9 | 3 | 0.49 |  |
| **Functioning** | M | SD | N | M | SD | N | M | SD | N | M | SD | N | F | Df | p | Subgroup differences |
| Independence |  |  |  |  |  |  |  |  |  |  |  |  |  |  |  |  |
| Overall social functioning* |  |  |  | 94.0 | 8.4 | 2 | 56.2 |  | 1 | 55.2 | 15.5 | 8 | 5.6 | 2 | 0.03 | 2>4 |
| Prosocial behavior |  |  |  |  |  |  |  |  |  |  |  |  |  |  |  |  |
| Vocational functioning |  |  |  |  |  |  |  |  |  |  |  |  |  |  |  |  |
| **Personal recovery and QOL** | M | SD | N | M | SD | N | M | SD | N | M | SD | N | F | Df | p | Subgroup differences |
| Personal recovery |  |  |  |  |  |  |  |  |  |  |  |  |  |  |  |  |
| Subjective quality of life |  |  |  |  |  |  |  |  |  |  |  |  |  |  |  |  |
| **Cognition** | M | SD | N | M | SD | N | M | SD | N | M | SD | N | F | Df | p | Subgroup differences |
| Executive functioning |  |  |  |  |  |  |  |  |  |  |  |  |  |  |  |  |
| Overall cognition | 71.9 |  | 1 | 99.9 |  | 1 |  |  |  | 32.0 | 27.5 | 8 | 3.3 | 2 | 0.10 |  |
| Processing speed |  |  |  |  |  |  |  |  |  |  |  |  |  |  |  |  |
| Verbal memory |  |  |  |  |  |  |  |  |  |  |  |  |  |  |  |  |
| **Study characteristics** | M | SD | N | M | SD | N | M | SD | N | M | SD | N | F | Df | p | Subgroup differences |
| Attrition rate | 52.0 |  | 1 | 17.6 | 14.1 | 2 | 42.1 |  | 1 | 37.8 | 14.2 | 9 | 1.7 | 3 | 0.24 |  |
|  | n | % | N | n | % | N | n | % | N | n | % | N | χ^2^ | df | p | Subgroup differences |
| DOI subgroup overlap (yes vs no) |  |  |  | 1 | 50.0% | 2 | 1 | 100.0% | 1 | 3 | 42.9% | 7 | 1.1 | 2 | 0.57 |  |
| Publication year <10 years | 1 | 100.0% | 1 | 1 | 50.0% | 2 | 1 | 100.0% | 1 | 5 | 55.6% | 9 | 1.5 | 3 | 0.68 |  |
| Study design: clinical trial | 1 | 100.0% | 1 | 1 | 50.0% | 2 | 0 | 0.0% | 1 | 2 | 25.0% | 8 | 3.0 | 3 | 0.39 |  |
| **Quality assessment** | n | % | N | n | % | N | n | % | N | n | % | N | χ^2^ | df | p | Subgroup differences |
| Study participation: Low ROB | 1 | 100.0% | 1 | 2 | 100.0% | 2 | 0 | 0.0% | 1 | 3 | 33.3% | 9 | 5.0 | 3 | 0.18 |  |
| Study attrition: Low ROB | 0 | 0.0% | 1 | 2 | 100.0% | 2 | 0 | 0.0% | 1 | 2 | 22.2% | 9 | 8.7 | 3 | 0.19 |  |
| Prognostic Factor Measurement: Low ROB | 1 | 100.0% | 1 | 1 | 50.0% | 2 | 0 | 0.0% | 1 | 0 | 0.0% | 9 | 12.4 | 3 | 0.06 |  |
| Outcome measurement: Low ROB | 0 | 0.0% | 1 | 2 | 100.0% | 2 | 1 | 100.0% | 1 | 8 | 88.9% | 9 | 6.2 | 3 | 0.10 |  |
| Study confounding: Low ROB | 0 | 0.0% | 1 | 1 | 50.0% | 2 | 0 | 0.0% | 1 | 5 | 55.6% | 9 | 7.8 | 3 | 0.25 |  |
| Statistical analysis and reporting: Low ROB |  |  |  |  |  |  |  |  |  |  |  |  |  |  |  |  |
| **Overall social functioning and Processing speed** | | | | | | | | | | | | | | | | |
| **Subgroup**:** | 1. both moderate-high improvement | | | 2. Only one moderate- high improvement | | | 3. Both marginal-no improvement | | | 4. No improvement or deterioration | | | **ANOVA** | | | |
| **Demographic variables** | M | SD | N | M | SD | N | M | SD | N | M | SD | N | F | Df | p | Subgroup differences |
| Age* | 26.9 | 3.1 | 2 | 27.5 | 1.1 | 2 | 54.6 |  | 1 | 37.0 | 6.6 | 6 | 6.7 | 3 | 0.02 | 2<3 / 2<4 |
| Age at onset* | 23.9 | 4.1 | 2 | 27.4 | 1.0 | 2 | 23.1 |  | 1 | 24.0 | 1.7 | 5 | 1.4 | 3 | 0.32 | 2>4 |
| Ethnicity: % Caucasian or born in country of residence |  |  |  |  |  |  |  |  |  |  |  |  |  |  |  |  |
| Female gender (%)* | 33.7 | 11.7 | 2 | 41.4 | 1.4 | 2 | 34.6 |  | 1 | 25.7 | 8.0 | 6 | 2.1 | 3 | 0.19 | 2>4 |
| Marital status: % not married/in relationship |  |  |  |  |  |  |  |  |  |  |  |  |  |  |  |  |
|  | n | % | N | n | % | N | n | % | N | n | % | N | χ^2^ | df | p | Subgroup differences |
| Education level (high level) | 1 | 50.0% | 2 | 2 | 100.0% | 2 | 0 | 0.0% | 1 | 3 | 50.0% | 6 | 2.9 | 3 | 0.40 |  |
| **Clinical variables** | M | SD | N | M | SD | N | M | SD | N | M | SD | N | F | Df | p | Subgroup differences |
| Duration of untreated psychosis in weeks |  |  |  |  |  |  |  |  |  |  |  |  |  |  |  |  |
| Duration of illness (DOI) in years* | 3.0 | 1.0 | 2 | 0.1 | 0.1 | 2 | 31.5 |  | 1 | 10.2 | 6.0 | 6 | 9.5 | 3 | 0.01 | 1<3 / 2<3 / 3>4 |
| Schizophrenia diagnosis (%)* | 70.2 | 14.4 | 2 | 51.1 | 1.6 | 2 | 100.0 |  | 1 | 88.9 | 15.4 | 5 | 4.6 | 3 | 0.05 | 2<3 / 2<4 |
|  | n | % | N | n | % | N | n | % | N | n | % | N | χ^2^ | df | p | Subgroup differences |
| Antipsychotic use by all participants | 2 | 100.0% | 2 | 0 | 0.0% | 2 | 0 | 0.0% | 1 | 2 | 33.3% | 6 | 5.2 | 3 | 0.15 |  |
| Combined treatment of psychosocial and pharmacological therapy provided |  |  |  |  |  |  |  |  |  |  |  |  |  |  |  |  |
| Treatment provided focused on targeted outcomes. |  |  |  |  |  |  |  |  |  |  |  |  |  |  |  |  |
| **Symptoms** | M | SD | N | M | SD | N | M | SD | N | M | SD | N | F | Df | p | Subgroup differences |
| Depressive symptoms |  |  |  |  |  |  |  |  |  |  |  |  |  |  |  |  |
| Disorganization symptoms |  |  |  |  |  |  |  |  |  |  |  |  |  |  |  |  |
| Negative symptoms |  |  |  |  |  |  |  |  |  |  |  |  |  |  |  |  |
| Overall symptoms |  |  |  |  |  |  |  |  |  |  |  |  |  |  |  |  |
| Positive symptoms | 62.0 | 38.8 | 2 | 61.8 | 2.7 | 2 | 95.5 |  | 1 | 59.3 | 40.2 | 5 | 0.3 | 3 | 0.84 |  |
| **Functioning** | M | SD | N | M | SD | N | M | SD | N | M | SD | N | F | Df | p | Subgroup differences |
| Independence |  |  |  |  |  |  |  |  |  |  |  |  |  |  |  |  |
| Overall social functioning | 99.9 |  | 1 | 37.1 | 7.8 | 2 | 67.2 |  | 1 | 57.0 | 18.8 | 6 | 3.0 | 3 | 0.12 |  |
| Prosocial behavior |  |  |  |  |  |  |  |  |  |  |  |  |  |  |  |  |
| Vocational functioning |  |  |  |  |  |  |  |  |  |  |  |  |  |  |  |  |
| **Personal recovery and QOL** | M | SD | N | M | SD | N | M | SD | N | M | SD | N | F | Df | p | Subgroup differences |
| Personal recovery |  |  |  |  |  |  |  |  |  |  |  |  |  |  |  |  |
| Subjective quality of life |  |  |  |  |  |  |  |  |  |  |  |  |  |  |  |  |
| **Cognition** | M | SD | N | M | SD | N | M | SD | N | M | SD | N | F | Df | p | Subgroup differences |
| Executive functioning |  |  |  |  |  |  |  |  |  |  |  |  |  |  |  |  |
| Overall cognition |  |  |  |  |  |  |  |  |  |  |  |  |  |  |  |  |
| Processing speed |  |  |  |  |  |  |  |  |  |  |  |  |  |  |  |  |
| Verbal memory |  |  |  |  |  |  |  |  |  |  |  |  |  |  |  |  |
| **Study characteristics** | M | SD | N | M | SD | N | M | SD | N | M | SD | N | F | Df | p | Subgroup differences |
| Attrition rate* | 39.8 | 17.3 | 2 | 38.7 | 0.3 | 2 | 28.2 |  | 1 | 31.1 | 13.9 | 6 | 0.4 | 3 | 0.79 | 2>3 |
|  | n | % | N | n | % | N | n | % | N | n | % | N | χ^2^ | df | p | Subgroup differences |
| DOI subgroup overlap (yes vs no) |  |  |  |  |  |  |  |  |  |  |  |  |  |  |  |  |
| Publication year <10 years | 1 | 50.0% | 2 | 1 | 50.0% | 2 | 0 | 0.0% | 1 | 3 | 50.0% | 6 | 0.9 | 3 | 0.82 |  |
| Study design: clinical trial | 1 | 50.0% | 2 | 0 | 0.0% | 2 | 0 | 0.0% | 1 | 1 | 16.7% | 6 | 2.0 | 3 | 0.57 |  |
| **Quality assessment** | n | % | N | n | % | N | n | % | N | n | % | N | χ^2^ | df | p | Subgroup differences |
| Study participation: Low ROB | 2 | 100.0% | 2 | 2 | 100.0% | 2 | 0 | 0.0% | 1 | 3 | 50.0% | 6 | 4.5 | 3 | 0.21 |  |
| Study attrition: Low ROB | 1 | 50.0% | 2 | 0 | 0.0% | 2 | 0 | 0.0% | 1 | 1 | 16.7% | 6 | 3.9 | 3 | 0.70 |  |
| Prognostic Factor Measurement: Low ROB | 1 | 50.0% | 2 | 2 | 100.0% | 2 | 0 | 0.0% | 1 | 1 | 16.7% | 6 | 8.1 | 3 | 0.23 |  |
| Outcome measurement: Low ROB | 1 | 50.0% | 2 | 2 | 100.0% | 2 | 1 | 100.0% | 1 | 4 | 66.7% | 6 | 1.8 | 3 | 0.62 |  |
| Study confounding: Low ROB | 1 | 50.0% | 2 | 2 | 100.0% | 2 | 1 | 100.0% | 1 | 3 | 50.0% | 6 | 4.2 | 3 | 0.65 |  |
| Statistical analysis and reporting: Low ROB |  |  |  |  |  |  |  |  |  |  |  |  |  |  |  |  |
| **Overall social functioning and Verbal memory** | | | | | | | | | | | | | | | | |
| **Subgroup**:** | 1. both moderate-high improvement | | | 2. Only one moderate- high improvement | | | 3. Both marginal-no improvement | | | 4. No improvement or deterioration | | | **ANOVA** | | | |
| **Demographic variables** | M | SD | N | M | SD | N | M | SD | N | M | SD | N | F | Df | p | Subgroup differences |
| Age* |  |  |  | 27.2 | 1.9 | 4 | 26.3 |  | 1 | 43.1 | 13.0 | 27.2 | 3.3 | 2 | 0.08 | 2<4 |
| Age at onset |  |  |  | 25.6 | 3.1 | 4 | 25.0 |  | 1 | 24.2 | 1.7 | 25.6 | 0.5 | 2 | 0.62 |  |
| Ethnicity: % Caucasian or born in country of residence |  |  |  |  |  |  |  |  |  |  |  |  |  |  |  |  |
| Female gender (%) |  |  |  | 37.6 | 8.1 | 4 | 26.2 |  | 1 | 28.3 | 8.3 | 8 | 1.9 | 2 | 0.21 |  |
| Marital status: % not married/in relationship |  |  |  |  |  |  |  |  |  |  |  |  |  |  |  |  |
|  | n | % | N | n | % | N | n | % | N | n | % | N | χ^2^ | df | p | Subgroup differences |
| Education level (high level) |  |  |  | 3 | 75.0% | 4 |  |  |  | 3 | 37.5% | 8 | 1.5 | 1 | 0.55 |  |
| **Clinical variables** | M | SD | N | M | SD | N | M | SD | N | M | SD | N | F | Df | p | Subgroup differences |
| Duration of untreated psychosis in weeks |  |  |  |  |  |  |  |  |  |  |  |  |  |  |  |  |
| Duration of illness (DOI) in years |  |  |  | 1.5 | 1.8 | 4 | 1.3 |  | 1 | 16.7 | 13.4 | 8 | 2.8 | 2 | 0.11 |  |
| Schizophrenia diagnosis (%)* |  |  |  | 60.7 | 13.8 | 4 | 100.0 |  | 1 | 92.1 | 13.7 | 7 | 7.6 | 2 | 0.01 | 2<4 |
|  | n | % | N | n | % | N | n | % | N | n | % | N | χ^2^ | df | p | Subgroup differences |
| Antipsychotic use by all participants |  |  |  | 2 | 50.0% | 4 | 0 | 0.0% | 1 | 2 | 25.0% | 8 | 1.3 | 2 | 0.53 |  |
| Combined treatment of psychosocial and pharmacological therapy provided |  |  |  |  |  |  |  |  |  |  |  |  |  |  |  |  |
| Treatment provided focused on targeted outcomes. |  |  |  |  |  |  |  |  |  |  |  |  |  |  |  |  |
| **Symptoms** | M | SD | N | M | SD | N | M | SD | N | M | SD | N | F | Df | p | Subgroup differences |
| Depressive symptoms |  |  |  |  |  |  |  |  |  |  |  |  |  |  |  |  |
| Disorganization symptoms |  |  |  |  |  |  |  |  |  |  |  |  |  |  |  |  |
| Negative symptoms |  |  |  | 37.2 | 46.1 | 3 |  |  |  | 62.1 | 24.1 | 7 | 1.3 | 1 | 0.28 |  |
| Overall symptoms |  |  |  |  |  |  |  |  |  |  |  |  |  |  |  |  |
| Positive symptoms |  |  |  | 61.9 | 22.5 | 4 |  |  |  | 62.3 | 36.4 | 7 | 0.0 | 1 | 0.98 |  |
| **Functioning** | M | SD | N | M | SD | N | M | SD | N | M | SD | N | F | Df | p | Subgroup differences |
| Independence |  |  |  |  |  |  |  |  |  |  |  |  |  |  |  |  |
| Overall social functioning |  |  |  | 58.0 | 36.7 | 3 |  |  |  | 55.2 | 18.7 | 8 | 0.0 | 1 | 0.87 |  |
| Prosocial behavior |  |  |  |  |  |  |  |  |  |  |  |  |  |  |  |  |
| Vocational functioning |  |  |  |  |  |  |  |  |  |  |  |  |  |  |  |  |
| **Personal recovery and QOL** | M | SD | N | M | SD | N | M | SD | N | M | SD | N | F | Df | p | Subgroup differences |
| Personal recovery |  |  |  |  |  |  |  |  |  |  |  |  |  |  |  |  |
| Subjective quality of life |  |  |  |  |  |  |  |  |  |  |  |  |  |  |  |  |
| **Cognition** | M | SD | N | M | SD | N | M | SD | N | M | SD | N | F | Df | p | Subgroup differences |
| Executive functioning |  |  |  |  |  |  |  |  |  |  |  |  |  |  |  |  |
| Overall cognition |  |  |  |  |  |  |  |  |  |  |  |  |  |  |  |  |
| Processing speed |  |  |  |  |  |  |  |  |  |  |  |  |  |  |  |  |
| Verbal memory |  |  |  | 74.1 | 13.3 | 3 |  |  |  | 46.3 | 25.5 | 7 | 3.1 | 1 | 0.12 |  |
| **Study characteristics** | M | SD | N | M | SD | N | M | SD | N | M | SD | N | F | Df | p | Subgroup differences |
| Attrition rate |  |  |  | 39.2 | 10.0 | 4 | 58.8 |  | 1 | 30.8 | 11.8 | 8 | 3.1 | 2 | 0.09 |  |
|  | n | % | N | n | % | N | n | % | N | n | % | N | χ^2^ | df | p | Subgroup differences |
| DOI subgroup overlap (yes vs no) |  |  |  | 0 | 0.0% | 3 |  |  |  | 3 | 42.9% | 7 | 1.8 | 1 | 0.48 |  |
| Publication year <10 years |  |  |  | 2 | 50.0% | 4 | 1 | 100.0% | 1 | 3 | 37.5% | 8 | 1.4 | 2 | 0.49 |  |
| Study design: clinical trial |  |  |  | 1 | 25.0% | 4 |  |  |  | 1 | 12.5% | 8 | 0.3 | 1 | 0.58 |  |
| **Quality assessment** | n | % | N | n | % | N | n | % | N | n | % | N | χ^2^ | df | p | Subgroup differences |
| Study participation: Low ROB |  |  |  | 4 | 100.0% | 4 | 0 | 0.0% | 1 | 4 | 50.0% | 8 | 4.6 | 2 | 0.10 |  |
| Study attrition: Low ROB |  |  |  | 1 | 25.0% | 4 | 0 | 0.0% | 1 | 1 | 12.5% | 8 | 3.3 | 2 | 0.52 |  |
| Prognostic Factor Measurement: Low ROB |  |  |  | 3 | 75.0% | 4 | 0 | 0.0% | 1 | 1 | 12.5% | 8 | 7.4 | 2 | 0.12 |  |
| Outcome measurement: Low ROB |  |  |  | 3 | 75.0% | 4 | 0 | 0.0% | 1 | 6 | 75.0% | 8 | 0.3 | 2 | 0.85 |  |
| Study confounding: Low ROB |  |  |  | 3 | 75.0% | 4 | 1 | 100.0% | 1 | 5 | 62.5% | 8 | 1.9 | 2 | 0.76 |  |
| Statistical analysis and reporting: Low ROB |  |  |  |  |  |  |  |  |  |  |  |  |  |  |  |  |

**Social functioning and PR/QOL associations**

| **Prosocial behavior and Subjective quality of life** | | | | | | | | | | | | | | | | | | |
| --- | --- | --- | --- | --- | --- | --- | --- | --- | --- | --- | --- | --- | --- | --- | --- | --- | --- | --- |
| **Subgroup**:** | 1. both moderate-high improvement | | | 2. Only one moderate- high improvement | | | | | 3. Both marginal-no improvement | | | 4. No improvement or deterioration | | | **ANOVA** | | | |
| **Demographic variables** | M | SD | N | M | SD | | N | | M | SD | N | M | SD | N | F | Df | p | Subgroup differences |
| Age |  |  |  | 26.9 | 4.9 | | 3 | | 32.4 |  | 1 | 39.6 | 10.1 | 6 | 2.1 | 2 | 0.20 |  |
| Age at onset |  |  |  | 25.3 | 4.9 | | 3 | | 20.4 |  | 1 | 23.0 | 2.9 | 6 | 0.8 | 2 | 0.48 |  |
| Ethnicity: % Caucasian or born in country of residence |  |  |  |  |  | |  | |  |  |  |  |  |  |  |  |  |  |
| Female gender (%) |  |  |  | 38.0 | 7.4 | | 3 | | 22.4 |  | 1 | 35.1 | 7.1 | 6 | 1.8 | 2 | 0.23 |  |
| Marital status: % not married/in relationship |  |  |  |  |  | |  | |  |  |  |  |  |  |  |  |  |  |
|  | n | % | N | n | % | | N | | n | % | N | n | % | N | χ^2^ | df | p | Subgroup differences |
| Education level (high level) |  |  |  |  |  | |  | |  |  |  |  |  |  |  |  |  |  |
| **Clinical variables** | M | SD | N | M | SD | | N | | M | SD | N | M | SD | N | F | Df | p | Subgroup differences |
| Duration of untreated psychosis in weeks |  |  |  |  |  | |  | |  |  |  |  |  |  |  |  |  |  |
| Duration of illness (DOI) in years |  |  |  | 7.6 | 10.8 | | 3 | | 12.0 |  | 1 | 16.7 | 10.6 | 6 | 0.7 | 1 | 0.49 |  |
| Schizophrenia diagnosis (%) |  |  |  |  |  | |  | |  |  |  |  |  |  |  |  |  |  |
|  | n | % | N | n | % | | N | | n | % | N | n | % | N | χ^2^ | df | p | Subgroup differences |
| Antipsychotic use by all participants |  |  |  | 2 | 66.7% | | 3 | | 0 | 0.0% | 1 | 2 | 33.3% | 6 | 1.7 | 2 | 0.44 |  |
| Combined treatment of psychosocial and pharmacological therapy provided |  |  |  |  |  | |  | |  |  |  |  |  |  |  |  |  |  |
| Treatment provided focused on targeted outcomes. |  |  |  |  |  | |  | |  |  |  |  |  |  |  |  |  |  |
| **Symptoms** | M | SD | N | M | SD | | N | | M | SD | N | M | SD | N | F | Df | p | Subgroup differences |
| Depressive symptoms |  |  |  |  |  | |  | |  |  |  |  |  |  |  |  |  |  |
| Disorganization symptoms |  |  |  |  |  | |  | |  |  |  |  |  |  |  |  |  |  |
| Negative symptoms |  |  |  |  |  | |  | |  |  |  |  |  |  |  |  |  |  |
| Overall symptoms |  |  |  |  |  | |  | |  |  |  |  |  |  |  |  |  |  |
| Positive symptoms |  |  |  |  |  | |  | |  |  |  |  |  |  |  |  |  |  |
| **Functioning** | M | SD | N | M | SD | | N | | M | SD | N | M | SD | N | F | Df | p | Subgroup differences |
| Independence |  |  |  |  |  | |  | |  |  |  |  |  |  |  |  |  |  |
| Overall social functioning |  |  |  |  |  | |  | |  |  |  |  |  |  |  |  |  |  |
| Prosocial behavior |  |  |  | 63.7 |  | | 1 | | 42.7 |  | 1 | 45.1 | 11.6 | 4 | 1.1 | 2 | 0.43 |  |
| Vocational functioning |  |  |  |  |  | |  | |  |  |  |  |  |  |  |  |  |  |
| **Personal recovery and QOL** | M | SD | N | M | SD | | N | | M | SD | N | M | SD | N | F | Df | p | Subgroup differences |
| Personal recovery |  |  |  |  |  | |  | |  |  |  |  |  |  |  |  |  |  |
| Subjective quality of life* |  |  |  | 67.1 | 8.0 | | 3 | | 87.5 |  | 1 | 59.4 | 8.4 | 6 | 5.1 | 2 | 0.04 | 3>4 |
| **Cognition** | M | SD | N | M | SD | | N | | M | SD | N | M | SD | N | F | Df | p | Subgroup differences |
| Executive functioning |  |  |  |  |  | |  | |  |  |  |  |  |  |  |  |  |  |
| Overall cognition |  |  |  |  |  | |  | |  |  |  |  |  |  |  |  |  |  |
| Processing speed |  |  |  |  |  | |  | |  |  |  |  |  |  |  |  |  |  |
| Verbal memory |  |  |  |  |  | |  | |  |  |  |  |  |  |  |  |  |  |
| **Study characteristics** | M | SD | N | M | SD | | N | | M | SD | N | M | SD | N | F | Df | p | Subgroup differences |
| Attrition rate |  |  |  | 44.5 | 27.8 | | 3 | | 23.1 |  | 1 | 20.0 | 11.8 | 6 | 1.9 | 2 | 0.22 |  |
|  | n | % | N | n | % | | N | | n | % | N | n | % | N | χ^2^ | df | p | Subgroup differences |
| DOI subgroup overlap (yes vs no) |  |  |  |  |  | |  | |  |  |  |  |  |  |  |  |  |  |
| Publication year <10 years |  |  |  | 2 | 66.7% | | 3 | | 0 | 0.0% | 1 | 2 | 33.3% | 6 | 1.7 | 2 | 0.44 |  |
| Study design: clinical trial |  |  |  | 2 | 66.7% | | 3 | | 1 | 100.0% | 1 | 2 | 33.3% | 6 | 2.0 | 2 | 0.37 |  |
| **Quality assessment** | n | % | N | n | | % | | N | n | % | N | n | % | N | χ^2^ | df | p | Subgroup differences |
| Study participation: Low ROB |  |  |  | 2 | | 66.7% | | 3 | 1 | 100.0% | 1 | 2 | 33.3% | 6 | 2.0 | 2 | 0.37 |  |
| Study attrition: Low ROB |  |  |  | 0 | | 0.0% | | 3 | 1 | 100.0% | 1 | 3 | 50.0% | 6 | 4.6 | 2 | 0.33 |  |
| Prognostic Factor Measurement: Low ROB |  |  |  | 2 | | 66.7% | | 3 | 0 | 0.0% | 1 | 4 | 66.7% | 6 | 5.0 | 2 | 0.29 |  |
| Outcome measurement: Low ROB |  |  |  |  | |  | |  |  |  |  |  |  |  |  |  |  |  |
| Study confounding: Low ROB |  |  |  | 1 | | 33.3% | | 3 | 0 | 0.0% | 1 | 3 | 50.0% | 6 | 2.3 | 2 | 0.69 |  |
| Statistical analysis and reporting: Low ROB |  |  |  | 3 | | 100.0% | | 3 | 1 | 100.0% | 1 | 4 | 66.7% | 6 | 1.7 | 2 | 0.80 |  |

* significant moderator: p<0.05

** Some moderators were remained empty in this analysis when these moderators were only presented in one or two subgroups.

# **Supplementary material 9.** Quality assessment (QUIPS) outcome included studies

| **Study name** | **Study participation** | **Study attrition** | **Prognostic factor measurement** | **Outcome measurement** | **Study confounding** | **Statistical analysis and report** |
| --- | --- | --- | --- | --- | --- | --- |
| Addington 2000 | Moderate | Low | Low | Low | High | High |
| Aguilar 2018 | High | Low | Unclear | Low | High | Low |
| Albus 2002 | Low | High | Unclear | Low | Low | Low |
| Alphs 2022 | Low | High | Moderate | Low | High | Low |
| Breier 2018 | Moderate | High | Moderate | Low | Moderate | Low |
| Buonocore 2018 | Moderate | Low | Low | Low | Moderate | Moderate |
| Cai 2022 | Low | Low | Low | Low | Moderate | Low |
| Cechnicki 2017 | Moderate | Moderate | Unclear | Moderate | High | Low |
| Chan 2003 | Low | Moderate | Unclear | Low | High | Moderate |
| Chan 2018 | Low | Low | Moderate | Low | Moderate | Moderate |
| Chanpattana 2010 | Low | High | Low | Moderate | Low | Low |
| Chen 2000 | Moderate | High | Moderate | Low | Moderate | Low |
| Chen 2005 | Moderate | Moderate | Moderate | Low | Low | Moderate |
| Chien 2017 | Low | Low | Low | Low | High | Low |
| Ciudad 2009 | Moderate | Low | Moderate | Low | Low | Low |
| Conley 2007 | Low | Low | Low | Low | Moderate | Low |
| Cullberg 2002 | Low | Moderate | Moderate | Low | Low | Low |
| Dal Santo 2020 | High | Moderate | Moderate | Moderate | High | Moderate |
| Dellazizzo 2023 | Moderate | Low | Unclear | Low | High | Low |
| Dixon 2015 | Low | High | Moderate | Low | Moderate | Low |
| Ekerholm 2012 | Moderate | High | Unclear | Moderate | Moderate | Low |
| Evensen 2016 | Low | Low | Low | Moderate | Moderate | Low |
| Fernandez-Modamio 2021 | Moderate | High | Unclear | Low | Moderate | Moderate |
| Fond 2018 | Moderate | High | Moderate | Moderate | High | Low |
| Foti 2010 | Low | Moderate | Moderate | Low | Low | Low |
| Fowler 2012 | Moderate | Moderate | Low | Low | High | Moderate |
| Fowler 2018 | Moderate | Moderate | Unclear | Low | High | Low |
| Galderisi 2020 | Low | Low | Moderate | Low | High | Low |
| Ganella 2018 | Moderate | High | Unclear | Moderate | High | Low |
| Gaughran 2017 | Low | Low | Unclear | Low | Moderate | Low |
| Godin 2019 | Low | Moderate | Low | Low | Moderate | Low |
| Gorna 2008 | Moderate | Moderate | Low | Low | Low | Low |
| Gorwood 2019 | Low | Moderate | Low | Low | Moderate | Moderate |
| Granholm 2020 | Moderate | Moderate | Moderate | Low | Low | Low |
| Grawe 2006 | Low | Low | Unclear | Moderate | High | Low |
| Gumley 2022 | Low | Moderate | Unclear | Moderate | High | Low |
| Harrow 2005 | Moderate | High | Moderate | High | Low | Low |
| Harvey 2010 | Low | Low | Unclear | Low | Low | Low |
| Hayhurst 2014 | Moderate | Moderate | Low | Low | Low | Low |
| Heeramun-Aubeeluck 2015 | Low | Low | Low | Low | High | Moderate |
| Heering 2015 | Moderate | Moderate | Moderate | Low | High | Low |
| Hoff 2005 | Moderate | Low | Unclear | Low | Moderate | Low |
| Horan 2012 | Moderate | Moderate | Moderate | Low | Low | Low |
| Hui 2023 | Moderate | High | High | Low | High | Moderate |
| Ito 2015 | Low | Low | Moderate | Low | Low | Low |
| Jørgensen 2015 | Low | Low | Unclear | Low | Low | Low |
| Kane 2016 | Low | High | Low | Moderate | Moderate | Low |
| Kelly 2009 | Moderate | High | Unclear | Low | Low | Low |
| Kim 2019 | Moderate | Low | Unclear | Moderate | Low | Low |
| Klærke 2019 | Low | Moderate | Unclear | Low | Moderate | Moderate |
| Koshiyama 2017 | Moderate | High | Moderate | Low | Low | Low |
| Lasser 2005 | Moderate | Moderate | Moderate | Low | Low | Low |
| Lee 2023 | Moderate | Moderate | Unclear | Low | Moderate | Low |
| Li 2017 | Low | High | High | Low | Low | Low |
| Lindgren 2020 | Low | Moderate | Low | Low | Low | Low |
| Litman 2023 | Low | Low | Low | Low | Low | Low |
| Liu 2023 | Moderate | Moderate | Moderate | Low | Moderate | Moderate |
| Lopez-Morinigo 2023 | Low | Low | Moderate | Low | High | Moderate |
| McGurk 2003 | High | Moderate | Moderate | Low | Moderate | Low |
| McNeely 2023 | Moderate | Low | Low | Low | Moderate | Moderate |
| Meade 2020 | Moderate | High | Moderate | Low | Low | Low |
| Meagher 2004 | Moderate | High | Moderate | Low | Low | Low |
| Melle 2008 | Low | Moderate | Low | Low | Low | Low |
| Moncrieff 2023 | Low | Moderate | Low | Low | Low | Low |
| Morrison 2018 | Low | Low | Unclear | Low | Low | Low |
| Na 2016 | Low | Low | Unclear | Low | High | Low |
| Najarian 2023 | Moderate | Low | Unclear | Low | High | Moderate |
| Nakamura 2019 | Low | Low | Unclear | Low | Moderate | Low |
| Neill 2022 | Moderate | High | Moderate | Low | Moderate | Low |
| Nordentoft 2006 | Low | Moderate | Low | Moderate | Low | Low |
| Oh 2017 | Low | Moderate | Moderate | Low | High | Low |
| Okin 1995 | Moderate | Moderate | Low | Low | Moderate | Moderate |
| Oribe 2015 | Moderate | Low | Unclear | Low | Moderate | Low |
| Ortega 2021 | Moderate | Moderate | Low | Low | Moderate | Moderate |
| Ozawa 2019 | Low | Moderate | Unclear | Low | High | Low |
| Prouteau 2005 | Low | Low | Low | Low | Low | Low |
| Putnam 2000 | Low | Moderate | Moderate | Low | Low | Low |
| Rodríguéz-Sánchez 2008 | Low | Low | Unclear | Low | Low | Low |
| Rossi 2009 | Low | Moderate | Low | Low | Low | Low |
| Rowland 2018 | Low | Moderate | Low | Low | Low | Low |
| Rund 2007 | Low | High | Low | Low | Low | Low |
| Ryu 2006 | Moderate | Moderate | Moderate | Low | Low | Low |
| Salyers 2014 | Low | High | Low | Low | Low | Low |
| Schmidt 2017 | Low | Low | Low | Low | Moderate | Low |
| Scottish Schizophrenia Research group 1988 | Moderate | Low | Unclear | High | Moderate | Low |
| She 2017 | Low | High | Unclear | Low | Moderate | Low |
| Siegel 2006 | Moderate | High | Moderate | Moderate | Low | Moderate |
| Sikira 2021 | Low | Low | Low | Low | High | Moderate |
| Smith 2002 | Low | High | Low | Low | Moderate | Low |
| Sommer 2021 | Moderate | High | Moderate | Low | Low | Low |
| Stouten 2014 | Moderate | Low | Low | Low | Low | Low |
| Sweeney 1991 | Moderate | Low | Moderate | Low | High | Low |
| Tabáres-Seisdesos 2005 | Moderate | Moderate | Moderate | Low | Low | Low |
| Tabo 2017 | Moderate | High | Moderate | Moderate | High | Low |
| Torgalsbøen 2015 | Low | Low | Low | Low | Low | Low |
| Üçok 2011 | Moderate | Moderate | Moderate | Moderate | Low | Low |
| Usui 2022 | Low | Low | Moderate | Low | Low | Low |
| Veerman 2016 | Low | Low | Low | Low | Moderate | Moderate |
| Veijola 2014 | Low | High | Moderate | Low | Moderate | Moderate |
| Whitehorn 2002 | Moderate | High | Unclear | Low | High | Moderate |
| Wilson-d'Almeida 2013 | Low | Low | Low | Low | Low | Low |
| Wittorf 2004 | Low | Moderate | Unclear | Low | High | Low |
| Wittorf 2008 | Low | High | Low | Low | Low | Low |
| Wojtalik 2022 | Low | High | Low | Moderate | Moderate | Low |
| Wunderink 2009 | Low | Moderate | Low | Low | Low | Low |
| Xie 2005 | Low | Low | Unclear | Low | Moderate | Low |
| Xu 2014 | Moderate | High | Low | Low | Low | Low |
| Zäske 2018 | Moderate | High | Low | Low | Moderate | Low |
| Zhu 2022 | Moderate | Low | Moderate | Low | Moderate | Low |

# **Supplementary material 10.** Outliers and its influence on the correlations per outcome domain.

| **Outcome domain** | **K positive outliers** | **K negative outliers** | **Correlations with outliers included** | **Correlations without positive outliers** | **Correlations without negative outliers** |
| --- | --- | --- | --- | --- | --- |
| Overall social functioning (k=69 studies) | 9 studies | 16 studies | - Positive symptoms - Negative symptoms - Disorganization symptoms - Depressive symptoms - Verbal memory - Executive functioning - Processing speed - Overall cognition | - Positive symptoms - Negative symptoms - Disorganization symptoms - Depressive symptoms - Processing speed | - Positive symptoms - Negative symptoms - Disorganization symptoms - Depressive symptoms - Verbal memory - Executive functioning - Processing speed - Overall cognition |
| Prosocial behavior  (k=31 studies) | 2 studies | 2 studies | - Positive symptoms - Negative symptoms - Subjective quality of life | - Negative symptoms | - Positive symptoms - Negative symptoms |
| Independence  (k=24 studies) | 4 studies | 0 studies | - Negative symptoms | No correlations | - Negative symptoms |
| Vocational functioning  (k=22 studies) | 4 studies | 2 studies | - Negative symptoms | No correlations | - Negative symptoms |
| Positive symptoms  (k=86 studies) | 13 studies | 18 studies | - Overall social functioning - Prosocial behavior - Executive functioning - Personal recovery - Subjective quality of life | - Overall social functioning - Executive functioning - Personal recovery - Subjective quality of life | - Overall social functioning - Prosocial behavior - Executive functioning - Personal recovery - Subjective quality of life |
| Negative symptoms  (k=86 studies) | 19 studies | 16 studies | - Overall social functioning - Prosocial behavior - Independence - Vocational functioning - Subjective quality of life - Executive functioning - Processing speed - Overall cognition | - Overall social functioning - Prosocial behavior - Subjective quality of life - Executive functioning - Overall cognition | - Overall social functioning - Prosocial behavior - Independence - Vocational functioning - Subjective quality of life - Executive functioning - Processing speed - Overall cognition |
| Disorganization symptoms  (k=19 studies) | 1 study | 0 studies | - Overall social functioning | - Overall social functioning | - Overall social functioning |
| Depressive symptoms  (k=37 studies) | 5 studies | 3 studies | - Overall social functioning - Verbal memory - Processing speed | - Overall social functioning - Processing speed | - Overall social functioning - Verbal memory - Processing speed |
| Personal recovery  (k=33 studies) | 4 studies | 3 studies | - Positive symptoms | - Positive symptoms | - Positive symptoms |
| Subjective quality of life  (k=25 studies) | 2 studies | 3 studies | - Positive symptoms - Negative symptoms - Prosocial behavior | - Positive symptoms - Negative symptoms | - Positive symptoms - Negative symptoms |
| Verbal memory  (k=27 studies) | 2 studies | 2 studies | - Overall social functioning - Depressive symptoms | No correlations | - Overall social functioning - Depressive symptoms |
| Executive functioning  (k=28 studies) | 2 studies | 2 studies | - Overall social functioning - Positive symptoms - Negative symptoms | - Positive symptoms - Negative symptoms | - Overall social functioning - Positive symptoms - Negative symptoms |
| Processing speed  (k=19 studies) | 1 study | 0 studies | - Overall social functioning - Negative symptoms - Depressive symptoms | - Overall social functioning - Negative symptoms - Depressive symptoms | - Overall social functioning - Negative symptoms   Depressive symptoms |
| Overall cognition  (k=26 studies) | 2 studies | 1 study | - Overall social functioning - Negative symptoms | - Negative symptoms | - Overall social functioning - Negative symptoms |

# **
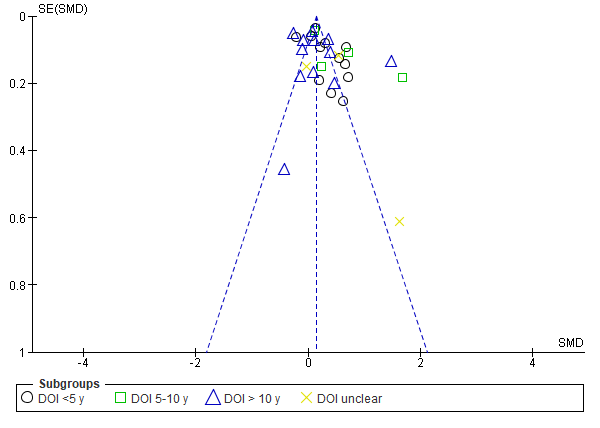
**
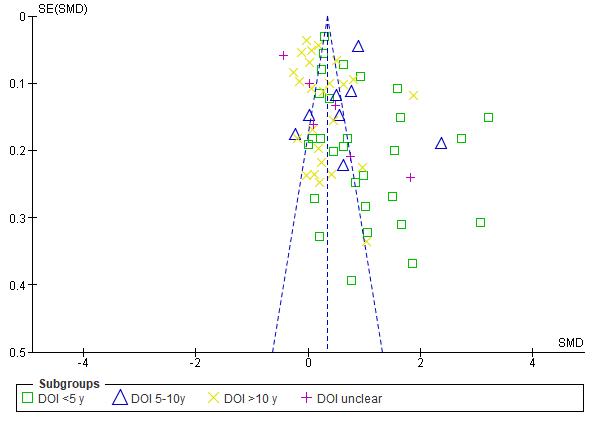
**Supplementary material 11.** Funnel plots of outcome domains

**
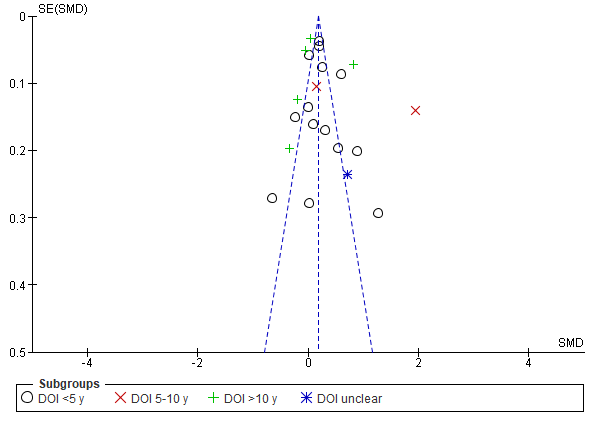

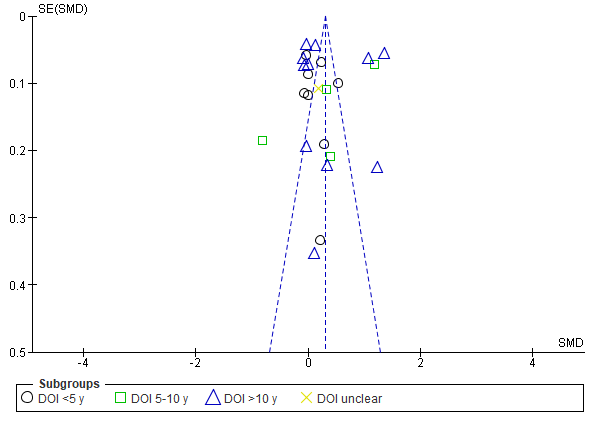
Overall social functioning Prosocial behavior**

**
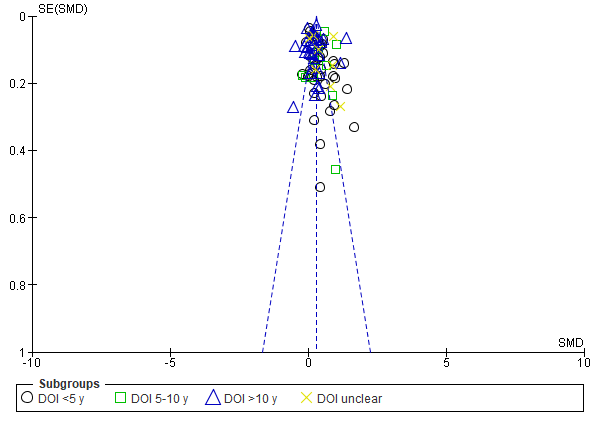

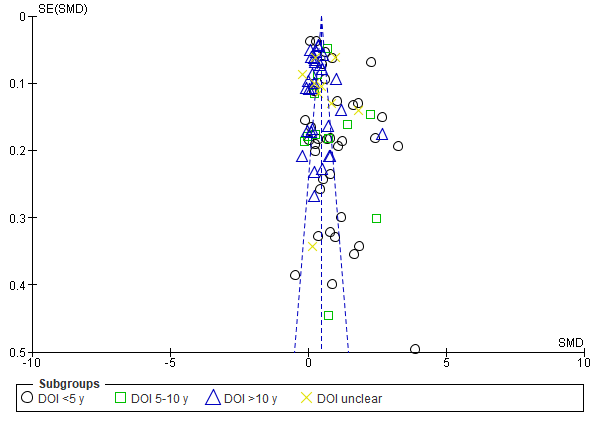
Independence Vocational functioning**

**
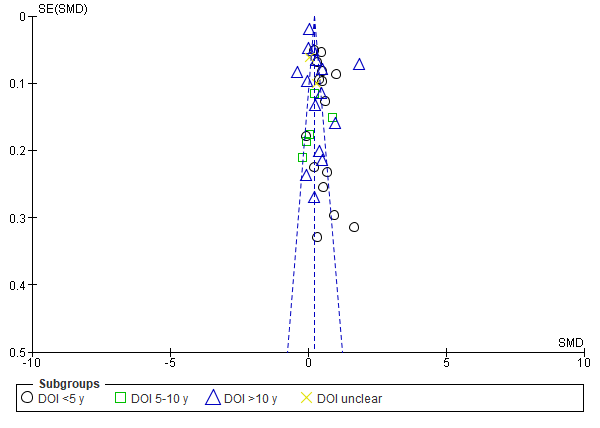

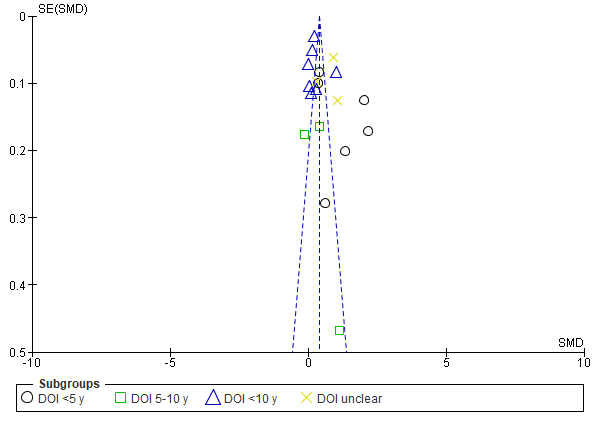
Positive symptoms Negative symptoms**

**Disorganization symptoms Depressive symptoms**

**
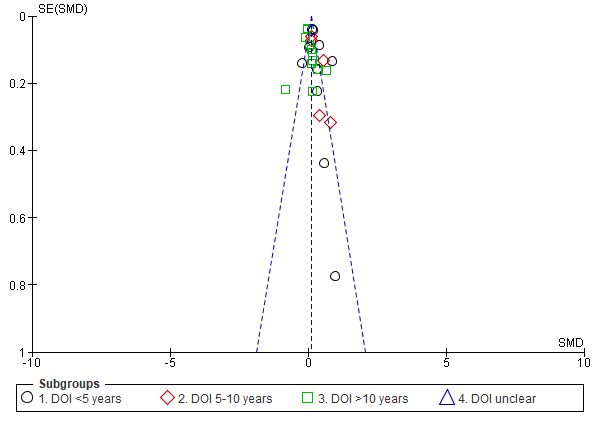

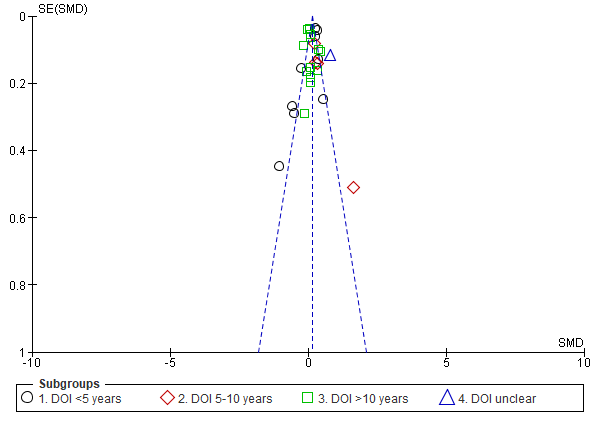

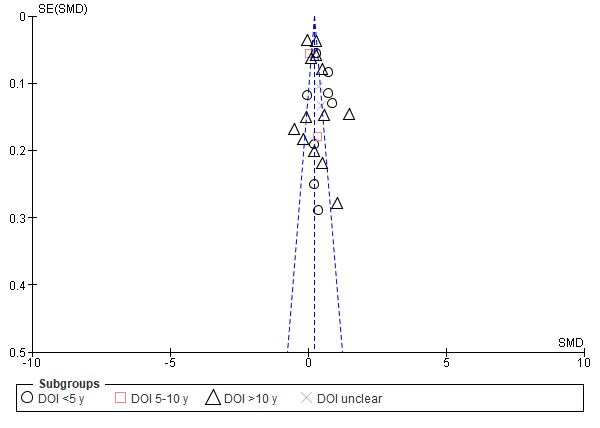

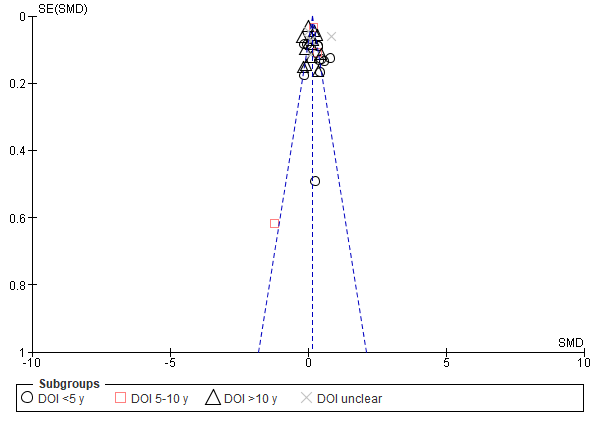
 Personal recovery Subjective quality of life**

**
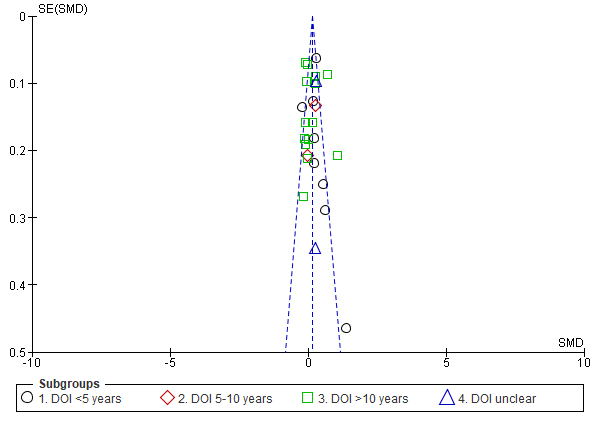

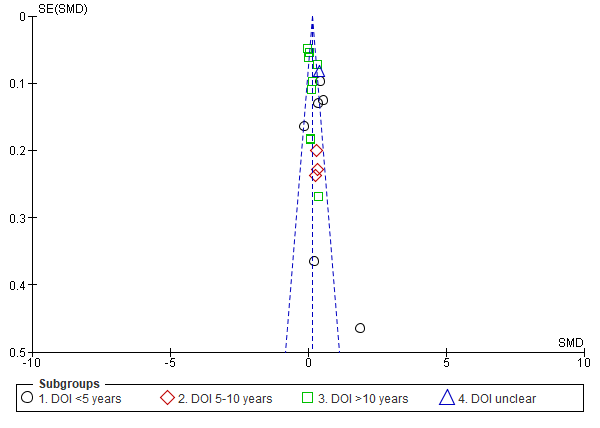
 Verbal memory Executive functioning**

**Processing speed Overall cognition**

# **Supplementary material 11.** Reference list of all included articles

S1. Addington, J., & Addington, D. (2000). Neurocognitive and social functioning in schizophrenia: a 2.5 year follow-up study. *Schizophrenia research*, *44*(1), 47-56.

S2. Aguilar, E. J., Corripio, I., García-Martí, G., Grasa, E., Martí-Bonmatí, L., Gómez-Ansón, B., ... & Turkington, D. (2018). Emotional fMR auditory paradigm demonstrates normalization of limbic hyperactivity after cognitive behavior therapy for auditory hallucinations. *Schizophrenia Research*, *193*, 304-312.

S3. Albus, M., Hubmann, W., Scherer, J., Dreikorn, B., Hecht, S., Sobizack, N., & Mohr, F. (2002). A prospective 2-year follow-up study of neurocognitive functioning in patients with first-episode schizophrenia. *European archives of psychiatry and clinical neuroscience*, *252*, 262-267.

S4. Albus, M., Hubmann, W., Mohr, F., Hecht, S., Hinterberger-Weber, P., Seitz, N. N., & Küchenhoff, H. (2006). Neurocognitive functioning in patients with first-episode schizophrenia: results of a prospective 5-year follow-up study. *European archives of psychiatry and clinical neuroscience*, *256*, 442-451.

S5. Alphs, L., Baker, P., Brown, B., Fu, D. J., Turkoz, I., & Nuechterlein, K. H. (2022). Evaluation of major treatment failure in patients with recent-onset schizophrenia or schizophreniform disorder: A post hoc analysis from the Disease Recovery Evaluation and Modification (DREaM) study. *Schizophrenia Research*, *248*, 58-63.

S6. Breier, A., Liffick, E., Hummer, T. A., Vohs, J. L., Yang, Z., Mehdiyoun, N. F., ... & Francis, M. M. (2018). Effects of 12-month, double-blind N-acetyl cysteine on symptoms, cognition and brain morphology in early phase schizophrenia spectrum disorders. *Schizophrenia Research*, *199*, 395-402.

S7. Buonocore, M., Spangaro, M., Bechi, M., Baraldi, M. A., Cocchi, F., Guglielmino, C., ... & Cavallaro, R. (2018). Integrated cognitive remediation and standard rehabilitation therapy in patients of schizophrenia: persistence after 5 years. *Schizophrenia research*, *192*, 335-339.

S8. Cai, Y., Gong, W., He, W., He, H., Hughes, J. P., Simoni, J., ... & Xu, D. R. (2022). Residual effect of Texting to promote medication adherence for villagers with schizophrenia in China: 18-month follow-up survey after the randomized controlled trial discontinuation. *JMIR mHealth and uHealth*, *10*(4), e33628.

S9. Cechnicki, A., & Bielańska, A. (2017). The influence of early psychosocial intervention on the long-term clinical outcomes of people suffering from schizophrenia. *Psychiatria polska*, *51*(1), 45-61.

S10. Chan, G. W. L., Ungvari, G. S., Shek, D. T. L., & Leung, J. P. (2003). Impact of deinstitutionalisation on the quality of life of Chinese patients with schizophrenia: A longitudinal pilot study. *Hong Kong Journal of Psychiatry*, *13*(4), 2-6.

S11. Chan, S. K. W., Hui, C. L. M., Chang, W. C., Lee, E. H. M., & Chen, E. Y. H. (2019). Ten-year follow up of patients with first-episode schizophrenia spectrum disorder from an early intervention service: predictors of clinical remission and functional recovery. *Schizophrenia research*, *204*, 65-71.

S12. Chan, S. K. W., So, H. C., Hui, C. L. M., Chang, W. C., Lee, E. H. M., Chung, D. W. S., ... & Chen, E. Y. H. (2015). 10-year outcome study of an early intervention program for psychosis compared with standard care service. *Psychological Medicine*, *45*(6), 1181-1193.

S13. Chanpattana, W., & Sackeim, H. A. (2010). Electroconvulsive therapy in treatment-resistant schizophrenia: prediction of response and the nature of symptomatic improvement. *The Journal of ECT*, *26*(4), 289-298.

S14. Chen, E. Y., Kwok, C. L., Au, J. W., Chen, R. Y., & Lau, B. S. (2000). Progressive deterioration of soft neurological signs in chronic schizophrenic patients. *Acta Psychiatrica Scandinavica*, *102*(5), 342-349.

S15. Chang, W. C., Hui, C. L., Tang, J. Y., Wong, G. H., Lam, M. M., Chan, S. K., & Chen, E. Y. (2011). Persistent negative symptoms in first-episode schizophrenia: a prospective three-year follow-up study. *Schizophrenia research*, *133*(1-3), 22-28.

S16. Chang, W. C., Hui, C. L. M., Tang, J. Y. M., Wong, G. H. Y., Chan, S. K. W., Lee, E. H. M., & Chen, E. Y. H. (2013). Impacts of duration of untreated psychosis on cognition and negative symptoms in first-episode schizophrenia: a 3-year prospective follow-up study. *Psychological Medicine*, *43*(9), 1883.

S17. Chang, W. C., Hui, C. L. M., Wong, G. H. Y., Chan, S. K. W., Lee, E. H. M., & Chen, E. Y. H. (2013). Symptomatic remission and cognitive impairment in first-episode schizophrenia: a prospective 3-year follow-up study. *The Journal of clinical psychiatry*, *74*(11), 1046-1053.

S18. Chang, W. C., Tang, J. Y. M., Hui, C. L. M., Wong, G. H. Y., Chan, S. K. W., Lee, E. H. M., & Chen, E. Y. H. (2013). The relationship of early premorbid adjustment with negative symptoms and cognitive functions in first-episode schizophrenia: a prospective three-year follow-up study. *Psychiatry research*, *209*(3), 353-360.

S19. Chang, W. C., Tang, J. Y. M., Hui, C. L. M., Chan, S. K. W., Lee, E. H. M., & Chen, E. Y. H. (2014). Clinical and cognitive predictors of vocational outcome in first-episode schizophrenia: a prospective 3 year follow-up study. *Psychiatry research*, *220*(3), 834-839.

S20. Chang, W. C., Hui, C. L. M., Chan, S. K. W., Lee, E. H. M., Wong, G. H. Y., & Chen, E. Y. H. (2014). Relationship between diminished expression and cognitive impairment in first-episode schizophrenia: a prospective three-year follow-up study. *Schizophrenia research*, *152*(1), 146-151.

S21. Chang, W. C., Ho, R. W. H., Tang, J. Y. M., Wong, C. S. M., Hui, C. L. M., Chan, S. K., ... & Chen, E. Y. (2019). Early-stage negative symptom trajectories and relationships with 13-year outcomes in first-episode nonaffective psychosis. *Schizophrenia bulletin*, *45*(3), 610-619.

S22. Chen, E. Y. H., Hui, C. L. M., Dunn, E. L. W., Miao, M. Y. K., Yeung, W. S., Wong, C. K., ... & Tang, W. N. (2005). A prospective 3-year longitudinal study of cognitive predictors of relapse in first-episode schizophrenic patients. *Schizophrenia Research*, *77*(1), 99-104.

S23. Chien, W. T., & Thompson, D. R. (2014). Effects of a mindfulness-based psychoeducation programme for Chinese patients with schizophrenia: 2-year follow-up. *The British Journal of Psychiatry*, *205*(1), 52-59.

S24. Chien, W. T., Bressington, D., Yip, A., & Karatzias, T. (2017). An international multi-site, randomized controlled trial of a mindfulness-based psychoeducation group programme for people with schizophrenia. *Psychological Medicine*, *47*(12), 2081-2096.

S25. Chien, W. T., Cheng, H. Y., McMaster, T. W., Yip, A. L., & Wong, J. C. (2019). Effectiveness of a mindfulness-based psychoeducation group programme for early-stage schizophrenia: An 18-month randomised controlled trial. *Schizophrenia research*, *212*, 140-149.

S26. Chien, W. T., Chow, K. M., Chong, Y. Y., Bressington, D., Choi, K. C., & Chan, C. W. H. (2020). The role of five facets of mindfulness in a mindfulness-based psychoeducation intervention for people with recent-onset psychosis on mental and psychosocial health outcomes. *Frontiers in psychiatry*, *11*, 177.

S27. Ciudad, A., Álvarez, E., Bobes, J., San, L., Polavieja, P., & Gilaberte, I. (2009). Remission in schizophrenia: Results from a 1-year follow-up observational study. *Schizophrenia research*, *108*(1-3), 214-222.

S28. Bobes, J., Ciudad, A., Álvarez, E., San, L., Polavieja, P., & Gilaberte, I. (2009). Recovery from schizophrenia: results from a 1-year follow-up observational study of patients in symptomatic remission. *Schizophrenia Research*, *115*(1), 58-66.

S29. Conley, R. R., Ascher-Svanum, H., Zhu, B., Faries, D. E., & Kinon, B. J. (2007). The burden of depressive symptoms in the long-term treatment of patients with schizophrenia. *Schizophrenia research*, *90*(1-3), 186-197.

S30. Carlsson, R., Nyman, H., Ganse, G., & Cullberg, J. (2006). Neuropsychological functions predict 1‐and 3‐year outcome in first‐episode psychosis. *Acta Psychiatrica Scandinavica*, *113*(2), 102-111.

S31. Cullberg, J., Levander, S., Holmqvist, R., Mattsson, M., & Wieselgren, I. M. (2002). One‐year outcome in first episode psychosis patients in the Swedish Parachute project. *Acta Psychiatrica Scandinavica*, *106*(4), 276-285.

S32. Dal Santo, F., Jarratt-Barnham, I., González-Blanco, L., García-Portilla, M. P., Bobes, J., & Fernández-Egea, E. (2020). Longitudinal effects of clozapine concentration and clozapine to N-desmethylclozapine ratio on cognition: A mediation model. *European Neuropsychopharmacology*, *33*, 158-163.

S33. Dellazizzo, L., Potvin, S., Phraxayavong, K., & Dumais, A. (2021). One-year randomized trial comparing virtual reality-assisted therapy to cognitive–behavioral therapy for patients with treatment-resistant schizophrenia. *npj Schizophrenia*, *7*(1), 9.

S34. Dixon, L. B., Goldman, H. H., Bennett, M. E., Wang, Y., McNamara, K. A., Mendon, S. J., ... & Essock, S. M. (2015). Implementing coordinated specialty care for early psychosis: the RAISE Connection Program. *Psychiatric Services*, *66*(7), 691-698.

S35. Humensky, J. L., Essock, S. M., & Dixon, L. B. (2017). Characteristics associated with the pursuit of work and school among participants in a treatment program for first episode of psychosis. *Psychiatric rehabilitation journal*, *40*(1), 108-112.

S36. Marino, L., Nossel, I., Choi, J. C., Nuechterlein, K., Wang, Y., Essock, S., ... & Dixon, L. (2015). The RAISE connection program for early psychosis: secondary outcomes and mediators and moderators of improvement. *The Journal of nervous and mental disease*, *203*(5), 365.

S37. Ekerholm, M., Waltersson, S. F., Fagerberg, T., Söderman, E., Terenius, L., Agartz, I., ... & Nyman, H. (2012). Neurocognitive function in long-term treated schizophrenia: a five-year follow-up study. *Psychiatry research*, *200*(2-3), 144-152.

S38. Evensen, S., Ueland, T., Lystad, J. U., Bull, H., Klungsøyr, O., Martinsen, E. W., & Falkum, E. (2017). Employment outcome and predictors of competitive employment at 2-year follow-up of a vocational rehabilitation programme for individuals with schizophrenia in a high-income welfare society. *Nordic journal of psychiatry*, *71*(3), 180-187.

S39. Fernández-Modamio, M., Gil-Sanz, D., Arrieta-Rodríguez, M., Santacoloma-Cabero, I., Bengochea-Seco, R., González-Fraile, E., & Muñiz, J. (2021). A randomized study on the efficacy of the Social Cognition Training Program-brief version in a sample of patients with schizophrenia. *Psychiatric Rehabilitation Journal*, *44*(1), 1.

S40. Fond, G., Bulzacka, E., Boucekine, M., Schürhoff, F., Berna, F., Godin, O., ... & Llorca, P. M. (2019). Machine learning for predicting psychotic relapse at 2 years in schizophrenia in the national FACE-SZ cohort. *Progress in Neuro-Psychopharmacology and Biological Psychiatry*, *92*, 8-18.

S41. Culbreth, A. J., Foti, D., Barch, D. M., Hajcak, G., & Kotov, R. (2018). Electrocortical responses to emotional stimuli in psychotic disorders: Comparing schizophrenia spectrum disorders and affective psychosis. *Frontiers in Psychiatry*, *9*, 586.

S42. Fett, A. K. J., Velthorst, E., Reichenberg, A., Ruggero, C. J., Callahan, J. L., Fochtmann, L. J., ... & Kotov, R. (2020). Long-term changes in cognitive functioning in individuals with psychotic disorders: findings from the Suffolk County Mental Health Project. *JAMA psychiatry*, *77*(4), 387-396.

S43. Foti, D. J., Kotov, R., Guey, L. T., & Bromet, E. J. (2010). Cannabis use and the course of schizophrenia: 10-year follow-up after first hospitalization. *American Journal of Psychiatry*, *167*(8), 987-993.

S44. Foti, D., Perlman, G., Hajcak, G., Mohanty, A., Jackson, F., & Kotov, R. (2016). Impaired error processing in late-phase psychosis: Four-year stability and relationships with negative symptoms. *Schizophrenia research*, *176*(2-3), 520-526.

S45. Kotov, R., Fochtmann, L., Li, K., Tanenberg-Karant, M., Constantino, E. A., Rubinstein, J., ... & Bromet, E. J. (2017). Declining clinical course of psychotic disorders over the two decades following first hospitalization: evidence from the Suffolk County Mental Health Project. *American Journal of Psychiatry*, *174*(11), 1064-1074.

S46. Fowler, D., Hodgekins, J., Garety, P., Freeman, D., Kuipers, E., Dunn, G., ... & Bebbington, P. E. (2012). Negative cognition, depressed mood, and paranoia: a longitudinal pathway analysis using structural equation modeling. *Schizophrenia bulletin*, *38*(5), 1063-1073.

S47. Fowler, D., Hodgekins, J., French, P., Marshall, M., Freemantle, N., McCrone, P., ... & Birchwood, M. (2018). Social recovery therapy in combination with early intervention services for enhancement of social recovery in patients with first-episode psychosis (SUPEREDEN3): a single-blind, randomised controlled trial. *The Lancet Psychiatry*, *5*(1), 41-50.

S48. Galderisi, S., Rucci, P., Mucci, A., Rossi, A., Rocca, P., Bertolino, A., ... & Brasso, C. (2020). The interplay among psychopathology, personal resources, context‐related factors and real‐life functioning in schizophrenia: stability in relationships after 4 years and differences in network structure between recovered and non‐recovered patients. *World Psychiatry*, *19*(1), 81-91.

S49. Ganella, E. P., Seguin, C., Pantelis, C., Whittle, S., Baune, B. T., Olver, J., ... & Bartholomeusz, C. F. (2018). Resting-state functional brain networks in first-episode psychosis: A 12-month follow-up study. *Australian & New Zealand Journal of Psychiatry*, *52*(9), 864-875.

S50. Gaughran, F., Stahl, D., Ismail, K., Greenwood, K., Atakan, Z., Gardner-Sood, P., ... & Lowe, P. (2017). Randomised control trial of the effectiveness of an integrated psychosocial health promotion intervention aimed at improving health and reducing substance use in established psychosis (IMPaCT). *BMC psychiatry*, *17*(1), 413.

S51. Godin, O., Fond, G., Bulzacka, E., Schürhoff, F., Boyer, L., Myrtille, A., ... & Zinetti-Bertschy, A. (2019). Validation and refinement of the clinical staging model in a French cohort of outpatient with schizophrenia (FACE-SZ). *Progress in Neuro-Psychopharmacology and Biological Psychiatry*, *92*, 226-234.

S52. Górna, K., Jaracz, K., Rybakowski, F., & Rybakowski, J. (2008). Determinants of objective and subjective quality of life in first-time-admission schizophrenic patients in Poland: a longitudinal study. *Quality of Life Research*, *17*, 237-247.

S53. Gorwood, P., Bouju, S., Deal, C., Gary, C., Delva, C., Lancrenon, S., & Llorca, P. M. (2019). Predictive factors of functional remission in patients with early to mid-stage schizophrenia treated by long acting antipsychotics and the specific role of clinical remission. *Psychiatry research*, *281*, 112560.

S54. Granholm, E., Link, P., Fish, S., Kraemer, H., & Jeste, D. (2010). Age-related practice effects across longitudinal neuropsychological assessments in older people with schizophrenia. *Neuropsychology*, *24*(5), 616.

S55. Grawe, R. W., Falloon, I. R. H., Widen, J. H., & Skogvoll, E. (2006). Two years of continued early treatment for recent‐onset schizophrenia: a randomised controlled study. *Acta Psychiatrica Scandinavica*, *114*(5), 328-336.

S56. Gumley, A. I., Bradstreet, S., Ainsworth, J., Allan, S., Alvarez-Jimenez, M., Birchwood, M., ... & Gleeson, J. (2022). Digital smartphone intervention to recognise and manage early warning signs in schizophrenia to prevent relapse: the EMPOWER feasibility cluster RCT. *Health Technology Assessment (Winchester, England)*, *26*(27), 1.

S57. Goghari, V. M., Harrow, M., Grossman, L. S., & Rosen, C. (2013). A 20-year multi-follow-up of hallucinations in schizophrenia, other psychotic, and mood disorders. *Psychological Medicine*, *43*(6), 1151-1160.

S58. Grossman, L. S., Harrow, M., Rosen, C., & Faull, R. (2006). Sex differences in outcome and recovery for schizophrenia and other psychotic and nonpsychotic disorders. *Psychiatric Services*, *57*(6), 844-850.

S59. Harrow, M., & Jobe, T. H. (2010). How frequent is chronic multiyear delusional activity and recovery in schizophrenia: A 20-year multi–follow-up. *Schizophrenia Bulletin*, *36*(1), 192-204.

S60. Harrow, M., Grossman, L. S., Jobe, T. H., & Herbener, E. S. (2005). Do patients with schizophrenia ever show periods of recovery? A 15-year multi-follow-up study. *Schizophrenia Bulletin*, *31*(3), 723-734.

S61. Harrow, M., Sands, J. R., Silverstein, M. L., & Goldberg, J. F. (1997). Course and outcome for schizophrenia versus other psychotic patients: a longitudinal study. *Schizophrenia Bulletin*, *23*(2), 287-303.

S62. Herbener, E. S., & Harrow, M. (2021). Course and symptom and functional correlates of passivity symptoms in schizophrenia: an 18-year multi-follow-up longitudinal study. *Psychological Medicine*, *51*(3), 503-510.

S63. Humpston, C., Harrow, M., & Rosen, C. (2020). Behind the opaque curtain: a 20-year longitudinal study of dissociative and first-rank symptoms in schizophrenia-spectrum psychoses, other psychoses and non-psychotic disorders. *Schizophrenia research*, *223*, 319-326.

S64. Harvey, P. D., Reichenberg, A., Bowie, C. R., Patterson, T. L., & Heaton, R. K. (2010). The course of neuropsychological performance and functional capacity in older patients with schizophrenia: influences of previous history of long-term institutional stay. *Biological psychiatry*, *67*(10), 933-939.

S65. Hayhurst, K. P., Drake, R. J., Massie, J. A., Dunn, G., Barnes, T. R. E., Jones, P. B., & Lewis, S. W. (2014). Improved quality of life over one year is associated with improved adherence in patients with schizophrenia. *European Psychiatry*, *29*(3), 191-196.

S66. Heeramun-Aubeeluck, A., Liu, N., Fischer, F., Huang, N., Chen, F., He, L., ... & Lu, Z. (2015). Effect of time and duration of untreated psychosis on cognitive and social functioning in Chinese patients with first-episode schizophrenia: A 1-year study. *Nordic journal of psychiatry*, *69*(4), 254-261.

S67. Heering, H. D., Janssens, M., Boyette, L. L., van Haren, N. E., & GROUP investigators. (2015). Remission criteria and functional outcome in patients with schizophrenia, a longitudinal study. *Australian & New Zealand Journal of Psychiatry*, *49*(3), 266-274.

S68. Hoff, A. L., Svetina, C., Shields, G., Stewart, J., & DeLisi, L. E. (2005). Ten year longitudinal study of neuropsychological functioning subsequent to a first episode of schizophrenia. *Schizophrenia research*, *78*(1), 27-34.

S69. Horan, W. P., Green, M. F., DeGroot, M., Fiske, A., Hellemann, G., Kee, K., ... & Sugar, C. A. (2012). Social cognition in schizophrenia, part 2: 12-month stability and prediction of functional outcome in first-episode patients. *Schizophrenia bulletin*, *38*(4), 865-872.

S70. Hui, C. L. M., Wong, A. K. H., Ho, E. C. N., Lam, B. S. T., Hui, P. W. M., Tao, T. J., ... & Chen, E. Y. H. (2023). Effectiveness and optimal duration of early intervention treatment in adult-onset psychosis: a randomized clinical trial. *Psychological Medicine*, *53*(6), 2339-2351.

S71. Ito, S., Nemoto, T., Tsujino, N., Ohmuro, N., Matsumoto, K., Matsuoka, H., ... & Ozawa, H. (2015). Differential impacts of duration of untreated psychosis (DUP) on cognitive function in first-episode schizophrenia according to mode of onset. *European Psychiatry*, *30*(8), 995-1001.

S72. Jørgensen, R., Zoffmann, V., Munk-Jørgensen, P., Buck, K. D., Jensen, S. O., Hansson, L., & Lysaker, P. H. (2015). Relationships over time of subjective and objective elements of recovery in persons with schizophreni. *Psychiatry research*, *228*(1), 14-19.

S73. Fulford, D., Piskulic, D., Addington, J., Kane, J. M., Schooler, N. R., & Mueser, K. T. (2018). Prospective relationships between motivation and functioning in recovery after a first episode of schizophrenia. *Schizophrenia bulletin*, *44*(2), 369-377.

S74. Kane, J. M., Robinson, D. G., Schooler, N. R., Mueser, K. T., Penn, D. L., Rosenheck, R. A., ... & Heinssen, R. K. (2016). Comprehensive versus usual community care for first-episode psychosis: 2-year outcomes from the NIMH RAISE early treatment program. *American Journal of Psychiatry*, *173*(4), 362-372.

S75. Kelly, D. L., Weiner, E., Ball, M. P., McMahon, R. P., Carpenter, W. T., & Buchanan, R. W. (2009). Remission in schizophrenia: the relationship to baseline symptoms and changes in symptom domains during a one-year study. *Journal of Psychopharmacology*, *23*(4), 436-441.

S76. Kim, S. H., Hwang, S. S., Jung, H. Y., Kim, Y., Ahn, Y. M., Chung, I. W., & Kim, Y. S. (2019). Differences between self-reported and clinician-rated evaluations of 1-year changes in auditory verbal hallucinations among schizophrenia patients. *Progress in Neuro-Psychopharmacology and Biological Psychiatry*, *95*, 109671.

S77. Klærke, L. R., Baandrup, L., Fagerlund, B., Ebdrup, B. H., Pantelis, C., Glenthøj, B. Y., & Nielsen, M. Ø. (2019). Diagnostic stability and long-term symptomatic and functional outcomes in first-episode antipsychotic-naïve patients with schizophrenia. *European Psychiatry*, *62*, 130-137.

S78. Koshiyama, D., Kirihara, K., Tada, M., Nagai, T., Koike, S., Suga, M., ... & Kasai, K. (2017). Duration and frequency mismatch negativity shows no progressive reduction in early stages of psychosis. *Schizophrenia research*, *190*, 32-38.

S79. Lasser, R. A., Bossie, C. A., Gharabawi, G. M., & Kane, J. M. (2005). Remission in schizophrenia: results from a 1-year study of long-acting risperidone injection. *Schizophrenia Research*, *77*(2-3), 215-227.

S80. Lee, M. A., Cola, P., Jayathilake, K., & Meltzer, H. Y. (2023). Long-Term Outcome of Clozapine in Treatment-Resistant Schizophrenia. *Journal of Clinical Psychopharmacology*, *43*(3), 211-219.

S81. Li, M., Deng, W., Das, T., Li, Y., Zhao, L., Ma, X., ... & Li, T. (2018). Neural substrate of unrelenting negative symptoms in schizophrenia: a longitudinal resting-state fMRI study. *European Archives of Psychiatry and Clinical Neuroscience*, *268*, 641-651.

S82. Li, M., Li, X., Das, T. K., Deng, W., Li, Y., Zhao, L., ... & Li, T. (2019). Prognostic utility of multivariate morphometry in schizophrenia. *Frontiers in psychiatry*, *10*, 245.

S83. Lindgren, M., Birling, H., Kieseppä, T., & Tuulio-Henriksson, A. (2020). Is cognitive performance associated with anxiety and depression in first-episode psychosis?. *Journal of Affective Disorders*, *263*, 221-227.

S84. Litman, R., Naber, D., Anta, L., Martínez, J., Filts, Y., & Correll, C. U. (2023). Personal and Social Functioning and Health-Related Quality of Life in Patients with Schizophrenia Treated with the Long-Acting Injectable Antipsychotic Risperidone ISM. *Neuropsychiatric Disease and Treatment*, 219-232.

S85. Liu, C. C., Hsieh, M. H., Chien, Y. L., Liu, C. M., Lin, Y. T., Hwang, T. J., & Hwu, H. G. (2023). Guided antipsychotic reduction to reach minimum effective dose (GARMED) in patients with remitted psychosis: a 2-year randomized controlled trial with a naturalistic cohort. *Psychological Medicine*, 1-9.

S86. Lopez-Morinigo, J. D., Martínez, A. S. E., Barrigón, M. L., Escobedo-Aedo, P. J., Ruiz-Ruano, V. G., Sánchez-Alonso, S., ... & David, A. S. (2023). A pilot 1-year follow-up randomised controlled trial comparing metacognitive training to psychoeducation in schizophrenia: effects on insight. *Schizophrenia*, *9*(1), 7.

S87. McGurk, S. R., Mueser, K. T., Harvey, P. D., LaPuglia, R., & Marder, J. (2003). Cognitive and symptom predictors of work outcomes for clients with schizophrenia in supported employment. *Psychiatric services*, *54*(8), 1129-1135.

S88. McNeely, H. E., Letts, L., Martin, M. L., & Strong, S. (2023). Participants’ Evaluation and Outcomes following Integration of Self-Management Support into Outpatient Schizophrenia Case Management. *International Journal of Environmental Research and Public Health*, *20*(4), 3035.

S89. Meade, N., Shi, L., Meehan, S. R., Weiss, C., & Ismail, Z. (2020). Efficacy and safety of brexpiprazole in patients with schizophrenia presenting with severe symptoms: Post-hoc analysis of short-and long-term studies. *Journal of Psychopharmacology*, *34*(8), 829-838.

S90. Meagher, D. J., Quinn, J. F., Bourke, S., Linehan, S., Murphy, P., Kinsella, A., ... & Waddington, J. L. (2004). Longitudinal assessment of psychopathological domains over late-stage schizophrenia in relation to duration of initially untreated psychosis: 3-year prospective study in a long-term inpatient population. *Psychiatry research*, *126*(3), 217-227.

S91. Gardsjord, E. S., Romm, K. L., Røssberg, J. I., Friis, S., Barder, H. E., Evensen, J., ... & Melle, I. (2018). Is going into stable symptomatic remission associated with a more positive development of life satisfaction? A 10-year follow-up study of first episode psychosis. *Schizophrenia research*, *193*, 364-369.

S92. Melle, I., Larsen, T. K., Haahr, U., Friis, S., Johannesen, J. O., Opjordsmoen, S., ... & McGlashan, T. (2008). Prevention of negative symptom psychopathologies in first-episode schizophrenia: two-year effects of reducing the duration of untreated psychosis. *Archives of general psychiatry*, *65*(6), 634-640.

S93. Moncrieff, J., Crellin, N., Stansfeld, J., Cooper, R., Marston, L., Freemantle, N., ... & Priebe, S. (2023). Antipsychotic dose reduction and discontinuation versus maintenance treatment in people with schizophrenia and other recurrent psychotic disorders in England (the RADAR trial): an open, parallel-group, randomised controlled trial. *The Lancet Psychiatry*, *10*(11), 848-859.

S94. Morrison, A. P., Law, H., Carter, L., Sellers, R., Emsley, R., Pyle, M., ... & Haddad, P. M. (2018). Antipsychotic drugs versus cognitive behavioural therapy versus a combination of both in people with psychosis: a randomised controlled pilot and feasibility study. *The Lancet Psychiatry*, *5*(5), 411-423.

S95. Na, E. J., Kang, N. I., Kim, M. Y., Cui, Y., Choi, H. E., Jung, A. J., & Chung, Y. C. (2016). Effects of community mental health service in subjects with early psychosis: One-year prospective follow up. *Community mental health journal*, *52*(6), 724-730.

S96. Najarian, D., Turkoz, I., Knight, R. K., Galderisi, S., Lamaison, H. F., Zalitacz, P., ... & Richarz, U. (2023). Long-term efficacy and safety of paliperidone 6-month formulation: an open-label 2-year extension of a 1-year double-blind study in adult participants with schizophrenia. *International Journal of Neuropsychopharmacology*, *26*(8), 537-544.

S97. Nakamura, R., Asami, T., Yoshimi, A., Kato, D., Fujita, E., Takaishi, M., ... & Hirayasu, Y. (2019). Clinical and brain structural effects of the Illness Management and Recovery program in middle‐aged and older patients with schizophrenia. *Psychiatry and clinical neurosciences*, *73*(12), 731-737.

S98. Neill, E., Rossell, S. L., Yolland, C., Meyer, D., Galletly, C., Harris, A., ... & Castle, D. J. (2022). N-acetylcysteine (NAC) in schizophrenia resistant to clozapine: a double-blind, randomized, placebo-controlled trial targeting negative symptoms. *Schizophrenia Bulletin*, *48*(6), 1263-1272.

S99. Albert, N., Glenthøj, L. B., Melau, M., Jensen, H., Hjorthøj, C., & Nordentoft, M. (2017). Course of illness in a sample of patients diagnosed with a schizotypal disorder and treated in a specialized early intervention setting. Findings from the 3.5 year follow-up of the OPUS II study. *Schizophrenia Research*, *182*, 24-30.

S100. Albert, N., Bertelsen, M., Thorup, A., Petersen, L., Jeppesen, P., Le Quack, P., ... & Nordentoft, M. (2011). Predictors of recovery from psychosis: analyses of clinical and social factors associated with recovery among patients with first-episode psychosis after 5 years. *Schizophrenia research*, *125*(2-3), 257-266.

S101. Clausen, L., Hjorthøj, C. R., Thorup, A., Jeppesen, P., Petersen, L., Bertelsen, M., & Nordentoft, M. (2014). Change in cannabis use, clinical symptoms and social functioning among patients with first-episode psychosis: a 5-year follow-up study of patients in the OPUS trial. *Psychological Medicine*, *44*(1), 117-126.

S102. Hansen, H. G., Starzer, M., Nilsson, S. F., Hjorthøj, C., Albert, N., & Nordentoft, M. (2023). Clinical recovery and long-term association of specialized early intervention services vs treatment as usual among individuals with first-episode schizophrenia spectrum disorder: 20-year follow-up of the OPUS trial. *JAMA psychiatry*, *80*(4), 371-379.

S103. Nordentoft, M., Thorup, A., Petersen, L., Øhlenschlæger, J., Melau, M., Christensen, T. Ø., ... & Jeppesen, P. (2006). Transition rates from schizotypal disorder to psychotic disorder for first-contact patients included in the OPUS trial. A randomized clinical trial of integrated treatment and standard treatment. *Schizophrenia Research*, *83*(1), 29-40.

S104. Oh, S., Kim, M., Kim, T., Lee, T. Y., & Kwon, J. S. (2020). Resting-state functional connectivity of the striatum predicts improvement in negative symptoms and general functioning in patients with first-episode psychosis: A 1-year naturalistic follow-up study. *Australian & New Zealand Journal of Psychiatry*, *54*(5), 509-518.

S105. Okin, R. L., Borus, J. F., Baer, L., & Jones, A. L. (1995). Long-term outcome of state hospital patients discharged into structured community residential settings. *Psychiatric Services, 46*(1), 73-78.

S106. Oribe, N., Hirano, Y., Kanba, S., Del Re, E., Seidman, L., Mesholam-Gately, R., ... & Niznikiewicz, M. (2015). Progressive reduction of visual P300 amplitude in patients with first-episode schizophrenia: an ERP study. *Schizophrenia bulletin*, *41*(2), 460-470.

S107. Oribe, N., Hirano, Y., Del Re, E., Seidman, L. J., Mesholam-Gately, R. I., Woodberry, K. A., ... & Spencer, K. M. (2019). Progressive reduction of auditory evoked gamma in first episode schizophrenia but not clinical high risk individuals. *Schizophrenia research*, *208*, 145-152.

S108. Ortega, L., Montalvo, I., Monseny, R., Burjales‐Martí, M. D., Martorell, L., Sanchez‐Gistau, V., ... & Labad, J. (2021). Perceived stress, social functioning and quality of life in first‐episode psychosis: A 1‐year follow‐up study. *Early Intervention in Psychiatry*, *15*(6), 1542-1550.

S109. Ozawa, C., Bies, R. R., Pillai, N., Suzuki, T., Mimura, M., & Uchida, H. (2019). Model-guided antipsychotic dose reduction in schizophrenia: a pilot, single-blind randomized controlled trial. *Journal of clinical psychopharmacology*, *39*(4), 329-335.

S110. Prouteau, A., Verdoux, H., Briand, C., Lesage, A., Lalonde, P., Nicole, L., ... & Stip, E. (2005). Cognitive predictors of psychosocial functioning outcome in schizophrenia: a follow-up study of subjects participating in a rehabilitation program. *Schizophrenia Research*, *77*(2-3), 343-353.

S111. Bowie, C. R., & Harvey, P. D. (2008). Communication abnormalities predict functional outcomes in chronic schizophrenia: Differential associations with social and adaptive functions. *Schizophrenia research*, *103*(1-3), 240-247.

S112. Friedman, J. I., Harvey, P. D., McGurk, S. R., White, L., Parrella, M., Raykov, T., ... & Davis, K. L. (2002). Correlates of change in functional status of institutionalized geriatric schizophrenic patients: focus on medical comorbidity. *American Journal of Psychiatry*, *159*(8), 1388-1394.

S113. Harvey, P. D., Lombardi, J., Leibman, M., White, L., Parrella, M., Powchik, P., & Davidson, M. (1996). Cognitive impairment and negative symptoms in geriatric chronic schizophrenic patients: a follow-up study. *Schizophrenia Research*, *22*(3), 223-231

S114. Harvey, P. D., Parrella, M., White, L., Mohs, R. C., Davidson, M., & Davis, K. L. (1999). Convergence of cognitive and adaptive decline in late-life schizophrenia. *Schizophrenia research*, *35*(1), 77-84.

S115. Harvey, P. D., Friedman, J. I., Bowie, C., Reichenberg, A., McGurk, S. R., Parrella, M., ... & Davis, K. L. (2006). Validity and stability of performance-based estimates of premorbid educational functioning in older patients with schizophrenia. *Journal of clinical and experimental neuropsychology*, *28*(2), 178-192.

S116. McGurk, S. R., Moriarty, P. J., Harvey, P. D., Parrella, M., White, L., & Davis, K. L. (2000). The longitudinal relationship of clinical symptoms, cognitive functioning, and adaptive life in geriatric schizophrenia. *Schizophrenia Research*, *42*(1), 47-55.

S117. Putnam, K. M., & Harvey, P. D. (2000). Cognitive impairment and enduring negative symptoms: a comparative study of geriatric and nongeriatric schizophrenia patients. *Schizophrenia bulletin*, *26*(4), 867-878.

S118. Pelayo-Terán, J. M., Gajardo-Galán, V., Gómez-Revuelta, M., de la Foz, V. O. G., Ayesa-Arriola, R., Tabarés-Seisdedos, R., & Crespo-Facorro, B. (2018). Duration of active psychosis and functional outcomes in first-episode non-affective psychosis. *European Psychiatry*, *52*, 29-37.

S119. Rodríguez-Sánchez, J. M., Pérez-Iglesias, R., González-Blanch, C., Pelayo-Terán, J. M., Mata, I., Martínez, O., ... & Crespo-Facorro, B. (2008). 1-year follow-up study of cognitive function in first-episode non-affective psychosis. *Schizophrenia Research*, *104*(1-3), 165-174.

S120. Rodríguez-Sánchez, J. M., Ayesa-Arriola, R., Pérez-Iglesias, R., Periañez, J. A., Martinez-Garcia, O., Gomez-Ruiz, E., ... & Crespo-Facorro, B. (2013). Course of cognitive deficits in first episode of non-affective psychosis: a 3-year follow-up study. *Schizophrenia research*, *150*(1), 121-128.

S121. Setién‐Suero, E., Neergaard, K., Ortiz‐García de la Foz, V., Suárez‐Pinilla, P., Martínez‐García, O., Crespo‐Facorro, B., & Ayesa‐Arriola, R. (2019). Stopping cannabis use benefits outcome in psychosis: findings from 10‐year follow‐up study in the PAFIP‐cohort. *Acta Psychiatrica Scandinavica*, *140*(4), 349-359.

S122. Rossi, A., Bagala, A., Del Curatolo, V., Scapati, F., Bernareggi, M. M., & Giustra, M. G. (2009). Remission in schizophrenia: one‐year Italian prospective study of risperidone long‐acting injectable (RLAI) in patients with schizophrenia or schizoaffective disorder. *Human Psychopharmacology: Clinical and Experimental*, *24*(7), 574-583.

S123. Rowland, T., Birchwood, M., Singh, S., Freemantle, N., Everard, L., Jones, P., ... & Thompson, A. (2019). Short-term outcome of first episode delusional disorder in an early intervention population. *Schizophrenia Research*, *204*, 72-79.

S124. Drosos, P., Brønnick, K., Joa, I., Johannessen, J. O., Johnsen, E., Kroken, R. A., ... & Larsen, T. K. (2020). One-year outcome and adherence to pharmacological guidelines in first-episode schizophrenia: results from a consecutive cohort study. *Journal of Clinical Psychopharmacology*, *40*(6), 534-540.

S125. Rund, B. R., Melle, I., Friis, S., Johannessen, J. O., Larsen, T. K., Midbøe, L. J., ... & McGlashan, T. (2007). The course of neurocognitive functioning in first-episode psychosis and its relation to premorbid adjustment, duration of untreated psychosis, and relapse. *Schizophrenia research*, *91*(1-3), 132-140.

S126. Kida, H., Niimura, H., Nemoto, T., Ryu, Y., Sakuma, K., Mimura, M., & Mizuno, M. (2020). Community transition at younger ages contributes to good cognitive function outcomes in long‐term hospitalized patients with schizophrenia spectrum disorder: A 15‐year follow‐up study with group‐based trajectory modeling. *Psychiatry and Clinical Neurosciences*, *74*(2), 105-111.

S127. Kumazaki, H., Kobayashi, H., Niimura, H., Kobayashi, Y., Ito, S., Nemoto, T., ... & Mizuno, M. (2012). Lower subjective quality of life and the development of social anxiety symptoms after the discharge of elderly patients with remitted schizophrenia: a 5-year longitudinal study. *Comprehensive Psychiatry*, *53*(7), 946-951.

S128. Nemoto, T., Niimura, H., Ryu, Y., Sakuma, K., & Mizuno, M. (2014). Long-term course of cognitive function in chronically hospitalized patients with schizophrenia transitioning to community-based living. *Schizophrenia research*, *155*(1-3), 90-95.

S129. Ryu, Y., Mizuno, M., Sakuma, K., Munakata, S., Takebayashi, T., Murakami, M., ... & Kashima, H. (2006). Deinstitutionalization of long-stay patients with schizophrenia: the 2-year social and clinical outcome of a comprehensive intervention program in Japan. *Australian & New Zealand Journal of Psychiatry*, *40*(5), 462-470.

S130. Luther, L., Fukui, S., Firmin, R. L., McGuire, A. B., White, D. A., Minor, K. S., & Salyers, M. P. (2015). Expectancies of success as a predictor of negative symptoms reduction over 18 months in individuals with schizophrenia. *Psychiatry research*, *229*(1-2), 505-510.

S131. Salyers, M. P., McGuire, A. B., Kukla, M., Fukui, S., Lysaker, P. H., & Mueser, K. T. (2014). A randomized controlled trial of illness management and recovery with an active control group. *Psychiatric services*, *65*(8), 1005-1011.

S132. Schmidt, S. J., Lange, M., Schöttle, D., Karow, A., Schimmelmann, B. G., & Lambert, M. (2018). Negative symptoms, anxiety, and depression as mechanisms of change of a 12-month trial of assertive community treatment as part of integrated care in patients with first-and multi-episode schizophrenia spectrum disorders (ACCESS I trial). *European archives of psychiatry and clinical neuroscience*, *268*, 593-602.

S133. McCreadie, R. G., Wiles, D. H., Grant, S. M., Moore, J. W., Crocket, G. T., Mahmood, Z., ... & Todd, N. A. (1988). The Scottish First Episode Schizophrenia Study V. One-year Follow-up: The Scottish Schizophrenia Research Group. *The British Journal of Psychiatry*, *152*(4), 470-476.

S134. McCreadie, R. G., Wiles, D. H., Livingston, M. G., Watt, J. A., Greene, J. G., Kershaw, P. W., ... & Loudon, J. (1992). The Scottish first episode schizophrenia study: VIII. Five-year follow-up: Clinical and psychosocial findings. *The British Journal of Psychiatry*, *161*(4), 496-500.

S135. Scottish Schizophrenia Research Group, McCreadie, R. G., Wiles, D., Grant, S., Crockett, G. T., Mahmood, Z., ... & Batchelor, D. (1989). The Scottish first episode schizophrenia study: VII. Two‐year follow‐up. *Acta Psychiatrica Scandinavica*, *80*(6), 597-602.

S136. She, S., Deng, Y., Chen, Y., Wu, C., Yi, W., Lu, X., ... & Xiao, D. (2017). Two-stage integrated care versus antipsychotic medication alone on outcomes of schizophrenia: One-year randomized controlled trial and follow-up. *Psychiatry Research*, *254*, 164-172.

S137. Siegel, S. J., Irani, F., Brensinger, C. M., Kohler, C. G., Bilker, W. B., Ragland, J. D., ... & Gur, R. E. (2006). Prognostic variables at intake and long-term level of function in schizophrenia. *American Journal of Psychiatry*, *163*(3), 433-441.

S138. Sikira, H., Janković, S., Slatina, M. S., Muhić, M., Sajun, S., Priebe, S., & Kulenović, A. D. (2021). The effectiveness of volunteer befriending for improving the quality of life of patients with schizophrenia in Bosnia and Herzegovina–an exploratory randomised controlled trial. *Epidemiology and Psychiatric Sciences*, *30*, e48.

S139. Smith, T. E., Hull, J. W., Huppert, J. D., & Silverstein, S. M. (2002). Recovery from psychosis in schizophrenia and schizoaffective disorder: symptoms and neurocognitive rate-limiters for the development of social behavior skills. *Schizophrenia Research*, *55*(3), 229-237.

S140. Sommer, I. E., Gangadin, S. S., de Witte, L. D., Koops, S., Van Baal, C., Bahn, S., ... & Begemann, M. J. (2021). Simvastatin augmentation for patients with early-phase schizophrenia-spectrum disorders: a double-blind, randomized placebo-controlled trial. *Schizophrenia Bulletin*, *47*(4), 1108-1115.

S141. Stouten, L. H., Veling, W., Laan, W., Van der Helm, M., & Van der Gaag, M. (2014). Psychotic symptoms, cognition and affect as predictors of psychosocial problems and functional change in first-episode psychosis. *Schizophrenia research*, *158*(1-3), 113-119.

S142. Stouten, L. H., Veling, W., Laan, W., & Van der Gaag, M. (2019). Psychopathology, cognition and outcome in Dutch and immigrant first‐episode psychosis patients. *Early intervention in psychiatry*, *13*(3), 646-656.

S143. Sweeney, J. A., Haas, G. L., Keilp, J. G., & Long, M. (1991). Evaluation of the stability of neuropsychological functioning after acute episodes of schizophrenia: one-year followup study. *Psychiatry Research*, *38*(1), 63-76.

S144. Balanzá-Martínez, V., Tabarés-Seisdedos, R., Selva-Vera, G., Martínez-Arán, A., Torrent, C., Salazar-Fraile, J., ... & Gómez-Beneyto, M. (2005). Persistent cognitive dysfunctions in bipolar I disorder and schizophrenic patients: a 3-year follow-up study. *Psychotherapy and psychosomatics*, *74*(2), 113-119.

S145. Tabarés-Seisdedos, R., Balanzá-Martínez, V., Sánchez-Moreno, J., Martinez-Aran, A., Salazar-Fraile, J., Selva-Vera, G., ... & Vieta, E. (2008). Neurocognitive and clinical predictors of functional outcome in patients with schizophrenia and bipolar I disorder at one-year follow-up. *Journal of affective disorders*, *109*(3), 286-299.

S146. Tabo, A., Aydın, E., Yumrukçal, H., Yiğit, S., Uzun, U. E., & Karamustafalıoğlu, O. (2017). Longer duration of untreated psychosis hinders improvement in treatment of chronic schizophrenia: community based early intervention is an evidence based option. *Community mental health journal*, *53*(8), 929-935.

S147. Mohn, C., & Torgalsbøen, A. K. (2018). Details of attention and learning change in first-episode schizophrenia. *Psychiatry research*, *260*, 324-330.

S148. Torgalsbøen, A. K., Mohn, C., Czajkowski, N., & Rund, B. R. (2015). Relationship between neurocognition and functional recovery in first-episode schizophrenia: results from the second year of the Oslo multi-follow-up study. *Psychiatry research*, *227*(2-3), 185-191.

S149. Ergül, C., & Üçok, A. (2015). Negative symptom subgroups have different effects on the clinical course of schizophrenia after the first episode: a 24-month follow up study. *European Psychiatry*, *30*(1), 14-19.

S150. Üçok, A., Serbest, S., & Kandemir, P. E. (2011). Remission after first-episode schizophrenia: results of a long-term follow-up. *Psychiatry research*, *189*(1), 33-37.

S151. Üçok, A., & Ergül, C. (2014). Persistent negative symptoms after first episode schizophrenia: a 2-year follow-up study. *Schizophrenia research*, *158*(1-3), 241-246.

S152. Usui, K., Kirihara, K., Tada, M., Fujioka, M., Koshiyama, D., Tani, M., ... & Kasai, K. (2022). The association between clinical symptoms and later subjective quality of life in individuals with ultra‐high risk for psychosis and recent‐onset psychotic disorder: A longitudinal investigation. *Psychiatry and Clinical Neurosciences*, *76*(11), 552-559.

S153. Veerman, S., Schulte, P., Deijen, J. B., & de Haan, L. (2016). Adjunctive memantine in clozapine-treated refractory schizophrenia: A one-year extension study. *European Psychiatry*, *33*(S1), S108-S109.

S154. Veijola, J., Guo, J. Y., Moilanen, J. S., Jääskeläinen, E., Miettunen, J., Kyllönen, M., ... & Kiviniemi, V. (2014). Longitudinal changes in total brain volume in schizophrenia: relation to symptom severity, cognition and antipsychotic medication. *PloS one*, *9*(7), e101689.

S155. Whitehorn, D., Brown, J., Richard, J., Rui, Q., & Kopala, L. (2002). Multiple dimensions of recovery in early psychosis. *International Review of Psychiatry*, *14*(4), 273-283.

S156. Wilson-d’Almeida, K., Karrow, A., Bralet, M. C., Bazin, N., Hardy-Baylé, M. C., & Falissard, B. (2013). In patients with schizophrenia, symptoms improvement can be uncorrelated with quality of life improvement. *European psychiatry*, *28*(3), 185-189.

S157. Wittorf, A., Klingberg, S., & Wiedemann, G. (2004). Secondary verbal memory: a potential endophenotype of schizophrenia. *Journal of psychiatric research*, *38*(6), 601-612.

S158. Wittorf, A., Wiedemann, G., Buchkremer, G., & Klingberg, S. (2008). Prediction of community outcome in schizophrenia 1 year after discharge from inpatient treatment. *European Archives of Psychiatry and Clinical Neuroscience*, *258*(1), 48-58.

S159. Wojtalik, J. A., Mesholam-Gately, R. I., Hogarty, S. S., Greenwald, D. P., Litschge, M. Y., Sandoval, L. R., ... & Eack, S. M. (2022). Confirmatory efficacy of cognitive enhancement therapy for early schizophrenia: results from a multisite randomized trial. *Psychiatric Services*, *73*(5), 501-509.

S160. Wunderink, L., Sytema, S., Nienhuis, F. J., & Wiersma, D. (2009). Clinical recovery in first-episode psychosis. *Schizophrenia Bulletin*, *35*(2), 362-369.

S161. Drake, R. E., McHugo, G. J., Xie, H., Fox, M., Packard, J., & Helmstetter, B. (2006). Ten-year recovery outcomes for clients with co-occurring schizophrenia and substance use disorders. *Schizophrenia bulletin*, *32*(3), 464-473.

S162. Xie, H., McHugo, G. J., Helmstetter, B. S., & Drake, R. E. (2005). Three-year recovery outcomes for long-term patients with co-occurring schizophrenic and substance use disorders. *Schizophrenia Research*, *75*(2-3), 337-348.

S163. Liu, K. C., Chan, R. C., Chan, K. K., Tang, J. Y., Chiu, C. P., Lam, M. M., ... & Chen, E. Y. (2011). Executive function in first-episode schizophrenia: a three-year longitudinal study of an ecologically valid test. *Schizophrenia research*, *126*(1-3), 87-92.

S164. Xu, J. Q., Hui, C. L. M., Longenecker, J., Lee, E. H. M., Chang, W. C., Chan, S. K. W., & Chen, E. Y. H. (2014). Executive function as predictors of persistent thought disorder in first-episode schizophrenia: a one-year follow-up study. *Schizophrenia research*, *159*(2-3), 465-470.

S165. Zäske, H., Linden, M., Degner, D., Jockers-Scherübl, M., Klingberg, S., Klosterkötter, J., ... & Gaebel, W. (2019). Stigma experiences and perceived stigma in patients with first-episode schizophrenia in the course of 1 year after their first in-patient treatment. *European archives of psychiatry and clinical neuroscience*, *269*, 459-468.

S166. Zhu, X., Fan, H., Zou, Y., Tan, Y., Yang, F., Wang, Z., ... & Wykes, T. (2022). Computerized or manual? Long term effects of cognitive remediation on schizophrenia. *Schizophrenia Research*, *239*, 47-54.
